# Genome-Wide Association Study Implicates Chromosome 9q21.31 as a Susceptibility Locus for Asthma in Mexican Children

**DOI:** 10.1371/journal.pgen.1000623

**Published:** 2009-08-28

**Authors:** Dana B. Hancock, Isabelle Romieu, Min Shi, Juan-Jose Sienra-Monge, Hao Wu, Grace Y. Chiu, Huiling Li, Blanca Estela del Rio-Navarro, Saffron A. G. Willis-Owens, Scott T. Weiss, Benjamin A. Raby, Hong Gao, Celeste Eng, Rocio Chapela, Esteban G. Burchard, Hua Tang, Patrick F. Sullivan, Stephanie J. London

**Affiliations:** 1 Epidemiology Branch, National Institute of Environmental Health Sciences, National Institutes of Health, Department of Health and Human Services, Research Triangle Park, North Carolina, United States of America; 2 Instituto Nacional de salud Publica, Cuernavaca, Mexico; 3 Biostatistics Branch, National Institute of Environmental Health Sciences, National Institutes of Health, Department of Health and Human Services, Research Triangle Park, North Carolina, United States of America; 4 Hospital Infantil de Mexico Federico Gomez, Mexico City, Mexico; 5 Laboratory of Respiratory Biology, National Institute of Environmental Health Sciences, National Institutes of Health, Department of Health and Human Services, Research Triangle Park, North Carolina, United States of America; 6 Westat, Research Triangle Park, North Carolina, United States of America; 7 Molecular Genetics, National Heart and Lung Institute, Imperial College, London, United Kingdom; 8 Channing Laboratory, Brigham and Women's Hospital and Harvard Medical School, Boston, Massachusetts, United States of America; 9 Department of Genetics, Stanford University, Stanford, California, United States of America; 10 Department of Biopharmaceutical Sciences, University of California San Francisco, San Francisco, California, United States of America; 11 Instituto Nacional de Enfermedades Respiratorias, Mexico City, Mexico; 12 Department of Genetics, University of North Carolina at Chapel Hill, Chapel Hill, North Carolina, United States of America; The University of Queensland, Australia

## Abstract

Many candidate genes have been studied for asthma, but replication has varied. Novel candidate genes have been identified for various complex diseases using genome-wide association studies (GWASs). We conducted a GWAS in 492 Mexican children with asthma, predominantly atopic by skin prick test, and their parents using the Illumina HumanHap 550 K BeadChip to identify novel genetic variation for childhood asthma. The 520,767 autosomal single nucleotide polymorphisms (SNPs) passing quality control were tested for association with childhood asthma using log-linear regression with a log-additive risk model. Eleven of the most significantly associated GWAS SNPs were tested for replication in an independent study of 177 Mexican case–parent trios with childhood-onset asthma and atopy using log-linear analysis. The chromosome 9q21.31 SNP rs2378383 (p = 7.10×10^−6^ in the GWAS), located upstream of *transducin-like enhancer of split 4 (TLE4)*, gave a p-value of 0.03 and the same direction and magnitude of association in the replication study (combined p = 6.79×10^−7^). Ancestry analysis on chromosome 9q supported an inverse association between the rs2378383 minor allele (G) and childhood asthma. This work identifies chromosome 9q21.31 as a novel susceptibility locus for childhood asthma in Mexicans. Further, analysis of genome-wide expression data in 51 human tissues from the Novartis Research Foundation showed that median GWAS significance levels for SNPs in genes expressed in the lung differed most significantly from genes not expressed in the lung when compared to 50 other tissues, supporting the biological plausibility of our overall GWAS findings and the multigenic etiology of childhood asthma.

## Author Summary

Asthma is a leading chronic childhood disease with a presumed strong genetic component, but no genes have been definitely shown to influence asthma development. Few genetic studies of asthma have included Hispanic populations. Here, we conducted a genome-wide association study of asthma in 492 Mexican children with asthma, predominantly atopic by skin prick test, and their parents to identify novel genetic variation for childhood asthma. We implicated several polymorphisms in or near *TLE4* on chromosome 9q21.31 (a novel candidate region for childhood asthma) and replicated one polymorphism in an independent study of childhood-onset asthmatics with atopy and their parents of Mexican ethnicity. Hispanics have differing proportions of Native American, European, and African ancestries, and we found less Native American ancestry than expected at chromosome 9q21.31. This suggests that chromosome 9q21.31 may underlie ethnic differences in childhood asthma and that future replication would be most effective in populations with Native American ancestry. Analysis of publicly available genome-wide expression data revealed that association signals in genes expressed in the lung differed most significantly from genes not expressed in the lung when compared to 50 other tissues, supporting the biological plausibility of the overall GWAS findings and the multigenic etiology of asthma.

## Introduction

Asthma (OMIM 600807) is a leading chronic childhood disease with prevalence rates reaching a historically high level (8.9%) in the United States and continuing to increase in many countries worldwide [1],[2]. Asthma is characterized by airway inflammation and bronchoconstriction leading to airflow obstruction, but the mechanisms leading to asthma development remain unknown. Genetic risk factors likely play a central role in asthma development. Twin studies support a strong genetic component to asthma (especially childhood asthma) with heritability estimates suggesting that 48–79% of asthma risk is attributable to genetic risk factors [3]. In an effort to localize disease susceptibility genes, genome-wide linkage studies have identified at least 20 linkage regions potentially harboring disease genes [4], and over 100 positional and biological candidate genes have been tested for association with asthma [3]. However, no genes have been definitely shown to influence this complex disease.

Genome-wide association studies (GWASs) have emerged as a powerful approach for identifying novel candidate genes for common, complex diseases. In the first asthma GWAS, using 307,328 single nucleotide polymorphisms (SNPs), Moffatt et al. found highly statistically significant associations of SNPs in adjacent genes *ORM1-like (S. cerevisiae)* (*ORMDL3*; OMIM 610075) and *gasdermin B* (*GSDMB* or *GSDML*; OMIM 611221) with risk of childhood asthma in German and British populations [5]. Meta-analysis of the Moffatt et al. study and five subsequent replication studies, including our own study, supports the association of *ORMDL3* and *GSDML* SNPs with risk for childhood asthma across various populations [6]. More recently, using 518,230 SNPs, Himes et al. implicated SNPs in *phosphodiesterase 4D, cAMP-specific (phosphodiesterase E3 dunce homolog, Drosophila)* (*PDE4D*; OMIM 600129) with risk of asthma in whites from the United States and replicated this finding in two other white populations [7]. Using only 97,112 SNPs, Choudhry et al. implicated chromosome 5q23 SNPs for association with asthma in Puerto Ricans [8], but no other Puerto Rican cohorts are available for replication.

Few genetic studies of asthma have included Hispanic populations, and replication of positive genetic findings is scarce across Hispanic groups. Hispanics have differing proportions of Native American, European, and African ancestries. This admixture introduces special considerations (such as controlling for population stratification in association studies), but admixture in Hispanic populations also provides a unique opportunity to use ancestry analysis to evaluate our genetic association findings [9],[10].

Mexico City is one of the most polluted cities in the world, and its inhabitants experience chronic ozone exposure, which has been linked to asthma development in children and adults and asthma exacerbations [11]–[13]. We conducted a GWAS to identify novel candidate susceptibility genes associated with childhood asthma in case-parent trios from Mexico City and tested the most significantly associated SNPs in an independent study of trios of Mexican ethnicity. GWAS findings were then examined in the context of ancestry analysis and genome-wide expression data to provide supportive evidence for associations with childhood asthma.

## Results

In the GWAS, there were more male (58.7%) than female (41.3%) children with asthma, and the mean age at enrollment was 9.0±2.4 years (Table 1). The replication study had a similar distribution of males (59.7%) to females (40.3%), but there was an older mean age at enrollment distribution of 13.4±5.4 years. All GWAS and replication study subjects with asthma were clinically diagnosed before age 18.

**Table 1:** Demographic and clinical characteristics of the 492 children with asthma.

Characteristic	Category	N (%) or Mean (SD^a^)
Age at enrollment in years, mean (SD^a^)		9.0 (2.4)
Sex, N (%)	Male	289 (58.7)
	Female	203 (41.3)
Asthma severity, N (%)^b^	Mild	339 (72.3)
	Moderate/Severe	130 (27.7)
Skin test positivity (out of 24 aeroallergens), N (%)^b^	0 allergens	37 (8.3)
	1–4 allergen(s)	179 (40.2)
	≥5 allergens	229 (51.5)
Percent predicted FEV_1_, mean (SD^a^)		90.5 (16.8)
Parental smoking, N (%)	Mother smoked during pregnancy	23 (4.8)
	Current smoking parent	253 (52.1)
Residential ambient ozone exposure (annual average of the daily maximum 8 hour averages)^b^	≤67 ppb	199 (48.2)
	>67 ppb	214 (51.8)

a SD, standard deviation.

b Numbers do not sum to 492 because of missing data. Percentages are based on non-missing data for each characteristic.

Additional demographic and clinical characteristics of the 492 children with asthma evaluated in the GWAS are presented in Table 1. More children had mild asthma (72.3%) than moderate to severe asthma (27.7%). Among the 445 children with skin prick test data, nearly all (91.7%) were atopic based on having a positive skin test to at least one aeroallergen. Few mothers reported smoking during pregnancy (4.8%), but at least one parent currently smoked in about half of the families. The median annual average of the daily maximum 8 hour averages, a measure of residential ambient ozone exposure, was 67 parts-per-billion (ppb). The residence of 48.2% of the children had a low ambient ozone exposure (≤67 ppb), and the residence of 51.8% of the children had a high exposure (>67 ppb).

### Genome-wide association scan

**Figure 1:**
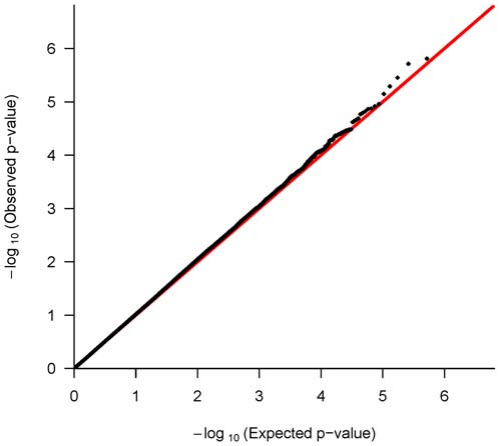
Quantile-quantile plot. Observed log-linear p-values (black dots) and the corresponding expected log-linear p-values (red line) are plotted on the logarithmic scale for the 520,767 autosomal GWAS SNPs tested for association with childhood asthma.

The 520,767 autosomal SNPs passing quality control were tested for association with childhood asthma using additive modeling with the log-linear method in 492 children with asthma and their biological parents from Mexico City. Not surprisingly given the study size, no SNP met genome-wide significance using a conservative Bonferroni adjustment. Nonetheless, the comparison of observed and expected p-values in the quantile-quantile plot (Figure 1) shows several top SNPs with some deviation from expectation. These deviations may occur by chance or may represent a true excess of small p-values.

**Figure 2:**
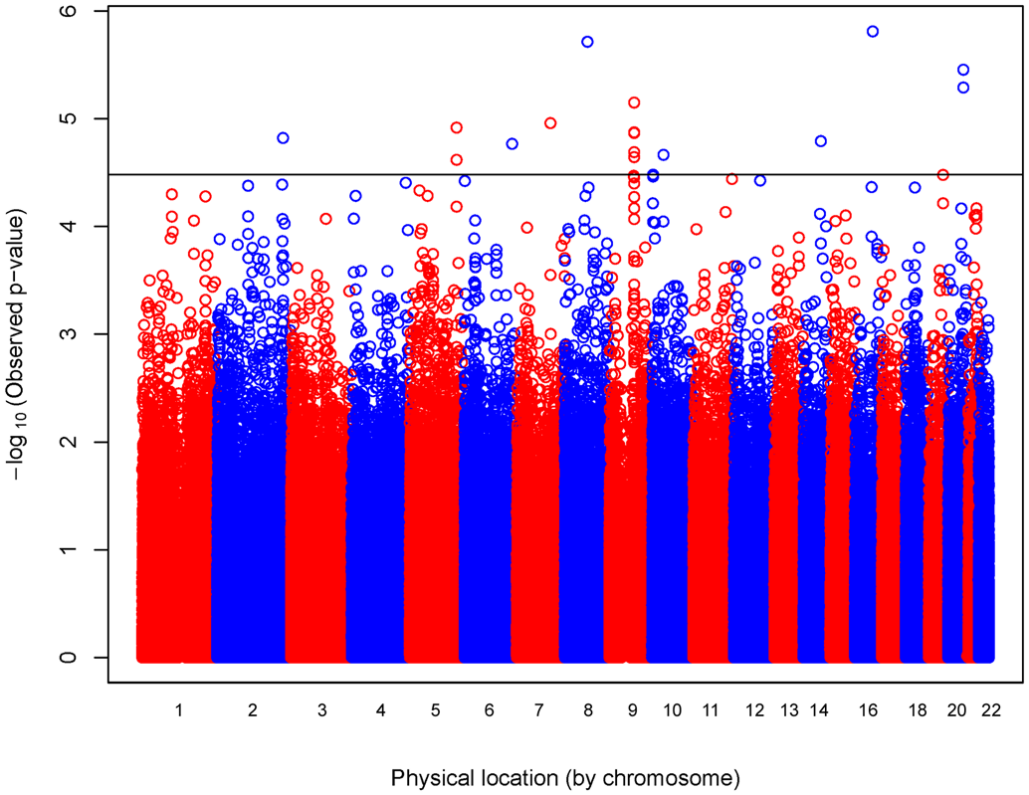
Physical location of GWAS p-values. Observed log-linear p-values on the logarithmic scale are sorted by physical location on the 22 autosomes for the 520,767 SNPs tested for association with childhood asthma. The 11 of the top 18 SNPs selected for replication are located at or above the solid line (p≤3.3×10^−5^).

Figure 2 shows the observed p-values plotted against chromosomal location. An intergenic SNP on chromosome 16 had the most significant association with childhood asthma [rs1867612 (p = 1.55×10^−6^)], followed by an intronic SNP in *potassium voltage-gated channel, Shab-related subfamily, member 2* (*KCNB2*; OMIM 607738) on chromosome 8 [rs2247572 (p = 1.94×10^−6^)] and two intergenic SNPs on chromosome 20 [rs6063725 (p = 3.52×10^−6^)] and rs720810 (p = 5.13×10^−6^)] with only moderate linkage disequilibrium (LD) (r^2^ = 0.59). The next most significant SNP (rs2378383) highlights a cluster of SNPs on chromosome 9q21.31 ranking among the top GWAS SNPs. This cluster of SNPs spans *transducin-like enhancer of split 4* (*E(sp1) homolog, Drosophila*) (*TLE4*; OMIM 605132) and its upstream region. LD analysis of SNPs with p≤0.001 in this cluster shows two large LD blocks in this region with one block encompassing *TLE4* and the other block encompassing the upstream region (Figure S1).

### Replication of top findings

Eleven of the 18 most significantly associated SNPs met our criteria to be selected for replication in 177 case-parent trios of Mexican ethnicity from the Genetics of Asthma in Latino Americans (GALA) study [14]. The GWAS p-values for the 11 SNPs selected for replication testing ranged from 3.30×10^−5^ to 1.55×10^−6^ (Figure 2). There were no significant deviations in Hardy-Weinberg equilibrium (HWE) for the replication SNPs in either the GWAS or replication study (p>0.12), and minor allele frequencies (MAFs) were similar between the two studies (Table 2). The replication study had at least 70% power to detect an association with four SNPs (rs2378377, rs4674039, rs1830206, and rs3814593) and at least 80% power to detect an association with the remaining seven SNPs (rs1867612, rs2247572, rs6063725, rs720810, rs2378383, rs6951506, and rs3734083) for similar MAFs and relative risk (RR) estimates observed in the GWAS.

**Table 2:** Replication testing for 11 top GWAS SNPs in an independent study of case–parent trios of Mexican ethnicity.

SNP^a^	Chr	SNP type	Gene^b^	Minor allele	GWAS (N = 492 trios)					Replication study (N = 177 trios)				
					MAF	RR^c^	Lower CI	Upper CI	Log-linear p-value	MAF	RR^c^	Lower CI	Upper CI	Log-linear p-value
rs1867612	16	Intergenic	(GOT2, CDH8)	A	0.08	0.44	0.31	0.63	1.55×10^−6^	0.11	1.11	0.68	1.84	0.67
rs2247572	8	Intronic	KCNB2	T	0.15	1.88	1.45	2.44	1.94×10^−6^	0.16	1.27	0.84	1.89	0.26
rs6063725	20	Intergenic	(SALL4, ZFP64)	C	0.34	1.56	1.30	1.89	3.52×10^−6^	0.38	1.15	0.86	1.56	0.35
rs720810	20	Intergenic	(SALL4, ZFP64)	G	0.26	1.61	1.32	2.00	5.13×10^−6^	0.32	1.16	0.85	1.61	0.34
rs2378383	9	Intergenic	(CHCHD9, TLE4)	G	0.22	0.61	0.49	0.76	7.10×10^−6^	0.20	0.63	0.41	0.96	0.030
rs6951506	7	Intronic	DOCK4	A	0.20	0.60	0.47	0.76	1.10×10^−5^	0.24	0.88	0.62	1.26	0.50
rs3734083	5	Intronic	GALNT10	T	0.18	1.68	1.33	2.13	1.21×10^−5^	0.19	0.72	0.48	1.07	0.10
rs2378377	9	Intergenic	(CHCHD9, TLE4)	G	0.28	0.64	0.53	0.79	1.36×10^−5^	0.28	0.71	0.50	1.02	0.061
rs4674039	2	Intergenic	(FN1, MREG)	C	0.42	0.67	0.56	0.81	1.51×10^−5^	0.49	1.11	0.82	1.51	0.50
rs1830206	10	Intergenic	(FZD8, ANKRD30A)	G	0.26	1.55	1.27	1.89	2.17×10^−5^	0.25	1.20	0.85	1.71	0.30
rs3814593	10	Intronic	PITRM1	T	0.32	0.67	0.55	0.81	3.30×10^−5^	0.35	0.82	0.58	1.15	0.24

a SNPs are ranked from smallest to largest GWAS p-value.

b Gene names are listed for SNPs mapping within genes. For intergenic SNPs, the nearest genes (upstream, downstream) are listed.

c Relative risk for carrying one copy of the minor allele compared to carrying no copies.

Association results in the GWAS and replication studies are compared in Table 2. No SNPs were significant with conservative Bonferroni correction for multiple testing, but two SNPs were associated with childhood asthma in the replication study with a p-value close to 0.05. The chromosome 9q21.31 SNP rs2378383, which is located 147 kb upstream of *TLE4* in an intergenic region between *coiled-coil-helix-coiled-coil-helix domain containing 9* (*CHCHD9*) and *TLE4*, was associated with childhood asthma in the replication study with p = 0.03. Meta-analysis of rs2378383 in the two studies gave a combined p-value of 6.79×10^−7^, and the RR estimate for carrying one copy of the rs2378383 minor allele (G) compared to carrying no copies in the GWAS [RR, 0.61; 95% confidence interval (CI), 0.49–0.76] was quite similar to the RR estimate in the replication study (RR, 0.63; 95% CI, 0.41–0.96). The SNP rs2378377, a neighboring intergenic SNP in moderate LD with rs2378383 (r^2^ = 0.73), had a marginal association with childhood asthma in the replication study with p = 0.06 (combined p = 2.68×10^−6^). RR estimates for the rs2378377 minor allele (G) were also similar between the GWAS (RR, 0.64; 95% CI, 0.53–0.79) and the replication study (RR, 0.71; 95%CI, 0.50–1.02). None of the other nine SNPs were associated in the replication study (Table 2).

Association results from additive modeling for SNPs in the region spanning *TLE4* and its upstream region (chromosome 9 nucleotide positions from 81,114,500 to 81,531,500, NCBI build 36.3) were obtained from the previous GWASs of asthma [5],[7],[8]. Our top two SNPs from this region were genotyped only in the GWAS in whites from the United States [7], where they were not associated with asthma (p = 0.59 for rs2378383 and p = 0.65 for rs2378377). Eighty-nine other SNPs were available in this region, and the smallest p-value was observed for rs1328406 (p = 0.056). There were 54 SNPs available in this region from the GWAS in German and British populations [5], with the smallest p-values observed for rs2807312 (p = 0.0041), rs7849719 (p = 0.018), rs7862187 (p = 0.033), rs10491790 (p = 0.043), and rs946808 (p = 0.049). From the 26 SNPs available from the GWAS in Puerto Ricans [8], the smallest p-value was 0.19. In our GWAS in Mexicans, there were several SNPs in *TLE4* and its upstream region with small p-values, and the SNPs listed above are located in close proximity to many of our associated SNPs. Similar to our GWAS and replication study, only cases with childhood-onset asthma were included in both GWASs in white populations [5],[7], and the cases from Himes et al. were predominantly atopic (91.2%) as defined by at least one positive skin prick test [7]. In contrast, the GWAS in Puerto Ricans included both childhood-onset and adulthood-onset asthma cases, and 83% of the cases were considered atopic as defined by total IgE>100 IU/mL [8].

### Associations in the presence and absence of environmental exposures

Associations of rs2378383 and rs2378377 were examined in data from the GWAS population stratified by residential ambient ozone exposure (199 trios with ≤67 ppb and 214 trios with>67 ppb annual average of the maximum 8 hour averages) and by current parental smoking (253 trios with and 233 trios without current parental smoking). The minor alleles of both SNPs were inversely associated with asthma at p<0.05 in all strata (results not shown), thus giving no evidence for effect modification in the presence of these environmental exposures.

### Associations in asthmatic children with atopy

Among the 445 cases with skin test data available, 408 can be classified as atopic by virtue of having at least one positive skin test. We repeated the GWA scan in this subset of 408 trios. Chromosome 9q21.31 SNPs predominated among the top ranked SNPs, with rs2378383 (p = 7.18×10^−7^) and rs2378377 (p = 1.08×10^−6^) being the two top ranking SNPs. In addition to smaller p-values, the magnitudes of association with asthma were slightly stronger for rs2378383 (RR, 0.54; 95% CI, 0.42–0.69) and rs2378377 (RR, 0.54; 95% CI, 0.42–0.72) in the subset of trios where the asthmatic child is also known to be atopic.

The chromosome 9q21.31 SNPs rs2378383 and rs2378377 were tested for association with the number of positive skin tests as a quantitative measure of the degree of atopy in the trios with skin test data. Both SNPs were associated with degree of atopy (p = 0.0018 for rs2378383 and p = 0.0010 for rs2378377). Their RR estimates indicate an inverse association in which carrying one copy of the minor allele is associated with a decreasing number of positive skin tests (RR, 0.92; 95% CI, 0.87–0.97 for rs2378383 and RR, 0.92; 95% CI, 0.88–0.97 for rs2378377), consistent with the direction of association for asthma.

### Ancestry analysis

**Figure 3:**
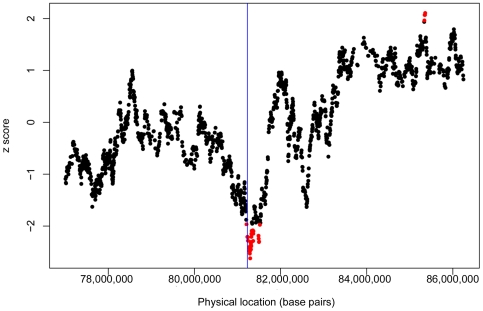
Chromosome 9q21.31 ancestry analysis. Segregation of Native American ancestry was tested along chromosome 9q21.31, the chromosomal region containing the replicated SNP (rs2378383). A 2,000 SNP window surrounding rs2378383 (located at the vertical blue line) is shown. Data points in red show a significant under- (or over-) transmission of Native American ancestry with |z-score| greater than 1.96, corresponding to an uncorrected p-value of 0.05.

The Mexican subjects in the GWAS had mean ancestral proportions of 69.5±15.6% for Native American, 27.3±14.3% for European, and 3.2±3.0% for African ancestries. Given the predominance of Native American ancestry, we evaluated Native American transmission in the GWAS along the chromosomal arm (9q) containing the replicated SNP (rs2378383) by ancestry analysis. As shown in Figure 3, there is a significant under-transmission of Native American ancestry at rs2378383 (z-score = −2.21 and two-sided p = 0.028) and surrounding SNPs.

The deficiency in Native American ancestry at this locus suggests that a protective allele occurred at a higher frequency in the Native American ancestral population than in the European and African ancestral populations. An examination of this SNP in the HapMap and Human Genome Diversity Panel (HGDP) data reveals that the frequency of the G allele is generally low in European, African, and East Asian populations (0.125 in HapMap European, 0.033 in HapMap African, 0.122 in HapMap Chinese, and 0.273 in HapMap Japanese), while its frequency is much higher in Native American populations (0.57 in HGDP Pima and 0.36 in HGDP Mayan). This pattern suggests that the G allele may be tagging a protective allele in the Native American ancestral population. This conclusion is consistent with the finding that the G allele is associated with a decreased risk for childhood asthma in the GWAS and replication analyses (Table 2).

### GWAS findings in the context of genome-wide expression data

**Figure 4:**
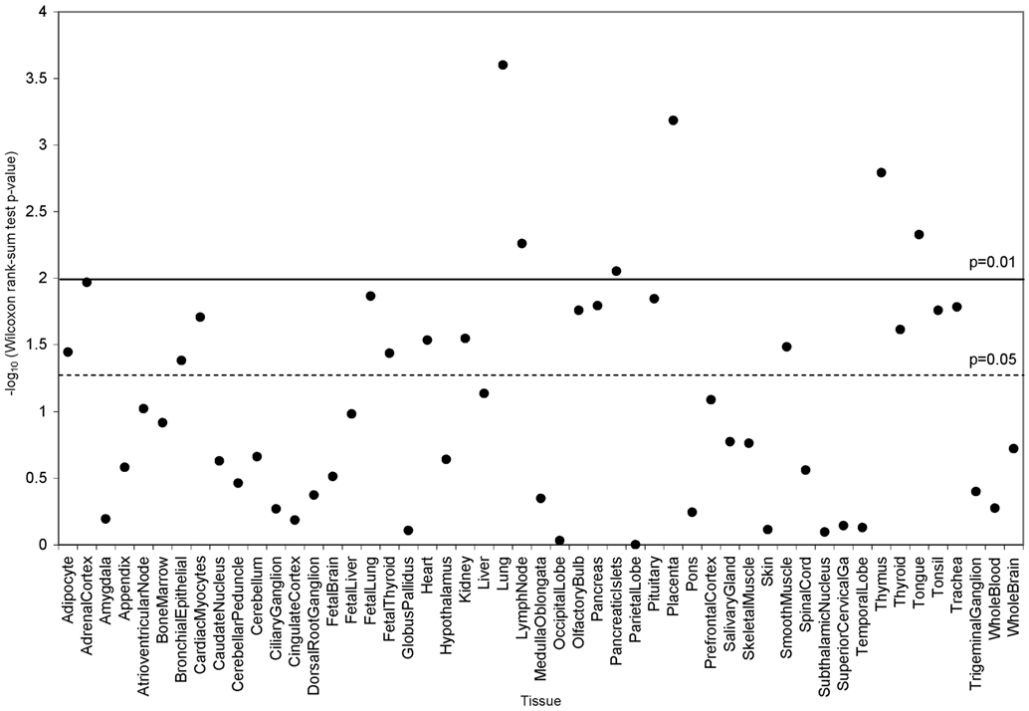
GWAS findings in the context of genome-wide expression. Genome-wide expression profiles were obtained from publicly available Novartis Research Foundation data, and the gene expression patterns were examined in the context of GWAS results for 14,330 genes. P-values for the Wilcoxon rank-sum test on the logarithmic scale indicate the significance in the difference of median GWAS q-values in genes expressed versus genes not expressed in each of 51 human tissues. Significance thresholds of p<0.01 (solid line) and p<0.05 (dashed line) are shown.

We examined gene expression patterns in 51 diverse human tissues in the context of GWAS findings to determine whether genes expressed in asthma relevant tissues ranked higher than genes not expressed in such tissues and thus to assess the biological plausibility of our overall GWAS findings. These results are presented in Figure 4. In the 14,330 genes with GWAS SNPs in the gene or nearby, median false discovery rate q-values (derived from the GWAS p-values) were compared between genes expressed versus genes not expressed in each of 51 human tissues.

Among the 51 tissues, the most significant deviation between the median q-values was found between 3,618 genes expressed in the lung versus 10,712 genes not expressed in the lung (Figure 4, p = 0.00025). This finding remains significant even after a conservative Bonferroni correction for multiple testing. The q-values for the 3,618 lung-expressed genes are presented in Table S1. *TLE4* did not contribute to the observation of significantly lower GWAS q-values in lung-expressed genes, as *TLE4* was classified as not expressed. *TLE4* displays a nearly ubiquitous expression pattern with similar low intensity levels across many tissues, so even though it is present in the lung, its expression level in the lung did not exceed our 75^th^ percentile threshold to be classified as expressed.

Other tissues in the respiratory and immune system also showed deviations in the median GWAS q-values for expressed versus not expressed genes, including thymus and lymph node at the p<0.01 level (uncorrected) and fetal lung, trachea, tonsil, smooth muscle, and bronchial epithelium at the p<0.05 level (uncorrected). Thus, SNPs in genes more highly expressed in tissues related to the pathogenesis of asthma and allergies tend to give more significant GWAS results than genes more highly expressed in other tissues. These results give biological credibility to our overall GWAS findings and are consistent with the multigenic etiology of asthma.

A two-dimensional cluster analysis was conducted to identify the implicated tissues with correlated gene expression patterns. As shown in Figure S2, lung tissue is grouped in a cluster with fetal lung and placenta tissues, thus suggesting that gene expression patterns in lung are most similar to gene expression patterns in fetal lung and placenta and that their signals are correlated. There are 2,385 genes classified as expressed in all three tissues – lung, fetal lung, and placenta. The gene expression patterns of other implicated tissues are also highly correlated, including the immune tissues tonsil, lymph node, and thymus (Figure S2).

## Discussion

Genetic studies of asthma are few in Hispanic populations, and to our knowledge, this work presents the first asthma GWAS in Mexicans and the most extensive coverage of genetic variation for an asthma GWAS in any Hispanic population. The GWAS included 492 Mexican case-parent trios. Given the moderate GWAS sample size, no SNP met genome-wide significance. However, the ranking of GWAS SNPs highlighted a potentially important candidate region for childhood asthma susceptibility, chromosome 9q21.31. Several chromosome 9q21.31 SNPs with small GWAS p-values were located in *TLE4* and its upstream region, and two of these SNPs (rs2378383 and rs2378377) were tested for replication in an independent study of 177 case-parent trios of Mexican ethnicity. Despite the small sample size for replication, both SNPs gave p-values close to 0.05 and the same direction and magnitude of association as the GWAS. Neither rs2378383 nor rs2378377 have a known impact on *TLE4* expression, but given their location upstream of *TLE4*, it is possible that these SNPs reside in a *TLE4* regulatory region.

Ancestry analysis in this chromosomal region provided supportive evidence that rs2378383 (G) is associated with a decreased risk of childhood asthma in Mexicans. Ancestry and transmission-based association analyses provide complementary but not completely independent lines of evidence. At each SNP, the log-linear method only used parents who were heterozygous in genotype, while ancestry analysis used all parents who are heterozygous in ancestry, including parents who are homozygous in genotype. We did not *a priori* expect that ancestry analysis results would corroborate log-linear association results.

Our ancestry analysis uses the same principles that underlie admixture mapping and relies on the key assumption of different risk allele frequencies between the ancestral populations, primarily Native American and European in this study. Under this assumption, individuals with disease in the admixed population would be expected to share an excess of ancestry from the population with the highest risk allele frequency at the disease locus [9]. In contrast, individuals with disease in the admixed population would be expected to share a shortage of ancestry from the population with the highest protective allele frequency at the disease locus. At chromosome 9q21.31, there was less Native American ancestry than expected, suggesting that the Native American ancestral population had a higher frequency of the protective rs2378383 allele (G).

Ancestry analysis implicated chromosome 9q21.31 as a chromosomal region that may underlie ethnic differences in childhood asthma. Complex diseases with differing disease prevalence rates in the ancestral populations are most suitable for ancestry analysis [15]. Prevalence rates of childhood asthma in the true ancestral Native American, European, and African populations are unknowable, but it is interesting to note that Mexicans have the highest Native American ancestry and the lowest asthma prevalence rate among Hispanic populations [16]. Differing frequencies of genetic risk factors in the ancestral populations presumably contribute to the differing prevalence rates of childhood asthma in modern populations. Our study found an association between Native American ancestry and a lower disease risk. Similarly, Native American ancestry was associated with milder asthma in a previous study of subjects of Mexican ethnicity from the GALA study [17]. These findings collectively suggest that the Native American ancestral population had higher frequencies of alleles that decrease prevalence and severity of asthma in the modern Mexican population. A comparison of asthma prevalence and severity among modern Native Americans, Europeans, and Africans would further support this interpretation, but such data are scarce [17].

The evidence for locus-specific ancestry around rs2378383 has implications for replication. Because rs2378383 (G) occurs at relatively low frequency in European, African and East Asian populations, genetic association studies in these populations are likely to suffer from lack of power at this locus. In contrast, the G allele occurs at moderate frequency in the Native American populations surveyed in HGDP [18]. Such disparate allele frequencies facilitate ancestry analysis in the region and improve the statistical power of transmission-based tests, as there are many more heterozygous parents in the Mexican population than a European, African or East Asian population. In fact, we obtained association results for SNPs in the chromosome 9q21.31 region from previous GWASs and found that SNPs in this region had only nominal evidence for association with asthma in the GWASs in white populations [5],[7]. It is not surprising that substantial evidence for replication was not found given the ethnicity differences (whites for Moffatt et al. and Himes et al. [5],[7] and Puerto Ricans for Choudhry et al. [8]). Future replication and fine-mapping of the region would be most effective if performed in Native American populations, or admixed populations with high Native American ancestral contributions.

The chromosome 9q21.31 SNPs associated with childhood asthma in the GWAS map to *TLE4* and its upstream region. The TLE family of proteins in humans is homologous to the Drosophila Groucho protein, which participates in cell fate determination for neurogenesis and segmentation. The highly conserved structure among the Drosophila Groucho and human TLE gene products suggest similar functions as transcriptional regulators in cell fate determination and differentiation [19]. Six genes encode the TLE family of proteins in humans (*TLE1*, *TLE2*, *TLE3*, *TLE4*, *TLE5*, *TLE6*), as deposited in the NCBI database. The distinct expression patterns among the TLE genes suggest a complex mechanism in humans involving non-redundant roles for the TLE genes [20]. The *TLE4* gene, in particular, shows ubiquitous expression across many tissues [19],[21], and TLE4 functions as a transcriptional co-repressor in several key developmental pathways [22]. More specifically, TLE4 has been implicated in early B-cell differentiation. TLE4 interacts with the transcription factor Paired box 5 (PAX5; OMIM 167414), which activates B-cell specific genes and represses alternative lineage fates [23]. A spliced version of TLE4 acts as a negative regulator for the PAX5/TLE4 function [23]. An alteration of B-cell differentiation involving TLE4 could be relevant to immune development and thus asthma.

TLE interacts with Runt-related transcription factor 3 (RUNX3; OMIM 600210) in a manner that may be directly relevant to asthma. In mice, loss of RUNX3 function results in an allergic asthma phenotype due to accelerated dendritic cell maturation and resulting increased efficacy to stimulate T cells [24]. Interaction with TLE is required for RUNX3 to inhibit dendritic cell maturation [25]. A recent paper provides support for the interaction of RUNX3 specifically with TLE4 [26]. Interestingly, the chromosome 9q21.31 SNPs rs2378383 and rs2378377 near *TLE4* are associated with asthma as well as degree of atopy in our data, and their associations with asthma became more pronounced when considering only the asthmatic children with atopy and their parents. These findings suggest that the influence of *TLE4* on asthma may be related to its influence on immune system development.

Childhood asthma is a complex disease, and there are likely many susceptibility genes influencing immune system development and asthma in the Mexican population. The examination of GWAS in the context of genome-wide expression illustrated the biological plausibility of our GWAS findings and showed consistency with the involvement of multiple genes. Genes expressed in the lung show association signals that differ most significantly from the association signals from genes not expressed in the lung when compared to 50 other human tissues. The lung represents a major pathogenic site for asthma, and this finding implies that multiple genes expressed in the lung are collectively associated with an increased risk of childhood asthma. Some of the other implicated tissues may represent false positives, but several of the highlighted tissues are biologically plausible for childhood asthma, including trachea, bronchial epithelium, smooth muscle, and immune tissues such as thymus, tonsil, and lymph node.

Other GWASs have implicated different susceptibility loci. Several SNPs implicated in the first asthma GWAS by Moffatt et al. in the *ORMDL3* region [5] were associated with childhood asthma in our GWAS [including rs9303277 (p = 0.036), rs11557467 (p = 0.014), rs8067378 (p = 0.020), rs2290400 (p = 0.037), and rs7216389 (p = 0.042)] but were not ranked among our top 5,000 SNPs. More recent GWASs have implicated loci other than *ORMDL3*. The *PDE4D* SNPs implicated by Himes et al. [7] were not associated with childhood asthma in our GWAS at p<0.05. Two nearby SNPs, not in LD with the implicated SNPs, were associated [rs13158277 (p = 0.030) and rs7717864 (p = 0.015)] but were also not ranked among our top 5,000 SNPs. Chromosome 5q23 SNPs implicated by Choudhry et al. [8] were not associated with childhood asthma in our GWAS at p<0.05. Initial GWAS findings regarded as replicated may not be ranked among the front runners in a genome-wide scan in the replication populations for statistical [27] as well as other biological reasons (such as ethnic differences, phenotypic heterogeneity, genetic heterogeneity, differing patterns of interacting environmental exposures, or multigenic etiology). This trend in discordant GWAS findings is quite common for various complex diseases [28], and follow-up studies are crucial in separating true genetic associations from false positives.

The major limitation of this study is the sample size for the GWAS and replication study. The Mexican population is largely under-studied given its size, and only moderate sample sizes are currently available for the study of asthma genetics. In our study, no SNPs met genome-wide significance, and no replication SNPs met the significance threshold when using a conservative Bonferroni correction for multiple testing. Despite this limitation, top GWAS findings, replication in an independent population, and ancestry analysis taken together implicate a novel region for association with asthma in Mexican children.

This study has several strengths. The case-parent trio design and the log-linear analysis protects against bias due to population stratification [29], so our GWAS results are not confounded by population stratification in this admixed population. Also, disease misclassification is minimal. Although bronchial hyper-reactivity was not tested, children with asthma were given reliable diagnoses based on clinical grounds by pediatric allergists at a pediatric allergy specialty clinic. The allergy clinic is a tertiary referral clinic, so the children with asthma were previously seen by a generalist and a pediatrician over time for recurrent asthma symptoms. Physician diagnosis of asthma has been shown to have a high level of validity in children after the first few years of life [30]. Further, the asthmatic children were predominantly atopic to aeroallergens based on skin prick testing limiting heterogeneity of the disease phenotype.

The GWAS and replication association results and the supporting ancestry analysis implicate chromosome 9q21.31 as a novel susceptibility locus for childhood asthma in the Mexican population. This region contains a biologically plausible novel susceptibility gene for childhood asthma, *TLE4*, but further work is needed to decipher whether *TLE4* or a nearby gene explain the signals from the chromosome 9q21.31 region. Further, childhood asthma is a complex disease with a proposed multigenic etiology, but most single studies will not have sufficient power to examine such complex relationships. Identification of important interacting risk factors in childhood asthma and other complex diseases will require very large sample sizes. This work identifies chromosome 9q21.31 (including *TLE4*) as a novel candidate susceptibility locus for childhood asthma, suggests that this region may underlie ethnic differences in childhood asthma, and emphasizes the presence of multiple genetic risk factors in the complex mechanism leading to childhood asthma.

## Materials and Methods

### Ethics statement

The study protocol was approved by the Institutional Review Boards of the Mexican National Institute of Public Health, Hospital Infantil de Mexico Frederico Gomez, and the US National Institute of Environmental Health Sciences (NIEHS). Parents gave written informed consent for the children's participation, and children gave their assent.

### Study subjects

Children with asthma (aged 5–17) and their biological parents were recruited between June 1998 and November 2003 from a pediatric allergy specialty clinic at a large public hospital in central Mexico City, Hospital Infantil de Mexico Frederico Gomez. The case-parent trio design protects against bias due to population stratification in this admixed population [29],[31]. Blood samples were collected from enrolled children and their parents for DNA extraction.

Children were diagnosed with asthma by a pediatric allergist at the referral clinic based on clinical symptoms and response to treatment [32]. Asthma severity was rated as mild (intermittent or persistent), moderate, or severe by the pediatric allergist according to symptoms in the Global Initiative on Asthma schema [33]. Questionnaires on the children's asthma symptoms and risk factors, including environmental tobacco smoking, were completed by parents, nearly always the mother.

The clinical evaluation also included skin prick testing to measure atopy and pulmonary function testing, as previously described [6]. A battery of 24 aeroallergens common in Mexico City was used for skin prick testing. Histamine was used as a positive control, and the test was considered valid if the histamine reaction was 6 mm or greater [34]. Glycerin was used as a negative control. Children were considered atopic if the diameter of skin reaction to at least one allergen exceeded 4 mm. Pulmonary function testing was performed at a later date using the EasyOne spirometer (ndd Medical Technologies, Andover, Massachusetts) according to American Thoracic Society guidelines [35]. Children were asked to withhold asthma medications on the morning of the test. The best test of three technically acceptable tests was selected. Percent predicted forced expiratory volume in 1 second (FEV_1_) was calculated using spirometric prediction equations from a childhood population in Mexico City [36].

Measurements of ambient ozone were obtained from the Mexican government's air monitoring station closest to each child's residence (within 5 km). The annual average of the daily maximum 8 hour averages of the ozone level was collected for the year prior to study entry and dichotomized at the median for stratified analyses. Further details on the ozone measurement protocol have been described elsewhere [6].

### Genome-wide association study genotyping and quality control

Peripheral blood lymphocytes were isolated from whole blood, and DNA was extracted using Gentra Puregene kits (Gentra Systems, Minneapolis, Minnesota). A total of 498 complete case-parent trios with previously confirmed parentage and sufficient amounts of DNA were genotyped for 561,466 SNPs using the Illumina HumanHap 550 K BeadChip, version 3 (Illumina, San Diego, California) at the University of Washington, Department of Genome Sciences. Genotypes were determined using the Illumina BeadStudio Genotyping Module, following the recommended conditions. Results for three unrelated study subjects fell below the genotyping call rate threshold of 95% resulting in exclusion of three trios. The remaining study subjects were genotyped with an average call rate of 99.7%.

Quality control analyses were conducted using PLINK (http://pngu.mgh.harvard.edu/~purcell/plink/) [37], unless otherwise stated. In preliminary SNP-level quality control, SNPs were excluded due to poor chromosomal mapping (N = 173), missingness>10% (N = 988), MAF<0.001% (N = 253), HWE p-value (in parents only)<1×10^−10^ (N = 557), Mendelian errors in more than two families (N = 4,945), and heterozygous genotype calls for chromosome X SNPs in more than one male (N = 380). All SNP exclusions were made sequentially in the above order.

Subject-level quality control verified that no subjects had unusual autosomal homozygosity or an inconsistent sex between genotype and collected phenotype data. Subject-level quality control next assessed subject relatedness to identify unknown intra- and inter-family relationships. This identified two duplicated trios and one trio with first-degree relative parents requiring exclusion. Trio exclusions were not necessary for other identified relationships, including parents in different trios being first-degree or second-degree relatives and nuclear families with two children with asthma being split into two case-parent trios. There were 492 complete case-parent trios (1,468 study subjects) in the final analysis data set.

Final SNP-level quality control made exclusions due to one or more discordant genotypes across 14 HapMap replicate samples identified using the Genotyping Library and Utilities application (http://code.google.com/p/glu-genetics/) (N = 921) [38]. Final SNP exclusions were also made due to more stringent missingness and MAF thresholds, missingness>5% (N = 3,137) and MAF<1% (N = 16,696). Of the 533,416 SNPs passing all quality control criteria (95.0%), the 520,767 autosomal SNPs were analyzed for purposes of this study.

### Replication study subjects

Subjects of Mexican ethnicity from the GALA study were used for replication testing. The GALA study protocol has been described elsewhere [14]. Subjects of Mexican ethnicity with physician-diagnosed asthma and presence of two or more asthma symptoms in the past two years (wheezing, coughing, and shortness of breath) were enrolled along with both biological parents in San Francisco, California, US, and Mexico City, Mexico. Total plasma IgE was measured for all subjects with asthma.

The children with asthma in the GWAS (less than 18 years old) were enrolled at a pediatric allergy clinic, so nearly all had allergic asthma. To maximize comparability with the GWAS, the replication study included only the 177 complete case-parent trios comprising subjects of Mexican ethnicity having childhood-onset (age of onset<18 years) asthma and atopy (total IgE>100 IU/mL) and their parents.

### Replication SNP selection and genotyping

SNPs were ranked by GWAS p-value. The top SNP and 10 other top ranking GWAS SNPs were tested for replication in the GALA study. The highest ranking SNP along with 10 other top ranking SNPs with no strong LD (r^2^<0.9) with other higher-ranked SNPs and MAF>10% were selected for replication. Statistical power to detect associations in the replication study of 177 case-parent trios was calculated for each selected SNP using QUANTO (http://hydra.usc.edu/gxe) [39]. The MAFs and RR estimates observed from the GWAS with a log-additive model were specified in the power calculation for each selected SNP.

Genotyping for replication SNPs was performed on the Applied Biosystems (ABI, Foster City, California) PRISM 7500 Real-Time PCR System using primers and probes from ABI's Assay-by-Demand. The assay was performed under universal conditions, with each reaction containing 3.75 ng DNA, 0.125 µL 40X Assay Mix and 2.5 µL TaqMan Universal PCR master mix brought to a final volume of 5 µL with sterile water. Thermal cycling conditions began at 95°C for 10 minutes and then proceeded with 60 cycles of 92°C for 15 seconds and 60°C for 2 minutes. After the PCR reaction, plates were scanned by the ABI PRISM 7500 PCR system to determine genotypes by allelic discrimination.

### Statistical analysis of genotype data

The log-linear likelihood approach was used to examine associations of individual SNPs with childhood asthma in the GWAS and replication study [29],[40]. The log-linear method is a generalization of the classic family-based test for association between genetic variants and disease, the transmission disequilibrium test [41], which compares the distribution of alleles transmitted from parents to affected offspring with the distribution of alleles not transmitted. An asymmetry of allele distributions implies that the variant under study is associated with disease within families. This inference requires the assumption of Mendelian inheritance, such that the allele under study is not related to the parents' fertility or to the offspring's survival [40]. The log-linear method and other transmission-based methods test the same null hypothesis of no linkage or no association (i.e. no LD) between the allele and disease [40]. Unlike other transmission-based methods, the log-linear method provides risk estimates to assess the direction and magnitude of association.

Robustness to population stratification is a well-known property of the case-parent trio design and transmission-based methods. The log-linear method achieves robustness to population stratification by stratifying on the six possible parental mating types, which are defined by the number of copies of the allele carried by each of the two parents [40]. The assumption of HWE is not required for the log-linear method, but we tested for HWE in parents as a check for genotyping error.

The log-linear method was implemented using the LEM computer program [42] with a one degree-of-freedom log-additive risk model specified. When missing genotypes occur, the log-linear method uses the expectation-maximization algorithm to maximize the likelihood, allowing incomplete trios to contribute information and minimizing loss of statistical power [31]. P-values were generated to assess statistical significance, and the RR of carrying one copy of the risk allele was calculated to assess the direction and magnitude of association. For the most significantly associated GWAS SNPs, pair-wise LD was assessed in the parents using PLINK (http://pngu.mgh.harvard.edu/~purcell/plink/) [37] or HAPLOVIEW (http://www.broad.mit.edu/mpg/haploview/) [43]. A combined p-value from meta-analysis of the GWAS and replication association results was computed using MANTEL [44].

Interesting SNPs from the GWAS and replication study were tested for effect modification with environmental exposures relating to air pollution and environmental tobacco smoking. Data from the GWAS population were stratified by current parental smoking (yes/no) and residential ambient ozone exposure (stratified at the median of 67 ppb annual average of the daily maximum 8 hour averages), and the log-linear method was used to test for genetic associations in each stratum.

Additional analyses were conducted in the GWAS population using the skin prick testing data. The genome-wide association scan was repeated in the subset of trios with children classified as atopic (one or more positive skin tests) in an effort to reduce phenotypic heterogeneity and thus reduce genetic heterogeneity. Then, an extension of the log-linear method for quantitative traits [45] was used to test for associations of interesting SNPs with the number of positive skin tests to a battery of 24 aeroallergens as a measure of atopy. This analysis was performed only on the case-parent trios with skin test data and complete genotype data.

### Ancestry analysis

The program FRAPPE was used to estimate individual ancestry proportions, assuming three ancestral populations: Native American, European, and African [46]. HapMap (phase 3) genotype data from 109 individuals from the United States with northern and western European descent and 108 individuals from Nigeria were included to represent ancestral European and African individuals, respectively. Genotype data from 35 Mayan and Pima Indians were taken from the HGDP, which have been genotyped using Illumina 650 K arrays [18], as the best available representation of ancestral Native American individuals. Individual ancestry proportions were averaged across all study subjects to determine the mean Native American, European, and African ancestral contributions.

The program SABER was used to infer locus-specific ancestry in each individual [47]. Our goal was to elucidate the pattern of ancestry segregation near the replicated chromosome 9q SNP (rs2378383). SNPs along chromosome 9q were analyzed to more accurately infer ancestry, but since we were only interested in examining the pattern of ancestry segregation at rs2378383 *a priori*, a multiple testing correction was not applied. A trio-based ancestry analysis test was implemented similar to that described in Clarke and Whittemore [48], which parallels the transmission disequilibrium test [41]. Because the African ancestry in this population is quite low (<5%), we tested Native American versus non-Native American ancestry. At each SNP, parents were considered to have no Native American ancestral alleles if their posterior Native American ancestry was less than 0.1, one ancestral allele if between 0.4 and 0.6, and two ancestral alleles if greater than 0.9. Conservative thresholds were set to minimize misclassification. Parents with intermediate ancestry estimates falling outside the specified ranges were excluded, resulting in a 10% missing rate. For parents who were heterozygous in ancestry, the null hypothesis that the Native American allele is transmitted to the children with probability equal to ½ was tested at each SNP [48].

### Genome-wide expression data

The Genomics Institute of the Novartis Research Foundation maintains a freely accessible database (http://symatlas.gnf.org) of genome-wide expression profiles of the protein-encoding transcriptome in many diverse human and mouse tissues and cell lines [21]. As reported, tissue samples were predominantly obtained from the normal physiological state [21]. The custom expression array for humans targeted 44,775 transcripts corresponding to known, predicted, and poorly characterized protein-encoding genes [21]. We obtained the expression data for the 44,775 transcripts in 51 diverse human tissues and mapped these transcripts to 15,047 unique genes after accounting for multiple transcripts per gene and mapping to current nomenclature.

Gene expression patterns in the Novartis data were examined in the context of GWAS results. Of the 15,047 genes with expression data, 12,199 genes had at least one genotyped GWAS SNP mapping within the gene, and an additional 2,131 genes had at least one genotyped SNP mapping near the gene for a total of 14,330 genes. SNPs mapping within the 5′- most extent to the 3′- most extent over all isoforms for a gene or within a larger region expanded by 50 kb in both directions were considered. This broader region was considered in order to capture potential regulatory regions. For each gene, one false discovery rate q-value was calculated using the log-linear p-values of SNPs mapping within or near the gene based on a method for combining p-values by Peng et al. [49]. Genes were then categorized as expressed or not expressed in each of the 51 tissues examined. The expression threshold was the 75^th^ percentile of normalized intensity values for each tissue. The global median q-values across genes expressed versus genes not expressed were calculated for each tissue, and a two-tailed Wilcoxon rank-sum test was conducted to generate a test of significance for this difference in median q-values.

Gene expression patterns are correlated across different tissues. We performed a two-dimensional hierarchical clustering to describe the correlation of expression patterns using Spearman's correlation coefficient across genes and across tissues. Genes and tissues with similar gene expression patterns were grouped into clusters using Ward's distance as the linkage function to be optimized. Partek Genomics Suite 6.08.1010 (Partek Inc., St. Louis, Missouri) software was used to perform this analysis using the continuous expression values in the 15,047 genes with expression data.

## Supporting Information

Figure S1 LD structure of SNPs in or near *TLE4*.
(0.08 MB DOC)

Figure S2 Two-dimensional cluster analysis of genome-wide expression data.
(3.72 MB DOC)

Table S1 Q-values for the 3,618 genes classified as expressed in lung tissue.
(2.99 MB DOC)
