# Correction: Genome-Wide Association Study Implicates Chromosome 9q21.31 as a Susceptibility Locus for Asthma in Mexican Children

**DOI:** 10.1371/annotation/dde89c4c-03f7-4747-8426-180c4ecee5d5

**Published:** 2009-12-02

**Authors:** Dana B. Hancock, Isabelle Romieu, Min Shi, Juan-Jose Sienra-Monge, Hao Wu, Grace Y. Chiu, Huiling Li, Blanca Estela del Rio-Navarro, Saffron A. G. Willis-Owen, Scott T. Weiss, Benjamin A. Raby, Hong Gao, Celeste Eng, Rocio Chapela, Esteban G. Burchard, Hua Tang, Patrick F. Sullivan, Stephanie J. London

The ninth author's name was spelled incorrectly. The correct name is: Saffron A. G. Willis-Owen.

