# Supplementary material for: Genome-Wide Association Study Implicates Chromosome 9q21.31 as a Susceptibility Locus for Asthma in Mexican Children
Source: PLoS Genet. 2009 Aug 28;5(8):e1000623. doi: 10.1371/journal.pgen.1000623 (PMC2722731; doi:10.1371/journal.pgen.1000623)
Supplement: Figure S1 — LD structure of SNPs in or near TLE4. (0.08 MB DOC) [file pgen.1000623.s001.doc]

**
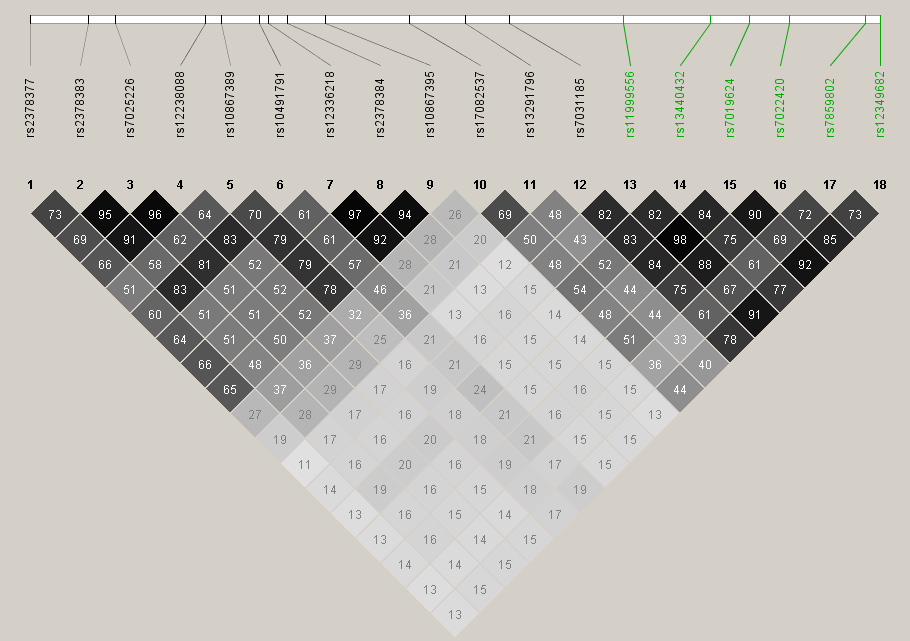
**

Figure S1. LD structure of SNPs in or near *TLE4*. LD structure among the cluster of chromosome 9q21.31 SNPs associated with childhood asthma at p<0.001 in the GWAS scan. Pairwise r^2^ values in the parents are shown, with darker shading indicating stronger LD. SNPs highlighted in green are located in *TLE4*, while other SNPs are located upstream of *TLE4*.
