# Supplementary material for: Genome-Wide Association Study Implicates Chromosome 9q21.31 as a Susceptibility Locus for Asthma in Mexican Children
Source: PLoS Genet. 2009 Aug 28;5(8):e1000623. doi: 10.1371/journal.pgen.1000623 (PMC2722731; doi:10.1371/journal.pgen.1000623)
Supplement: Figure S2 — Two-dimensional cluster analysis of genome-wide expression data. (3.72 MB DOC) [file pgen.1000623.s002.doc]

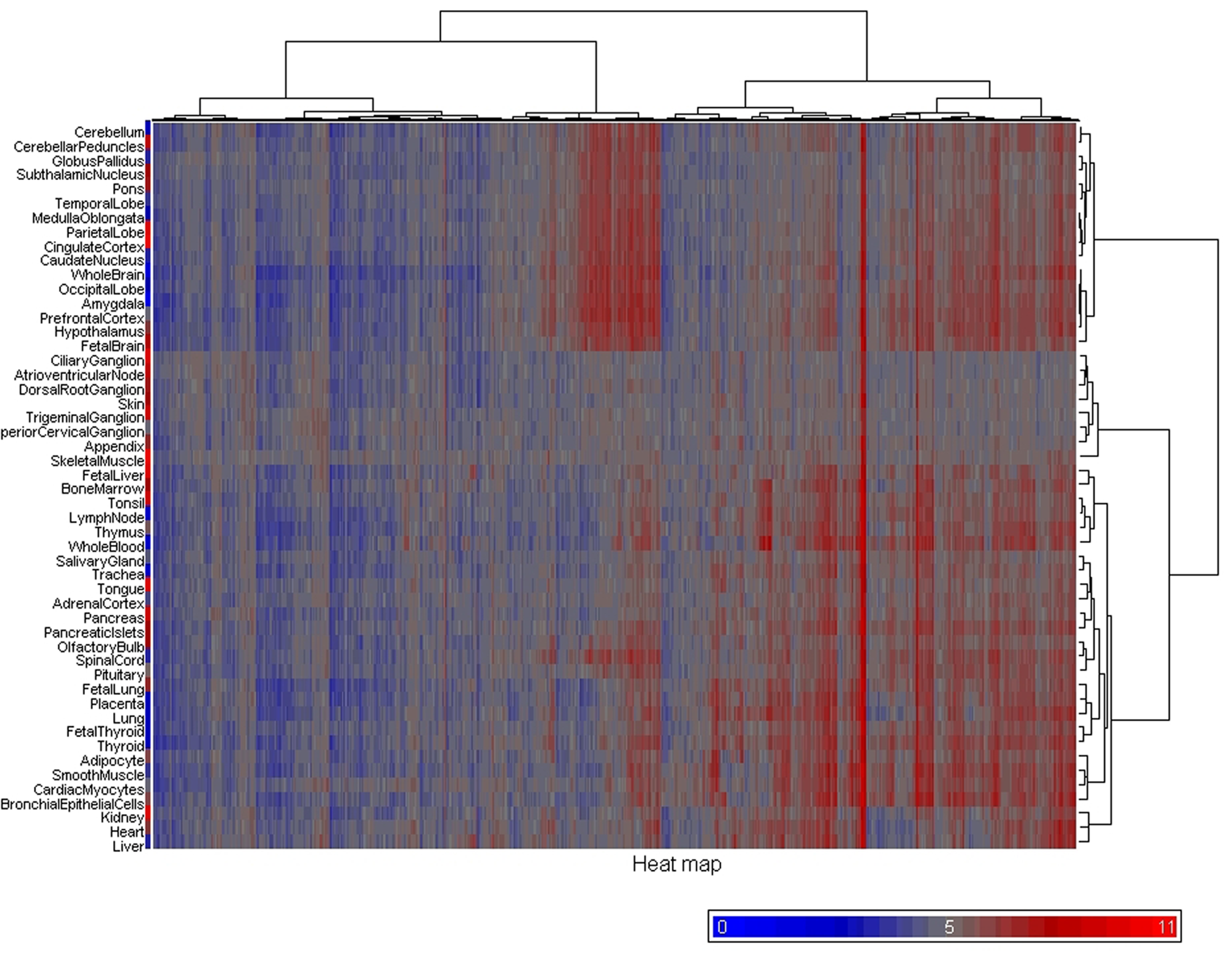


Figure S2. Two-dimensional cluster analysis of genome-wide expression data. The heat map illustrates the gene expression patterns clustered across 51 human tissues and across the 15,047 genes available in the Novartis expression database. The color scale ranges from blue (indicating no expression) to red (indicating the highest level of expression intensity).
