# Supplementary material for: Genome-Wide Association Study Implicates Chromosome 9q21.31 as a Susceptibility Locus for Asthma in Mexican Children
Source: PLoS Genet. 2009 Aug 28;5(8):e1000623. doi: 10.1371/journal.pgen.1000623 (PMC2722731; doi:10.1371/journal.pgen.1000623)
Supplement: Table S1 — Q-values for the 3,618 genes classified as expressed in lung tissue. (2.99 MB DOC) [file pgen.1000623.s003.doc]

Table S1. Q-values for the 3,618 genes classified as expressed in lung tissue. The false discovery rate q-value for each gene was derived from combining the log-linear p-values of SNPs in or near the gene, while accounting for the differing number of SNPs per gene. The minimum p-value among SNPs located in or near the gene is also shown.

| **Gene** | **Number of SNPs** | **Minimum p-value** | **q-value** |
| --- | --- | --- | --- |
| *A1CF* | 25 | 6.51 x 10^-3^ | 1.63 x 10^-1^ |
| *A2M* | 30 | 2.03 x 10^-1^ | 9.96 x 10^-1^ |
| *AAMP* | 11 | 6.15 x 10^-2^ | 5.02 x 10^-1^ |
| *AARS* | 3 | 9.38 x 10^-2^ | 2.81 x 10^-1^ |
| *AATF* | 20 | 6.48 x 10^-3^ | 1.30 x 10^-1^ |
| *AATK* | 23 | 4.38 x 10^-2^ | 6.00 x 10^-1^ |
| *ABCA3* | 14 | 9.31 x 10^-3^ | 1.29 x 10^-1^ |
| *ABCA7* | 28 | 4.64 x 10^-2^ | 5.29 x 10^-1^ |
| *ABCB5* | 64 | 3.20 x 10^-3^ | 2.05 x 10^-1^ |
| *ABCC1* | 92 | 1.47 x 10^-2^ | 4.97 x 10^-1^ |
| *ABCC10* | 13 | 1.04 x 10^-1^ | 2.60 x 10^-1^ |
| *ABCC5* | 36 | 6.74 x 10^-2^ | 9.48 x 10^-1^ |
| *ABCF1* | 12 | 5.03 x 10^-3^ | 5.56 x 10^-2^ |
| *ABCF3* | 22 | 3.54 x 10^-2^ | 2.18 x 10^-1^ |
| *ABCG1* | 68 | 6.91 x 10^-4^ | 4.49 x 10^-2^ |
| *ABHD10* | 52 | 7.55 x 10^-3^ | 2.49 x 10^-1^ |
| *ABHD11* | 11 | 6.60 x 10^-2^ | 6.16 x 10^-1^ |
| *ABHD12* | 14 | 7.43 x 10^-2^ | 9.95 x 10^-1^ |
| *ABHD12B* | 43 | 2.45 x 10^-2^ | 6.00 x 10^-1^ |
| *ABHD14A* | 4 | 1.41 x 10^-1^ | 4.02 x 10^-1^ |
| *ABHD14B* | 3 | 1.41 x 10^-1^ | 2.07 x 10^-1^ |
| *ABHD2* | 48 | 6.38 x 10^-2^ | 6.91 x 10^-1^ |
| *ABHD4* | 57 | 1.44 x 10^-2^ | 8.20 x 10^-1^ |
| *ABI3* | 19 | 1.39 x 10^-2^ | 4.42 x 10^-2^ |
| *ABI3BP* | 93 | 1.65 x 10^-2^ | 5.26 x 10^-1^ |
| *ABL1* | 51 | 7.59 x 10^-2^ | 8.83 x 10^-1^ |
| *ABLIM1* | 87 | 2.03 x 10^-2^ | 8.64 x 10^-1^ |
| *ABR* | 69 | 9.13 x 10^-3^ | 1.39 x 10^-1^ |
| *ABT1* | 11 | 1.46 x 10^-1^ | 2.61 x 10^-1^ |
| *ACAA1* | 10 | 6.49 x 10^-2^ | 2.25 x 10^-1^ |
| *ACAD9* | 19 | 1.52 x 10^-3^ | 1.76 x 10^-2^ |
| *ACADVL* | 14 | 1.41 x 10^-1^ | 7.24 x 10^-1^ |
| *ACAT1* | 15 | 7.06 x 10^-2^ | 8.76 x 10^-1^ |
| *ACBD3* | 22 | 1.45 x 10^-2^ | 1.00 x 10^-1^ |
| *ACBD6* | 52 | 3.33 x 10^-2^ | 6.46 x 10^-1^ |
| *ACO1* | 46 | 1.07 x 10^-2^ | 4.69 x 10^-1^ |
| *ACO2* | 14 | 7.88 x 10^-2^ | 8.39 x 10^-1^ |
| *ACOT2* | 9 | 2.53 x 10^-1^ | 8.87 x 10^-1^ |
| *ACOT8* | 21 | 1.55 x 10^-1^ | 4.81 x 10^-1^ |
| *ACP2* | 14 | 1.01 x 10^-1^ | 5.45 x 10^-1^ |
| *ACP5* | 16 | 7.07 x 10^-2^ | 5.52 x 10^-1^ |
| *ACP6* | 29 | 1.98 x 10^-1^ | 9.20 x 10^-1^ |
| *ACSL1* | 44 | 2.94 x 10^-2^ | 4.70 x 10^-1^ |
| *ACSL5* | 25 | 4.56 x 10^-2^ | 6.97 x 10^-1^ |
| *ACTA2* | 39 | 1.16 x 10^-1^ | 8.68 x 10^-1^ |
| *ACTB* | 21 | 1.50 x 10^-1^ | 1.00 |
| *ACTG1* | 12 | 1.54 x 10^-2^ | 1.29 x 10^-1^ |
| *ACTG2* | 34 | 1.29 x 10^-1^ | 9.34 x 10^-1^ |
| *ACTN1* | 81 | 1.29 x 10^-2^ | 9.88 x 10^-1^ |
| *ACTN4* | 23 | 2.32 x 10^-1^ | 8.21 x 10^-1^ |
| *ACTR10* | 7 | 1.26 x 10^-1^ | 6.04 x 10^-1^ |
| *ACTR1A* | 14 | 1.30 x 10^-1^ | 6.67 x 10^-1^ |
| *ACTR1B* | 8 | 3.39 x 10^-1^ | 8.45 x 10^-1^ |
| *ACTR3* | 28 | 4.22 x 10^-2^ | 9.95 x 10^-1^ |
| *ACVRL1* | 27 | 9.43 x 10^-2^ | 8.40 x 10^-1^ |
| *ADAMTS1* | 48 | 4.47 x 10^-2^ | 9.92 x 10^-1^ |
| *ADAMTS12* | 141 | 3.37 x 10^-3^ | 4.65 x 10^-1^ |
| *ADAMTS8* | 30 | 5.94 x 10^-2^ | 6.65 x 10^-1^ |
| *ADAR* | 25 | 3.55 x 10^-2^ | 5.79 x 10^-1^ |
| *ADARB1* | 56 | 3.74 x 10^-2^ | 6.78 x 10^-1^ |
| *ADC* | 11 | 2.69 x 10^-1^ | 1.00 |
| *ADCY6* | 16 | 5.28 x 10^-3^ | 7.01 x 10^-2^ |
| *ADD1* | 24 | 5.75 x 10^-2^ | 7.16 x 10^-1^ |
| *ADFP* | 27 | 1.08 x 10^-2^ | 2.49 x 10^-1^ |
| *ADH1B* | 22 | 4.13 x 10^-2^ | 4.96 x 10^-1^ |
| *ADH5* | 20 | 4.51 x 10^-3^ | 8.43 x 10^-2^ |
| *ADI1* | 18 | 8.24 x 10^-2^ | 7.39 x 10^-1^ |
| *ADIPOR1* | 26 | 4.97 x 10^-2^ | 1.86 x 10^-1^ |
| *ADIPOR2* | 52 | 9.14 x 10^-3^ | 3.58 x 10^-1^ |
| *ADM* | 14 | 3.16 x 10^-1^ | 8.42 x 10^-1^ |
| *ADORA2A* | 12 | 9.94 x 10^-2^ | 8.50 x 10^-1^ |
| *ADORA2B* | 15 | 2.14 x 10^-2^ | 1.83 x 10^-1^ |
| *ADPGK* | 19 | 4.06 x 10^-2^ | 7.23 x 10^-1^ |
| *ADPRHL2* | 13 | 2.59 x 10^-3^ | 1.84 x 10^-2^ |
| *ADRB2* | 34 | 6.55 x 10^-2^ | 9.79 x 10^-1^ |
| *ADRBK1* | 11 | 1.86 x 10^-2^ | 1.31 x 10^-1^ |
| *ADRM1* | 14 | 1.98 x 10^-2^ | 1.33 x 10^-1^ |
| *ADSL* | 11 | 5.35 x 10^-2^ | 5.89 x 10^-1^ |
| *AEBP1* | 21 | 2.81 x 10^-2^ | 2.81 x 10^-1^ |
| *AES* | 23 | 6.30 x 10^-2^ | 7.17 x 10^-1^ |
| *AFG3L2* | 17 | 1.42 x 10^-3^ | 2.29 x 10^-2^ |
| *AGER* | 50 | 1.18 x 10^-2^ | 5.70 x 10^-1^ |
| *AGPAT1* | 47 | 1.18 x 10^-2^ | 5.41 x 10^-1^ |
| *AGPAT2* | 21 | 2.68 x 10^-2^ | 2.83 x 10^-1^ |
| *AGRN* | 5 | 3.94 x 10^-1^ | 7.58 x 10^-1^ |
| *AGXT2L2* | 44 | 1.52 x 10^-2^ | 3.48 x 10^-1^ |
| *AHCY* | 10 | 1.61 x 10^-1^ | 8.32 x 10^-1^ |
| *AHCYL1* | 26 | 1.15 x 10^-1^ | 9.18 x 10^-1^ |
| *AHNAK* | 45 | 3.30 x 10^-2^ | 4.63 x 10^-1^ |
| *AHSA1* | 25 | 9.56 x 10^-2^ | 6.45 x 10^-1^ |
| *AHSG* | 22 | 1.33 x 10^-2^ | 2.51 x 10^-1^ |
| *AIM1* | 53 | 2.60 x 10^-1^ | 9.81 x 10^-1^ |
| *AIP* | 10 | 6.23 x 10^-2^ | 2.11 x 10^-1^ |
| *AK1* | 18 | 3.49 x 10^-2^ | 5.54 x 10^-1^ |
| *AKAP1* | 31 | 1.78 x 10^-2^ | 5.51 x 10^-1^ |
| *AKAP12* | 73 | 2.91 x 10^-2^ | 9.70 x 10^-1^ |
| *AKAP13* | 134 | 2.09 x 10^-2^ | 9.62 x 10^-1^ |
| *AKIRIN2* | 19 | 9.79 x 10^-3^ | 4.98 x 10^-2^ |
| *AKR1A1* | 12 | 3.46 x 10^-1^ | 8.42 x 10^-1^ |
| *AKR1B1* | 24 | 9.39 x 10^-3^ | 1.37 x 10^-1^ |
| *AKR1C1* | 22 | 5.10 x 10^-2^ | 7.23 x 10^-1^ |
| *AKR1C2* | 22 | 5.10 x 10^-2^ | 6.51 x 10^-1^ |
| *AKR1C3* | 22 | 2.20 x 10^-1^ | 8.62 x 10^-1^ |
| *AKR7A2* | 24 | 3.72 x 10^-3^ | 4.52 x 10^-2^ |
| *AKR7A3* | 18 | 2.72 x 10^-2^ | 2.23 x 10^-1^ |
| *AKT1* | 8 | 6.64 x 10^-2^ | 2.66 x 10^-1^ |
| *AKT1S1* | 21 | 3.32 x 10^-2^ | 2.59 x 10^-1^ |
| *ALAS1* | 7 | 4.01 x 10^-1^ | 9.27 x 10^-1^ |
| *ALDH1L2* | 29 | 1.53 x 10^-2^ | 1.24 x 10^-1^ |
| *ALDH2* | 9 | 3.81 x 10^-1^ | 9.63 x 10^-1^ |
| *ALDH3A1* | 15 | 7.31 x 10^-2^ | 7.46 x 10^-1^ |
| *ALDH3A2* | 14 | 3.41 x 10^-1^ | 7.99 x 10^-1^ |
| *ALDH3B1* | 13 | 1.27 x 10^-2^ | 7.55 x 10^-2^ |
| *ALDH6A1* | 10 | 7.68 x 10^-2^ | 7.68 x 10^-1^ |
| *ALDH9A1* | 41 | 1.08 x 10^-2^ | 2.89 x 10^-1^ |
| *ALDOA* | 11 | 1.84 x 10^-1^ | 9.81 x 10^-1^ |
| *ALDOC* | 6 | 4.50 x 10^-2^ | 6.24 x 10^-2^ |
| *ALG3* | 15 | 2.49 x 10^-2^ | 2.71 x 10^-1^ |
| *ALG5* | 15 | 1.21 x 10^-1^ | 7.84 x 10^-1^ |
| *ALKBH5* | 22 | 3.06 x 10^-1^ | 9.51 x 10^-1^ |
| *ALKBH7* | 14 | 4.58 x 10^-2^ | 6.41 x 10^-1^ |
| *ALOX15B* | 37 | 2.79 x 10^-2^ | 3.90 x 10^-1^ |
| *ALOX5* | 27 | 3.50 x 10^-2^ | 5.19 x 10^-1^ |
| *ALOX5AP* | 48 | 6.64 x 10^-2^ | 8.93 x 10^-1^ |
| *ALPL* | 69 | 3.82 x 10^-3^ | 2.40 x 10^-1^ |
| *ALS2CL* | 31 | 4.99 x 10^-3^ | 1.07 x 10^-1^ |
| *ALS2CR2* | 10 | 3.98 x 10^-1^ | 9.63 x 10^-1^ |
| *AMAC1L2* | 21 | 7.63 x 10^-3^ | 1.23 x 10^-1^ |
| *AMOTL2* | 27 | 9.87 x 10^-2^ | 9.31 x 10^-1^ |
| *AMPD2* | 20 | 4.00 x 10^-2^ | 4.96 x 10^-1^ |
| *AMT* | 1 | 8.98 x 10^-1^ | 8.98 x 10^-1^ |
| *ANAPC5* | 13 | 3.13 x 10^-1^ | 9.40 x 10^-1^ |
| *ANGPTL2* | 14 | 6.55 x 10^-2^ | 5.20 x 10^-1^ |
| *ANKRD22* | 32 | 1.97 x 10^-2^ | 4.01 x 10^-1^ |
| *ANKS1A* | 44 | 2.39 x 10^-4^ | 1.49 x 10^-3^ |
| *ANP32B* | 16 | 3.51 x 10^-2^ | 4.64 x 10^-1^ |
| *ANPEP* | 23 | 1.33 x 10^-1^ | 8.36 x 10^-1^ |
| *ANXA1* | 41 | 6.22 x 10^-3^ | 2.55 x 10^-1^ |
| *ANXA11* | 27 | 1.68 x 10^-3^ | 1.47 x 10^-2^ |
| *ANXA2* | 34 | 5.35 x 10^-2^ | 5.02 x 10^-1^ |
| *ANXA4* | 26 | 5.27 x 10^-2^ | 6.53 x 10^-1^ |
| *ANXA5* | 38 | 3.71 x 10^-3^ | 6.64 x 10^-2^ |
| *ANXA6* | 93 | 4.22 x 10^-3^ | 1.57 x 10^-1^ |
| *ANXA7* | 6 | 3.40 x 10^-1^ | 8.83 x 10^-1^ |
| *ANXA8* | 3 | 2.44 x 10^-1^ | 3.62 x 10^-1^ |
| *AOC3* | 7 | 2.96 x 10^-1^ | 1.00 |
| *AP1B1* | 23 | 3.40 x 10^-1^ | 9.45 x 10^-1^ |
| *AP2A2* | 19 | 2.14 x 10^-1^ | 5.27 x 10^-1^ |
| *AP2B1* | 39 | 1.67 x 10^-3^ | 1.16 x 10^-2^ |
| *AP2M1* | 24 | 3.54 x 10^-2^ | 2.60 x 10^-1^ |
| *AP2S1* | 12 | 3.07 x 10^-2^ | 3.16 x 10^-1^ |
| *AP3D1* | 24 | 5.00 x 10^-2^ | 5.41 x 10^-1^ |
| *AP3S1* | 31 | 1.28 x 10^-3^ | 3.69 x 10^-2^ |
| *AP3S2* | 25 | 8.21 x 10^-2^ | 8.75 x 10^-1^ |
| *APBB1* | 24 | 5.37 x 10^-2^ | 3.54 x 10^-1^ |
| *APCDD1* | 41 | 1.58 x 10^-2^ | 6.34 x 10^-1^ |
| *APEH* | 5 | 3.54 x 10^-1^ | 1.00 |
| *APEX1* | 35 | 1.30 x 10^-3^ | 4.39 x 10^-2^ |
| *APH1A* | 13 | 3.09 x 10^-2^ | 2.91 x 10^-1^ |
| *APLP2* | 24 | 5.79 x 10^-2^ | 7.21 x 10^-1^ |
| *APOC1* | 16 | 7.24 x 10^-3^ | 9.62 x 10^-2^ |
| *APOC2* | 12 | 1.64 x 10^-1^ | 8.38 x 10^-1^ |
| *APOD* | 3 | 3.00 x 10^-1^ | 4.19 x 10^-1^ |
| *APOE* | 19 | 7.24 x 10^-3^ | 1.23 x 10^-1^ |
| *APOL3* | 49 | 4.15 x 10^-3^ | 1.05 x 10^-1^ |
| *APP* | 88 | 6.26 x 10^-2^ | 9.43 x 10^-1^ |
| *APRT* | 27 | 4.03 x 10^-2^ | 7.21 x 10^-1^ |
| *AQP1* | 37 | 9.49 x 10^-2^ | 8.53 x 10^-1^ |
| *AQP2* | 25 | 6.51 x 10^-2^ | 9.16 x 10^-1^ |
| *AQP3* | 31 | 5.45 x 10^-2^ | 6.35 x 10^-1^ |
| *ARCN1* | 12 | 4.05 x 10^-1^ | 9.47 x 10^-1^ |
| *ARF1* | 11 | 1.96 x 10^-1^ | 8.39 x 10^-1^ |
| *ARF3* | 11 | 2.91 x 10^-1^ | 5.87 x 10^-1^ |
| *ARF4* | 15 | 2.48 x 10^-2^ | 6.90 x 10^-2^ |
| *ARF5* | 17 | 1.10 x 10^-1^ | 8.90 x 10^-1^ |
| *ARF6* | 9 | 2.42 x 10^-2^ | 4.14 x 10^-2^ |
| *ARFGAP1* | 17 | 2.18 x 10^-2^ | 1.83 x 10^-1^ |
| *ARFGAP2* | 13 | 1.90 x 10^-2^ | 1.44 x 10^-1^ |
| *ARFIP2* | 25 | 8.04 x 10^-3^ | 1.37 x 10^-1^ |
| *ARHGAP1* | 9 | 1.32 x 10^-2^ | 3.48 x 10^-2^ |
| *ARHGAP29* | 27 | 6.99 x 10^-2^ | 8.57 x 10^-1^ |
| *ARHGAP9* | 11 | 3.04 x 10^-1^ | 8.13 x 10^-1^ |
| *ARHGDIB* | 30 | 1.13 x 10^-1^ | 7.26 x 10^-1^ |
| *ARHGEF1* | 11 | 1.00 x 10^-1^ | 3.35 x 10^-1^ |
| *ARHGEF10L* | 52 | 1.74 x 10^-2^ | 3.38 x 10^-1^ |
| *ARHGEF15* | 24 | 1.42 x 10^-2^ | 2.74 x 10^-1^ |
| *ARHGEF18* | 30 | 1.59 x 10^-1^ | 8.50 x 10^-1^ |
| *ARHGEF2* | 10 | 1.51 x 10^-1^ | 7.32 x 10^-1^ |
| *ARHGEF7* | 66 | 3.47 x 10^-3^ | 9.99 x 10^-2^ |
| *ARID1A* | 6 | 1.97 x 10^-1^ | 9.44 x 10^-1^ |
| *ARL2BP* | 10 | 5.30 x 10^-2^ | 4.68 x 10^-1^ |
| *ARL5A* | 11 | 1.68 x 10^-2^ | 1.51 x 10^-1^ |
| *ARL6IP4* | 4 | 3.58 x 10^-1^ | 8.79 x 10^-1^ |
| *ARL6IP5* | 23 | 2.73 x 10^-2^ | 3.25 x 10^-1^ |
| *ARL8B* | 22 | 6.00 x 10^-3^ | 9.18 x 10^-2^ |
| *ARMC10* | 5 | 2.77 x 10^-1^ | 9.83 x 10^-1^ |
| *ARMET* | 18 | 1.60 x 10^-1^ | 5.97 x 10^-1^ |
| *ARPC1A* | 12 | 2.78 x 10^-2^ | 2.21 x 10^-1^ |
| *ARPC1B* | 9 | 2.78 x 10^-2^ | 1.80 x 10^-1^ |
| *ARPC2* | 14 | 6.15 x 10^-2^ | 5.36 x 10^-1^ |
| *ARPC4* | 16 | 1.62 x 10^-2^ | 2.50 x 10^-1^ |
| *ARPC5* | 10 | 3.86 x 10^-2^ | 1.93 x 10^-1^ |
| *ARPC5L* | 14 | 9.19 x 10^-2^ | 3.23 x 10^-1^ |
| *ARPM1* | 6 | 5.66 x 10^-2^ | 1.55 x 10^-1^ |
| *ARRB2* | 30 | 1.04 x 10^-2^ | 2.48 x 10^-1^ |
| *ARRDC2* | 24 | 5.66 x 10^-2^ | 4.62 x 10^-1^ |
| *ARS2* | 15 | 1.60 x 10^-2^ | 1.77 x 10^-1^ |
| *ARSA* | 29 | 3.48 x 10^-2^ | 5.78 x 10^-1^ |
| *ARSG* | 44 | 1.11 x 10^-2^ | 4.76 x 10^-1^ |
| *ART1* | 37 | 2.84 x 10^-2^ | 2.49 x 10^-1^ |
| *ASAH1* | 61 | 2.57 x 10^-2^ | 5.87 x 10^-1^ |
| *ASB13* | 58 | 3.85 x 10^-2^ | 8.90 x 10^-1^ |
| *ASCC3L1* | 11 | 6.09 x 10^-3^ | 6.08 x 10^-2^ |
| *ASF1B* | 7 | 5.87 x 10^-2^ | 8.91 x 10^-2^ |
| *ASH2L* | 5 | 8.89 x 10^-2^ | 4.44 x 10^-1^ |
| *ASL* | 10 | 5.13 x 10^-1^ | 8.29 x 10^-1^ |
| *ASNA1* | 5 | 3.20 x 10^-2^ | 1.60 x 10^-1^ |
| *ASRGL1* | 38 | 3.30 x 10^-2^ | 9.86 x 10^-1^ |
| *ASS1* | 63 | 3.16 x 10^-2^ | 9.34 x 10^-1^ |
| *ASXL1* | 6 | 3.01 x 10^-1^ | 5.21 x 10^-1^ |
| *ASXL2* | 23 | 7.00 x 10^-2^ | 5.06 x 10^-1^ |
| *ATAD4* | 16 | 6.76 x 10^-2^ | 7.50 x 10^-1^ |
| *ATF3* | 55 | 5.54 x 10^-2^ | 9.82 x 10^-1^ |
| *ATF4* | 20 | 6.77 x 10^-2^ | 6.51 x 10^-1^ |
| *ATG2A* | 15 | 4.55 x 10^-2^ | 5.42 x 10^-1^ |
| *ATG9A* | 19 | 1.11 x 10^-1^ | 9.73 x 10^-1^ |
| *ATIC* | 26 | 2.38 x 10^-3^ | 2.85 x 10^-2^ |
| *ATOH8* | 45 | 1.27 x 10^-3^ | 2.17 x 10^-2^ |
| *ATOX1* | 11 | 8.69 x 10^-2^ | 2.72 x 10^-1^ |
| *ATP13A1* | 16 | 6.25 x 10^-2^ | 8.46 x 10^-1^ |
| *ATP1A1* | 8 | 4.94 x 10^-3^ | 3.63 x 10^-2^ |
| *ATP1B1* | 35 | 1.99 x 10^-2^ | 3.79 x 10^-1^ |
| *ATP1B3* | 21 | 8.45 x 10^-2^ | 7.75 x 10^-1^ |
| *ATP2A2* | 6 | 2.41 x 10^-1^ | 1.00 |
| *ATP2B4* | 37 | 1.07 x 10^-1^ | 9.79 x 10^-1^ |
| *ATP5A1* | 17 | 6.37 x 10^-2^ | 5.21 x 10^-1^ |
| *ATP5B* | 13 | 7.88 x 10^-2^ | 3.66 x 10^-1^ |
| *ATP5C1* | 34 | 1.76 x 10^-2^ | 4.01 x 10^-1^ |
| *ATP5D* | 7 | 3.71 x 10^-1^ | 8.19 x 10^-1^ |
| *ATP5E* | 18 | 6.37 x 10^-2^ | 2.38 x 10^-1^ |
| *ATP5F1* | 23 | 1.25 x 10^-1^ | 4.82 x 10^-1^ |
| *ATP5G1* | 12 | 1.74 x 10^-3^ | 5.83 x 10^-3^ |
| *ATP5G2* | 24 | 1.03 x 10^-1^ | 1.00 |
| *ATP5G3* | 6 | 4.15 x 10^-1^ | 6.88 x 10^-1^ |
| *ATP5H* | 19 | 1.14 x 10^-1^ | 9.67 x 10^-1^ |
| *ATP5I* | 8 | 6.27 x 10^-2^ | 2.09 x 10^-1^ |
| *ATP5J* | 14 | 7.43 x 10^-2^ | 5.50 x 10^-1^ |
| *ATP5J2* | 5 | 8.14 x 10^-2^ | 2.74 x 10^-1^ |
| *ATP5L* | 9 | 7.05 x 10^-2^ | 6.35 x 10^-1^ |
| *ATP5O* | 33 | 2.69 x 10^-2^ | 3.61 x 10^-1^ |
| *ATP5SL* | 17 | 1.13 x 10^-3^ | 5.91 x 10^-3^ |
| *ATP6V0A1* | 12 | 7.58 x 10^-2^ | 5.12 x 10^-1^ |
| *ATP6V0B* | 16 | 1.03 x 10^-2^ | 1.33 x 10^-1^ |
| *ATP6V0C* | 1 | 4.29 x 10^-1^ | 3.68 x 10^-1^ |
| *ATP6V0D1* | 5 | 1.75 x 10^-2^ | 8.59 x 10^-2^ |
| *ATP6V0E1* | 22 | 6.04 x 10^-2^ | 6.48 x 10^-1^ |
| *ATP6V0E2* | 15 | 3.01 x 10^-1^ | 9.96 x 10^-1^ |
| *ATP6V1A* | 8 | 9.53 x 10^-2^ | 3.44 x 10^-1^ |
| *ATP6V1B2* | 37 | 1.90 x 10^-1^ | 9.68 x 10^-1^ |
| *ATP6V1E1* | 29 | 1.62 x 10^-2^ | 1.56 x 10^-1^ |
| *ATP6V1F* | 15 | 3.94 x 10^-1^ | 9.71 x 10^-1^ |
| *ATP6V1G1* | 33 | 7.43 x 10^-2^ | 8.78 x 10^-1^ |
| *ATP6V1H* | 18 | 1.24 x 10^-2^ | 2.23 x 10^-1^ |
| *ATP8B1* | 47 | 5.66 x 10^-2^ | 9.05 x 10^-1^ |
| *ATP8B2* | 24 | 2.24 x 10^-2^ | 2.33 x 10^-1^ |
| *ATPAF1* | 23 | 6.77 x 10^-2^ | 2.58 x 10^-1^ |
| *ATPIF1* | 12 | 1.04 x 10^-1^ | 2.81 x 10^-1^ |
| *ATXN10* | 66 | 9.64 x 10^-3^ | 2.73 x 10^-1^ |
| *AUP1* | 3 | 5.80 x 10^-1^ | 9.59 x 10^-1^ |
| *AURKC* | 23 | 1.99 x 10^-2^ | 2.26 x 10^-1^ |
| *AVPI1* | 15 | 6.39 x 10^-2^ | 3.54 x 10^-1^ |
| *AXIN1* | 36 | 4.60 x 10^-2^ | 8.01 x 10^-1^ |
| *AXL* | 19 | 1.86 x 10^-1^ | 8.68 x 10^-1^ |
| *AXUD1* | 25 | 2.05 x 10^-2^ | 5.13 x 10^-1^ |
| *B2M* | 13 | 2.30 x 10^-1^ | 6.93 x 10^-1^ |
| *B3GALT4* | 14 | 2.20 x 10^-2^ | 8.70 x 10^-2^ |
| *B3GNT6* | 13 | 1.06 x 10^-3^ | 1.18 x 10^-2^ |
| *B4GALT2* | 20 | 1.03 x 10^-2^ | 1.84 x 10^-1^ |
| *B4GALT3* | 18 | 7.05 x 10^-2^ | 2.68 x 10^-1^ |
| *B4GALT5* | 38 | 1.20 x 10^-3^ | 2.30 x 10^-2^ |
| *BACE2* | 88 | 5.67 x 10^-4^ | 3.89 x 10^-2^ |
| *BAD* | 16 | 1.88 x 10^-2^ | 2.89 x 10^-1^ |
| *BAG1* | 18 | 5.04 x 10^-2^ | 5.14 x 10^-1^ |
| *BAG3* | 34 | 1.55 x 10^-2^ | 1.38 x 10^-1^ |
| *BAMBI* | 30 | 2.57 x 10^-1^ | 9.64 x 10^-1^ |
| *BANF1* | 18 | 4.44 x 10^-2^ | 3.49 x 10^-1^ |
| *BANP* | 20 | 1.39 x 10^-2^ | 5.03 x 10^-2^ |
| *BAP1* | 4 | 1.93 x 10^-1^ | 7.38 x 10^-1^ |
| *BASP1* | 37 | 5.88 x 10^-2^ | 9.41 x 10^-1^ |
| *BAT1* | 51 | 6.03 x 10^-2^ | 8.73 x 10^-1^ |
| *BAT3* | 31 | 1.30 x 10^-2^ | 2.22 x 10^-1^ |
| *BAT5* | 28 | 1.30 x 10^-2^ | 1.34 x 10^-1^ |
| *BBC3* | 9 | 4.51 x 10^-2^ | 3.78 x 10^-1^ |
| *BCAM* | 15 | 1.29 x 10^-1^ | 9.50 x 10^-1^ |
| *BCAS3* | 81 | 6.95 x 10^-3^ | 5.56 x 10^-1^ |
| *BCAS4* | 38 | 4.09 x 10^-2^ | 3.98 x 10^-1^ |
| *BCKDHA* | 18 | 3.32 x 10^-5^ | 1.84 x 10^-4^ |
| *BCKDK* | 6 | 9.82 x 10^-2^ | 1.11 x 10^-1^ |
| *BCL2L2* | 14 | 1.12 x 10^-1^ | 8.50 x 10^-1^ |
| *BCL6* | 29 | 2.42 x 10^-2^ | 3.42 x 10^-1^ |
| *BCL7B* | 4 | 4.37 x 10^-2^ | 1.39 x 10^-1^ |
| *BCL7C* | 5 | 2.56 x 10^-1^ | 6.47 x 10^-1^ |
| *BCS1L* | 9 | 1.45 x 10^-2^ | 1.87 x 10^-2^ |
| *BFAR* | 2 | 9.29 x 10^-2^ | 1.08 x 10^-1^ |
| *BHLHB2* | 29 | 6.31 x 10^-2^ | 6.67 x 10^-1^ |
| *BIN1* | 48 | 1.01 x 10^-2^ | 4.76 x 10^-1^ |
| *BLCAP* | 17 | 1.20 x 10^-1^ | 8.65 x 10^-1^ |
| *BLOC1S1* | 12 | 9.94 x 10^-2^ | 9.52 x 10^-1^ |
| *BLVRA* | 17 | 6.63 x 10^-1^ | 9.44 x 10^-1^ |
| *BLVRB* | 15 | 5.27 x 10^-3^ | 7.90 x 10^-2^ |
| *BNIP3L* | 29 | 9.38 x 10^-2^ | 6.89 x 10^-1^ |
| *BOC* | 44 | 8.50 x 10^-5^ | 3.65 x 10^-3^ |
| *BOK* | 16 | 1.63 x 10^-2^ | 2.62 x 10^-1^ |
| *BRD1* | 17 | 7.28 x 10^-2^ | 3.01 x 10^-1^ |
| *BRD2* | 71 | 4.85 x 10^-4^ | 2.62 x 10^-2^ |
| *BRD3* | 27 | 8.10 x 10^-2^ | 9.93 x 10^-1^ |
| *BRD4* | 10 | 6.52 x 10^-2^ | 6.15 x 10^-1^ |
| *BRD9* | 8 | 3.89 x 10^-1^ | 9.20 x 10^-1^ |
| *BRE* | 82 | 7.06 x 10^-3^ | 2.59 x 10^-1^ |
| *BRI3* | 4 | 4.06 x 10^-1^ | 9.23 x 10^-1^ |
| *BRMS1* | 10 | 3.39 x 10^-3^ | 2.63 x 10^-2^ |
| *BRP44* | 11 | 4.05 x 10^-2^ | 3.03 x 10^-1^ |
| *BRP44L* | 15 | 1.73 x 10^-1^ | 3.40 x 10^-1^ |
| *BSG* | 17 | 4.71 x 10^-2^ | 2.90 x 10^-1^ |
| *BST2* | 16 | 1.51 x 10^-1^ | 9.15 x 10^-1^ |
| *BTBD6* | 7 | 3.31 x 10^-1^ | 6.91 x 10^-1^ |
| *BTF3* | 23 | 1.58 x 10^-1^ | 9.65 x 10^-1^ |
| *BTG1* | 23 | 5.60 x 10^-2^ | 5.73 x 10^-1^ |
| *BTG2* | 20 | 1.24 x 10^-1^ | 6.84 x 10^-1^ |
| *BTG3* | 27 | 7.75 x 10^-2^ | 6.46 x 10^-1^ |
| *BTN3A3* | 30 | 1.26 x 10^-1^ | 6.29 x 10^-1^ |
| *BTNL3* | 7 | 5.92 x 10^-1^ | 8.57 x 10^-1^ |
| *BUD31* | 7 | 7.30 x 10^-2^ | 2.33 x 10^-1^ |
| *BZW2* | 61 | 1.81 x 10^-2^ | 4.62 x 10^-1^ |
| *C10orf10* | 23 | 4.35 x 10^-2^ | 9.46 x 10^-1^ |
| *C10orf116* | 18 | 9.82 x 10^-2^ | 5.81 x 10^-1^ |
| *C10orf125* | 10 | 4.20 x 10^-1^ | 9.04 x 10^-1^ |
| *C10orf128* | 34 | 2.90 x 10^-1^ | 9.43 x 10^-1^ |
| *C10orf26* | 31 | 1.10 x 10^-1^ | 8.06 x 10^-1^ |
| *C10orf35* | 53 | 1.23 x 10^-2^ | 3.61 x 10^-1^ |
| *C10orf46* | 29 | 1.89 x 10^-1^ | 9.39 x 10^-1^ |
| *C10orf58* | 16 | 7.55 x 10^-3^ | 5.13 x 10^-2^ |
| *C11orf10* | 21 | 7.24 x 10^-2^ | 4.59 x 10^-1^ |
| *C11orf2* | 19 | 1.46 x 10^-1^ | 1.00 |
| *C11orf49* | 28 | 1.90 x 10^-2^ | 2.08 x 10^-1^ |
| *C11orf51* | 12 | 1.90 x 10^-1^ | 6.34 x 10^-1^ |
| *C11orf58* | 21 | 1.26 x 10^-1^ | 7.04 x 10^-1^ |
| *C11orf59* | 9 | 2.73 x 10^-1^ | 6.01 x 10^-1^ |
| *C11orf68* | 9 | 7.46 x 10^-2^ | 7.80 x 10^-2^ |
| *C11orf75* | 36 | 1.84 x 10^-2^ | 5.51 x 10^-1^ |
| *C11orf79* | 11 | 1.70 x 10^-1^ | 6.57 x 10^-1^ |
| *C12orf10* | 11 | 2.77 x 10^-1^ | 7.91 x 10^-1^ |
| *C12orf34* | 21 | 1.50 x 10^-1^ | 7.93 x 10^-1^ |
| *C12orf39* | 44 | 3.89 x 10^-2^ | 3.62 x 10^-1^ |
| *C12orf45* | 18 | 1.73 x 10^-1^ | 5.39 x 10^-1^ |
| *C12orf49* | 23 | 2.46 x 10^-2^ | 4.51 x 10^-1^ |
| *C12orf57* | 11 | 3.17 x 10^-2^ | 2.52 x 10^-1^ |
| *C13orf15* | 23 | 1.65 x 10^-2^ | 3.07 x 10^-1^ |
| *C13orf33* | 42 | 7.61 x 10^-2^ | 8.21 x 10^-1^ |
| *C14orf119* | 32 | 6.26 x 10^-2^ | 5.53 x 10^-1^ |
| *C14orf124* | 36 | 2.79 x 10^-3^ | 6.69 x 10^-2^ |
| *C14orf133* | 28 | 9.56 x 10^-2^ | 6.24 x 10^-1^ |
| *C14orf147* | 14 | 6.82 x 10^-2^ | 7.35 x 10^-1^ |
| *C14orf153* | 15 | 1.23 x 10^-2^ | 7.95 x 10^-2^ |
| *C14orf156* | 21 | 2.70 x 10^-2^ | 5.67 x 10^-1^ |
| *C14orf166* | 44 | 3.90 x 10^-3^ | 1.29 x 10^-1^ |
| *C14orf2* | 16 | 2.17 x 10^-2^ | 2.16 x 10^-1^ |
| *C14orf4* | 33 | 1.43 x 10^-4^ | 4.74 x 10^-3^ |
| *C14orf94* | 13 | 3.24 x 10^-1^ | 8.27 x 10^-1^ |
| *C15orf2* | 18 | 4.33 x 10^-2^ | 3.33 x 10^-1^ |
| *C15orf39* | 8 | 9.66 x 10^-2^ | 1.49 x 10^-1^ |
| *C16orf13* | 17 | 1.99 x 10^-1^ | 9.55 x 10^-1^ |
| *C16orf14* | 18 | 1.99 x 10^-1^ | 9.55 x 10^-1^ |
| *C16orf33* | 12 | 3.97 x 10^-2^ | 3.52 x 10^-1^ |
| *C16orf35* | 20 | 5.48 x 10^-3^ | 9.61 x 10^-2^ |
| *C16orf46* | 21 | 1.28 x 10^-2^ | 4.68 x 10^-2^ |
| *C16orf80* | 16 | 9.17 x 10^-2^ | 9.55 x 10^-1^ |
| *C16orf89* | 27 | 7.52 x 10^-2^ | 7.13 x 10^-1^ |
| *C17orf28* | 19 | 8.18 x 10^-2^ | 3.86 x 10^-1^ |
| *C17orf37* | 2 | 6.04 x 10^-2^ | 3.25 x 10^-2^ |
| *C17orf62* | 15 | 2.75 x 10^-1^ | 8.97 x 10^-1^ |
| *C17orf63* | 14 | 2.96 x 10^-1^ | 9.72 x 10^-1^ |
| *C17orf70* | 15 | 1.54 x 10^-2^ | 6.27 x 10^-2^ |
| *C17orf71* | 5 | 3.45 x 10^-3^ | 8.81 x 10^-3^ |
| *C17orf79* | 13 | 8.80 x 10^-3^ | 5.12 x 10^-2^ |
| *C18orf10* | 16 | 2.50 x 10^-3^ | 1.55 x 10^-2^ |
| *C18orf45* | 41 | 3.54 x 10^-3^ | 4.04 x 10^-2^ |
| *C18orf8* | 18 | 4.24 x 10^-3^ | 4.38 x 10^-2^ |
| *C19orf10* | 21 | 9.54 x 10^-2^ | 9.12 x 10^-1^ |
| *C19orf12* | 25 | 9.78 x 10^-2^ | 7.12 x 10^-1^ |
| *C19orf21* | 26 | 2.79 x 10^-2^ | 7.25 x 10^-1^ |
| *C19orf22* | 21 | 9.19 x 10^-2^ | 9.91 x 10^-1^ |
| *C19orf29* | 20 | 7.63 x 10^-2^ | 8.50 x 10^-1^ |
| *C19orf39* | 7 | 1.33 x 10^-1^ | 9.28 x 10^-1^ |
| *C19orf42* | 12 | 6.12 x 10^-2^ | 3.28 x 10^-1^ |
| *C19orf43* | 5 | 3.20 x 10^-2^ | 1.60 x 10^-1^ |
| *C19orf53* | 14 | 1.58 x 10^-2^ | 8.33 x 10^-2^ |
| *C19orf54* | 20 | 2.23 x 10^-1^ | 7.96 x 10^-1^ |
| *C19orf56* | 8 | 3.20 x 10^-2^ | 2.56 x 10^-1^ |
| *C19orf60* | 9 | 1.58 x 10^-1^ | 5.80 x 10^-1^ |
| *C19orf61* | 29 | 1.22 x 10^-2^ | 2.66 x 10^-1^ |
| *C19orf62* | 22 | 6.42 x 10^-2^ | 9.62 x 10^-1^ |
| *C19orf66* | 25 | 6.04 x 10^-3^ | 1.50 x 10^-1^ |
| *C1orf106* | 22 | 7.00 x 10^-2^ | 7.12 x 10^-1^ |
| *C1orf115* | 17 | 3.46 x 10^-3^ | 2.05 x 10^-2^ |
| *C1orf116* | 23 | 4.89 x 10^-3^ | 1.03 x 10^-1^ |
| *C1orf123* | 20 | 3.84 x 10^-2^ | 7.29 x 10^-1^ |
| *C1orf144* | 14 | 1.60 x 10^-1^ | 6.94 x 10^-1^ |
| *C1orf149* | 10 | 1.91 x 10^-1^ | 8.98 x 10^-1^ |
| *C1orf162* | 19 | 1.25 x 10^-1^ | 5.52 x 10^-1^ |
| *C1orf198* | 34 | 3.62 x 10^-2^ | 5.09 x 10^-1^ |
| *C1orf216* | 7 | 4.91 x 10^-3^ | 7.54 x 10^-3^ |
| *C1orf38* | 12 | 2.03 x 10^-1^ | 9.01 x 10^-1^ |
| *C1orf43* | 15 | 6.80 x 10^-2^ | 7.24 x 10^-1^ |
| *C1orf54* | 12 | 3.09 x 10^-2^ | 2.99 x 10^-1^ |
| *C1orf59* | 6 | 5.62 x 10^-1^ | 8.65 x 10^-1^ |
| *C1orf66* | 18 | 2.20 x 10^-2^ | 3.90 x 10^-1^ |
| *C1orf85* | 13 | 8.41 x 10^-4^ | 9.49 x 10^-3^ |
| *C1QA* | 23 | 4.92 x 10^-2^ | 8.06 x 10^-1^ |
| *C1QB* | 20 | 1.30 x 10^-1^ | 8.86 x 10^-1^ |
| *C1QC* | 21 | 1.30 x 10^-1^ | 9.20 x 10^-1^ |
| *C1QTNF1* | 24 | 1.98 x 10^-1^ | 9.10 x 10^-1^ |
| *C1QTNF5* | 24 | 3.35 x 10^-2^ | 8.04 x 10^-1^ |
| *C1R* | 27 | 2.50 x 10^-3^ | 6.75 x 10^-2^ |
| *C1S* | 9 | 2.50 x 10^-3^ | 1.60 x 10^-2^ |
| *C2* | 27 | 6.09 x 10^-3^ | 6.25 x 10^-2^ |
| *C20orf108* | 34 | 4.86 x 10^-3^ | 1.65 x 10^-1^ |
| *C20orf11* | 29 | 1.71 x 10^-2^ | 4.96 x 10^-1^ |
| *C20orf111* | 37 | 5.25 x 10^-2^ | 7.98 x 10^-1^ |
| *C20orf114* | 24 | 9.31 x 10^-3^ | 1.21 x 10^-1^ |
| *C20orf116* | 28 | 8.56 x 10^-4^ | 2.22 x 10^-2^ |
| *C20orf149* | 15 | 1.71 x 10^-1^ | 9.05 x 10^-1^ |
| *C20orf160* | 18 | 1.40 x 10^-1^ | 9.80 x 10^-1^ |
| *C20orf24* | 7 | 6.23 x 10^-2^ | 3.72 x 10^-1^ |
| *C20orf29* | 26 | 2.97 x 10^-2^ | 2.26 x 10^-1^ |
| *C20orf3* | 25 | 3.84 x 10^-1^ | 9.61 x 10^-1^ |
| *C20orf30* | 15 | 2.80 x 10^-1^ | 9.63 x 10^-1^ |
| *C20orf4* | 8 | 2.07 x 10^-1^ | 1.00 |
| *C20orf43* | 31 | 1.68 x 10^-2^ | 4.44 x 10^-1^ |
| *C20orf46* | 32 | 3.42 x 10^-1^ | 9.86 x 10^-1^ |
| *C20orf52* | 7 | 3.52 x 10^-1^ | 8.26 x 10^-1^ |
| *C20orf85* | 17 | 2.31 x 10^-1^ | 8.50 x 10^-1^ |
| *C20orf96* | 32 | 7.02 x 10^-2^ | 9.16 x 10^-1^ |
| *C21orf33* | 21 | 5.36 x 10^-2^ | 9.20 x 10^-1^ |
| *C21orf63* | 34 | 6.33 x 10^-3^ | 1.63 x 10^-1^ |
| *C22orf13* | 17 | 4.58 x 10^-2^ | 8.59 x 10^-2^ |
| *C22orf28* | 59 | 1.14 x 10^-3^ | 5.11 x 10^-2^ |
| *C22orf40* | 21 | 2.78 x 10^-2^ | 3.82 x 10^-1^ |
| *C22orf9* | 54 | 9.66 x 10^-3^ | 4.84 x 10^-1^ |
| *C2orf28* | 11 | 4.18 x 10^-2^ | 6.87 x 10^-2^ |
| *C2orf40* | 31 | 6.39 x 10^-2^ | 9.87 x 10^-1^ |
| *C2orf51* | 11 | 2.54 x 10^-2^ | 3.57 x 10^-2^ |
| *C2orf53* | 11 | 3.45 x 10^-2^ | 1.87 x 10^-1^ |
| *C2orf73* | 33 | 4.63 x 10^-2^ | 5.82 x 10^-1^ |
| *C3* | 36 | 2.48 x 10^-2^ | 8.94 x 10^-1^ |
| *C3AR1* | 15 | 1.32 x 10^-1^ | 9.21 x 10^-1^ |
| *C3orf1* | 35 | 5.14 x 10^-4^ | 9.67 x 10^-3^ |
| *C3orf10* | 10 | 1.61 x 10^-1^ | 5.59 x 10^-1^ |
| *C3orf37* | 14 | 2.06 x 10^-1^ | 8.36 x 10^-1^ |
| *C3orf38* | 4 | 3.08 x 10^-1^ | 9.49 x 10^-1^ |
| *C3orf60* | 5 | 1.26 x 10^-1^ | 1.89 x 10^-1^ |
| *C4A* | 30 | 1.94 x 10^-2^ | 5.83 x 10^-1^ |
| *C4BPA* | 25 | 2.26 x 10^-2^ | 2.87 x 10^-1^ |
| *C4orf14* | 22 | 5.87 x 10^-2^ | 9.32 x 10^-1^ |
| *C4orf31* | 21 | 5.48 x 10^-2^ | 6.70 x 10^-1^ |
| *C4orf8* | 18 | 2.94 x 10^-2^ | 4.54 x 10^-1^ |
| *C5orf15* | 22 | 7.01 x 10^-3^ | 7.70 x 10^-2^ |
| *C5orf32* | 16 | 1.16 x 10^-1^ | 9.14 x 10^-1^ |
| *C5orf37* | 31 | 6.41 x 10^-2^ | 7.00 x 10^-1^ |
| *C5orf4* | 8 | 3.44 x 10^-2^ | 1.66 x 10^-1^ |
| *C5orf49* | 44 | 5.07 x 10^-2^ | 9.58 x 10^-1^ |
| *C6orf1* | 6 | 8.49 x 10^-2^ | 5.09 x 10^-1^ |
| *C6orf105* | 68 | 1.39 x 10^-2^ | 8.73 x 10^-1^ |
| *C6orf108* | 19 | 1.62 x 10^-2^ | 1.13 x 10^-1^ |
| *C6orf125* | 47 | 8.26 x 10^-3^ | 1.45 x 10^-1^ |
| *C6orf145* | 32 | 5.22 x 10^-2^ | 8.55 x 10^-1^ |
| *C6orf153* | 9 | 4.95 x 10^-1^ | 9.62 x 10^-1^ |
| *C6orf206* | 14 | 1.49 x 10^-1^ | 5.86 x 10^-1^ |
| *C6orf48* | 14 | 6.09 x 10^-3^ | 6.29 x 10^-2^ |
| *C6orf72* | 9 | 9.93 x 10^-2^ | 8.94 x 10^-1^ |
| *C6orf89* | 37 | 8.74 x 10^-2^ | 9.30 x 10^-1^ |
| *C7* | 46 | 2.67 x 10^-2^ | 6.12 x 10^-1^ |
| *C7orf23* | 15 | 3.80 x 10^-1^ | 9.33 x 10^-1^ |
| *C7orf26* | 11 | 8.17 x 10^-3^ | 5.58 x 10^-2^ |
| *C7orf27* | 19 | 2.65 x 10^-1^ | 9.64 x 10^-1^ |
| *C7orf28B* | 1 | 9.39 x 10^-1^ | 9.39 x 10^-1^ |
| *C7orf30* | 15 | 1.48 x 10^-1^ | 5.87 x 10^-1^ |
| *C7orf33* | 23 | 1.82 x 10^-1^ | 7.61 x 10^-1^ |
| *C7orf41* | 27 | 2.14 x 10^-2^ | 2.09 x 10^-1^ |
| *C7orf42* | 11 | 3.48 x 10^-2^ | 3.82 x 10^-1^ |
| *C7orf49* | 11 | 1.59 x 10^-1^ | 8.21 x 10^-1^ |
| *C7orf50* | 34 | 9.87 x 10^-3^ | 3.36 x 10^-1^ |
| *C8orf30A* | 3 | 3.53 x 10^-1^ | 8.32 x 10^-1^ |
| *C9orf150* | 49 | 4.30 x 10^-3^ | 2.11 x 10^-1^ |
| *C9orf16* | 15 | 1.38 x 10^-1^ | 5.24 x 10^-1^ |
| *C9orf23* | 13 | 2.23 x 10^-1^ | 9.22 x 10^-1^ |
| *C9orf3* | 51 | 3.17 x 10^-3^ | 1.58 x 10^-1^ |
| *C9orf37* | 5 | 2.65 x 10^-1^ | 1.00 |
| *C9orf5* | 41 | 1.58 x 10^-2^ | 2.39 x 10^-1^ |
| *C9orf61* | 36 | 1.28 x 10^-1^ | 9.58 x 10^-1^ |
| *C9orf64* | 12 | 2.85 x 10^-1^ | 9.77 x 10^-1^ |
| *C9orf7* | 7 | 9.97 x 10^-2^ | 3.96 x 10^-1^ |
| *C9orf75* | 2 | 2.99 x 10^-1^ | 5.98 x 10^-1^ |
| *C9orf78* | 27 | 2.40 x 10^-2^ | 5.09 x 10^-1^ |
| *CA4* | 8 | 3.62 x 10^-1^ | 6.27 x 10^-1^ |
| *CA7* | 6 | 2.79 x 10^-1^ | 6.25 x 10^-1^ |
| *CA9* | 25 | 1.36 x 10^-1^ | 7.33 x 10^-1^ |
| *CABIN1* | 20 | 1.36 x 10^-2^ | 2.73 x 10^-1^ |
| *CABLES1* | 36 | 4.91 x 10^-2^ | 7.17 x 10^-1^ |
| *CABP7* | 35 | 3.82 x 10^-3^ | 5.19 x 10^-2^ |
| *CACNA2D2* | 18 | 5.73 x 10^-2^ | 7.86 x 10^-1^ |
| *CACNG4* | 39 | 1.17 x 10^-2^ | 4.55 x 10^-1^ |
| *CADM1* | 94 | 2.37 x 10^-3^ | 2.18 x 10^-1^ |
| *CALCOCO1* | 28 | 4.92 x 10^-2^ | 6.44 x 10^-1^ |
| *CALCOCO2* | 22 | 1.74 x 10^-3^ | 8.64 x 10^-3^ |
| *CALHM2* | 29 | 4.14 x 10^-2^ | 3.66 x 10^-1^ |
| *CALM1* | 25 | 1.74 x 10^-2^ | 3.32 x 10^-1^ |
| *CALM2* | 24 | 3.27 x 10^-1^ | 9.90 x 10^-1^ |
| *CALM3* | 19 | 8.51 x 10^-2^ | 9.72 x 10^-1^ |
| *CALR* | 11 | 1.04 x 10^-3^ | 6.06 x 10^-3^ |
| *CAMK1* | 15 | 1.62 x 10^-2^ | 2.26 x 10^-1^ |
| *CAMK2N1* | 27 | 3.96 x 10^-2^ | 8.43 x 10^-1^ |
| *CAMKK1* | 31 | 1.08 x 10^-1^ | 9.18 x 10^-1^ |
| *CAMKK2* | 24 | 2.93 x 10^-2^ | 4.43 x 10^-1^ |
| *CAMLG* | 6 | 2.07 x 10^-1^ | 4.59 x 10^-1^ |
| *CAMTA1* | 295 | 8.19 x 10^-4^ | 1.55 x 10^-1^ |
| *CAMTA2* | 17 | 3.36 x 10^-1^ | 9.21 x 10^-1^ |
| *CANX* | 11 | 1.41 x 10^-1^ | 7.59 x 10^-1^ |
| *CAP1* | 30 | 1.73 x 10^-1^ | 6.59 x 10^-1^ |
| *CAPG* | 20 | 4.56 x 10^-2^ | 3.82 x 10^-1^ |
| *CAPN2* | 38 | 7.26 x 10^-2^ | 6.07 x 10^-1^ |
| *CAPNS1* | 16 | 9.35 x 10^-2^ | 9.38 x 10^-1^ |
| *CAPS* | 14 | 5.42 x 10^-2^ | 3.41 x 10^-1^ |
| *CAPZB* | 62 | 1.29 x 10^-3^ | 2.99 x 10^-2^ |
| *CARD6* | 17 | 3.36 x 10^-2^ | 2.70 x 10^-1^ |
| *CARHSP1* | 25 | 6.24 x 10^-2^ | 6.60 x 10^-1^ |
| *CARM1* | 11 | 1.23 x 10^-1^ | 7.15 x 10^-1^ |
| *CARS* | 31 | 7.22 x 10^-2^ | 5.13 x 10^-1^ |
| *CARS2* | 26 | 3.95 x 10^-2^ | 9.76 x 10^-1^ |
| *CASC3* | 14 | 1.26 x 10^-1^ | 8.71 x 10^-1^ |
| *CASP9* | 17 | 5.24 x 10^-2^ | 3.96 x 10^-1^ |
| *CASZ1* | 56 | 3.26 x 10^-2^ | 9.96 x 10^-1^ |
| *CAT* | 50 | 8.04 x 10^-3^ | 3.08 x 10^-1^ |
| *CAV1* | 33 | 4.35 x 10^-2^ | 5.79 x 10^-1^ |
| *CAV2* | 21 | 1.38 x 10^-1^ | 6.28 x 10^-1^ |
| *CBARA1* | 20 | 3.39 x 10^-2^ | 4.23 x 10^-1^ |
| *CBLN2* | 22 | 2.37 x 10^-1^ | 8.68 x 10^-1^ |
| *CBR1* | 16 | 7.59 x 10^-2^ | 6.10 x 10^-1^ |
| *CBX6* | 15 | 5.48 x 10^-3^ | 5.66 x 10^-2^ |
| *CBX7* | 26 | 3.37 x 10^-1^ | 9.83 x 10^-1^ |
| *CCDC115* | 11 | 1.08 x 10^-2^ | 1.19 x 10^-1^ |
| *CCDC12* | 14 | 1.39 x 10^-2^ | 3.66 x 10^-2^ |
| *CCDC130* | 14 | 1.58 x 10^-2^ | 7.87 x 10^-2^ |
| *CCDC142* | 6 | 5.80 x 10^-1^ | 7.99 x 10^-1^ |
| *CCDC23* | 26 | 6.87 x 10^-2^ | 4.87 x 10^-1^ |
| *CCDC24* | 19 | 1.03 x 10^-2^ | 1.88 x 10^-1^ |
| *CCDC28A* | 28 | 3.04 x 10^-3^ | 6.42 x 10^-2^ |
| *CCDC43* | 8 | 6.10 x 10^-2^ | 1.76 x 10^-1^ |
| *CCDC49* | 22 | 4.32 x 10^-2^ | 5.26 x 10^-1^ |
| *CCDC51* | 5 | 4.33 x 10^-1^ | 9.63 x 10^-1^ |
| *CCDC56* | 6 | 5.47 x 10^-1^ | 1.00 |
| *CCDC60* | 86 | 4.16 x 10^-2^ | 7.38 x 10^-1^ |
| *CCDC72* | 5 | 4.33 x 10^-1^ | 9.63 x 10^-1^ |
| *CCDC86* | 23 | 9.96 x 10^-2^ | 9.53 x 10^-1^ |
| *CCDC88C* | 59 | 2.25 x 10^-3^ | 1.33 x 10^-1^ |
| *CCDC9* | 9 | 2.87 x 10^-1^ | 1.00 |
| *CCDC90A* | 23 | 1.84 x 10^-2^ | 3.66 x 10^-1^ |
| *CCDC92* | 26 | 2.60 x 10^-1^ | 9.58 x 10^-1^ |
| *CCL15* | 24 | 8.82 x 10^-2^ | 9.55 x 10^-1^ |
| *CCL18* | 24 | 8.82 x 10^-2^ | 7.82 x 10^-1^ |
| *CCL19* | 20 | 2.36 x 10^-2^ | 2.24 x 10^-1^ |
| *CCL21* | 22 | 2.36 x 10^-2^ | 2.14 x 10^-1^ |
| *CCL4* | 17 | 1.12 x 10^-1^ | 8.98 x 10^-1^ |
| *CCL5* | 6 | 1.32 x 10^-1^ | 2.53 x 10^-1^ |
| *CCM2* | 30 | 1.32 x 10^-1^ | 8.76 x 10^-1^ |
| *CCNB1IP1* | 25 | 9.04 x 10^-3^ | 2.26 x 10^-1^ |
| *CCND1* | 12 | 1.23 x 10^-2^ | 8.97 x 10^-2^ |
| *CCND3* | 24 | 3.60 x 10^-2^ | 4.85 x 10^-1^ |
| *CCNDBP1* | 4 | 6.02 x 10^-2^ | 9.59 x 10^-2^ |
| *CCNI* | 18 | 4.39 x 10^-2^ | 2.02 x 10^-1^ |
| *CCRN4L* | 12 | 2.24 x 10^-1^ | 7.14 x 10^-1^ |
| *CCT2* | 26 | 4.27 x 10^-2^ | 8.69 x 10^-1^ |
| *CCT3* | 26 | 8.41 x 10^-4^ | 2.10 x 10^-2^ |
| *CCT4* | 11 | 2.05 x 10^-1^ | 9.35 x 10^-1^ |
| *CCT5* | 37 | 3.01 x 10^-2^ | 9.10 x 10^-1^ |
| *CCT7* | 16 | 8.36 x 10^-2^ | 6.68 x 10^-1^ |
| *CD14* | 15 | 6.05 x 10^-1^ | 9.33 x 10^-1^ |
| *CD151* | 11 | 9.58 x 10^-3^ | 2.07 x 10^-2^ |
| *CD163* | 23 | 2.99 x 10^-2^ | 1.01 x 10^-1^ |
| *CD164* | 14 | 1.07 x 10^-2^ | 1.15 x 10^-1^ |
| *CD1A* | 10 | 6.57 x 10^-2^ | 6.57 x 10^-1^ |
| *CD2* | 16 | 1.32 x 10^-1^ | 7.64 x 10^-1^ |
| *CD247* | 86 | 3.91 x 10^-3^ | 1.77 x 10^-1^ |
| *CD248* | 10 | 6.73 x 10^-3^ | 5.73 x 10^-2^ |
| *CD300C* | 26 | 2.04 x 10^-1^ | 9.48 x 10^-1^ |
| *CD320* | 23 | 2.78 x 10^-2^ | 1.80 x 10^-1^ |
| *CD34* | 25 | 1.13 x 10^-3^ | 2.61 x 10^-2^ |
| *CD37* | 9 | 1.50 x 10^-2^ | 5.52 x 10^-2^ |
| *CD3D* | 17 | 7.05 x 10^-2^ | 4.73 x 10^-1^ |
| *CD3E* | 24 | 3.30 x 10^-2^ | 3.47 x 10^-1^ |
| *CD4* | 38 | 1.97 x 10^-2^ | 3.27 x 10^-1^ |
| *CD40* | 28 | 2.54 x 10^-3^ | 6.30 x 10^-2^ |
| *CD44* | 73 | 1.04 x 10^-2^ | 5.71 x 10^-1^ |
| *CD47* | 31 | 1.17 x 10^-1^ | 8.97 x 10^-1^ |
| *CD48* | 24 | 9.35 x 10^-3^ | 2.24 x 10^-1^ |
| *CD52* | 26 | 7.71 x 10^-2^ | 5.81 x 10^-1^ |
| *CD53* | 29 | 1.05 x 10^-1^ | 6.47 x 10^-1^ |
| *CD55* | 13 | 6.85 x 10^-2^ | 2.80 x 10^-1^ |
| *CD59* | 42 | 9.94 x 10^-2^ | 9.32 x 10^-1^ |
| *CD63* | 14 | 9.94 x 10^-2^ | 9.52 x 10^-1^ |
| *CD74* | 13 | 2.93 x 10^-2^ | 2.89 x 10^-1^ |
| *CD79A* | 10 | 1.00 x 10^-1^ | 2.86 x 10^-1^ |
| *CD81* | 21 | 1.33 x 10^-2^ | 1.03 x 10^-1^ |
| *CD82* | 53 | 2.12 x 10^-3^ | 1.12 x 10^-1^ |
| *CD83* | 27 | 4.35 x 10^-2^ | 6.21 x 10^-1^ |
| *CD8A* | 13 | 5.41 x 10^-2^ | 6.68 x 10^-1^ |
| *CD9* | 22 | 6.19 x 10^-3^ | 5.03 x 10^-2^ |
| *CD93* | 36 | 1.45 x 10^-2^ | 5.20 x 10^-1^ |
| *CD97* | 21 | 1.10 x 10^-1^ | 7.19 x 10^-1^ |
| *CDC123* | 33 | 5.87 x 10^-2^ | 8.75 x 10^-1^ |
| *CDC25B* | 22 | 2.97 x 10^-2^ | 2.24 x 10^-1^ |
| *CDC26* | 25 | 3.46 x 10^-2^ | 4.51 x 10^-1^ |
| *CDC34* | 18 | 4.71 x 10^-2^ | 5.25 x 10^-1^ |
| *CDC37* | 12 | 8.51 x 10^-2^ | 2.82 x 10^-1^ |
| *CDC42* | 19 | 5.09 x 10^-2^ | 9.18 x 10^-1^ |
| *CDC42BPB* | 32 | 4.48 x 10^-2^ | 7.18 x 10^-1^ |
| *CDC42EP4* | 34 | 3.28 x 10^-2^ | 5.76 x 10^-1^ |
| *CDC42SE1* | 14 | 6.94 x 10^-3^ | 6.33 x 10^-2^ |
| *CDCA4* | 8 | 1.44 x 10^-1^ | 2.90 x 10^-1^ |
| *CDCA7L* | 66 | 1.93 x 10^-2^ | 7.63 x 10^-1^ |
| *CDH1* | 26 | 1.30 x 10^-1^ | 9.81 x 10^-1^ |
| *CDH11* | 36 | 1.95 x 10^-2^ | 3.39 x 10^-1^ |
| *CDH24* | 23 | 7.07 x 10^-2^ | 4.87 x 10^-1^ |
| *CDH5* | 42 | 9.68 x 10^-3^ | 4.06 x 10^-1^ |
| *CDIPT* | 11 | 1.58 x 10^-1^ | 4.27 x 10^-1^ |
| *CDK2AP1* | 7 | 3.79 x 10^-1^ | 7.91 x 10^-1^ |
| *CDK2AP2* | 11 | 6.23 x 10^-2^ | 2.73 x 10^-1^ |
| *CDK4* | 12 | 5.23 x 10^-3^ | 1.85 x 10^-2^ |
| *CDK5RAP3* | 17 | 6.76 x 10^-2^ | 7.79 x 10^-1^ |
| *CDKN1A* | 31 | 4.90 x 10^-2^ | 3.60 x 10^-1^ |
| *CDKN1B* | 32 | 1.63 x 10^-1^ | 9.32 x 10^-1^ |
| *CDKN1C* | 31 | 2.63 x 10^-2^ | 3.00 x 10^-1^ |
| *CEACAM3* | 12 | 6.45 x 10^-2^ | 1.87 x 10^-1^ |
| *CEACAM4* | 18 | 1.32 x 10^-2^ | 2.00 x 10^-1^ |
| *CEACAM5* | 19 | 2.94 x 10^-2^ | 5.18 x 10^-1^ |
| *CEACAM6* | 15 | 6.45 x 10^-2^ | 3.60 x 10^-1^ |
| *CEBPA* | 20 | 5.01 x 10^-2^ | 4.04 x 10^-1^ |
| *CEBPB* | 14 | 7.39 x 10^-2^ | 5.28 x 10^-1^ |
| *CECR1* | 42 | 2.87 x 10^-2^ | 7.60 x 10^-1^ |
| *CECR5* | 43 | 2.87 x 10^-2^ | 6.74 x 10^-1^ |
| *CENTA1* | 17 | 3.86 x 10^-2^ | 3.35 x 10^-1^ |
| *CENTA2* | 8 | 1.11 x 10^-1^ | 1.28 x 10^-1^ |
| *CENTD2* | 31 | 7.28 x 10^-2^ | 7.64 x 10^-1^ |
| *CENTG3* | 21 | 7.50 x 10^-3^ | 5.72 x 10^-2^ |
| *CEP250* | 10 | 2.92 x 10^-3^ | 2.80 x 10^-2^ |
| *CEP27* | 10 | 2.70 x 10^-1^ | 5.88 x 10^-1^ |
| *CER1* | 49 | 1.22 x 10^-3^ | 5.08 x 10^-2^ |
| *CERK* | 38 | 1.06 x 10^-1^ | 1.00 |
| *CES1* | 23 | 4.86 x 10^-2^ | 4.93 x 10^-1^ |
| *CES2* | 7 | 1.69 x 10^-2^ | 1.12 x 10^-1^ |
| *CFD* | 19 | 9.19 x 10^-2^ | 9.81 x 10^-1^ |
| *CFL1* | 12 | 3.67 x 10^-2^ | 7.87 x 10^-2^ |
| *CFLAR* | 10 | 5.68 x 10^-3^ | 2.67 x 10^-2^ |
| *CGGBP1* | 8 | 4.06 x 10^-2^ | 3.25 x 10^-1^ |
| *CGN* | 15 | 7.92 x 10^-2^ | 8.58 x 10^-1^ |
| *CGNL1* | 148 | 3.19 x 10^-2^ | 9.65 x 10^-1^ |
| *CHCHD1* | 8 | 4.73 x 10^-1^ | 9.31 x 10^-1^ |
| *CHCHD2* | 7 | 1.29 x 10^-2^ | 5.76 x 10^-2^ |
| *CHCHD4* | 18 | 4.50 x 10^-2^ | 4.10 x 10^-1^ |
| *CHCHD5* | 16 | 1.37 x 10^-2^ | 2.19 x 10^-1^ |
| *CHCHD8* | 17 | 3.01 x 10^-1^ | 9.48 x 10^-1^ |
| *CHD4* | 17 | 1.69 x 10^-1^ | 9.11 x 10^-1^ |
| *CHERP* | 13 | 2.88 x 10^-2^ | 1.01 x 10^-1^ |
| *CHFR* | 25 | 1.50 x 10^-1^ | 8.61 x 10^-1^ |
| *CHI3L1* | 53 | 7.24 x 10^-3^ | 3.16 x 10^-1^ |
| *CHI3L2* | 41 | 6.59 x 10^-2^ | 9.31 x 10^-1^ |
| *CHIA* | 57 | 6.19 x 10^-2^ | 9.74 x 10^-1^ |
| *CHIC2* | 16 | 1.49 x 10^-3^ | 9.48 x 10^-3^ |
| *CHID1* | 10 | 1.49 x 10^-2^ | 9.24 x 10^-2^ |
| *CHMP1A* | 14 | 3.03 x 10^-2^ | 1.40 x 10^-1^ |
| *CHMP2A* | 9 | 4.44 x 10^-1^ | 9.76 x 10^-1^ |
| *CHMP4B* | 29 | 2.19 x 10^-1^ | 8.99 x 10^-1^ |
| *CHMP7* | 55 | 4.56 x 10^-2^ | 9.82 x 10^-1^ |
| *CHPF* | 15 | 2.75 x 10^-2^ | 2.23 x 10^-1^ |
| *CHPT1* | 27 | 6.83 x 10^-2^ | 9.84 x 10^-1^ |
| *CHRM4* | 4 | 2.89 x 10^-1^ | 2.99 x 10^-1^ |
| *CHRNA6* | 9 | 1.66 x 10^-1^ | 8.14 x 10^-1^ |
| *CHST10* | 17 | 9.74 x 10^-2^ | 8.48 x 10^-1^ |
| *CHST12* | 22 | 1.37 x 10^-1^ | 9.70 x 10^-1^ |
| *CIB1* | 15 | 3.23 x 10^-1^ | 9.04 x 10^-1^ |
| *CIC* | 2 | 3.41 x 10^-1^ | 2.94 x 10^-1^ |
| *CIDEB* | 22 | 4.17 x 10^-2^ | 8.76 x 10^-1^ |
| *CILP* | 16 | 1.25 x 10^-1^ | 9.57 x 10^-1^ |
| *CIRBP* | 8 | 1.03 x 10^-1^ | 8.19 x 10^-1^ |
| *CISH* | 7 | 1.13 x 10^-1^ | 5.73 x 10^-1^ |
| *CIT* | 36 | 8.56 x 10^-2^ | 6.07 x 10^-1^ |
| *CITED2* | 26 | 2.57 x 10^-2^ | 2.14 x 10^-1^ |
| *CIZ1* | 23 | 5.57 x 10^-2^ | 2.94 x 10^-1^ |
| *CKAP4* | 35 | 7.29 x 10^-2^ | 5.24 x 10^-1^ |
| *CKB* | 16 | 1.23 x 10^-2^ | 5.51 x 10^-2^ |
| *CKLF* | 7 | 6.63 x 10^-2^ | 1.01 x 10^-1^ |
| *CLDN18* | 20 | 1.57 x 10^-1^ | 8.67 x 10^-1^ |
| *CLDN19* | 15 | 1.34 x 10^-2^ | 5.30 x 10^-2^ |
| *CLDN4* | 8 | 3.52 x 10^-1^ | 9.45 x 10^-1^ |
| *CLDN5* | 15 | 2.15 x 10^-1^ | 9.89 x 10^-1^ |
| *CLEC16A* | 82 | 9.40 x 10^-3^ | 2.38 x 10^-1^ |
| *CLEC3B* | 21 | 1.12 x 10^-1^ | 8.26 x 10^-1^ |
| *CLIC1* | 15 | 1.87 x 10^-2^ | 1.05 x 10^-1^ |
| *CLIC3* | 12 | 2.04 x 10^-1^ | 9.59 x 10^-1^ |
| *CLIC5* | 84 | 4.98 x 10^-3^ | 3.98 x 10^-1^ |
| *CLIC6* | 35 | 3.20 x 10^-2^ | 4.14 x 10^-1^ |
| *CLK2* | 5 | 2.40 x 10^-1^ | 4.28 x 10^-1^ |
| *CLK3* | 14 | 1.21 x 10^-2^ | 1.58 x 10^-1^ |
| *CLN3* | 3 | 1.66 x 10^-1^ | 1.38 x 10^-1^ |
| *CLNS1A* | 8 | 9.36 x 10^-2^ | 5.05 x 10^-1^ |
| *CLP1* | 13 | 1.04 x 10^-1^ | 3.99 x 10^-1^ |
| *CLPP* | 16 | 4.58 x 10^-2^ | 7.32 x 10^-1^ |
| *CLPTM1* | 14 | 8.57 x 10^-2^ | 9.66 x 10^-1^ |
| *CLSTN1* | 18 | 3.92 x 10^-2^ | 3.85 x 10^-1^ |
| *CLTA* | 24 | 1.65 x 10^-1^ | 9.28 x 10^-1^ |
| *CLTB* | 16 | 3.35 x 10^-3^ | 4.37 x 10^-2^ |
| *CLTC* | 12 | 5.58 x 10^-2^ | 6.70 x 10^-1^ |
| *CLU* | 38 | 7.33 x 10^-2^ | 8.91 x 10^-1^ |
| *CMTM3* | 10 | 7.44 x 10^-2^ | 1.95 x 10^-1^ |
| *CMTM5* | 27 | 1.21 x 10^-1^ | 9.83 x 10^-1^ |
| *CMTM6* | 19 | 3.25 x 10^-3^ | 4.41 x 10^-2^ |
| *CMTM7* | 58 | 2.18 x 10^-3^ | 3.40 x 10^-2^ |
| *CMTM8* | 86 | 2.18 x 10^-3^ | 1.39 x 10^-1^ |
| *CNBP* | 9 | 2.25 x 10^-1^ | 3.54 x 10^-1^ |
| *CNDP2* | 46 | 1.88 x 10^-1^ | 9.51 x 10^-1^ |
| *CNGB1* | 55 | 6.08 x 10^-2^ | 9.77 x 10^-1^ |
| *CNIH* | 17 | 1.16 x 10^-2^ | 3.08 x 10^-2^ |
| *CNN1* | 15 | 1.65 x 10^-2^ | 1.78 x 10^-1^ |
| *CNN2* | 21 | 8.77 x 10^-2^ | 5.76 x 10^-1^ |
| *CNN3* | 40 | 3.45 x 10^-2^ | 9.02 x 10^-1^ |
| *CNNM2* | 20 | 2.09 x 10^-1^ | 7.21 x 10^-1^ |
| *CNNM3* | 8 | 3.09 x 10^-2^ | 1.65 x 10^-1^ |
| *CNOT10* | 17 | 7.50 x 10^-2^ | 3.71 x 10^-1^ |
| *CNOT7* | 28 | 1.36 x 10^-1^ | 7.23 x 10^-1^ |
| *CNP* | 11 | 7.19 x 10^-2^ | 5.37 x 10^-1^ |
| *CNPY2* | 7 | 2.27 x 10^-1^ | 9.16 x 10^-1^ |
| *CNR2* | 1 | 2.59 x 10^-1^ | 1.34 x 10^-1^ |
| *CNRIP1* | 25 | 2.85 x 10^-1^ | 8.89 x 10^-1^ |
| *CNTD2* | 11 | 1.45 x 10^-1^ | 9.62 x 10^-1^ |
| *CNTNAP1* | 7 | 2.37 x 10^-1^ | 7.28 x 10^-1^ |
| *COASY* | 6 | 1.54 x 10^-1^ | 5.99 x 10^-1^ |
| *COBL* | 78 | 2.38 x 10^-2^ | 6.26 x 10^-1^ |
| *COG1* | 23 | 6.85 x 10^-2^ | 2.69 x 10^-1^ |
| *COG2* | 74 | 1.77 x 10^-2^ | 3.99 x 10^-1^ |
| *COL18A1* | 56 | 5.19 x 10^-3^ | 1.25 x 10^-1^ |
| *COL1A1* | 23 | 1.38 x 10^-1^ | 8.85 x 10^-1^ |
| *COL1A2* | 36 | 1.61 x 10^-1^ | 9.44 x 10^-1^ |
| *COL3A1* | 29 | 1.76 x 10^-2^ | 3.01 x 10^-1^ |
| *COL4A1* | 109 | 3.81 x 10^-3^ | 3.49 x 10^-1^ |
| *COL4A2* | 118 | 1.83 x 10^-3^ | 1.14 x 10^-1^ |
| *COL6A1* | 29 | 2.42 x 10^-3^ | 2.41 x 10^-2^ |
| *COL6A2* | 40 | 1.15 x 10^-3^ | 1.52 x 10^-2^ |
| *COL6A3* | 64 | 1.40 x 10^-3^ | 7.06 x 10^-2^ |
| *COLEC12* | 128 | 2.19 x 10^-2^ | 8.48 x 10^-1^ |
| *COMMD1* | 23 | 3.75 x 10^-2^ | 8.62 x 10^-1^ |
| *COMMD4* | 4 | 3.52 x 10^-1^ | 9.87 x 10^-1^ |
| *COMMD9* | 34 | 4.01 x 10^-2^ | 5.79 x 10^-1^ |
| *COMT* | 47 | 1.44 x 10^-1^ | 9.66 x 10^-1^ |
| *COMTD1* | 14 | 2.41 x 10^-1^ | 8.95 x 10^-1^ |
| *COPB1* | 12 | 2.80 x 10^-1^ | 8.66 x 10^-1^ |
| *COPB2* | 23 | 4.39 x 10^-2^ | 9.89 x 10^-1^ |
| *COPE* | 14 | 1.04 x 10^-3^ | 1.12 x 10^-2^ |
| *COPG* | 13 | 2.06 x 10^-1^ | 8.49 x 10^-1^ |
| *COPS6* | 11 | 2.07 x 10^-1^ | 8.37 x 10^-1^ |
| *COPS7A* | 14 | 1.71 x 10^-1^ | 9.54 x 10^-1^ |
| *COPZ1* | 14 | 6.19 x 10^-2^ | 4.54 x 10^-1^ |
| *COPZ2* | 17 | 6.76 x 10^-2^ | 5.24 x 10^-1^ |
| *COQ10A* | 5 | 2.27 x 10^-1^ | 9.16 x 10^-1^ |
| *COQ10B* | 5 | 2.20 x 10^-1^ | 6.09 x 10^-1^ |
| *COQ5* | 15 | 2.06 x 10^-1^ | 3.43 x 10^-1^ |
| *COQ9* | 22 | 3.29 x 10^-2^ | 1.75 x 10^-1^ |
| *CORO1A* | 3 | 2.63 x 10^-1^ | 6.62 x 10^-1^ |
| *CORO1B* | 12 | 1.56 x 10^-2^ | 1.39 x 10^-1^ |
| *CORO1C* | 18 | 3.93 x 10^-2^ | 4.21 x 10^-1^ |
| *COTL1* | 86 | 6.75 x 10^-2^ | 9.65 x 10^-1^ |
| *COX10* | 52 | 1.56 x 10^-3^ | 8.10 x 10^-2^ |
| *COX4I1* | 20 | 6.70 x 10^-2^ | 6.14 x 10^-1^ |
| *COX4I2* | 11 | 5.33 x 10^-1^ | 9.70 x 10^-1^ |
| *COX4NB* | 24 | 6.70 x 10^-2^ | 6.12 x 10^-1^ |
| *COX5A* | 11 | 2.41 x 10^-1^ | 8.07 x 10^-1^ |
| *COX5B* | 6 | 7.67 x 10^-1^ | 8.45 x 10^-1^ |
| *COX6A1* | 8 | 9.48 x 10^-2^ | 4.00 x 10^-1^ |
| *COX6B1* | 14 | 2.76 x 10^-3^ | 1.12 x 10^-2^ |
| *COX6C* | 13 | 1.06 x 10^-1^ | 1.48 x 10^-1^ |
| *COX7A1* | 13 | 2.86 x 10^-1^ | 9.23 x 10^-1^ |
| *COX7A2* | 15 | 1.12 x 10^-1^ | 7.35 x 10^-1^ |
| *COX7A2L* | 12 | 4.49 x 10^-1^ | 9.10 x 10^-1^ |
| *COX7C* | 9 | 2.60 x 10^-1^ | 9.61 x 10^-1^ |
| *COX8A* | 10 | 4.23 x 10^-1^ | 7.88 x 10^-1^ |
| *CPA2* | 27 | 4.78 x 10^-2^ | 7.63 x 10^-1^ |
| *CPE* | 53 | 3.70 x 10^-2^ | 4.58 x 10^-1^ |
| *CPEB2* | 20 | 6.42 x 10^-2^ | 4.49 x 10^-1^ |
| *CPSF3L* | 2 | 2.59 x 10^-1^ | 1.66 x 10^-1^ |
| *CPSF4* | 6 | 7.30 x 10^-2^ | 1.61 x 10^-1^ |
| *CPT1A* | 23 | 4.73 x 10^-2^ | 7.25 x 10^-1^ |
| *CPT1C* | 13 | 5.83 x 10^-2^ | 3.40 x 10^-1^ |
| *CRADD* | 71 | 8.89 x 10^-3^ | 6.22 x 10^-1^ |
| *CRB3* | 16 | 1.16 x 10^-2^ | 4.88 x 10^-2^ |
| *CREB3* | 13 | 7.73 x 10^-2^ | 7.37 x 10^-1^ |
| *CREB3L1* | 15 | 3.52 x 10^-2^ | 1.68 x 10^-1^ |
| *CREB3L2* | 57 | 6.30 x 10^-2^ | 6.24 x 10^-1^ |
| *CREB3L4* | 7 | 1.40 x 10^-1^ | 2.77 x 10^-1^ |
| *CREBL2* | 26 | 5.48 x 10^-2^ | 9.87 x 10^-1^ |
| *CREG1* | 69 | 1.52 x 10^-2^ | 7.80 x 10^-1^ |
| *CRELD2* | 9 | 1.69 x 10^-2^ | 5.65 x 10^-2^ |
| *CRIP2* | 7 | 9.17 x 10^-2^ | 1.48 x 10^-1^ |
| *CRISPLD2* | 61 | 7.63 x 10^-2^ | 8.53 x 10^-1^ |
| *CRLF3* | 9 | 3.47 x 10^-2^ | 7.90 x 10^-2^ |
| *CRMP1* | 81 | 1.33 x 10^-2^ | 9.83 x 10^-1^ |
| *CRTAC1* | 55 | 1.10 x 10^-2^ | 2.04 x 10^-1^ |
| *CRTAP* | 29 | 1.37 x 10^-2^ | 3.96 x 10^-1^ |
| *CRTC2* | 5 | 2.84 x 10^-1^ | 5.90 x 10^-1^ |
| *CRYAB* | 5 | 2.75 x 10^-1^ | 3.70 x 10^-1^ |
| *CRYL1* | 67 | 7.93 x 10^-2^ | 9.19 x 10^-1^ |
| *CRYM* | 25 | 1.32 x 10^-2^ | 2.60 x 10^-1^ |
| *CS* | 7 | 2.27 x 10^-1^ | 9.16 x 10^-1^ |
| *CSDA* | 33 | 3.86 x 10^-3^ | 1.27 x 10^-1^ |
| *CSDE1* | 18 | 1.27 x 10^-1^ | 7.99 x 10^-1^ |
| *CSF1R* | 62 | 1.38 x 10^-2^ | 8.56 x 10^-1^ |
| *CSH1* | 11 | 6.50 x 10^-2^ | 1.64 x 10^-1^ |
| *CSH2* | 6 | 1.02 x 10^-1^ | 1.41 x 10^-1^ |
| *CSHL1* | 11 | 6.50 x 10^-2^ | 1.36 x 10^-1^ |
| *CSK* | 18 | 9.49 x 10^-2^ | 5.27 x 10^-1^ |
| *CSNK1A1* | 34 | 1.16 x 10^-1^ | 9.54 x 10^-1^ |
| *CSNK1D* | 12 | 1.76 x 10^-1^ | 7.22 x 10^-1^ |
| *CSNK1G2* | 23 | 4.56 x 10^-2^ | 5.84 x 10^-1^ |
| *CSNK2A2* | 30 | 9.17 x 10^-2^ | 7.28 x 10^-1^ |
| *CSNK2B* | 31 | 1.30 x 10^-2^ | 1.58 x 10^-1^ |
| *CSRP1* | 35 | 6.80 x 10^-2^ | 7.83 x 10^-1^ |
| *CSRP3* | 36 | 7.64 x 10^-2^ | 7.93 x 10^-1^ |
| *CST3* | 20 | 1.13 x 10^-2^ | 1.06 x 10^-1^ |
| *CST6* | 17 | 4.44 x 10^-2^ | 3.49 x 10^-1^ |
| *CST7* | 18 | 3.40 x 10^-1^ | 9.61 x 10^-1^ |
| *CSTA* | 22 | 1.38 x 10^-1^ | 9.18 x 10^-1^ |
| *CSTB* | 24 | 1.59 x 10^-2^ | 3.15 x 10^-1^ |
| *CTBP1* | 21 | 2.40 x 10^-1^ | 9.47 x 10^-1^ |
| *CTBP2* | 88 | 1.45 x 10^-3^ | 8.80 x 10^-2^ |
| *CTCF* | 7 | 2.83 x 10^-1^ | 1.00 |
| *CTDP1* | 29 | 2.65 x 10^-2^ | 3.32 x 10^-1^ |
| *CTDSP1* | 16 | 2.24 x 10^-2^ | 3.59 x 10^-1^ |
| *CTDSP2* | 9 | 1.47 x 10^-2^ | 4.61 x 10^-2^ |
| *CTGF* | 35 | 5.63 x 10^-3^ | 1.47 x 10^-1^ |
| *CTNNA1* | 20 | 2.80 x 10^-2^ | 7.04 x 10^-2^ |
| *CTNNB1* | 15 | 2.58 x 10^-2^ | 1.28 x 10^-1^ |
| *CTNNBIP1* | 17 | 1.50 x 10^-2^ | 1.36 x 10^-1^ |
| *CTNND1* | 8 | 2.94 x 10^-2^ | 8.99 x 10^-2^ |
| *CTNS* | 28 | 4.14 x 10^-2^ | 6.64 x 10^-1^ |
| *CTSA* | 23 | 1.40 x 10^-2^ | 1.15 x 10^-1^ |
| *CTSB* | 29 | 1.93 x 10^-2^ | 5.60 x 10^-1^ |
| *CTSC* | 39 | 4.65 x 10^-2^ | 6.26 x 10^-1^ |
| *CTSD* | 16 | 2.89 x 10^-2^ | 2.40 x 10^-1^ |
| *CTSF* | 14 | 5.53 x 10^-2^ | 1.16 x 10^-1^ |
| *CTSH* | 25 | 6.19 x 10^-3^ | 1.13 x 10^-1^ |
| *CTSK* | 6 | 1.65 x 10^-2^ | 4.76 x 10^-2^ |
| *CTSL1* | 32 | 1.42 x 10^-1^ | 8.05 x 10^-1^ |
| *CTSO* | 30 | 3.17 x 10^-3^ | 7.99 x 10^-2^ |
| *CTSZ* | 14 | 6.37 x 10^-2^ | 1.77 x 10^-1^ |
| *CUEDC2* | 15 | 1.30 x 10^-1^ | 7.17 x 10^-1^ |
| *CUGBP1* | 11 | 2.14 x 10^-1^ | 6.90 x 10^-1^ |
| *CUGBP2* | 163 | 2.18 x 10^-2^ | 9.61 x 10^-1^ |
| *CUTA* | 9 | 9.25 x 10^-4^ | 4.00 x 10^-3^ |
| *CUX1* | 84 | 1.05 x 10^-2^ | 5.53 x 10^-1^ |
| *CX3CL1* | 23 | 2.29 x 10^-2^ | 2.74 x 10^-1^ |
| *CXCL12* | 38 | 1.04 x 10^-1^ | 1.00 |
| *CXCL16* | 30 | 2.53 x 10^-2^ | 4.99 x 10^-1^ |
| *CXCL2* | 5 | 2.85 x 10^-1^ | 7.83 x 10^-1^ |
| *CXCR4* | 15 | 1.93 x 10^-2^ | 2.90 x 10^-1^ |
| *CXXC1* | 22 | 1.35 x 10^-1^ | 9.92 x 10^-1^ |
| *CYB561* | 12 | 1.54 x 10^-1^ | 9.62 x 10^-1^ |
| *CYB561D2* | 6 | 1.45 x 10^-1^ | 6.56 x 10^-1^ |
| *CYB5A* | 35 | 3.10 x 10^-2^ | 4.72 x 10^-1^ |
| *CYB5D2* | 19 | 8.61 x 10^-2^ | 6.70 x 10^-1^ |
| *CYB5R1* | 23 | 4.97 x 10^-2^ | 1.90 x 10^-1^ |
| *CYB5R3* | 36 | 3.74 x 10^-2^ | 3.80 x 10^-1^ |
| *CYBA* | 26 | 3.89 x 10^-2^ | 3.64 x 10^-1^ |
| *CYC1* | 4 | 3.53 x 10^-1^ | 9.07 x 10^-1^ |
| *CYFIP1* | 83 | 1.76 x 10^-3^ | 1.45 x 10^-1^ |
| *CYGB* | 18 | 2.03 x 10^-2^ | 3.65 x 10^-1^ |
| *CYP1A1* | 8 | 1.61 x 10^-1^ | 5.92 x 10^-1^ |
| *CYP1A2* | 13 | 1.61 x 10^-1^ | 5.71 x 10^-1^ |
| *CYP1B1* | 33 | 4.84 x 10^-2^ | 9.74 x 10^-1^ |
| *CYP27A1* | 11 | 8.44 x 10^-2^ | 1.66 x 10^-1^ |
| *CYP2A7* | 13 | 5.99 x 10^-2^ | 3.52 x 10^-1^ |
| *CYP2B6* | 25 | 1.09 x 10^-2^ | 2.39 x 10^-1^ |
| *CYP4B1* | 23 | 1.23 x 10^-1^ | 9.54 x 10^-1^ |
| *CYR61* | 31 | 1.17 x 10^-2^ | 1.40 x 10^-1^ |
| *DAB2* | 36 | 8.89 x 10^-3^ | 1.55 x 10^-1^ |
| *DAD1* | 62 | 1.44 x 10^-2^ | 8.63 x 10^-1^ |
| *DAG1* | 7 | 3.16 x 10^-1^ | 6.08 x 10^-1^ |
| *DAO* | 24 | 5.72 x 10^-2^ | 7.19 x 10^-1^ |
| *DAP* | 46 | 6.22 x 10^-2^ | 9.75 x 10^-1^ |
| *DAPK1* | 123 | 2.63 x 10^-2^ | 8.82 x 10^-1^ |
| *DAPP1* | 29 | 3.14 x 10^-3^ | 7.02 x 10^-2^ |
| *DARC* | 18 | 1.30 x 10^-2^ | 2.04 x 10^-1^ |
| *DAZAP2* | 13 | 3.85 x 10^-2^ | 5.01 x 10^-1^ |
| *DBI* | 19 | 7.58 x 10^-2^ | 1.00 |
| *DBNDD2* | 27 | 1.09 x 10^-2^ | 1.96 x 10^-1^ |
| *DBNL* | 9 | 1.27 x 10^-1^ | 2.77 x 10^-1^ |
| *DBT* | 15 | 1.12 x 10^-1^ | 8.67 x 10^-1^ |
| *DC2* | 12 | 1.21 x 10^-1^ | 4.49 x 10^-1^ |
| *DCI* | 15 | 6.04 x 10^-2^ | 4.72 x 10^-1^ |
| *DCN* | 17 | 1.08 x 10^-1^ | 4.83 x 10^-1^ |
| *DCP1A* | 18 | 1.80 x 10^-1^ | 8.95 x 10^-1^ |
| *DCTD* | 49 | 5.47 x 10^-2^ | 9.59 x 10^-1^ |
| *DCTN1* | 5 | 4.99 x 10^-1^ | 9.66 x 10^-1^ |
| *DCTN2* | 8 | 3.16 x 10^-1^ | 1.00 |
| *DCTN3* | 14 | 2.23 x 10^-1^ | 9.12 x 10^-1^ |
| *DCTN6* | 13 | 1.40 x 10^-2^ | 1.30 x 10^-1^ |
| *DCXR* | 10 | 9.84 x 10^-2^ | 4.30 x 10^-1^ |
| *DDA1* | 20 | 2.18 x 10^-1^ | 9.11 x 10^-1^ |
| *DDAH1* | 70 | 3.26 x 10^-2^ | 4.90 x 10^-1^ |
| *DDAH2* | 16 | 1.87 x 10^-2^ | 9.60 x 10^-2^ |
| *DDB1* | 3 | 3.75 x 10^-1^ | 7.57 x 10^-1^ |
| *DDIT4* | 18 | 1.45 x 10^-2^ | 2.19 x 10^-1^ |
| *DDOST* | 26 | 1.31 x 10^-1^ | 9.44 x 10^-1^ |
| *DDR1* | 31 | 3.50 x 10^-2^ | 8.48 x 10^-1^ |
| *DDT* | 9 | 5.55 x 10^-1^ | 9.22 x 10^-1^ |
| *DDX11* | 12 | 4.50 x 10^-2^ | 2.20 x 10^-1^ |
| *DDX17* | 21 | 7.16 x 10^-2^ | 7.99 x 10^-1^ |
| *DDX24* | 42 | 6.43 x 10^-3^ | 8.23 x 10^-2^ |
| *DDX27* | 16 | 2.10 x 10^-2^ | 1.53 x 10^-1^ |
| *DDX39* | 22 | 9.09 x 10^-3^ | 1.58 x 10^-1^ |
| *DDX41* | 2 | 4.73 x 10^-1^ | 6.63 x 10^-1^ |
| *DDX42* | 8 | 4.69 x 10^-2^ | 1.48 x 10^-1^ |
| *DDX47* | 29 | 1.80 x 10^-2^ | 4.14 x 10^-1^ |
| *DDX5* | 7 | 1.19 x 10^-1^ | 3.35 x 10^-1^ |
| *DDX56* | 11 | 1.25 x 10^-1^ | 9.09 x 10^-1^ |
| *DDX59* | 30 | 5.91 x 10^-2^ | 9.65 x 10^-1^ |
| *DECR1* | 16 | 2.36 x 10^-1^ | 3.96 x 10^-1^ |
| *DECR2* | 19 | 1.83 x 10^-1^ | 7.82 x 10^-1^ |
| *DEDD2* | 6 | 3.89 x 10^-1^ | 6.41 x 10^-1^ |
| *DENND3* | 54 | 3.66 x 10^-4^ | 1.45 x 10^-2^ |
| *DENND4B* | 6 | 2.67 x 10^-1^ | 5.12 x 10^-1^ |
| *DERL1* | 36 | 1.68 x 10^-1^ | 9.72 x 10^-1^ |
| *DEXI* | 23 | 1.60 x 10^-2^ | 1.78 x 10^-1^ |
| *DFFA* | 20 | 7.79 x 10^-3^ | 3.41 x 10^-2^ |
| *DFNA5* | 36 | 1.70 x 10^-2^ | 6.13 x 10^-1^ |
| *DGCR6* | 7 | 1.34 x 10^-1^ | 7.83 x 10^-1^ |
| *DGKZ* | 7 | 3.52 x 10^-2^ | 1.34 x 10^-1^ |
| *DGUOK* | 33 | 1.29 x 10^-1^ | 7.09 x 10^-1^ |
| *DHCR24* | 25 | 6.42 x 10^-2^ | 7.04 x 10^-1^ |
| *DHCR7* | 27 | 2.31 x 10^-2^ | 2.65 x 10^-1^ |
| *DHRS1* | 22 | 2.60 x 10^-2^ | 3.88 x 10^-1^ |
| *DHRS3* | 40 | 5.47 x 10^-3^ | 1.66 x 10^-1^ |
| *DHRS4* | 11 | 1.53 x 10^-1^ | 9.26 x 10^-1^ |
| *DHX15* | 27 | 4.93 x 10^-2^ | 6.81 x 10^-1^ |
| *DHX16* | 18 | 5.03 x 10^-3^ | 5.90 x 10^-2^ |
| *DHX30* | 11 | 2.93 x 10^-1^ | 8.21 x 10^-1^ |
| *DHX32* | 6 | 1.10 x 10^-2^ | 1.28 x 10^-2^ |
| *DHX38* | 17 | 8.00 x 10^-3^ | 1.04 x 10^-1^ |
| *DIABLO* | 11 | 4.16 x 10^-1^ | 9.74 x 10^-1^ |
| *DIAPH1* | 33 | 1.62 x 10^-1^ | 9.17 x 10^-1^ |
| *DIP2C* | 77 | 6.69 x 10^-2^ | 9.48 x 10^-1^ |
| *DKFZP434B0335* | 10 | 2.84 x 10^-2^ | 2.84 x 10^-1^ |
| *DKFZP564O0823* | 28 | 8.82 x 10^-2^ | 9.50 x 10^-1^ |
| *DKK3* | 54 | 3.72 x 10^-2^ | 7.92 x 10^-1^ |
| *DLC1* | 208 | 1.11 x 10^-2^ | 7.62 x 10^-1^ |
| *DLL1* | 23 | 1.08 x 10^-1^ | 9.72 x 10^-1^ |
| *DLST* | 16 | 2.98 x 10^-2^ | 2.76 x 10^-1^ |
| *DMWD* | 14 | 1.02 x 10^-1^ | 4.78 x 10^-1^ |
| *DNAJA1* | 34 | 1.31 x 10^-1^ | 7.69 x 10^-1^ |
| *DNAJA3* | 11 | 5.26 x 10^-1^ | 1.00 |
| *DNAJA4* | 43 | 2.72 x 10^-3^ | 9.50 x 10^-2^ |
| *DNAJB1* | 17 | 9.48 x 10^-2^ | 1.00 |
| *DNAJB2* | 17 | 3.49 x 10^-1^ | 9.07 x 10^-1^ |
| *DNAJC11* | 42 | 3.82 x 10^-2^ | 7.17 x 10^-1^ |
| *DNAJC5* | 20 | 4.72 x 10^-3^ | 4.52 x 10^-2^ |
| *DNASE2* | 11 | 1.04 x 10^-3^ | 6.47 x 10^-3^ |
| *DNM2* | 25 | 1.76 x 10^-2^ | 3.94 x 10^-1^ |
| *DNMT1* | 22 | 6.04 x 10^-3^ | 1.08 x 10^-1^ |
| *DNPEP* | 17 | 7.26 x 10^-3^ | 1.08 x 10^-1^ |
| *DOCK1* | 219 | 4.11 x 10^-3^ | 8.88 x 10^-1^ |
| *DOCK3* | 53 | 5.34 x 10^-2^ | 8.62 x 10^-1^ |
| *DOK2* | 16 | 1.61 x 10^-3^ | 1.19 x 10^-2^ |
| *DOLPP1* | 11 | 3.17 x 10^-1^ | 8.22 x 10^-1^ |
| *DPEP1* | 14 | 3.03 x 10^-2^ | 1.66 x 10^-1^ |
| *DPF2* | 10 | 7.05 x 10^-2^ | 3.73 x 10^-1^ |
| *DPM2* | 11 | 3.49 x 10^-2^ | 2.51 x 10^-1^ |
| *DPP7* | 6 | 1.53 x 10^-1^ | 6.38 x 10^-1^ |
| *DPYSL2* | 69 | 8.68 x 10^-3^ | 5.90 x 10^-1^ |
| *DPYSL3* | 28 | 1.77 x 10^-2^ | 4.26 x 10^-1^ |
| *DRAP1* | 9 | 7.46 x 10^-2^ | 7.80 x 10^-2^ |
| *DRD5* | 14 | 5.80 x 10^-2^ | 2.74 x 10^-1^ |
| *DRG1* | 12 | 1.02 x 10^-1^ | 4.74 x 10^-1^ |
| *DSP* | 48 | 1.27 x 10^-1^ | 9.78 x 10^-1^ |
| *DSTN* | 28 | 6.62 x 10^-2^ | 9.24 x 10^-1^ |
| *DUOX1* | 24 | 1.85 x 10^-4^ | 2.79 x 10^-3^ |
| *DUS1L* | 9 | 9.84 x 10^-2^ | 4.52 x 10^-1^ |
| *DUSP1* | 38 | 8.79 x 10^-2^ | 8.26 x 10^-1^ |
| *DUSP11* | 8 | 6.74 x 10^-1^ | 9.76 x 10^-1^ |
| *DUSP14* | 15 | 1.36 x 10^-1^ | 6.82 x 10^-1^ |
| *DUSP18* | 30 | 1.56 x 10^-2^ | 3.20 x 10^-1^ |
| *DUSP2* | 9 | 1.44 x 10^-2^ | 1.14 x 10^-1^ |
| *DUSP22* | 15 | 6.63 x 10^-3^ | 9.94 x 10^-2^ |
| *DUSP23* | 27 | 8.94 x 10^-3^ | 2.24 x 10^-1^ |
| *DUSP3* | 15 | 9.97 x 10^-2^ | 3.48 x 10^-1^ |
| *DUSP6* | 21 | 3.91 x 10^-2^ | 8.20 x 10^-1^ |
| *DUT* | 16 | 6.88 x 10^-3^ | 8.22 x 10^-2^ |
| *DYNC1H1* | 28 | 7.15 x 10^-2^ | 6.17 x 10^-1^ |
| *DYNC1I2* | 17 | 1.36 x 10^-1^ | 9.00 x 10^-1^ |
| *DYNC1LI2* | 6 | 2.87 x 10^-1^ | 7.00 x 10^-1^ |
| *DYNLL1* | 13 | 9.48 x 10^-2^ | 4.57 x 10^-1^ |
| *DYNLL2* | 20 | 2.23 x 10^-1^ | 1.00 |
| *DYNLRB1* | 4 | 3.70 x 10^-1^ | 8.41 x 10^-1^ |
| *DYNLT1* | 27 | 2.37 x 10^-1^ | 8.89 x 10^-1^ |
| *DYSF* | 98 | 5.69 x 10^-2^ | 9.64 x 10^-1^ |
| *E2F4* | 8 | 2.87 x 10^-1^ | 9.38 x 10^-1^ |
| *EBPL* | 17 | 2.01 x 10^-2^ | 2.89 x 10^-1^ |
| *ECH1* | 15 | 1.73 x 10^-1^ | 8.72 x 10^-1^ |
| *ECHDC2* | 9 | 1.58 x 10^-2^ | 1.26 x 10^-1^ |
| *ECHDC3* | 27 | 8.08 x 10^-2^ | 9.49 x 10^-1^ |
| *ECHS1* | 12 | 4.20 x 10^-1^ | 9.51 x 10^-1^ |
| *ECM1* | 13 | 1.71 x 10^-2^ | 2.23 x 10^-1^ |
| *EDAR* | 36 | 1.14 x 10^-1^ | 8.06 x 10^-1^ |
| *EDEM1* | 17 | 4.26 x 10^-2^ | 1.77 x 10^-1^ |
| *EDEM2* | 18 | 5.68 x 10^-2^ | 4.60 x 10^-1^ |
| *EDF1* | 7 | 3.48 x 10^-1^ | 5.34 x 10^-1^ |
| *EDN1* | 28 | 4.53 x 10^-3^ | 7.73 x 10^-2^ |
| *EDNRB* | 18 | 1.65 x 10^-1^ | 4.24 x 10^-1^ |
| *EEF1A1* | 13 | 3.12 x 10^-2^ | 4.05 x 10^-1^ |
| *EEF1B2* | 17 | 3.68 x 10^-2^ | 1.38 x 10^-1^ |
| *EEF1D* | 31 | 9.88 x 10^-2^ | 6.09 x 10^-1^ |
| *EEF1G* | 6 | 2.03 x 10^-1^ | 4.65 x 10^-1^ |
| *EEF2* | 18 | 1.99 x 10^-1^ | 1.00 |
| *EEFSEC* | 42 | 4.26 x 10^-2^ | 9.29 x 10^-1^ |
| *EFEMP1* | 34 | 6.03 x 10^-2^ | 7.66 x 10^-1^ |
| *EFEMP2* | 9 | 3.67 x 10^-2^ | 1.35 x 10^-1^ |
| *EFHD1* | 17 | 8.46 x 10^-2^ | 4.20 x 10^-1^ |
| *EFHD2* | 18 | 5.11 x 10^-2^ | 7.44 x 10^-1^ |
| *EFNA1* | 12 | 1.85 x 10^-1^ | 9.17 x 10^-1^ |
| *EFNB2* | 36 | 4.46 x 10^-2^ | 9.84 x 10^-1^ |
| *EGFL7* | 21 | 3.67 x 10^-2^ | 4.25 x 10^-1^ |
| *EGLN2* | 16 | 1.07 x 10^-1^ | 6.98 x 10^-1^ |
| *EGR1* | 13 | 1.18 x 10^-1^ | 1.87 x 10^-1^ |
| *EHD1* | 17 | 4.55 x 10^-2^ | 6.20 x 10^-1^ |
| *EHD2* | 26 | 1.93 x 10^-1^ | 9.40 x 10^-1^ |
| *EHD4* | 59 | 1.04 x 10^-2^ | 3.77 x 10^-1^ |
| *EI24* | 17 | 5.53 x 10^-1^ | 9.84 x 10^-1^ |
| *EIF1* | 12 | 3.57 x 10^-2^ | 1.94 x 10^-1^ |
| *EIF1B* | 9 | 2.34 x 10^-2^ | 8.06 x 10^-2^ |
| *EIF2AK1* | 21 | 5.28 x 10^-2^ | 8.93 x 10^-1^ |
| *EIF2B2* | 4 | 4.92 x 10^-1^ | 8.18 x 10^-1^ |
| *EIF2B3* | 24 | 5.64 x 10^-2^ | 2.24 x 10^-1^ |
| *EIF2B4* | 3 | 7.20 x 10^-2^ | 2.55 x 10^-2^ |
| *EIF2B5* | 21 | 3.54 x 10^-2^ | 1.90 x 10^-1^ |
| *EIF2C1* | 6 | 1.92 x 10^-2^ | 5.28 x 10^-3^ |
| *EIF2C2* | 41 | 3.10 x 10^-3^ | 5.95 x 10^-2^ |
| *EIF3B* | 14 | 1.48 x 10^-1^ | 9.74 x 10^-1^ |
| *EIF3C* | 11 | 1.66 x 10^-1^ | 3.96 x 10^-1^ |
| *EIF3D* | 37 | 1.71 x 10^-3^ | 6.13 x 10^-2^ |
| *EIF3EIP* | 9 | 1.32 x 10^-1^ | 7.77 x 10^-1^ |
| *EIF3F* | 43 | 2.61 x 10^-2^ | 5.62 x 10^-1^ |
| *EIF3G* | 16 | 6.04 x 10^-3^ | 8.50 x 10^-2^ |
| *EIF3H* | 34 | 7.51 x 10^-2^ | 5.11 x 10^-1^ |
| *EIF3I* | 9 | 2.90 x 10^-1^ | 9.49 x 10^-1^ |
| *EIF3K* | 17 | 2.32 x 10^-1^ | 9.44 x 10^-1^ |
| *EIF4A1* | 14 | 6.98 x 10^-2^ | 4.37 x 10^-1^ |
| *EIF4A2* | 23 | 1.05 x 10^-1^ | 9.55 x 10^-1^ |
| *EIF4A3* | 21 | 1.74 x 10^-1^ | 9.52 x 10^-1^ |
| *EIF4B* | 23 | 9.26 x 10^-2^ | 8.91 x 10^-1^ |
| *EIF4G1* | 20 | 2.42 x 10^-3^ | 3.53 x 10^-2^ |
| *EIF4G2* | 22 | 2.52 x 10^-2^ | 3.53 x 10^-1^ |
| *EIF4H* | 7 | 6.34 x 10^-2^ | 4.43 x 10^-1^ |
| *EIF5* | 19 | 2.12 x 10^-3^ | 4.03 x 10^-2^ |
| *EIF5A* | 23 | 4.84 x 10^-2^ | 5.97 x 10^-1^ |
| *EIF6* | 15 | 1.27 x 10^-1^ | 7.71 x 10^-1^ |
| *ELAVL1* | 24 | 2.33 x 10^-3^ | 5.22 x 10^-2^ |
| *ELF3* | 27 | 3.06 x 10^-3^ | 5.52 x 10^-2^ |
| *ELMO1* | 207 | 4.97 x 10^-3^ | 5.02 x 10^-1^ |
| *ELN* | 18 | 1.01 x 10^-1^ | 9.44 x 10^-1^ |
| *ELOF1* | 14 | 7.07 x 10^-2^ | 4.31 x 10^-1^ |
| *ELOVL1* | 5 | 2.83 x 10^-2^ | 6.12 x 10^-2^ |
| *EML3* | 6 | 2.03 x 10^-1^ | 3.69 x 10^-1^ |
| *EMP2* | 57 | 2.45 x 10^-2^ | 9.81 x 10^-1^ |
| *EMP3* | 15 | 1.98 x 10^-1^ | 6.22 x 10^-1^ |
| *ENG* | 22 | 3.49 x 10^-2^ | 6.77 x 10^-1^ |
| *ENO1* | 21 | 1.27 x 10^-1^ | 9.15 x 10^-1^ |
| *ENPP2* | 32 | 8.04 x 10^-2^ | 8.15 x 10^-1^ |
| *ENSA* | 7 | 2.38 x 10^-2^ | 1.42 x 10^-1^ |
| *ENTPD6* | 22 | 1.65 x 10^-2^ | 2.88 x 10^-1^ |
| *ENTPD8* | 1 | 1.39 x 10^-1^ | 3.86 x 10^-2^ |
| *EPAS1* | 83 | 2.29 x 10^-2^ | 6.72 x 10^-1^ |
| *EPHA2* | 22 | 9.75 x 10^-2^ | 8.17 x 10^-1^ |
| *EPHX1* | 23 | 4.30 x 10^-3^ | 4.21 x 10^-2^ |
| *EPOR* | 8 | 1.33 x 10^-1^ | 9.48 x 10^-1^ |
| *EPS8L2* | 20 | 2.33 x 10^-1^ | 9.94 x 10^-1^ |
| *EPSTI1* | 70 | 1.36 x 10^-2^ | 3.24 x 10^-1^ |
| *ERAL1* | 6 | 2.96 x 10^-1^ | 9.54 x 10^-1^ |
| *ERBB2* | 10 | 6.04 x 10^-2^ | 2.79 x 10^-1^ |
| *ERCC1* | 15 | 1.40 x 10^-1^ | 7.32 x 10^-1^ |
| *ERCC4* | 21 | 1.69 x 10^-1^ | 8.49 x 10^-1^ |
| *ERGIC3* | 14 | 2.92 x 10^-3^ | 3.90 x 10^-2^ |
| *ERH* | 18 | 3.11 x 10^-1^ | 9.68 x 10^-1^ |
| *ERP29* | 5 | 1.12 x 10^-1^ | 5.59 x 10^-1^ |
| *ESAM* | 21 | 1.26 x 10^-1^ | 9.78 x 10^-1^ |
| *ESD* | 31 | 6.14 x 10^-2^ | 8.00 x 10^-1^ |
| *ETFB* | 35 | 5.05 x 10^-2^ | 7.10 x 10^-1^ |
| *ETHE1* | 20 | 2.52 x 10^-1^ | 9.00 x 10^-1^ |
| *ETS2* | 34 | 1.16 x 10^-2^ | 2.47 x 10^-1^ |
| *ETV5* | 27 | 1.28 x 10^-1^ | 8.17 x 10^-1^ |
| *EVL* | 24 | 1.89 x 10^-2^ | 4.53 x 10^-1^ |
| *EWSR1* | 27 | 9.05 x 10^-2^ | 8.04 x 10^-1^ |
| *EXOSC7* | 18 | 1.67 x 10^-1^ | 8.92 x 10^-1^ |
| *EXPH5* | 31 | 3.58 x 10^-3^ | 7.63 x 10^-2^ |
| *EXT2* | 71 | 1.25 x 10^-2^ | 4.12 x 10^-1^ |
| *EZR* | 34 | 2.21 x 10^-2^ | 4.85 x 10^-1^ |
| *F13A1* | 132 | 4.44 x 10^-3^ | 2.88 x 10^-1^ |
| *FABP4* | 19 | 1.30 x 10^-1^ | 4.33 x 10^-1^ |
| *FABP5* | 18 | 1.55 x 10^-2^ | 2.14 x 10^-1^ |
| *FADD* | 26 | 9.43 x 10^-2^ | 9.34 x 10^-1^ |
| *FAM107A* | 29 | 1.45 x 10^-2^ | 4.21 x 10^-1^ |
| *FAM107B* | 153 | 4.44 x 10^-3^ | 6.09 x 10^-1^ |
| *FAM108A1* | 17 | 1.59 x 10^-1^ | 7.19 x 10^-1^ |
| *FAM115C* | 3 | 5.51 x 10^-1^ | 5.80 x 10^-1^ |
| *FAM120A* | 35 | 2.26 x 10^-2^ | 2.96 x 10^-1^ |
| *FAM128B* | 2 | 4.79 x 10^-2^ | 8.43 x 10^-3^ |
| *FAM129A* | 30 | 1.55 x 10^-2^ | 1.36 x 10^-1^ |
| *FAM129B* | 33 | 6.77 x 10^-2^ | 5.38 x 10^-1^ |
| *FAM131A* | 17 | 2.42 x 10^-3^ | 3.42 x 10^-2^ |
| *FAM134A* | 14 | 1.11 x 10^-1^ | 8.93 x 10^-1^ |
| *FAM14B* | 40 | 9.94 x 10^-5^ | 1.96 x 10^-3^ |
| *FAM152B* | 7 | 2.63 x 10^-1^ | 8.78 x 10^-1^ |
| *FAM160A2* | 40 | 4.92 x 10^-2^ | 9.38 x 10^-1^ |
| *FAM162A* | 23 | 1.33 x 10^-1^ | 5.59 x 10^-1^ |
| *FAM171A1* | 72 | 9.67 x 10^-4^ | 6.55 x 10^-2^ |
| *FAM173A* | 17 | 6.13 x 10^-2^ | 6.11 x 10^-1^ |
| *FAM174B* | 53 | 1.53 x 10^-1^ | 9.62 x 10^-1^ |
| *FAM176A* | 70 | 2.09 x 10^-2^ | 9.75 x 10^-1^ |
| *FAM22A* | 2 | 7.61 x 10^-1^ | 8.15 x 10^-1^ |
| *FAM32A* | 16 | 5.53 x 10^-2^ | 8.07 x 10^-1^ |
| *FAM3B* | 53 | 5.26 x 10^-3^ | 2.38 x 10^-1^ |
| *FAM40A* | 17 | 4.13 x 10^-1^ | 9.46 x 10^-1^ |
| *FAM46A* | 31 | 2.82 x 10^-2^ | 4.42 x 10^-1^ |
| *FAM46B* | 13 | 1.66 x 10^-1^ | 8.33 x 10^-1^ |
| *FAM50B* | 32 | 7.63 x 10^-3^ | 2.06 x 10^-1^ |
| *FAM53B* | 48 | 5.12 x 10^-2^ | 9.96 x 10^-1^ |
| *FAM53C* | 12 | 2.07 x 10^-1^ | 9.79 x 10^-1^ |
| *FAM57A* | 20 | 7.29 x 10^-2^ | 8.61 x 10^-1^ |
| *FAM60A* | 32 | 1.16 x 10^-1^ | 9.71 x 10^-1^ |
| *FAM62B* | 32 | 2.53 x 10^-2^ | 6.30 x 10^-1^ |
| *FAM63A* | 21 | 6.94 x 10^-3^ | 3.97 x 10^-2^ |
| *FAM65A* | 2 | 2.73 x 10^-1^ | 5.46 x 10^-1^ |
| *FAM71C* | 20 | 2.11 x 10^-1^ | 8.91 x 10^-1^ |
| *FAM80A* | 22 | 2.83 x 10^-3^ | 2.53 x 10^-2^ |
| *FAM82A2* | 7 | 7.35 x 10^-2^ | 1.45 x 10^-1^ |
| *FAM83A* | 30 | 1.14 x 10^-1^ | 1.00 |
| *FAM83D* | 17 | 3.45 x 10^-2^ | 3.86 x 10^-1^ |
| *FAM84A* | 12 | 2.40 x 10^-2^ | 1.97 x 10^-1^ |
| *FAM89A* | 25 | 4.36 x 10^-2^ | 4.20 x 10^-1^ |
| *FAM96A* | 12 | 2.07 x 10^-1^ | 7.28 x 10^-1^ |
| *FAM96B* | 7 | 1.69 x 10^-2^ | 1.12 x 10^-1^ |
| *FANCC* | 26 | 3.17 x 10^-3^ | 8.13 x 10^-2^ |
| *FARP1* | 152 | 3.71 x 10^-3^ | 4.10 x 10^-1^ |
| *FARSB* | 33 | 2.01 x 10^-2^ | 3.26 x 10^-1^ |
| *FASTK* | 16 | 7.50 x 10^-3^ | 5.08 x 10^-2^ |
| *FAT* | 58 | 2.42 x 10^-2^ | 8.48 x 10^-1^ |
| *FAU* | 15 | 1.46 x 10^-1^ | 1.00 |
| *FBL* | 23 | 6.06 x 10^-1^ | 9.99 x 10^-1^ |
| *FBLN1* | 78 | 2.06 x 10^-2^ | 8.94 x 10^-1^ |
| *FBLN5* | 55 | 1.12 x 10^-3^ | 4.57 x 10^-2^ |
| *FBP1* | 36 | 7.29 x 10^-3^ | 2.55 x 10^-1^ |
| *FBXL12* | 7 | 2.31 x 10^-1^ | 7.12 x 10^-1^ |
| *FBXL15* | 14 | 1.30 x 10^-1^ | 8.92 x 10^-1^ |
| *FBXO18* | 98 | 6.26 x 10^-2^ | 9.42 x 10^-1^ |
| *FBXO21* | 29 | 7.03 x 10^-2^ | 8.23 x 10^-1^ |
| *FBXO25* | 69 | 2.07 x 10^-4^ | 9.26 x 10^-3^ |
| *FBXO32* | 46 | 6.52 x 10^-2^ | 8.83 x 10^-1^ |
| *FBXO34* | 14 | 2.15 x 10^-1^ | 7.50 x 10^-1^ |
| *FBXO7* | 64 | 1.14 x 10^-3^ | 6.61 x 10^-2^ |
| *FBXO9* | 23 | 9.43 x 10^-2^ | 2.82 x 10^-1^ |
| *FBXW4* | 16 | 2.48 x 10^-2^ | 3.27 x 10^-1^ |
| *FBXW5* | 12 | 1.24 x 10^-1^ | 6.73 x 10^-1^ |
| *FBXW9* | 9 | 3.20 x 10^-2^ | 2.88 x 10^-1^ |
| *FCAMR* | 21 | 4.89 x 10^-3^ | 8.96 x 10^-2^ |
| *FCER1G* | 33 | 2.16 x 10^-2^ | 3.71 x 10^-1^ |
| *FCGR2A* | 9 | 3.85 x 10^-2^ | 1.35 x 10^-1^ |
| *FCGR3A* | 7 | 3.85 x 10^-2^ | 1.08 x 10^-1^ |
| *FCGRT* | 14 | 2.85 x 10^-2^ | 3.98 x 10^-1^ |
| *FCN1* | 39 | 1.77 x 10^-2^ | 6.71 x 10^-1^ |
| *FCN3* | 9 | 7.36 x 10^-3^ | 4.90 x 10^-2^ |
| *FCRLA* | 52 | 1.50 x 10^-2^ | 7.78 x 10^-1^ |
| *FDFT1* | 54 | 1.93 x 10^-2^ | 4.86 x 10^-1^ |
| *FDPS* | 4 | 2.40 x 10^-1^ | 3.08 x 10^-1^ |
| *FDX1* | 17 | 2.65 x 10^-1^ | 8.50 x 10^-1^ |
| *FEM1A* | 23 | 1.70 x 10^-1^ | 9.53 x 10^-1^ |
| *FERMT3* | 15 | 6.33 x 10^-2^ | 6.88 x 10^-1^ |
| *FEZ1* | 34 | 1.93 x 10^-1^ | 8.43 x 10^-1^ |
| *FEZ2* | 35 | 1.69 x 10^-2^ | 1.52 x 10^-1^ |
| *FGD3* | 36 | 6.87 x 10^-2^ | 9.77 x 10^-1^ |
| *FGF4* | 23 | 1.81 x 10^-1^ | 7.28 x 10^-1^ |
| *FGFR4* | 8 | 3.70 x 10^-1^ | 9.78 x 10^-1^ |
| *FGR* | 9 | 4.97 x 10^-2^ | 4.47 x 10^-1^ |
| *FHL2* | 48 | 2.96 x 10^-2^ | 3.76 x 10^-1^ |
| *FHOD1* | 13 | 2.87 x 10^-1^ | 1.00 |
| *FIBP* | 8 | 7.46 x 10^-2^ | 1.60 x 10^-1^ |
| *FICD* | 20 | 4.56 x 10^-2^ | 6.51 x 10^-1^ |
| *FIG4* | 31 | 6.74 x 10^-3^ | 8.78 x 10^-2^ |
| *FIS1* | 12 | 3.59 x 10^-2^ | 2.04 x 10^-1^ |
| *FKBP11* | 13 | 2.37 x 10^-1^ | 6.84 x 10^-1^ |
| *FKBP15* | 45 | 1.47 x 10^-2^ | 5.17 x 10^-1^ |
| *FKBP1A* | 50 | 5.41 x 10^-2^ | 7.26 x 10^-1^ |
| *FKBP2* | 12 | 6.33 x 10^-2^ | 6.91 x 10^-1^ |
| *FKBP4* | 19 | 2.53 x 10^-2^ | 3.96 x 10^-1^ |
| *FKBP9* | 20 | 1.88 x 10^-2^ | 8.03 x 10^-2^ |
| *FLII* | 13 | 3.94 x 10^-1^ | 9.29 x 10^-1^ |
| *FLJ10357* | 38 | 6.04 x 10^-3^ | 8.85 x 10^-2^ |
| *FLJ10404* | 2 | 4.92 x 10^-1^ | 6.63 x 10^-1^ |
| *FLJ10769* | 22 | 1.38 x 10^-2^ | 3.03 x 10^-1^ |
| *FLJ12529* | 5 | 1.85 x 10^-1^ | 5.83 x 10^-1^ |
| *FLJ14154* | 24 | 5.72 x 10^-2^ | 8.35 x 10^-1^ |
| *FLJ20309* | 25 | 4.12 x 10^-3^ | 9.36 x 10^-2^ |
| *FLJ21438* | 14 | 1.42 x 10^-1^ | 9.12 x 10^-1^ |
| *FLJ21865* | 35 | 9.63 x 10^-4^ | 3.37 x 10^-2^ |
| *FLJ22222* | 15 | 2.75 x 10^-1^ | 8.76 x 10^-1^ |
| *FLJ22662* | 23 | 4.25 x 10^-2^ | 9.77 x 10^-1^ |
| *FLJ40504* | 17 | 4.14 x 10^-2^ | 7.04 x 10^-1^ |
| *FLNC* | 19 | 3.94 x 10^-1^ | 9.71 x 10^-1^ |
| *FLOT1* | 27 | 2.38 x 10^-2^ | 1.76 x 10^-1^ |
| *FLOT2* | 7 | 2.96 x 10^-1^ | 9.54 x 10^-1^ |
| *FLVCR2* | 33 | 5.18 x 10^-2^ | 7.92 x 10^-1^ |
| *FMO2* | 38 | 1.15 x 10^-2^ | 2.09 x 10^-1^ |
| *FMOD* | 25 | 4.63 x 10^-2^ | 4.43 x 10^-1^ |
| *FN1* | 35 | 2.38 x 10^-3^ | 6.97 x 10^-2^ |
| *FN3KRP* | 14 | 7.95 x 10^-2^ | 6.06 x 10^-1^ |
| *FNBP1* | 40 | 2.40 x 10^-2^ | 9.59 x 10^-1^ |
| *FOLR1* | 13 | 1.90 x 10^-1^ | 8.24 x 10^-1^ |
| *FOLR2* | 11 | 2.63 x 10^-1^ | 9.08 x 10^-1^ |
| *FOS* | 30 | 6.98 x 10^-2^ | 7.50 x 10^-1^ |
| *FOXF1* | 44 | 4.08 x 10^-2^ | 6.63 x 10^-1^ |
| *FOXF2* | 35 | 3.03 x 10^-3^ | 8.58 x 10^-2^ |
| *FOXJ2* | 17 | 1.32 x 10^-1^ | 9.63 x 10^-1^ |
| *FOXK2* | 31 | 4.18 x 10^-2^ | 4.76 x 10^-1^ |
| *FOXO1* | 41 | 6.32 x 10^-3^ | 2.40 x 10^-1^ |
| *FOXO3* | 53 | 2.29 x 10^-2^ | 3.80 x 10^-1^ |
| *FPGS* | 17 | 1.17 x 10^-1^ | 9.47 x 10^-1^ |
| *FPR1* | 40 | 4.62 x 10^-2^ | 7.21 x 10^-1^ |
| *FRAG1* | 28 | 1.41 x 10^-2^ | 3.66 x 10^-1^ |
| *FRAT1* | 14 | 6.52 x 10^-2^ | 8.43 x 10^-1^ |
| *FRAT2* | 16 | 6.52 x 10^-2^ | 9.81 x 10^-1^ |
| *FRMD3* | 123 | 6.07 x 10^-2^ | 9.84 x 10^-1^ |
| *FRMD5* | 32 | 3.61 x 10^-3^ | 1.15 x 10^-1^ |
| *FST* | 17 | 1.35 x 10^-1^ | 8.69 x 10^-1^ |
| *FSTL1* | 38 | 1.41 x 10^-3^ | 4.13 x 10^-2^ |
| *FSTL3* | 20 | 2.25 x 10^-3^ | 4.50 x 10^-2^ |
| *FTH1* | 21 | 4.42 x 10^-2^ | 4.33 x 10^-1^ |
| *FTL* | 22 | 5.70 x 10^-3^ | 9.73 x 10^-2^ |
| *FTSJ3* | 8 | 4.69 x 10^-2^ | 1.36 x 10^-1^ |
| *FUCA1* | 4 | 2.59 x 10^-1^ | 5.76 x 10^-1^ |
| *FUCA2* | 33 | 2.04 x 10^-2^ | 4.53 x 10^-1^ |
| *FUS* | 3 | 8.40 x 10^-1^ | 9.27 x 10^-1^ |
| *FUZ* | 17 | 1.02 x 10^-2^ | 1.21 x 10^-1^ |
| *FXYD1* | 22 | 5.97 x 10^-2^ | 6.52 x 10^-1^ |
| *FXYD3* | 30 | 3.51 x 10^-2^ | 3.50 x 10^-1^ |
| *FXYD5* | 27 | 3.33 x 10^-2^ | 5.33 x 10^-1^ |
| *FXYD6* | 57 | 1.70 x 10^-2^ | 6.36 x 10^-1^ |
| *FYN* | 79 | 1.93 x 10^-2^ | 6.60 x 10^-1^ |
| *FZD4* | 29 | 2.73 x 10^-2^ | 4.63 x 10^-1^ |
| *G0S2* | 21 | 5.55 x 10^-2^ | 5.27 x 10^-1^ |
| *G6PC3* | 10 | 1.69 x 10^-1^ | 5.49 x 10^-1^ |
| *GAA* | 19 | 2.10 x 10^-1^ | 9.47 x 10^-1^ |
| *GAB2* | 39 | 2.33 x 10^-2^ | 3.60 x 10^-1^ |
| *GABARAP* | 16 | 1.41 x 10^-1^ | 7.75 x 10^-1^ |
| *GABARAPL1* | 31 | 1.22 x 10^-2^ | 3.77 x 10^-1^ |
| *GABARAPL2* | 16 | 7.68 x 10^-3^ | 1.91 x 10^-2^ |
| *GADD45B* | 23 | 6.48 x 10^-2^ | 6.23 x 10^-1^ |
| *GADD45G* | 30 | 1.06 x 10^-1^ | 8.76 x 10^-1^ |
| *GAK* | 37 | 1.53 x 10^-1^ | 9.78 x 10^-1^ |
| *GAL3ST3* | 16 | 4.44 x 10^-2^ | 6.47 x 10^-1^ |
| *GALM* | 26 | 8.87 x 10^-2^ | 5.19 x 10^-1^ |
| *GALNAC4S-6ST* | 13 | 2.88 x 10^-3^ | 8.44 x 10^-3^ |
| *GALNT11* | 20 | 2.18 x 10^-2^ | 3.70 x 10^-1^ |
| *GALNT2* | 93 | 1.40 x 10^-3^ | 7.82 x 10^-2^ |
| *GALNT9* | 22 | 4.62 x 10^-2^ | 1.61 x 10^-1^ |
| *GANAB* | 6 | 1.66 x 10^-1^ | 3.94 x 10^-1^ |
| *GAPDH* | 17 | 1.69 x 10^-1^ | 9.11 x 10^-1^ |
| *GARNL3* | 42 | 2.82 x 10^-2^ | 3.21 x 10^-1^ |
| *GARS* | 20 | 1.61 x 10^-2^ | 1.34 x 10^-1^ |
| *GAS2L1* | 15 | 1.18 x 10^-1^ | 9.65 x 10^-1^ |
| *GAS6* | 19 | 1.06 x 10^-2^ | 1.01 x 10^-1^ |
| *GAS7* | 168 | 1.52 x 10^-2^ | 7.17 x 10^-1^ |
| *GATA2* | 34 | 1.28 x 10^-1^ | 9.38 x 10^-1^ |
| *GATA6* | 21 | 2.96 x 10^-2^ | 6.22 x 10^-1^ |
| *GATAD2A* | 11 | 1.61 x 10^-3^ | 1.59 x 10^-2^ |
| *GATAD2B* | 19 | 1.05 x 10^-1^ | 6.86 x 10^-1^ |
| *GBF1* | 21 | 1.27 x 10^-2^ | 1.36 x 10^-1^ |
| *GCC1* | 17 | 1.10 x 10^-1^ | 8.90 x 10^-1^ |
| *GCHFR* | 12 | 7.35 x 10^-2^ | 3.57 x 10^-1^ |
| *GCS1* | 4 | 8.78 x 10^-1^ | 7.20 x 10^-1^ |
| *GDE1* | 22 | 9.53 x 10^-4^ | 4.79 x 10^-3^ |
| *GDF10* | 37 | 2.89 x 10^-2^ | 7.06 x 10^-1^ |
| *GDF15* | 17 | 7.56 x 10^-2^ | 3.92 x 10^-1^ |
| *GDI2* | 45 | 5.41 x 10^-2^ | 9.47 x 10^-1^ |
| *GEFT* | 10 | 4.54 x 10^-2^ | 3.82 x 10^-1^ |
| *GFAP* | 27 | 6.27 x 10^-3^ | 1.07 x 10^-1^ |
| *GGA1* | 27 | 1.14 x 10^-2^ | 1.94 x 10^-1^ |
| *GGA3* | 13 | 1.26 x 10^-1^ | 9.52 x 10^-1^ |
| *GGT1* | 12 | 4.58 x 10^-2^ | 5.80 x 10^-2^ |
| *GGTLC1* | 14 | 3.95 x 10^-1^ | 8.88 x 10^-1^ |
| *GHITM* | 21 | 3.27 x 10^-3^ | 6.87 x 10^-2^ |
| *GIMAP1* | 23 | 1.33 x 10^-1^ | 9.40 x 10^-1^ |
| *GIMAP5* | 28 | 1.33 x 10^-1^ | 9.24 x 10^-1^ |
| *GIPC1* | 24 | 9.09 x 10^-3^ | 2.18 x 10^-1^ |
| *GIT1* | 11 | 2.21 x 10^-1^ | 1.00 |
| *GIYD2* | 42 | 1.58 x 10^-1^ | 9.28 x 10^-1^ |
| *GJD2* | 37 | 1.36 x 10^-2^ | 2.90 x 10^-1^ |
| *GLB1* | 45 | 4.04 x 10^-2^ | 9.27 x 10^-1^ |
| *GLG1* | 22 | 1.96 x 10^-1^ | 9.05 x 10^-1^ |
| *GLO1* | 36 | 9.24 x 10^-3^ | 6.34 x 10^-2^ |
| *GLRX* | 46 | 3.00 x 10^-3^ | 8.20 x 10^-2^ |
| *GLRX2* | 2 | 5.83 x 10^-1^ | 6.42 x 10^-1^ |
| *GLRX5* | 43 | 3.14 x 10^-2^ | 6.91 x 10^-1^ |
| *GLT25D1* | 23 | 1.97 x 10^-2^ | 4.52 x 10^-1^ |
| *GLTSCR2* | 21 | 1.72 x 10^-1^ | 8.51 x 10^-1^ |
| *GLUD1* | 6 | 6.34 x 10^-1^ | 9.95 x 10^-1^ |
| *GLUL* | 34 | 1.80 x 10^-1^ | 9.77 x 10^-1^ |
| *GLYATL1* | 18 | 3.48 x 10^-2^ | 1.66 x 10^-1^ |
| *GM2A* | 36 | 2.59 x 10^-2^ | 7.26 x 10^-1^ |
| *GMEB2* | 21 | 1.71 x 10^-1^ | 9.55 x 10^-1^ |
| *GMFG* | 16 | 2.57 x 10^-1^ | 1.00 |
| *GMPPB* | 4 | 3.54 x 10^-1^ | 1.00 |
| *GMPR* | 32 | 1.03 x 10^-2^ | 3.29 x 10^-1^ |
| *GNA11* | 29 | 1.35 x 10^-2^ | 3.88 x 10^-1^ |
| *GNA15* | 29 | 1.35 x 10^-2^ | 3.65 x 10^-1^ |
| *GNAI2* | 9 | 1.18 x 10^-1^ | 5.43 x 10^-1^ |
| *GNAS* | 34 | 1.44 x 10^-1^ | 9.61 x 10^-1^ |
| *GNB1* | 9 | 4.27 x 10^-2^ | 3.46 x 10^-1^ |
| *GNB2* | 8 | 6.48 x 10^-3^ | 5.19 x 10^-2^ |
| *GNB2L1* | 15 | 2.62 x 10^-1^ | 9.25 x 10^-1^ |
| *GNG11* | 22 | 5.49 x 10^-3^ | 1.21 x 10^-1^ |
| *GNG3* | 6 | 1.66 x 10^-1^ | 7.76 x 10^-1^ |
| *GNG4* | 34 | 2.20 x 10^-2^ | 3.83 x 10^-1^ |
| *GNG5* | 17 | 2.64 x 10^-3^ | 4.48 x 10^-2^ |
| *GNLY* | 28 | 2.99 x 10^-2^ | 4.09 x 10^-1^ |
| *GNPTG* | 14 | 7.10 x 10^-3^ | 8.24 x 10^-2^ |
| *GNS* | 17 | 5.12 x 10^-2^ | 8.71 x 10^-1^ |
| *GOLGA3* | 29 | 2.00 x 10^-2^ | 5.26 x 10^-1^ |
| *GOLGA7* | 21 | 8.36 x 10^-3^ | 1.24 x 10^-1^ |
| *GOLPH3* | 24 | 1.07 x 10^-2^ | 1.50 x 10^-1^ |
| *GORASP2* | 12 | 1.74 x 10^-1^ | 7.88 x 10^-1^ |
| *GOSR1* | 13 | 9.13 x 10^-2^ | 7.63 x 10^-1^ |
| *GOT1L1* | 1 | 3.26 x 10^-1^ | 2.13 x 10^-1^ |
| *GOT2* | 22 | 6.87 x 10^-2^ | 9.42 x 10^-1^ |
| *GPAA1* | 4 | 3.53 x 10^-1^ | 9.07 x 10^-1^ |
| *GPATCH3* | 6 | 4.53 x 10^-1^ | 9.23 x 10^-1^ |
| *GPD1L* | 45 | 4.50 x 10^-3^ | 1.72 x 10^-1^ |
| *GPER* | 12 | 1.89 x 10^-1^ | 7.19 x 10^-1^ |
| *GPI* | 9 | 6.11 x 10^-3^ | 4.55 x 10^-2^ |
| *GPN1* | 13 | 6.90 x 10^-3^ | 1.66 x 10^-2^ |
| *GPN2* | 7 | 4.53 x 10^-1^ | 9.09 x 10^-1^ |
| *GPNMB* | 18 | 1.48 x 10^-1^ | 8.93 x 10^-1^ |
| *GPR108* | 28 | 2.48 x 10^-2^ | 6.96 x 10^-1^ |
| *GPR116* | 51 | 5.46 x 10^-3^ | 2.79 x 10^-1^ |
| *GPR152* | 11 | 1.56 x 10^-2^ | 1.11 x 10^-1^ |
| *GPR153* | 9 | 3.82 x 10^-2^ | 3.36 x 10^-1^ |
| *GPR172A* | 11 | 1.83 x 10^-1^ | 8.32 x 10^-1^ |
| *GPR175* | 10 | 1.22 x 10^-2^ | 2.10 x 10^-2^ |
| *GPR56* | 55 | 6.97 x 10^-2^ | 8.24 x 10^-1^ |
| *GPR61* | 16 | 6.76 x 10^-2^ | 4.44 x 10^-1^ |
| *GPRC5A* | 40 | 1.80 x 10^-2^ | 4.86 x 10^-1^ |
| *GPRC5D* | 36 | 3.29 x 10^-2^ | 7.90 x 10^-1^ |
| *GPRIN2* | 1 | 4.56 x 10^-2^ | 4.16 x 10^-3^ |
| *GPS1* | 8 | 9.84 x 10^-2^ | 4.38 x 10^-1^ |
| *GPS2* | 22 | 4.84 x 10^-2^ | 5.75 x 10^-1^ |
| *GPSM1* | 9 | 1.52 x 10^-2^ | 9.86 x 10^-2^ |
| *GPSM3* | 60 | 1.18 x 10^-2^ | 7.06 x 10^-1^ |
| *GPSN2* | 24 | 2.97 x 10^-2^ | 7.13 x 10^-1^ |
| *GPX1* | 5 | 4.03 x 10^-1^ | 8.98 x 10^-1^ |
| *GPX2* | 20 | 3.76 x 10^-3^ | 5.68 x 10^-2^ |
| *GPX3* | 47 | 1.55 x 10^-2^ | 6.81 x 10^-1^ |
| *GPX4* | 21 | 4.64 x 10^-2^ | 5.93 x 10^-1^ |
| *GRAMD4* | 46 | 9.95 x 10^-2^ | 9.85 x 10^-1^ |
| *GRASP* | 15 | 1.30 x 10^-1^ | 6.89 x 10^-1^ |
| *GRB2* | 17 | 1.26 x 10^-1^ | 8.99 x 10^-1^ |
| *GRB7* | 4 | 6.04 x 10^-2^ | 5.84 x 10^-2^ |
| *GRHPR* | 34 | 8.45 x 10^-2^ | 9.73 x 10^-1^ |
| *GRINA* | 6 | 3.12 x 10^-2^ | 7.91 x 10^-2^ |
| *GRIP2* | 55 | 9.76 x 10^-2^ | 9.45 x 10^-1^ |
| *GRK5* | 87 | 1.36 x 10^-3^ | 5.06 x 10^-2^ |
| *GRN* | 8 | 1.99 x 10^-1^ | 6.97 x 10^-1^ |
| *GSK3A* | 3 | 3.41 x 10^-1^ | 5.49 x 10^-1^ |
| *GSK3B* | 40 | 4.08 x 10^-2^ | 6.67 x 10^-1^ |
| *GSN* | 23 | 7.82 x 10^-2^ | 3.83 x 10^-1^ |
| *GSTA1* | 6 | 1.90 x 10^-2^ | 4.83 x 10^-2^ |
| *GSTA4* | 35 | 1.12 x 10^-1^ | 6.08 x 10^-1^ |
| *GSTK1* | 8 | 1.12 x 10^-1^ | 8.96 x 10^-1^ |
| *GSTM2* | 15 | 1.06 x 10^-1^ | 8.53 x 10^-1^ |
| *GSTM3* | 22 | 9.75 x 10^-2^ | 6.08 x 10^-1^ |
| *GSTM5* | 20 | 9.75 x 10^-2^ | 6.10 x 10^-1^ |
| *GSTO1* | 17 | 6.96 x 10^-2^ | 9.61 x 10^-1^ |
| *GSTP1* | 13 | 2.34 x 10^-1^ | 8.89 x 10^-1^ |
| *GSTT1* | 6 | 1.36 x 10^-2^ | 8.18 x 10^-2^ |
| *GTF2F1* | 13 | 4.58 x 10^-2^ | 5.95 x 10^-1^ |
| *GTF2I* | 7 | 3.17 x 10^-1^ | 9.56 x 10^-1^ |
| *GTF3A* | 29 | 2.28 x 10^-2^ | 1.90 x 10^-1^ |
| *GTF3C1* | 22 | 4.13 x 10^-2^ | 4.08 x 10^-1^ |
| *GTF3C2* | 3 | 7.20 x 10^-2^ | 2.05 x 10^-2^ |
| *GTF3C4* | 22 | 6.83 x 10^-2^ | 5.27 x 10^-1^ |
| *GTPBP2* | 6 | 1.90 x 10^-1^ | 5.46 x 10^-1^ |
| *GTSF1* | 20 | 8.28 x 10^-2^ | 7.03 x 10^-1^ |
| *GTSF1L* | 19 | 1.58 x 10^-1^ | 1.00 |
| *GUCY1A3* | 50 | 2.19 x 10^-2^ | 3.79 x 10^-1^ |
| *GUK1* | 9 | 4.79 x 10^-1^ | 9.48 x 10^-1^ |
| *GUSB* | 7 | 2.87 x 10^-1^ | 9.55 x 10^-1^ |
| *GYG1* | 26 | 7.70 x 10^-2^ | 4.83 x 10^-1^ |
| *GYPC* | 42 | 2.25 x 10^-1^ | 9.57 x 10^-1^ |
| *GYS1* | 24 | 5.70 x 10^-3^ | 1.14 x 10^-1^ |
| *GZMB* | 23 | 5.12 x 10^-2^ | 9.69 x 10^-1^ |
| *H1FX* | 12 | 2.06 x 10^-1^ | 7.88 x 10^-1^ |
| *H2AFV* | 7 | 7.22 x 10^-1^ | 9.50 x 10^-1^ |
| *H2AFY* | 38 | 6.65 x 10^-3^ | 2.06 x 10^-1^ |
| *H2AFZ* | 4 | 1.32 x 10^-1^ | 3.15 x 10^-1^ |
| *H3F3A* | 14 | 7.96 x 10^-2^ | 7.40 x 10^-1^ |
| *H3F3B* | 13 | 1.70 x 10^-1^ | 4.66 x 10^-1^ |
| *HADHA* | 15 | 4.69 x 10^-3^ | 2.14 x 10^-2^ |
| *HADHB* | 12 | 9.49 x 10^-3^ | 5.42 x 10^-2^ |
| *HAGH* | 25 | 3.62 x 10^-2^ | 2.06 x 10^-1^ |
| *HAPLN3* | 43 | 2.17 x 10^-1^ | 1.00 |
| *HAX1* | 15 | 2.24 x 10^-2^ | 1.60 x 10^-1^ |
| *HBA2* | 12 | 5.08 x 10^-2^ | 5.17 x 10^-1^ |
| *HBB* | 26 | 8.80 x 10^-2^ | 7.84 x 10^-1^ |
| *HBD* | 20 | 8.80 x 10^-2^ | 5.24 x 10^-1^ |
| *HBG1* | 21 | 8.80 x 10^-2^ | 6.97 x 10^-1^ |
| *HBG2* | 20 | 8.80 x 10^-2^ | 7.19 x 10^-1^ |
| *HBXIP* | 25 | 1.15 x 10^-1^ | 9.53 x 10^-1^ |
| *HCFC1R1* | 14 | 1.79 x 10^-1^ | 8.43 x 10^-1^ |
| *HCK* | 19 | 2.37 x 10^-1^ | 9.80 x 10^-1^ |
| *HDAC5* | 12 | 1.69 x 10^-1^ | 6.71 x 10^-1^ |
| *HDGF* | 17 | 2.20 x 10^-2^ | 3.25 x 10^-1^ |
| *HDGF2* | 21 | 2.04 x 10^-1^ | 9.89 x 10^-1^ |
| *HDLBP* | 37 | 1.81 x 10^-1^ | 9.99 x 10^-1^ |
| *HEATR2* | 7 | 5.56 x 10^-2^ | 3.89 x 10^-1^ |
| *HEBP1* | 36 | 3.29 x 10^-2^ | 6.44 x 10^-1^ |
| *HEBP2* | 21 | 1.83 x 10^-1^ | 9.63 x 10^-1^ |
| *HECW1* | 148 | 3.18 x 10^-2^ | 8.51 x 10^-1^ |
| *HEG1* | 68 | 2.54 x 10^-2^ | 8.68 x 10^-1^ |
| *HELZ* | 31 | 1.17 x 10^-2^ | 2.66 x 10^-1^ |
| *HEPACAM* | 28 | 1.28 x 10^-2^ | 2.90 x 10^-1^ |
| *HERPUD1* | 45 | 7.48 x 10^-2^ | 9.17 x 10^-1^ |
| *HES1* | 21 | 2.68 x 10^-2^ | 5.62 x 10^-1^ |
| *HEXA* | 15 | 2.10 x 10^-1^ | 7.94 x 10^-1^ |
| *HEXB* | 41 | 5.50 x 10^-3^ | 1.41 x 10^-1^ |
| *HEXIM1* | 23 | 2.81 x 10^-3^ | 4.53 x 10^-2^ |
| *HEY1* | 36 | 1.56 x 10^-2^ | 3.60 x 10^-1^ |
| *HGS* | 11 | 2.97 x 10^-2^ | 1.16 x 10^-1^ |
| *HHEX* | 13 | 2.84 x 10^-2^ | 3.69 x 10^-1^ |
| *HIATL1* | 14 | 2.46 x 10^-2^ | 3.44 x 10^-1^ |
| *HIG2* | 8 | 2.06 x 10^-1^ | 7.19 x 10^-1^ |
| *HIGD1B* | 15 | 6.43 x 10^-2^ | 3.26 x 10^-1^ |
| *HINT1* | 15 | 3.15 x 10^-1^ | 8.94 x 10^-1^ |
| *HINT2* | 21 | 1.99 x 10^-2^ | 1.45 x 10^-1^ |
| *HIPK1* | 33 | 5.07 x 10^-2^ | 7.96 x 10^-1^ |
| *HIRA* | 24 | 7.48 x 10^-2^ | 8.40 x 10^-1^ |
| *HIST1H1C* | 20 | 1.30 x 10^-1^ | 8.27 x 10^-1^ |
| *HIST1H2BD* | 34 | 1.31 x 10^-1^ | 9.47 x 10^-1^ |
| *HIST1H2BK* | 8 | 1.37 x 10^-1^ | 3.96 x 10^-1^ |
| *HIST1H3E* | 23 | 1.29 x 10^-1^ | 9.32 x 10^-1^ |
| *HIST1H4F* | 21 | 1.29 x 10^-1^ | 9.11 x 10^-1^ |
| *HIST2H2AA3* | 2 | 2.38 x 10^-1^ | 4.76 x 10^-1^ |
| *HIST2H2BE* | 8 | 2.27 x 10^-1^ | 6.33 x 10^-1^ |
| *HK1* | 78 | 4.14 x 10^-2^ | 8.82 x 10^-1^ |
| *HLA-A* | 35 | 1.77 x 10^-2^ | 2.45 x 10^-1^ |
| *HLA-B* | 55 | 3.28 x 10^-4^ | 1.25 x 10^-2^ |
| *HLA-C* | 77 | 1.29 x 10^-4^ | 5.75 x 10^-3^ |
| *HLA-DMA* | 45 | 4.85 x 10^-4^ | 1.85 x 10^-2^ |
| *HLA-DMB* | 43 | 4.85 x 10^-4^ | 1.90 x 10^-2^ |
| *HLA-DPA1* | 53 | 1.12 x 10^-3^ | 3.66 x 10^-2^ |
| *HLA-DPB1* | 56 | 1.12 x 10^-3^ | 3.96 x 10^-2^ |
| *HLA-DQA1* | 20 | 2.95 x 10^-1^ | 9.40 x 10^-1^ |
| *HLA-DQA2* | 106 | 5.39 x 10^-2^ | 9.15 x 10^-1^ |
| *HLA-DQB1* | 69 | 6.36 x 10^-2^ | 8.99 x 10^-1^ |
| *HLA-DRA* | 45 | 4.62 x 10^-2^ | 6.85 x 10^-1^ |
| *HLA-E* | 17 | 5.09 x 10^-3^ | 2.65 x 10^-2^ |
| *HLA-F* | 54 | 1.76 x 10^-2^ | 7.04 x 10^-1^ |
| *HLA-G* | 54 | 1.82 x 10^-2^ | 2.20 x 10^-1^ |
| *HLX* | 16 | 5.12 x 10^-2^ | 4.49 x 10^-1^ |
| *HM13* | 11 | 1.76 x 10^-2^ | 1.82 x 10^-1^ |
| *HMBOX1* | 30 | 1.28 x 10^-3^ | 2.40 x 10^-2^ |
| *HMBS* | 10 | 9.45 x 10^-2^ | 7.89 x 10^-1^ |
| *HMGB1* | 22 | 6.86 x 10^-2^ | 6.57 x 10^-1^ |
| *HMGCL* | 7 | 2.59 x 10^-1^ | 8.20 x 10^-1^ |
| *HMGN1* | 18 | 1.72 x 10^-2^ | 4.92 x 10^-2^ |
| *HMGN2* | 11 | 2.92 x 10^-1^ | 9.14 x 10^-1^ |
| *HMGN3* | 28 | 8.88 x 10^-2^ | 1.00 |
| *HMGN4* | 7 | 2.39 x 10^-1^ | 6.70 x 10^-1^ |
| *HMHA1* | 27 | 4.64 x 10^-2^ | 7.63 x 10^-1^ |
| *HMOX1* | 43 | 1.87 x 10^-2^ | 7.86 x 10^-1^ |
| *HNF1B* | 50 | 8.97 x 10^-4^ | 4.48 x 10^-2^ |
| *HNRNPA0* | 4 | 1.19 x 10^-1^ | 4.74 x 10^-1^ |
| *HNRNPA1* | 7 | 2.35 x 10^-1^ | 7.29 x 10^-1^ |
| *HNRNPA2B1* | 13 | 9.54 x 10^-2^ | 3.39 x 10^-1^ |
| *HNRNPAB* | 38 | 1.52 x 10^-2^ | 3.07 x 10^-1^ |
| *HNRNPC* | 32 | 3.49 x 10^-1^ | 9.95 x 10^-1^ |
| *HNRNPD* | 13 | 1.43 x 10^-2^ | 7.79 x 10^-2^ |
| *HNRNPH1* | 12 | 1.68 x 10^-1^ | 7.15 x 10^-1^ |
| *HNRNPK* | 12 | 2.85 x 10^-1^ | 9.66 x 10^-1^ |
| *HNRNPL* | 16 | 1.73 x 10^-1^ | 9.30 x 10^-1^ |
| *HNRNPM* | 22 | 3.45 x 10^-2^ | 4.30 x 10^-1^ |
| *HNRNPUL1* | 19 | 3.32 x 10^-5^ | 6.31 x 10^-4^ |
| *HOOK2* | 5 | 5.25 x 10^-3^ | 2.62 x 10^-2^ |
| *HOPX* | 24 | 5.07 x 10^-3^ | 1.08 x 10^-1^ |
| *HOXA5* | 25 | 1.10 x 10^-1^ | 9.72 x 10^-1^ |
| *HOXB2* | 19 | 7.16 x 10^-2^ | 9.22 x 10^-1^ |
| *HOXB5* | 16 | 3.84 x 10^-2^ | 6.15 x 10^-1^ |
| *HP* | 15 | 8.00 x 10^-3^ | 7.73 x 10^-2^ |
| *HPCAL1* | 71 | 3.23 x 10^-2^ | 7.10 x 10^-1^ |
| *HRASLS3* | 15 | 1.69 x 10^-2^ | 1.31 x 10^-1^ |
| *HS1BP3* | 51 | 7.59 x 10^-2^ | 6.79 x 10^-1^ |
| *HS3ST2* | 61 | 4.22 x 10^-3^ | 2.15 x 10^-1^ |
| *HSBP1* | 63 | 3.62 x 10^-2^ | 8.60 x 10^-1^ |
| *HSD17B11* | 26 | 3.37 x 10^-2^ | 8.29 x 10^-1^ |
| *HSD17B12* | 48 | 2.83 x 10^-3^ | 1.36 x 10^-1^ |
| *HSD17B14* | 27 | 5.58 x 10^-2^ | 6.61 x 10^-1^ |
| *HSD17B4* | 37 | 1.06 x 10^-1^ | 9.48 x 10^-1^ |
| *HSD17B6* | 13 | 2.58 x 10^-1^ | 9.38 x 10^-1^ |
| *HSD17B7* | 27 | 6.01 x 10^-3^ | 1.62 x 10^-1^ |
| *HSD17B8* | 26 | 1.91 x 10^-3^ | 2.12 x 10^-2^ |
| *HSP90AA1* | 12 | 7.15 x 10^-2^ | 1.73 x 10^-1^ |
| *HSP90AB1* | 19 | 2.00 x 10^-1^ | 9.26 x 10^-1^ |
| *HSPA1A* | 8 | 2.46 x 10^-2^ | 1.06 x 10^-1^ |
| *HSPA1B* | 13 | 2.46 x 10^-2^ | 2.52 x 10^-1^ |
| *HSPA2* | 12 | 3.28 x 10^-1^ | 1.00 |
| *HSPA5* | 10 | 5.72 x 10^-3^ | 2.85 x 10^-2^ |
| *HSPA6* | 9 | 3.85 x 10^-2^ | 1.35 x 10^-1^ |
| *HSPA8* | 22 | 1.73 x 10^-1^ | 9.48 x 10^-1^ |
| *HSPB1* | 9 | 3.72 x 10^-1^ | 8.83 x 10^-1^ |
| *HSPB6* | 21 | 6.59 x 10^-4^ | 1.08 x 10^-2^ |
| *HSPB7* | 22 | 1.27 x 10^-1^ | 6.48 x 10^-1^ |
| *HSPB8* | 40 | 2.28 x 10^-2^ | 2.42 x 10^-1^ |
| *HSPC152* | 11 | 1.32 x 10^-2^ | 8.72 x 10^-2^ |
| *HSPE1* | 6 | 2.20 x 10^-1^ | 6.15 x 10^-1^ |
| *HSPG2* | 40 | 1.18 x 10^-2^ | 4.73 x 10^-1^ |
| *HTR3D* | 23 | 1.15 x 10^-1^ | 9.67 x 10^-1^ |
| *HTRA1* | 27 | 7.07 x 10^-2^ | 7.75 x 10^-1^ |
| *HVCN1* | 8 | 4.43 x 10^-1^ | 9.63 x 10^-1^ |
| *HYAL1* | 7 | 1.18 x 10^-1^ | 3.92 x 10^-1^ |
| *HYAL2* | 5 | 1.18 x 10^-1^ | 2.55 x 10^-1^ |
| *HYI* | 13 | 2.53 x 10^-2^ | 2.06 x 10^-1^ |
| *HYLS1* | 22 | 1.66 x 10^-1^ | 9.92 x 10^-1^ |
| *HYOU1* | 16 | 9.45 x 10^-2^ | 9.27 x 10^-1^ |
| *HYPK* | 6 | 8.27 x 10^-2^ | 4.96 x 10^-1^ |
| *ICAM1* | 28 | 1.52 x 10^-2^ | 4.24 x 10^-1^ |
| *ICAM2* | 21 | 7.30 x 10^-3^ | 9.37 x 10^-2^ |
| *ICAM3* | 29 | 7.63 x 10^-2^ | 8.10 x 10^-1^ |
| *ICMT* | 10 | 3.82 x 10^-2^ | 3.60 x 10^-1^ |
| *ID1* | 10 | 2.61 x 10^-1^ | 9.78 x 10^-1^ |
| *ID2* | 15 | 1.03 x 10^-1^ | 7.62 x 10^-1^ |
| *ID3* | 16 | 1.04 x 10^-1^ | 4.09 x 10^-1^ |
| *IDH1* | 16 | 8.73 x 10^-2^ | 7.53 x 10^-1^ |
| *IDH2* | 21 | 5.07 x 10^-2^ | 9.52 x 10^-1^ |
| *IDH3B* | 33 | 6.49 x 10^-2^ | 9.31 x 10^-1^ |
| *IER2* | 15 | 3.59 x 10^-2^ | 5.38 x 10^-1^ |
| *IER3* | 23 | 2.38 x 10^-2^ | 1.82 x 10^-1^ |
| *IER5* | 19 | 5.24 x 10^-1^ | 1.00 |
| *IFFO* | 18 | 1.69 x 10^-1^ | 9.11 x 10^-1^ |
| *IFI27* | 33 | 9.94 x 10^-5^ | 1.77 x 10^-3^ |
| *IFI30* | 18 | 2.08 x 10^-1^ | 8.60 x 10^-1^ |
| *IFI35* | 8 | 9.89 x 10^-3^ | 1.60 x 10^-2^ |
| *IFI6* | 9 | 4.97 x 10^-2^ | 4.47 x 10^-1^ |
| *IFITM1* | 25 | 1.11 x 10^-1^ | 8.72 x 10^-1^ |
| *IFITM2* | 24 | 1.11 x 10^-1^ | 8.79 x 10^-1^ |
| *IFITM3* | 24 | 1.11 x 10^-1^ | 8.35 x 10^-1^ |
| *IFNA17* | 10 | 4.80 x 10^-1^ | 1.00 |
| *IFNGR1* | 26 | 1.25 x 10^-2^ | 1.30 x 10^-1^ |
| *IFNGR2* | 22 | 9.13 x 10^-2^ | 6.17 x 10^-1^ |
| *IFRD2* | 7 | 1.18 x 10^-1^ | 3.92 x 10^-1^ |
| *IFT52* | 21 | 2.55 x 10^-1^ | 9.32 x 10^-1^ |
| *IGF1R* | 116 | 1.78 x 10^-3^ | 1.97 x 10^-1^ |
| *IGF2R* | 57 | 1.46 x 10^-2^ | 6.12 x 10^-1^ |
| *IGFBP2* | 28 | 2.57 x 10^-2^ | 6.30 x 10^-1^ |
| *IGFBP3* | 24 | 7.48 x 10^-2^ | 8.77 x 10^-1^ |
| *IGFBP4* | 15 | 6.81 x 10^-2^ | 2.91 x 10^-1^ |
| *IGFBP6* | 15 | 9.26 x 10^-2^ | 7.56 x 10^-1^ |
| *IGFBP7* | 56 | 5.20 x 10^-2^ | 9.49 x 10^-1^ |
| *IGHMBP2* | 22 | 4.46 x 10^-2^ | 1.73 x 10^-1^ |
| *IGLL1* | 34 | 1.44 x 10^-2^ | 4.90 x 10^-1^ |
| *IGSF21* | 132 | 6.13 x 10^-3^ | 4.92 x 10^-1^ |
| *IGSF3* | 12 | 2.26 x 10^-1^ | 7.75 x 10^-1^ |
| *IGSF8* | 29 | 4.58 x 10^-2^ | 8.59 x 10^-1^ |
| *IHPK2* | 8 | 7.42 x 10^-2^ | 3.96 x 10^-1^ |
| *IKBKE* | 31 | 1.85 x 10^-1^ | 1.00 |
| *IL10RA* | 29 | 1.52 x 10^-2^ | 2.45 x 10^-1^ |
| *IL10RB* | 31 | 1.17 x 10^-1^ | 9.32 x 10^-1^ |
| *IL15RA* | 78 | 6.26 x 10^-2^ | 8.69 x 10^-1^ |
| *IL17RA* | 35 | 6.95 x 10^-2^ | 9.62 x 10^-1^ |
| *IL17RD* | 35 | 2.91 x 10^-2^ | 7.33 x 10^-1^ |
| *IL1R1* | 36 | 1.97 x 10^-2^ | 2.64 x 10^-1^ |
| *IL20RB* | 19 | 1.57 x 10^-2^ | 2.82 x 10^-1^ |
| *IL2RB* | 57 | 6.15 x 10^-3^ | 3.37 x 10^-1^ |
| *IL32* | 17 | 1.79 x 10^-1^ | 8.44 x 10^-1^ |
| *IL34* | 23 | 2.54 x 10^-2^ | 2.69 x 10^-1^ |
| *IL4R* | 41 | 1.56 x 10^-2^ | 4.36 x 10^-1^ |
| *IL6* | 40 | 1.93 x 10^-2^ | 4.95 x 10^-1^ |
| *IL7R* | 27 | 1.00 x 10^-1^ | 6.66 x 10^-1^ |
| *ILK* | 23 | 1.08 x 10^-2^ | 2.41 x 10^-1^ |
| *ILKAP* | 24 | 1.39 x 10^-1^ | 8.90 x 10^-1^ |
| *ILVBL* | 23 | 1.35 x 10^-3^ | 1.47 x 10^-2^ |
| *IMMT* | 30 | 2.28 x 10^-2^ | 9.89 x 10^-2^ |
| *IMP3* | 11 | 1.11 x 10^-1^ | 4.81 x 10^-1^ |
| *IMP4* | 11 | 1.08 x 10^-2^ | 1.19 x 10^-1^ |
| *IMPA2* | 36 | 7.63 x 10^-2^ | 9.40 x 10^-1^ |
| *IMPDH1* | 15 | 1.34 x 10^-1^ | 8.21 x 10^-1^ |
| *IMPDH2* | 4 | 2.39 x 10^-1^ | 2.28 x 10^-1^ |
| *INDO* | 13 | 1.75 x 10^-1^ | 9.25 x 10^-1^ |
| *INHBB* | 15 | 6.61 x 10^-2^ | 9.91 x 10^-1^ |
| *INPP1* | 25 | 1.03 x 10^-1^ | 8.20 x 10^-1^ |
| *INPP5A* | 45 | 3.89 x 10^-2^ | 4.98 x 10^-1^ |
| *INPP5D* | 72 | 7.21 x 10^-2^ | 9.77 x 10^-1^ |
| *INPP5E* | 16 | 1.18 x 10^-2^ | 7.54 x 10^-2^ |
| *INPPL1* | 13 | 2.63 x 10^-1^ | 9.20 x 10^-1^ |
| *INSIG1* | 11 | 4.22 x 10^-1^ | 7.82 x 10^-1^ |
| *INTS10* | 45 | 4.59 x 10^-3^ | 2.07 x 10^-1^ |
| *IPO13* | 17 | 1.03 x 10^-2^ | 1.54 x 10^-1^ |
| *IRF1* | 22 | 1.15 x 10^-1^ | 9.62 x 10^-1^ |
| *IRF2* | 77 | 1.86 x 10^-2^ | 6.47 x 10^-1^ |
| *IRF2BP1* | 20 | 2.26 x 10^-2^ | 1.95 x 10^-1^ |
| *IRF3* | 11 | 5.83 x 10^-2^ | 5.25 x 10^-1^ |
| *IRF5* | 14 | 6.99 x 10^-2^ | 2.02 x 10^-1^ |
| *IRF6* | 27 | 3.52 x 10^-2^ | 3.24 x 10^-1^ |
| *IRF7* | 12 | 6.75 x 10^-2^ | 4.32 x 10^-1^ |
| *IRF8* | 54 | 3.24 x 10^-2^ | 9.28 x 10^-1^ |
| *IRF9* | 22 | 1.06 x 10^-1^ | 6.69 x 10^-1^ |
| *IRS2* | 35 | 3.85 x 10^-2^ | 1.00 |
| *IRX3* | 36 | 3.42 x 10^-2^ | 7.85 x 10^-1^ |
| *IRX5* | 13 | 4.36 x 10^-1^ | 9.19 x 10^-1^ |
| *ISCU* | 27 | 7.45 x 10^-3^ | 5.58 x 10^-2^ |
| *ISG20* | 20 | 9.80 x 10^-2^ | 8.83 x 10^-1^ |
| *ISLR* | 19 | 1.10 x 10^-2^ | 1.87 x 10^-1^ |
| *ISOC1* | 23 | 5.36 x 10^-2^ | 5.78 x 10^-1^ |
| *ISOC2* | 14 | 4.80 x 10^-2^ | 5.28 x 10^-1^ |
| *ISYNA1* | 10 | 7.80 x 10^-2^ | 3.09 x 10^-1^ |
| *ITFG3* | 25 | 4.60 x 10^-2^ | 6.19 x 10^-1^ |
| *ITGA10* | 12 | 2.75 x 10^-1^ | 7.58 x 10^-1^ |
| *ITGA3* | 21 | 1.03 x 10^-1^ | 6.62 x 10^-1^ |
| *ITGA5* | 11 | 6.19 x 10^-2^ | 1.45 x 10^-1^ |
| *ITGAE* | 32 | 1.16 x 10^-1^ | 7.40 x 10^-1^ |
| *ITGAM* | 21 | 2.11 x 10^-1^ | 9.46 x 10^-1^ |
| *ITGB2* | 44 | 4.00 x 10^-3^ | 1.40 x 10^-1^ |
| *ITGB5* | 63 | 1.10 x 10^-3^ | 5.70 x 10^-2^ |
| *ITLN1* | 15 | 1.01 x 10^-2^ | 8.99 x 10^-2^ |
| *ITM2B* | 9 | 1.67 x 10^-1^ | 4.68 x 10^-1^ |
| *ITM2C* | 37 | 8.73 x 10^-2^ | 9.46 x 10^-1^ |
| *ITPA* | 31 | 8.56 x 10^-4^ | 2.32 x 10^-2^ |
| *ITPK1* | 60 | 4.36 x 10^-2^ | 8.79 x 10^-1^ |
| *ITPKC* | 22 | 5.85 x 10^-2^ | 7.28 x 10^-1^ |
| *ITPR3* | 61 | 2.13 x 10^-4^ | 8.73 x 10^-3^ |
| *JAG2* | 18 | 1.79 x 10^-2^ | 1.59 x 10^-1^ |
| *JAGN1* | 14 | 2.85 x 10^-1^ | 1.00 |
| *JAK1* | 41 | 1.51 x 10^-2^ | 4.67 x 10^-1^ |
| *JAM2* | 30 | 7.43 x 10^-2^ | 7.86 x 10^-1^ |
| *JAM3* | 40 | 5.53 x 10^-2^ | 8.57 x 10^-1^ |
| *JARID2* | 87 | 1.46 x 10^-2^ | 3.83 x 10^-1^ |
| *JMJD1B* | 22 | 1.18 x 10^-1^ | 6.03 x 10^-1^ |
| *JMJD3* | 23 | 6.16 x 10^-2^ | 5.11 x 10^-1^ |
| *JOSD1* | 9 | 1.78 x 10^-2^ | 1.48 x 10^-1^ |
| *JTB* | 7 | 1.40 x 10^-1^ | 2.77 x 10^-1^ |
| *JTV1* | 17 | 5.28 x 10^-2^ | 8.88 x 10^-1^ |
| *JUN* | 14 | 1.22 x 10^-1^ | 5.05 x 10^-1^ |
| *JUNB* | 5 | 5.25 x 10^-3^ | 2.58 x 10^-2^ |
| *JUND* | 26 | 1.21 x 10^-2^ | 3.02 x 10^-1^ |
| *JUP* | 23 | 1.04 x 10^-2^ | 2.01 x 10^-1^ |
| *KANK1* | 176 | 4.14 x 10^-2^ | 9.66 x 10^-1^ |
| *KANK2* | 38 | 3.81 x 10^-3^ | 8.72 x 10^-2^ |
| *KANK3* | 16 | 2.78 x 10^-2^ | 1.34 x 10^-1^ |
| *KARS* | 12 | 7.68 x 10^-3^ | 6.26 x 10^-2^ |
| *KAT2A* | 12 | 2.26 x 10^-2^ | 2.05 x 10^-1^ |
| *KAT5* | 8 | 7.52 x 10^-2^ | 6.02 x 10^-1^ |
| *KATNB1* | 38 | 1.73 x 10^-3^ | 6.00 x 10^-2^ |
| *KBTBD2* | 16 | 1.88 x 10^-2^ | 6.13 x 10^-2^ |
| *KCMF1* | 24 | 3.72 x 10^-2^ | 2.50 x 10^-1^ |
| *KCNH3* | 12 | 1.03 x 10^-1^ | 6.54 x 10^-1^ |
| *KCNK17* | 50 | 8.69 x 10^-3^ | 3.24 x 10^-1^ |
| *KCNK3* | 18 | 3.15 x 10^-2^ | 3.86 x 10^-1^ |
| *KCNK4* | 13 | 1.32 x 10^-2^ | 9.29 x 10^-2^ |
| *KCNMB1* | 50 | 8.57 x 10^-4^ | 4.06 x 10^-2^ |
| *KCNMB4* | 57 | 1.18 x 10^-2^ | 6.64 x 10^-1^ |
| *KCNS1* | 20 | 1.45 x 10^-4^ | 7.45 x 10^-4^ |
| *KCNS3* | 46 | 4.43 x 10^-2^ | 6.18 x 10^-1^ |
| *KCTD10* | 21 | 1.67 x 10^-2^ | 3.29 x 10^-1^ |
| *KCTD14* | 21 | 6.42 x 10^-2^ | 7.67 x 10^-1^ |
| *KCTD2* | 21 | 1.14 x 10^-1^ | 9.67 x 10^-1^ |
| *KCTD20* | 16 | 2.66 x 10^-1^ | 7.49 x 10^-1^ |
| *KCTD5* | 12 | 6.92 x 10^-3^ | 2.31 x 10^-2^ |
| *KDELR1* | 12 | 1.98 x 10^-1^ | 8.37 x 10^-1^ |
| *KDELR2* | 20 | 2.45 x 10^-2^ | 1.52 x 10^-1^ |
| *KEAP1* | 13 | 8.17 x 10^-3^ | 1.04 x 10^-1^ |
| *KHDRBS1* | 9 | 6.43 x 10^-2^ | 5.79 x 10^-1^ |
| *KHSRP* | 16 | 1.41 x 10^-2^ | 2.01 x 10^-1^ |
| *KIAA0141* | 28 | 3.10 x 10^-2^ | 4.04 x 10^-1^ |
| *KIAA0146* | 19 | 3.77 x 10^-2^ | 3.39 x 10^-1^ |
| *KIAA0152* | 21 | 9.63 x 10^-2^ | 9.30 x 10^-1^ |
| *KIAA0174* | 13 | 8.34 x 10^-2^ | 5.02 x 10^-1^ |
| *KIAA0182* | 14 | 3.68 x 10^-3^ | 3.45 x 10^-2^ |
| *KIAA0247* | 20 | 2.90 x 10^-1^ | 9.68 x 10^-1^ |
| *KIAA0284* | 14 | 2.99 x 10^-2^ | 3.97 x 10^-1^ |
| *KIAA0355* | 14 | 7.23 x 10^-3^ | 2.61 x 10^-2^ |
| *KIAA0494* | 20 | 1.23 x 10^-1^ | 6.99 x 10^-1^ |
| *KIAA0556* | 37 | 7.79 x 10^-2^ | 5.49 x 10^-1^ |
| *KIAA0652* | 11 | 1.32 x 10^-2^ | 3.95 x 10^-2^ |
| *KIAA0664* | 13 | 1.75 x 10^-1^ | 5.75 x 10^-1^ |
| *KIAA0746* | 52 | 1.49 x 10^-2^ | 6.10 x 10^-1^ |
| *KIAA0859* | 27 | 2.30 x 10^-2^ | 2.40 x 10^-1^ |
| *KIAA0913* | 10 | 4.73 x 10^-1^ | 9.61 x 10^-1^ |
| *KIAA1147* | 10 | 7.57 x 10^-3^ | 7.57 x 10^-2^ |
| *KIAA1191* | 16 | 3.35 x 10^-3^ | 3.65 x 10^-2^ |
| *KIAA1429* | 20 | 2.46 x 10^-1^ | 9.50 x 10^-1^ |
| *KIAA1553* | 23 | 7.90 x 10^-2^ | 9.18 x 10^-1^ |
| *KIAA1754* | 20 | 6.96 x 10^-2^ | 9.52 x 10^-1^ |
| *KIAA1754L* | 10 | 3.60 x 10^-1^ | 7.42 x 10^-1^ |
| *KIAA1949* | 19 | 2.38 x 10^-2^ | 1.43 x 10^-1^ |
| *KIAA2013* | 17 | 1.63 x 10^-1^ | 8.56 x 10^-1^ |
| *KIF13B* | 58 | 1.28 x 10^-3^ | 5.18 x 10^-2^ |
| *KIR3DX1* | 30 | 1.22 x 10^-3^ | 3.49 x 10^-2^ |
| *KLC1* | 17 | 2.74 x 10^-2^ | 1.32 x 10^-1^ |
| *KLC2* | 12 | 1.90 x 10^-1^ | 5.64 x 10^-1^ |
| *KLC4* | 9 | 1.07 x 10^-1^ | 9.32 x 10^-1^ |
| *KLF10* | 29 | 7.94 x 10^-2^ | 9.39 x 10^-1^ |
| *KLF11* | 20 | 6.82 x 10^-3^ | 1.02 x 10^-1^ |
| *KLF15* | 30 | 5.20 x 10^-3^ | 1.07 x 10^-1^ |
| *KLF2* | 15 | 1.13 x 10^-1^ | 8.28 x 10^-1^ |
| *KLF4* | 15 | 2.94 x 10^-1^ | 9.81 x 10^-1^ |
| *KLF6* | 54 | 4.86 x 10^-2^ | 7.90 x 10^-1^ |
| *KLF7* | 46 | 2.84 x 10^-2^ | 7.20 x 10^-1^ |
| *KLHDC2* | 5 | 1.29 x 10^-1^ | 4.49 x 10^-1^ |
| *KLHDC3* | 11 | 1.37 x 10^-1^ | 9.62 x 10^-1^ |
| *KLHDC8B* | 8 | 3.48 x 10^-1^ | 8.75 x 10^-1^ |
| *KLK11* | 51 | 1.89 x 10^-2^ | 3.52 x 10^-1^ |
| *KLK3* | 34 | 9.48 x 10^-2^ | 6.49 x 10^-1^ |
| *KNCN* | 23 | 4.30 x 10^-2^ | 3.12 x 10^-1^ |
| *KRT10* | 29 | 1.38 x 10^-2^ | 3.32 x 10^-1^ |
| *KRT18* | 15 | 2.03 x 10^-1^ | 8.55 x 10^-1^ |
| *KRT19* | 27 | 2.72 x 10^-2^ | 5.03 x 10^-1^ |
| *KRT2* | 40 | 2.74 x 10^-2^ | 4.54 x 10^-1^ |
| *KRT38* | 37 | 5.40 x 10^-2^ | 3.92 x 10^-1^ |
| *KRT7* | 46 | 1.29 x 10^-1^ | 9.35 x 10^-1^ |
| *KRT8* | 24 | 2.03 x 10^-1^ | 8.20 x 10^-1^ |
| *KRTAP10^-1^2* | 35 | 4.46 x 10^-3^ | 1.21 x 10^-1^ |
| *KRTAP4-5* | 11 | 3.81 x 10^-1^ | 9.49 x 10^-1^ |
| *KRTCAP2* | 8 | 4.28 x 10^-1^ | 8.75 x 10^-1^ |
| *LAD1* | 42 | 4.34 x 10^-2^ | 8.80 x 10^-1^ |
| *LAIR1* | 33 | 4.82 x 10^-3^ | 6.84 x 10^-2^ |
| *LAMA5* | 24 | 1.98 x 10^-2^ | 2.87 x 10^-1^ |
| *LAMB2* | 8 | 2.20 x 10^-1^ | 8.75 x 10^-1^ |
| *LAMB3* | 35 | 3.45 x 10^-2^ | 4.49 x 10^-1^ |
| *LAMC1* | 31 | 1.39 x 10^-1^ | 7.92 x 10^-1^ |
| *LAMC3* | 48 | 7.06 x 10^-2^ | 7.13 x 10^-1^ |
| *LAMP1* | 13 | 3.79 x 10^-2^ | 4.93 x 10^-1^ |
| *LAMP3* | 33 | 1.62 x 10^-2^ | 5.33 x 10^-1^ |
| *LAP3* | 10 | 7.24 x 10^-2^ | 4.12 x 10^-1^ |
| *LAPTM4A* | 24 | 5.06 x 10^-2^ | 3.26 x 10^-1^ |
| *LAPTM4B* | 38 | 2.35 x 10^-2^ | 2.70 x 10^-1^ |
| *LAPTM5* | 33 | 4.44 x 10^-2^ | 8.35 x 10^-1^ |
| *LARP1* | 25 | 1.71 x 10^-3^ | 4.27 x 10^-2^ |
| *LARP6* | 15 | 8.11 x 10^-2^ | 9.33 x 10^-1^ |
| *LASP1* | 36 | 1.02 x 10^-2^ | 2.53 x 10^-1^ |
| *LASS2* | 13 | 6.94 x 10^-3^ | 1.93 x 10^-2^ |
| *LAT2* | 7 | 6.34 x 10^-2^ | 4.43 x 10^-1^ |
| *LATS2* | 37 | 8.49 x 10^-2^ | 9.42 x 10^-1^ |
| *LBH* | 40 | 2.83 x 10^-2^ | 3.29 x 10^-1^ |
| *LCE3D* | 5 | 7.89 x 10^-1^ | 9.91 x 10^-1^ |
| *LCMT1* | 27 | 1.47 x 10^-1^ | 9.81 x 10^-1^ |
| *LCN2* | 13 | 8.05 x 10^-2^ | 4.07 x 10^-1^ |
| *LCP1* | 45 | 9.27 x 10^-3^ | 1.64 x 10^-1^ |
| *LDHA* | 14 | 9.97 x 10^-2^ | 3.42 x 10^-1^ |
| *LDHB* | 12 | 9.69 x 10^-2^ | 4.35 x 10^-1^ |
| *LDLRAP1* | 9 | 5.60 x 10^-1^ | 9.22 x 10^-1^ |
| *LDOC1L* | 29 | 4.56 x 10^-2^ | 6.14 x 10^-1^ |
| *LEMD2* | 47 | 1.47 x 10^-2^ | 3.02 x 10^-1^ |
| *LEPRE1* | 15 | 1.34 x 10^-2^ | 1.34 x 10^-1^ |
| *LEPREL1* | 68 | 4.05 x 10^-2^ | 8.16 x 10^-1^ |
| *LEPROTL1* | 12 | 1.40 x 10^-2^ | 8.50 x 10^-2^ |
| *LETMD1* | 18 | 2.05 x 10^-1^ | 8.46 x 10^-1^ |
| *LGALS1* | 15 | 1.14 x 10^-2^ | 1.19 x 10^-1^ |
| *LGALS3* | 11 | 3.15 x 10^-1^ | 9.45 x 10^-1^ |
| *LGALS3BP* | 23 | 3.69 x 10^-2^ | 8.49 x 10^-1^ |
| *LGALS9* | 16 | 3.16 x 10^-2^ | 7.62 x 10^-2^ |
| *LGI3* | 23 | 1.02 x 10^-2^ | 9.05 x 10^-2^ |
| *LGMN* | 36 | 2.06 x 10^-2^ | 2.28 x 10^-1^ |
| *LGTN* | 25 | 9.59 x 10^-2^ | 5.85 x 10^-1^ |
| *LHFP* | 98 | 8.22 x 10^-2^ | 9.55 x 10^-1^ |
| *LHFPL2* | 45 | 2.22 x 10^-1^ | 9.72 x 10^-1^ |
| *LHPP* | 94 | 5.12 x 10^-2^ | 9.18 x 10^-1^ |
| *LHX3* | 16 | 9.19 x 10^-2^ | 8.86 x 10^-1^ |
| *LHX4* | 50 | 3.33 x 10^-2^ | 3.64 x 10^-1^ |
| *LIMA1* | 14 | 3.61 x 10^-1^ | 9.80 x 10^-1^ |
| *LIMS2* | 23 | 1.70 x 10^-2^ | 3.85 x 10^-1^ |
| *LIPA* | 35 | 4.76 x 10^-3^ | 1.23 x 10^-1^ |
| *LIPH* | 8 | 3.78 x 10^-1^ | 7.63 x 10^-1^ |
| *LITAF* | 26 | 3.76 x 10^-3^ | 9.77 x 10^-2^ |
| *LLGL2* | 34 | 3.19 x 10^-2^ | 9.61 x 10^-1^ |
| *LMAN2* | 13 | 1.61 x 10^-1^ | 8.86 x 10^-1^ |
| *LMAN2L* | 5 | 3.19 x 10^-2^ | 1.35 x 10^-1^ |
| *LMBRD1* | 46 | 9.40 x 10^-3^ | 3.81 x 10^-1^ |
| *LMCD1* | 55 | 1.08 x 10^-3^ | 2.50 x 10^-2^ |
| *LMNA* | 17 | 8.48 x 10^-3^ | 7.97 x 10^-2^ |
| *LMO2* | 32 | 2.91 x 10^-2^ | 8.09 x 10^-1^ |
| *LMO3* | 34 | 3.59 x 10^-3^ | 7.12 x 10^-2^ |
| *LOC201164* | 20 | 2.53 x 10^-2^ | 5.06 x 10^-1^ |
| *LOC201229* | 16 | 3.18 x 10^-2^ | 1.90 x 10^-1^ |
| *LOC342897* | 13 | 1.59 x 10^-1^ | 6.92 x 10^-1^ |
| *LOC57228* | 14 | 3.85 x 10^-2^ | 5.39 x 10^-1^ |
| *LOC87769* | 15 | 4.27 x 10^-2^ | 4.77 x 10^-1^ |
| *LOC91461* | 27 | 8.30 x 10^-2^ | 9.08 x 10^-1^ |
| *LONP1* | 9 | 1.34 x 10^-1^ | 8.45 x 10^-1^ |
| *LOXL1* | 32 | 7.03 x 10^-3^ | 1.45 x 10^-1^ |
| *LPCAT1* | 58 | 2.43 x 10^-2^ | 4.69 x 10^-1^ |
| *LPL* | 31 | 3.84 x 10^-2^ | 6.46 x 10^-1^ |
| *LPXN* | 12 | 3.97 x 10^-1^ | 7.96 x 10^-1^ |
| *LRCH4* | 6 | 3.79 x 10^-1^ | 1.00 |
| *LRIG1* | 46 | 1.72 x 10^-3^ | 3.89 x 10^-2^ |
| *LRP1* | 21 | 8.83 x 10^-3^ | 6.92 x 10^-2^ |
| *LRP10* | 22 | 3.76 x 10^-2^ | 7.90 x 10^-1^ |
| *LRP5* | 26 | 4.06 x 10^-2^ | 3.96 x 10^-1^ |
| *LRPAP1* | 23 | 5.41 x 10^-2^ | 9.26 x 10^-1^ |
| *LRRC32* | 41 | 4.03 x 10^-2^ | 8.73 x 10^-1^ |
| *LRRC47* | 23 | 1.67 x 10^-2^ | 3.22 x 10^-1^ |
| *LRRC4C* | 84 | 2.24 x 10^-3^ | 1.45 x 10^-1^ |
| *LRRC59* | 18 | 3.79 x 10^-3^ | 5.88 x 10^-2^ |
| *LRRC8A* | 12 | 2.07 x 10^-2^ | 2.49 x 10^-1^ |
| *LRRFIP1* | 45 | 2.64 x 10^-1^ | 9.78 x 10^-1^ |
| *LRRN4* | 35 | 6.43 x 10^-2^ | 9.46 x 10^-1^ |
| *LRRTM1* | 29 | 1.38 x 10^-1^ | 9.28 x 10^-1^ |
| *LSM10* | 19 | 3.07 x 10^-1^ | 9.60 x 10^-1^ |
| *LSM2* | 11 | 1.90 x 10^-2^ | 1.05 x 10^-1^ |
| *LSM4* | 34 | 1.21 x 10^-2^ | 3.98 x 10^-1^ |
| *LSM6* | 19 | 3.08 x 10^-2^ | 5.84 x 10^-1^ |
| *LSM7* | 15 | 1.23 x 10^-1^ | 6.97 x 10^-1^ |
| *LSMD1* | 22 | 6.16 x 10^-2^ | 4.89 x 10^-1^ |
| *LSR* | 32 | 2.65 x 10^-3^ | 6.39 x 10^-2^ |
| *LST1* | 36 | 2.70 x 10^-2^ | 5.25 x 10^-1^ |
| *LTA4H* | 58 | 2.35 x 10^-2^ | 5.89 x 10^-1^ |
| *LTBP2* | 43 | 5.06 x 10^-2^ | 9.31 x 10^-1^ |
| *LTBP3* | 11 | 5.82 x 10^-2^ | 6.40 x 10^-1^ |
| *LTBP4* | 20 | 2.13 x 10^-1^ | 9.65 x 10^-1^ |
| *LTBR* | 27 | 2.33 x 10^-1^ | 8.88 x 10^-1^ |
| *LTC4S* | 13 | 1.44 x 10^-1^ | 8.86 x 10^-1^ |
| *LTF* | 14 | 4.66 x 10^-2^ | 6.53 x 10^-1^ |
| *LY6E* | 11 | 1.93 x 10^-1^ | 3.74 x 10^-1^ |
| *LY86* | 72 | 1.16 x 10^-2^ | 6.14 x 10^-1^ |
| *LYK5* | 8 | 2.54 x 10^-1^ | 2.04 x 10^-1^ |
| *LYPLA2* | 7 | 2.59 x 10^-1^ | 8.20 x 10^-1^ |
| *LYSMD2* | 25 | 3.24 x 10^-2^ | 6.34 x 10^-1^ |
| *LYZ* | 20 | 1.29 x 10^-1^ | 9.33 x 10^-1^ |
| *LZTR1* | 41 | 7.45 x 10^-2^ | 9.29 x 10^-1^ |
| *LZTS2* | 15 | 1.40 x 10^-1^ | 8.61 x 10^-1^ |
| *M6PRBP1* | 29 | 6.67 x 10^-2^ | 9.60 x 10^-1^ |
| *MACF1* | 47 | 2.80 x 10^-2^ | 7.81 x 10^-1^ |
| *MACROD1* | 46 | 4.73 x 10^-3^ | 1.60 x 10^-1^ |
| *MAD1L1* | 79 | 8.39 x 10^-3^ | 2.31 x 10^-1^ |
| *MAD2L2* | 19 | 1.26 x 10^-2^ | 2.40 x 10^-1^ |
| *MADD* | 22 | 1.01 x 10^-1^ | 5.99 x 10^-1^ |
| *MAEA* | 23 | 5.72 x 10^-2^ | 9.47 x 10^-1^ |
| *MAFB* | 27 | 2.33 x 10^-1^ | 8.90 x 10^-1^ |
| *MAFF* | 19 | 6.31 x 10^-2^ | 5.98 x 10^-1^ |
| *MAGEF1* | 28 | 7.78 x 10^-2^ | 7.63 x 10^-1^ |
| *MAL* | 9 | 1.69 x 10^-1^ | 5.94 x 10^-1^ |
| *MALL* | 9 | 1.14 x 10^-1^ | 9.59 x 10^-1^ |
| *MAML1* | 10 | 1.41 x 10^-1^ | 8.18 x 10^-1^ |
| *MAML2* | 173 | 5.96 x 10^-4^ | 9.11 x 10^-2^ |
| *MAN1B1* | 10 | 1.53 x 10^-1^ | 9.43 x 10^-1^ |
| *MAN1C1* | 31 | 1.53 x 10^-2^ | 3.25 x 10^-1^ |
| *MAN2B1* | 9 | 3.20 x 10^-2^ | 2.51 x 10^-1^ |
| *MAN2C1* | 5 | 3.52 x 10^-1^ | 9.87 x 10^-1^ |
| *MANEAL* | 6 | 1.64 x 10^-1^ | 3.70 x 10^-1^ |
| *MANSC1* | 35 | 1.53 x 10^-2^ | 2.65 x 10^-1^ |
| *MAP1LC3A* | 7 | 2.57 x 10^-1^ | 1.00 |
| *MAP1LC3B* | 18 | 7.18 x 10^-2^ | 8.73 x 10^-1^ |
| *MAP1S* | 27 | 3.61 x 10^-2^ | 5.02 x 10^-1^ |
| *MAP2K1* | 15 | 2.79 x 10^-1^ | 8.85 x 10^-1^ |
| *MAP2K2* | 20 | 5.16 x 10^-2^ | 5.51 x 10^-1^ |
| *MAP2K3* | 12 | 1.94 x 10^-1^ | 8.97 x 10^-1^ |
| *MAP3K11* | 12 | 2.56 x 10^-1^ | 9.12 x 10^-1^ |
| *MAP3K3* | 9 | 5.07 x 10^-2^ | 2.16 x 10^-1^ |
| *MAP3K6* | 9 | 7.36 x 10^-3^ | 4.90 x 10^-2^ |
| *MAP4* | 19 | 6.72 x 10^-2^ | 6.97 x 10^-1^ |
| *MAP7D1* | 14 | 7.95 x 10^-3^ | 1.11 x 10^-1^ |
| *MAPBPIP* | 12 | 4.57 x 10^-2^ | 3.15 x 10^-1^ |
| *MAPK1IP1L* | 13 | 3.15 x 10^-1^ | 8.06 x 10^-1^ |
| *MAPK3* | 9 | 1.84 x 10^-1^ | 7.23 x 10^-1^ |
| *MAPKAPK2* | 29 | 4.32 x 10^-2^ | 4.46 x 10^-1^ |
| *MAPKAPK3* | 7 | 1.13 x 10^-1^ | 5.73 x 10^-1^ |
| *MAPRE1* | 23 | 3.99 x 10^-2^ | 6.27 x 10^-1^ |
| *MARCKS* | 25 | 8.56 x 10^-4^ | 1.40 x 10^-2^ |
| *MARCKSL1* | 2 | 4.48 x 10^-1^ | 5.91 x 10^-1^ |
| *MARCO* | 40 | 6.44 x 10^-2^ | 8.95 x 10^-1^ |
| *MAT2B* | 30 | 4.09 x 10^-2^ | 7.00 x 10^-1^ |
| *MAX* | 29 | 3.76 x 10^-3^ | 5.24 x 10^-2^ |
| *MB* | 33 | 2.75 x 10^-2^ | 7.56 x 10^-1^ |
| *MBD3* | 13 | 1.23 x 10^-2^ | 1.22 x 10^-1^ |
| *MBOAT1* | 70 | 3.87 x 10^-2^ | 9.82 x 10^-1^ |
| *MBOAT7* | 26 | 3.71 x 10^-2^ | 8.12 x 10^-1^ |
| *MBP* | 111 | 8.80 x 10^-3^ | 6.17 x 10^-1^ |
| *MBTPS1* | 50 | 1.27 x 10^-1^ | 9.48 x 10^-1^ |
| *MCAM* | 23 | 1.14 x 10^-1^ | 9.74 x 10^-1^ |
| *MCC* | 140 | 5.32 x 10^-4^ | 3.33 x 10^-2^ |
| *MCCC2* | 27 | 1.92 x 10^-2^ | 3.85 x 10^-1^ |
| *MCF2L* | 48 | 7.30 x 10^-3^ | 3.43 x 10^-1^ |
| *MCL1* | 17 | 9.95 x 10^-4^ | 1.35 x 10^-2^ |
| *MCOLN1* | 24 | 1.59 x 10^-1^ | 9.17 x 10^-1^ |
| *MDH1* | 9 | 4.03 x 10^-1^ | 9.75 x 10^-1^ |
| *MDH2* | 6 | 3.98 x 10^-1^ | 9.58 x 10^-1^ |
| *MEA1* | 11 | 1.37 x 10^-1^ | 9.62 x 10^-1^ |
| *MED1* | 5 | 2.57 x 10^-1^ | 4.05 x 10^-1^ |
| *MED15* | 38 | 3.10 x 10^-2^ | 3.58 x 10^-1^ |
| *MED16* | 20 | 9.19 x 10^-2^ | 9.81 x 10^-1^ |
| *MED19* | 7 | 1.32 x 10^-1^ | 4.57 x 10^-1^ |
| *MED22* | 12 | 2.48 x 10^-1^ | 9.70 x 10^-1^ |
| *MED24* | 26 | 7.82 x 10^-3^ | 1.22 x 10^-1^ |
| *MED28* | 7 | 7.24 x 10^-2^ | 4.88 x 10^-1^ |
| *MEN1* | 14 | 8.89 x 10^-2^ | 5.47 x 10^-1^ |
| *MEPCE* | 9 | 4.71 x 10^-1^ | 9.09 x 10^-1^ |
| *MESDC1* | 19 | 2.74 x 10^-1^ | 7.91 x 10^-1^ |
| *MEST* | 15 | 3.15 x 10^-1^ | 8.07 x 10^-1^ |
| *METAP1* | 23 | 1.50 x 10^-3^ | 1.24 x 10^-2^ |
| *METT11D1* | 31 | 1.06 x 10^-2^ | 2.89 x 10^-1^ |
| *METTL1* | 12 | 5.23 x 10^-3^ | 1.28 x 10^-2^ |
| *METTL11A* | 29 | 2.00 x 10^-2^ | 5.72 x 10^-1^ |
| *METTL7A* | 15 | 8.97 x 10^-2^ | 4.26 x 10^-1^ |
| *METTL7B* | 25 | 5.80 x 10^-2^ | 9.79 x 10^-1^ |
| *METTL9* | 11 | 2.68 x 10^-1^ | 5.54 x 10^-1^ |
| *MFAP1* | 8 | 8.27 x 10^-2^ | 3.90 x 10^-1^ |
| *MFAP4* | 9 | 1.85 x 10^-2^ | 1.66 x 10^-1^ |
| *MFGE8* | 36 | 2.17 x 10^-1^ | 1.00 |
| *MFN2* | 19 | 2.65 x 10^-1^ | 7.96 x 10^-1^ |
| *MFRP* | 24 | 3.35 x 10^-2^ | 8.04 x 10^-1^ |
| *MFSD1* | 38 | 1.81 x 10^-2^ | 3.41 x 10^-1^ |
| *MFSD10* | 13 | 2.87 x 10^-1^ | 9.74 x 10^-1^ |
| *MFSD5* | 13 | 1.20 x 10^-1^ | 6.06 x 10^-1^ |
| *MGAT1* | 15 | 9.58 x 10^-2^ | 8.57 x 10^-1^ |
| *MGAT4B* | 15 | 1.44 x 10^-1^ | 8.63 x 10^-1^ |
| *MGC21874* | 27 | 1.41 x 10^-1^ | 9.14 x 10^-1^ |
| *MGC3196* | 10 | 5.80 x 10^-2^ | 3.61 x 10^-1^ |
| *MGLL* | 48 | 3.75 x 10^-2^ | 6.29 x 10^-1^ |
| *MGMT* | 86 | 7.20 x 10^-2^ | 9.72 x 10^-1^ |
| *MGP* | 16 | 2.33 x 10^-1^ | 6.51 x 10^-1^ |
| *MGRN1* | 18 | 2.68 x 10^-1^ | 8.89 x 10^-1^ |
| *MGST1* | 28 | 9.93 x 10^-2^ | 9.24 x 10^-1^ |
| *MGST2* | 60 | 8.27 x 10^-3^ | 2.42 x 10^-1^ |
| *MGST3* | 34 | 1.08 x 10^-2^ | 2.32 x 10^-1^ |
| *MICAL2* | 140 | 6.59 x 10^-3^ | 8.08 x 10^-1^ |
| *MICALCL* | 75 | 6.59 x 10^-3^ | 4.94 x 10^-1^ |
| *MICALL2* | 7 | 1.26 x 10^-1^ | 3.32 x 10^-1^ |
| *MIDN* | 9 | 3.71 x 10^-1^ | 8.19 x 10^-1^ |
| *MIF* | 36 | 1.16 x 10^-1^ | 9.89 x 10^-1^ |
| *MINK1* | 33 | 7.70 x 10^-3^ | 1.38 x 10^-1^ |
| *MKNK1* | 32 | 4.30 x 10^-2^ | 3.00 x 10^-1^ |
| *MKNK2* | 23 | 4.56 x 10^-2^ | 8.87 x 10^-1^ |
| *MKRN1* | 22 | 1.37 x 10^-2^ | 1.24 x 10^-1^ |
| *MLF2* | 20 | 1.71 x 10^-1^ | 9.64 x 10^-1^ |
| *MLH1* | 11 | 2.08 x 10^-2^ | 2.29 x 10^-1^ |
| *MLPH* | 53 | 1.28 x 10^-1^ | 8.38 x 10^-1^ |
| *MLX* | 7 | 1.54 x 10^-1^ | 5.82 x 10^-1^ |
| *MLYCD* | 36 | 2.66 x 10^-2^ | 6.51 x 10^-1^ |
| *MMEL1* | 7 | 2.63 x 10^-1^ | 8.88 x 10^-1^ |
| *MMP19* | 4 | 1.15 x 10^-1^ | 3.96 x 10^-1^ |
| *MMP2* | 48 | 9.01 x 10^-3^ | 3.47 x 10^-1^ |
| *MMP24* | 19 | 1.27 x 10^-1^ | 7.40 x 10^-1^ |
| *MMP9* | 23 | 1.37 x 10^-3^ | 1.93 x 10^-2^ |
| *MMRN2* | 21 | 4.58 x 10^-2^ | 6.28 x 10^-1^ |
| *MMS19* | 26 | 1.33 x 10^-1^ | 6.62 x 10^-1^ |
| *MOAP1* | 29 | 1.42 x 10^-1^ | 8.91 x 10^-1^ |
| *MOBKL2C* | 17 | 6.77 x 10^-2^ | 1.35 x 10^-1^ |
| *MORF4L1* | 18 | 3.79 x 10^-3^ | 3.54 x 10^-2^ |
| *MOSPD3* | 7 | 6.48 x 10^-3^ | 4.54 x 10^-2^ |
| *MPDU1* | 17 | 6.98 x 10^-2^ | 5.46 x 10^-1^ |
| *MPEG1* | 18 | 1.48 x 10^-1^ | 8.83 x 10^-1^ |
| *MPG* | 16 | 5.48 x 10^-3^ | 6.91 x 10^-2^ |
| *MPHOSPH6* | 40 | 8.30 x 10^-2^ | 6.07 x 10^-1^ |
| *MPP3* | 12 | 9.97 x 10^-2^ | 5.40 x 10^-1^ |
| *MPRIP* | 46 | 2.53 x 10^-2^ | 7.01 x 10^-1^ |
| *MPST* | 28 | 5.61 x 10^-2^ | 8.93 x 10^-1^ |
| *MRC1* | 13 | 4.17 x 10^-2^ | 3.40 x 10^-1^ |
| *MRC2* | 22 | 1.01 x 10^-1^ | 8.91 x 10^-1^ |
| *MRCL3* | 28 | 6.27 x 10^-2^ | 5.03 x 10^-1^ |
| *MRFAP1* | 28 | 5.80 x 10^-2^ | 9.29 x 10^-1^ |
| *MRLC2* | 32 | 7.03 x 10^-2^ | 7.56 x 10^-1^ |
| *MRO* | 40 | 2.38 x 10^-2^ | 5.03 x 10^-1^ |
| *MRP63* | 20 | 4.85 x 10^-2^ | 2.79 x 10^-1^ |
| *MRPL10* | 29 | 7.13 x 10^-2^ | 8.21 x 10^-1^ |
| *MRPL11* | 6 | 2.94 x 10^-3^ | 3.77 x 10^-3^ |
| *MRPL12* | 9 | 2.97 x 10^-2^ | 1.03 x 10^-1^ |
| *MRPL14* | 24 | 1.95 x 10^-3^ | 3.09 x 10^-2^ |
| *MRPL15* | 12 | 2.83 x 10^-1^ | 9.64 x 10^-1^ |
| *MRPL16* | 8 | 1.71 x 10^-2^ | 1.37 x 10^-1^ |
| *MRPL18* | 21 | 1.54 x 10^-2^ | 1.07 x 10^-1^ |
| *MRPL2* | 7 | 2.71 x 10^-1^ | 9.62 x 10^-1^ |
| *MRPL23* | 19 | 1.27 x 10^-2^ | 2.02 x 10^-1^ |
| *MRPL24* | 17 | 2.20 x 10^-2^ | 3.62 x 10^-1^ |
| *MRPL28* | 19 | 6.75 x 10^-2^ | 6.45 x 10^-1^ |
| *MRPL3* | 24 | 1.63 x 10^-1^ | 8.01 x 10^-1^ |
| *MRPL33* | 10 | 5.68 x 10^-2^ | 1.82 x 10^-1^ |
| *MRPL34* | 21 | 2.18 x 10^-1^ | 9.56 x 10^-1^ |
| *MRPL37* | 27 | 9.49 x 10^-2^ | 8.48 x 10^-1^ |
| *MRPL38* | 10 | 1.25 x 10^-1^ | 5.76 x 10^-1^ |
| *MRPL4* | 22 | 1.52 x 10^-2^ | 3.21 x 10^-1^ |
| *MRPL41* | 7 | 1.88 x 10^-1^ | 1.00 |
| *MRPL45* | 5 | 2.65 x 10^-1^ | 5.87 x 10^-1^ |
| *MRPL46* | 9 | 5.90 x 10^-3^ | 5.31 x 10^-2^ |
| *MRPL49* | 16 | 1.46 x 10^-1^ | 1.00 |
| *MRPL52* | 29 | 3.76 x 10^-2^ | 6.66 x 10^-1^ |
| *MRPS12* | 15 | 1.12 x 10^-1^ | 9.27 x 10^-1^ |
| *MRPS14* | 14 | 1.62 x 10^-2^ | 1.15 x 10^-1^ |
| *MRPS18A* | 20 | 1.49 x 10^-1^ | 7.36 x 10^-1^ |
| *MRPS25* | 11 | 1.57 x 10^-1^ | 9.88 x 10^-1^ |
| *MRPS26* | 27 | 1.17 x 10^-3^ | 2.96 x 10^-2^ |
| *MRPS35* | 26 | 7.67 x 10^-2^ | 7.53 x 10^-1^ |
| *MRPS36* | 9 | 1.22 x 10^-1^ | 2.67 x 10^-1^ |
| *MRPS5* | 7 | 1.69 x 10^-1^ | 6.96 x 10^-1^ |
| *MRPS7* | 11 | 1.26 x 10^-1^ | 9.34 x 10^-1^ |
| *MS4A4A* | 34 | 1.05 x 10^-3^ | 2.10 x 10^-2^ |
| *MS4A6A* | 11 | 3.39 x 10^-2^ | 8.77 x 10^-2^ |
| *MSLN* | 21 | 6.13 x 10^-2^ | 6.47 x 10^-1^ |
| *MSRA* | 203 | 1.53 x 10^-2^ | 9.42 x 10^-1^ |
| *MSRB2* | 23 | 2.34 x 10^-2^ | 2.22 x 10^-1^ |
| *MST1R* | 11 | 1.02 x 10^-1^ | 9.09 x 10^-1^ |
| *MT1F* | 19 | 1.13 x 10^-1^ | 7.43 x 10^-1^ |
| *MT1G* | 19 | 1.13 x 10^-1^ | 7.43 x 10^-1^ |
| *MT1H* | 19 | 1.13 x 10^-1^ | 7.43 x 10^-1^ |
| *MT1M* | 14 | 8.57 x 10^-2^ | 4.01 x 10^-1^ |
| *MT1X* | 21 | 1.13 x 10^-1^ | 8.03 x 10^-1^ |
| *MT2A* | 24 | 4.30 x 10^-5^ | 6.07 x 10^-4^ |
| *MTCH1* | 45 | 8.74 x 10^-2^ | 9.59 x 10^-1^ |
| *MTCH2* | 8 | 4.34 x 10^-1^ | 7.98 x 10^-1^ |
| *MTHFD1* | 24 | 2.57 x 10^-2^ | 2.59 x 10^-1^ |
| *MTMR14* | 19 | 1.62 x 10^-2^ | 2.88 x 10^-1^ |
| *MTPN* | 36 | 7.27 x 10^-2^ | 9.02 x 10^-1^ |
| *MTX1* | 6 | 4.28 x 10^-1^ | 7.85 x 10^-1^ |
| *MUC1* | 8 | 4.28 x 10^-1^ | 9.99 x 10^-1^ |
| *MUC5B* | 15 | 2.11 x 10^-1^ | 9.19 x 10^-1^ |
| *MUL1* | 24 | 7.21 x 10^-2^ | 8.49 x 10^-1^ |
| *MUS81* | 12 | 3.67 x 10^-2^ | 8.07 x 10^-2^ |
| *MVK* | 23 | 1.67 x 10^-2^ | 2.49 x 10^-1^ |
| *MVP* | 8 | 1.58 x 10^-1^ | 2.54 x 10^-1^ |
| *MX1* | 56 | 1.54 x 10^-2^ | 4.21 x 10^-1^ |
| *MX2* | 57 | 1.60 x 10^-2^ | 8.63 x 10^-1^ |
| *MXD4* | 23 | 2.21 x 10^-2^ | 4.89 x 10^-1^ |
| *MXI1* | 29 | 1.39 x 10^-1^ | 6.55 x 10^-1^ |
| *MXRA7* | 26 | 2.88 x 10^-3^ | 3.30 x 10^-2^ |
| *MYADM* | 14 | 2.35 x 10^-2^ | 8.94 x 10^-2^ |
| *MYD88* | 9 | 6.53 x 10^-2^ | 3.33 x 10^-1^ |
| *MYH10* | 24 | 2.14 x 10^-1^ | 9.90 x 10^-1^ |
| *MYH11* | 59 | 8.02 x 10^-2^ | 7.50 x 10^-1^ |
| *MYH9* | 52 | 3.68 x 10^-2^ | 7.82 x 10^-1^ |
| *MYL4* | 28 | 8.04 x 10^-2^ | 7.56 x 10^-1^ |
| *MYL6* | 9 | 8.79 x 10^-2^ | 3.96 x 10^-1^ |
| *MYL6B* | 9 | 8.79 x 10^-2^ | 3.96 x 10^-1^ |
| *MYL9* | 6 | 2.07 x 10^-1^ | 3.63 x 10^-1^ |
| *MYLIP* | 33 | 9.09 x 10^-3^ | 2.86 x 10^-1^ |
| *MYLK* | 45 | 4.44 x 10^-2^ | 5.41 x 10^-1^ |
| *MYO1C* | 22 | 5.62 x 10^-2^ | 5.25 x 10^-1^ |
| *MYO1F* | 22 | 4.62 x 10^-2^ | 4.44 x 10^-1^ |
| *MYO5C* | 39 | 3.98 x 10^-2^ | 5.67 x 10^-1^ |
| *MYST1* | 6 | 9.82 x 10^-2^ | 1.11 x 10^-1^ |
| *MYST2* | 11 | 3.84 x 10^-2^ | 3.64 x 10^-1^ |
| *MYST4* | 31 | 3.33 x 10^-2^ | 5.05 x 10^-1^ |
| *NAAA* | 21 | 3.99 x 10^-2^ | 7.78 x 10^-1^ |
| *NACA* | 8 | 7.88 x 10^-2^ | 4.85 x 10^-1^ |
| *NAGA* | 8 | 1.78 x 10^-1^ | 3.17 x 10^-1^ |
| *NAGK* | 28 | 9.94 x 10^-2^ | 9.74 x 10^-1^ |
| *NANP* | 10 | 9.09 x 10^-2^ | 2.38 x 10^-1^ |
| *NANS* | 20 | 3.51 x 10^-2^ | 7.03 x 10^-1^ |
| *NAP1L1* | 24 | 1.77 x 10^-1^ | 9.51 x 10^-1^ |
| *NAPA* | 17 | 9.73 x 10^-2^ | 3.97 x 10^-1^ |
| *NARS* | 38 | 7.89 x 10^-2^ | 9.74 x 10^-1^ |
| *NAT10* | 21 | 5.62 x 10^-2^ | 3.41 x 10^-1^ |
| *NAT5* | 39 | 1.88 x 10^-2^ | 7.33 x 10^-1^ |
| *NAV1* | 49 | 4.13 x 10^-2^ | 4.78 x 10^-1^ |
| *NBEAL2* | 8 | 1.39 x 10^-2^ | 2.27 x 10^-2^ |
| *NBL1* | 30 | 4.29 x 10^-2^ | 7.20 x 10^-1^ |
| *NBPF1* | 1 | 4.33 x 10^-1^ | 3.75 x 10^-1^ |
| *NCF1* | 1 | 8.27 x 10^-1^ | 8.27 x 10^-1^ |
| *NCF2* | 19 | 3.86 x 10^-2^ | 6.89 x 10^-1^ |
| *NCF4* | 45 | 3.54 x 10^-2^ | 7.60 x 10^-1^ |
| *NCK2* | 58 | 7.14 x 10^-4^ | 1.25 x 10^-2^ |
| *NCL* | 20 | 4.56 x 10^-2^ | 5.36 x 10^-1^ |
| *NCOA1* | 22 | 2.37 x 10^-1^ | 9.46 x 10^-1^ |
| *NCOA4* | 14 | 1.98 x 10^-1^ | 8.27 x 10^-1^ |
| *NCOA6* | 15 | 1.91 x 10^-1^ | 9.19 x 10^-1^ |
| *NCOR2* | 74 | 1.75 x 10^-2^ | 4.24 x 10^-1^ |
| *NCSTN* | 23 | 2.06 x 10^-1^ | 9.43 x 10^-1^ |
| *NDE1* | 25 | 8.63 x 10^-2^ | 2.80 x 10^-1^ |
| *NDEL1* | 12 | 4.85 x 10^-2^ | 5.62 x 10^-1^ |
| *NDFIP1* | 38 | 4.26 x 10^-3^ | 1.41 x 10^-1^ |
| *NDN* | 12 | 3.72 x 10^-2^ | 2.65 x 10^-1^ |
| *NDRG1* | 83 | 7.59 x 10^-3^ | 2.53 x 10^-1^ |
| *NDRG2* | 38 | 6.04 x 10^-3^ | 9.92 x 10^-2^ |
| *NDRG3* | 9 | 6.23 x 10^-2^ | 3.04 x 10^-1^ |
| *NDUFA10* | 50 | 3.47 x 10^-2^ | 4.31 x 10^-1^ |
| *NDUFA11* | 15 | 5.42 x 10^-2^ | 3.57 x 10^-1^ |
| *NDUFA12* | 43 | 4.75 x 10^-2^ | 9.56 x 10^-1^ |
| *NDUFA13* | 5 | 6.20 x 10^-3^ | 2.58 x 10^-2^ |
| *NDUFA3* | 23 | 3.87 x 10^-4^ | 8.87 x 10^-3^ |
| *NDUFA4* | 26 | 2.26 x 10^-1^ | 1.00 |
| *NDUFA6* | 6 | 1.78 x 10^-1^ | 2.12 x 10^-1^ |
| *NDUFA8* | 41 | 4.59 x 10^-2^ | 9.51 x 10^-1^ |
| *NDUFA9* | 31 | 6.70 x 10^-2^ | 7.41 x 10^-1^ |
| *NDUFAB1* | 6 | 1.93 x 10^-1^ | 8.30 x 10^-1^ |
| *NDUFB1* | 15 | 2.80 x 10^-1^ | 9.46 x 10^-1^ |
| *NDUFB10* | 21 | 1.55 x 10^-1^ | 9.64 x 10^-1^ |
| *NDUFB2* | 8 | 1.95 x 10^-1^ | 9.55 x 10^-1^ |
| *NDUFB3* | 5 | 5.68 x 10^-3^ | 1.87 x 10^-2^ |
| *NDUFB4* | 3 | 3.29 x 10^-1^ | 5.76 x 10^-1^ |
| *NDUFB5* | 11 | 7.83 x 10^-2^ | 5.63 x 10^-1^ |
| *NDUFB6* | 23 | 1.66 x 10^-1^ | 7.88 x 10^-1^ |
| *NDUFB7* | 19 | 2.97 x 10^-2^ | 5.64 x 10^-1^ |
| *NDUFB8* | 10 | 9.46 x 10^-3^ | 2.75 x 10^-2^ |
| *NDUFB9* | 35 | 2.87 x 10^-2^ | 8.92 x 10^-1^ |
| *NDUFC1* | 14 | 4.21 x 10^-2^ | 3.02 x 10^-1^ |
| *NDUFC2* | 20 | 6.42 x 10^-2^ | 3.19 x 10^-1^ |
| *NDUFS2* | 27 | 2.16 x 10^-2^ | 2.63 x 10^-1^ |
| *NDUFS5* | 11 | 6.91 x 10^-2^ | 7.60 x 10^-1^ |
| *NDUFS6* | 36 | 8.41 x 10^-2^ | 8.98 x 10^-1^ |
| *NDUFS7* | 19 | 1.33 x 10^-1^ | 7.87 x 10^-1^ |
| *NDUFS8* | 14 | 1.27 x 10^-2^ | 8.97 x 10^-2^ |
| *NDUFV1* | 19 | 9.51 x 10^-2^ | 7.23 x 10^-1^ |
| *NDUFV2* | 20 | 1.80 x 10^-2^ | 3.60 x 10^-1^ |
| *NECAB3* | 6 | 1.33 x 10^-1^ | 2.85 x 10^-1^ |
| *NECAP2* | 15 | 1.59 x 10^-1^ | 3.80 x 10^-1^ |
| *NEDD8* | 23 | 2.60 x 10^-2^ | 3.22 x 10^-1^ |
| *NEDD9* | 60 | 6.53 x 10^-2^ | 9.89 x 10^-1^ |
| *NEK11* | 40 | 1.30 x 10^-2^ | 5.22 x 10^-1^ |
| *NEK6* | 39 | 5.59 x 10^-2^ | 8.47 x 10^-1^ |
| *NENF* | 28 | 8.53 x 10^-3^ | 1.76 x 10^-1^ |
| *NEO1* | 42 | 1.13 x 10^-2^ | 3.14 x 10^-1^ |
| *NES* | 28 | 2.20 x 10^-2^ | 6.03 x 10^-1^ |
| *NEU1* | 17 | 6.09 x 10^-3^ | 2.94 x 10^-2^ |
| *NFAM1* | 41 | 4.54 x 10^-2^ | 7.89 x 10^-1^ |
| *NFE2L1* | 14 | 6.76 x 10^-2^ | 6.67 x 10^-1^ |
| *NFIC* | 24 | 1.22 x 10^-2^ | 2.00 x 10^-1^ |
| *NFIL3* | 20 | 5.00 x 10^-2^ | 3.70 x 10^-1^ |
| *NFKB1* | 35 | 7.17 x 10^-3^ | 2.45 x 10^-1^ |
| *NFKBIA* | 24 | 1.44 x 10^-1^ | 7.91 x 10^-1^ |
| *NFS1* | 11 | 3.52 x 10^-1^ | 8.12 x 10^-1^ |
| *NFYC* | 27 | 8.83 x 10^-2^ | 5.91 x 10^-1^ |
| *NGEF* | 57 | 5.30 x 10^-2^ | 8.42 x 10^-1^ |
| *NHEJ1* | 26 | 1.11 x 10^-1^ | 9.73 x 10^-1^ |
| *NHP2L1* | 3 | 1.36 x 10^-1^ | 4.02 x 10^-1^ |
| *NINJ1* | 25 | 2.21 x 10^-1^ | 9.53 x 10^-1^ |
| *NINJ2* | 68 | 1.66 x 10^-3^ | 1.08 x 10^-1^ |
| *NIPA2* | 42 | 2.71 x 10^-2^ | 9.31 x 10^-1^ |
| *NIPSNAP1* | 16 | 3.59 x 10^-1^ | 8.95 x 10^-1^ |
| *NISCH* | 11 | 1.93 x 10^-1^ | 1.00 |
| *NIT1* | 9 | 3.09 x 10^-2^ | 2.78 x 10^-1^ |
| *NKD2* | 19 | 7.05 x 10^-2^ | 8.67 x 10^-1^ |
| *NKG7* | 29 | 5.05 x 10^-2^ | 5.60 x 10^-1^ |
| *NKX2^-1^* | 20 | 1.46 x 10^-1^ | 8.38 x 10^-1^ |
| *NMB* | 13 | 1.38 x 10^-1^ | 7.32 x 10^-1^ |
| *NME1* | 8 | 1.70 x 10^-1^ | 7.42 x 10^-1^ |
| *NME3* | 16 | 2.88 x 10^-2^ | 1.53 x 10^-1^ |
| *NME4* | 20 | 1.83 x 10^-1^ | 8.61 x 10^-1^ |
| *NNMT* | 30 | 1.68 x 10^-3^ | 3.61 x 10^-2^ |
| *NOB1* | 11 | 1.45 x 10^-1^ | 6.78 x 10^-1^ |
| *NOD1* | 28 | 9.02 x 10^-2^ | 9.37 x 10^-1^ |
| *NOL5A* | 32 | 6.49 x 10^-2^ | 9.67 x 10^-1^ |
| *NOL9* | 21 | 6.74 x 10^-2^ | 8.20 x 10^-1^ |
| *NOLA2* | 20 | 1.52 x 10^-2^ | 2.55 x 10^-1^ |
| *NOLA3* | 17 | 1.38 x 10^-1^ | 8.89 x 10^-1^ |
| *NOSIP* | 11 | 1.69 x 10^-1^ | 6.50 x 10^-1^ |
| *NOTCH2* | 21 | 2.69 x 10^-2^ | 2.11 x 10^-1^ |
| *NOTCH3* | 19 | 3.27 x 10^-2^ | 2.14 x 10^-1^ |
| *NOTCH4* | 67 | 1.18 x 10^-2^ | 7.92 x 10^-1^ |
| *NP* | 33 | 5.55 x 10^-2^ | 8.02 x 10^-1^ |
| *NPAL2* | 46 | 2.32 x 10^-3^ | 1.01 x 10^-1^ |
| *NPC1* | 26 | 7.66 x 10^-3^ | 1.99 x 10^-1^ |
| *NPC2* | 19 | 6.90 x 10^-2^ | 5.25 x 10^-1^ |
| *NPDC1* | 10 | 1.53 x 10^-1^ | 1.00 |
| *NPLOC4* | 20 | 1.54 x 10^-2^ | 8.09 x 10^-2^ |
| *NPM1* | 16 | 1.15 x 10^-1^ | 6.84 x 10^-1^ |
| *NPM2* | 18 | 8.05 x 10^-2^ | 8.54 x 10^-1^ |
| *NPR1* | 17 | 3.43 x 10^-1^ | 9.26 x 10^-1^ |
| *NPTN* | 17 | 1.23 x 10^-1^ | 2.03 x 10^-1^ |
| *NQO1* | 11 | 3.39 x 10^-1^ | 9.66 x 10^-1^ |
| *NR1H2* | 12 | 3.76 x 10^-2^ | 2.17 x 10^-1^ |
| *NR1H3* | 15 | 1.01 x 10^-1^ | 4.88 x 10^-1^ |
| *NR2C2AP* | 13 | 3.73 x 10^-3^ | 4.84 x 10^-2^ |
| *NR2F2* | 19 | 5.58 x 10^-2^ | 4.65 x 10^-1^ |
| *NR4A1* | 19 | 1.21 x 10^-1^ | 8.40 x 10^-1^ |
| *NRGN* | 21 | 1.19 x 10^-1^ | 8.87 x 10^-1^ |
| *NRM* | 18 | 2.38 x 10^-2^ | 1.48 x 10^-1^ |
| *NRN1* | 35 | 5.17 x 10^-2^ | 4.86 x 10^-1^ |
| *NSFL1C* | 45 | 7.92 x 10^-2^ | 6.56 x 10^-1^ |
| *NSMCE1* | 27 | 1.00 x 10^-2^ | 2.60 x 10^-1^ |
| *NSUN5* | 3 | 5.46 x 10^-1^ | 7.64 x 10^-1^ |
| *NT5C1A* | 25 | 1.24 x 10^-2^ | 2.78 x 10^-1^ |
| *NT5C3L* | 15 | 1.04 x 10^-2^ | 8.12 x 10^-2^ |
| *NUAK1* | 45 | 2.39 x 10^-2^ | 9.87 x 10^-1^ |
| *NUAK2* | 33 | 8.93 x 10^-2^ | 9.00 x 10^-1^ |
| *NUBP1* | 26 | 1.93 x 10^-1^ | 9.46 x 10^-1^ |
| *NUBP2* | 18 | 2.88 x 10^-2^ | 1.43 x 10^-1^ |
| *NUCB1* | 28 | 1.55 x 10^-2^ | 3.35 x 10^-1^ |
| *NUDC* | 9 | 3.80 x 10^-1^ | 7.03 x 10^-1^ |
| *NUDCD3* | 18 | 3.78 x 10^-2^ | 4.10 x 10^-1^ |
| *NUDT16L1* | 15 | 2.68 x 10^-1^ | 7.33 x 10^-1^ |
| *NUDT3* | 8 | 8.49 x 10^-2^ | 6.79 x 10^-1^ |
| *NUMA1* | 8 | 2.79 x 10^-1^ | 7.50 x 10^-1^ |
| *NUP214* | 24 | 6.78 x 10^-2^ | 3.64 x 10^-1^ |
| *NUTF2* | 5 | 6.28 x 10^-1^ | 6.90 x 10^-1^ |
| *NXF1* | 9 | 5.80 x 10^-2^ | 3.27 x 10^-1^ |
| *NXN* | 92 | 3.06 x 10^-3^ | 2.60 x 10^-1^ |
| *NXPH3* | 12 | 4.06 x 10^-2^ | 2.96 x 10^-1^ |
| *NXT1* | 29 | 1.24 x 10^-1^ | 8.61 x 10^-1^ |
| *OASL* | 27 | 1.11 x 10^-2^ | 2.99 x 10^-1^ |
| *OAT* | 35 | 6.12 x 10^-2^ | 8.79 x 10^-1^ |
| *OAZ1* | 12 | 1.57 x 10^-1^ | 7.03 x 10^-1^ |
| *OAZ2* | 8 | 4.24 x 10^-1^ | 8.06 x 10^-1^ |
| *OCEL1* | 21 | 6.42 x 10^-2^ | 9.62 x 10^-1^ |
| *OCIAD1* | 7 | 9.79 x 10^-2^ | 8.12 x 10^-2^ |
| *ODC1* | 26 | 8.45 x 10^-2^ | 9.21 x 10^-1^ |
| *OGDH* | 21 | 1.86 x 10^-1^ | 9.52 x 10^-1^ |
| *OLFML2A* | 15 | 4.98 x 10^-3^ | 4.50 x 10^-2^ |
| *OLFML3* | 23 | 9.81 x 10^-2^ | 7.69 x 10^-1^ |
| *OPN5* | 35 | 3.17 x 10^-2^ | 7.29 x 10^-1^ |
| *OR10A5* | 25 | 1.52 x 10^-1^ | 5.80 x 10^-1^ |
| *OR13J1* | 25 | 2.06 x 10^-2^ | 3.93 x 10^-1^ |
| *OR1C1* | 19 | 1.51 x 10^-1^ | 8.38 x 10^-1^ |
| *OR2D3* | 32 | 1.45 x 10^-1^ | 7.65 x 10^-1^ |
| *OR4D1* | 31 | 1.21 x 10^-1^ | 1.00 |
| *OR4M2* | 1 | 6.08 x 10^-1^ | 6.08 x 10^-1^ |
| *OR5AP2* | 20 | 4.84 x 10^-2^ | 3.26 x 10^-1^ |
| *OR7D4* | 19 | 1.78 x 10^-1^ | 9.70 x 10^-1^ |
| *ORAI1* | 9 | 2.48 x 10^-1^ | 7.51 x 10^-1^ |
| *ORAI3* | 4 | 5.32 x 10^-1^ | 6.75 x 10^-1^ |
| *ORMDL2* | 5 | 2.55 x 10^-1^ | 8.62 x 10^-1^ |
| *OSTF1* | 37 | 1.02 x 10^-1^ | 9.76 x 10^-1^ |
| *OTUB1* | 15 | 1.68 x 10^-1^ | 7.77 x 10^-1^ |
| *OTX2* | 39 | 1.92 x 10^-2^ | 3.86 x 10^-1^ |
| *OXA1L* | 38 | 1.01 x 10^-1^ | 8.77 x 10^-1^ |
| *OXER1* | 26 | 4.39 x 10^-2^ | 3.83 x 10^-1^ |
| *P2RX4* | 35 | 2.93 x 10^-2^ | 4.88 x 10^-1^ |
| *P4HA2* | 20 | 1.62 x 10^-2^ | 2.20 x 10^-1^ |
| *P4HA3* | 13 | 1.55 x 10^-1^ | 7.68 x 10^-1^ |
| *PABPC1* | 29 | 2.72 x 10^-1^ | 9.91 x 10^-1^ |
| *PABPC3* | 28 | 6.14 x 10^-2^ | 9.87 x 10^-1^ |
| *PABPC4* | 13 | 6.58 x 10^-2^ | 2.84 x 10^-1^ |
| *PABPN1* | 17 | 2.54 x 10^-1^ | 9.48 x 10^-1^ |
| *PACS2* | 3 | 2.18 x 10^-1^ | 5.80 x 10^-1^ |
| *PACSIN2* | 23 | 1.76 x 10^-1^ | 8.00 x 10^-1^ |
| *PADI2* | 31 | 1.14 x 10^-1^ | 7.07 x 10^-1^ |
| *PAG1* | 73 | 2.02 x 10^-2^ | 6.89 x 10^-1^ |
| *PAK4* | 24 | 2.03 x 10^-2^ | 4.87 x 10^-1^ |
| *PALM* | 31 | 2.79 x 10^-2^ | 8.10 x 10^-1^ |
| *PALM2* | 119 | 4.54 x 10^-3^ | 4.35 x 10^-1^ |
| *PAM* | 30 | 6.02 x 10^-2^ | 9.93 x 10^-1^ |
| *PANK2* | 26 | 6.52 x 10^-2^ | 7.42 x 10^-1^ |
| *PANK4* | 15 | 1.04 x 10^-1^ | 5.06 x 10^-1^ |
| *PAPSS1* | 36 | 8.40 x 10^-2^ | 9.36 x 10^-1^ |
| *PAPSS2* | 40 | 1.09 x 10^-2^ | 3.43 x 10^-1^ |
| *PAQR7* | 10 | 3.37 x 10^-2^ | 1.22 x 10^-1^ |
| *PARK7* | 11 | 2.26 x 10^-1^ | 7.53 x 10^-1^ |
| *PARL* | 32 | 6.74 x 10^-2^ | 4.33 x 10^-1^ |
| *PARP1* | 25 | 8.50 x 10^-2^ | 9.90 x 10^-1^ |
| *PARP12* | 26 | 1.02 x 10^-1^ | 8.91 x 10^-1^ |
| *PARP16* | 16 | 2.57 x 10^-1^ | 8.75 x 10^-1^ |
| *PARP2* | 23 | 9.04 x 10^-3^ | 1.02 x 10^-1^ |
| *PARP4* | 31 | 2.13 x 10^-2^ | 4.24 x 10^-1^ |
| *PCBD1* | 37 | 1.87 x 10^-2^ | 5.12 x 10^-1^ |
| *PCBP1* | 6 | 3.77 x 10^-1^ | 9.28 x 10^-1^ |
| *PCBP2* | 11 | 2.13 x 10^-1^ | 7.18 x 10^-1^ |
| *PCBP4* | 4 | 1.41 x 10^-1^ | 3.37 x 10^-1^ |
| *PCDH12* | 30 | 4.35 x 10^-2^ | 6.00 x 10^-1^ |
| *PCDH17* | 31 | 3.80 x 10^-2^ | 9.84 x 10^-1^ |
| *PCDH18* | 14 | 1.80 x 10^-1^ | 8.24 x 10^-1^ |
| *PCDHGC3* | 22 | 1.62 x 10^-1^ | 7.45 x 10^-1^ |
| *PCGF2* | 21 | 5.55 x 10^-2^ | 8.00 x 10^-1^ |
| *PCGF5* | 28 | 8.29 x 10^-4^ | 1.78 x 10^-2^ |
| *PCK2* | 14 | 6.56 x 10^-2^ | 8.53 x 10^-1^ |
| *PCMT1* | 8 | 1.66 x 10^-1^ | 6.34 x 10^-1^ |
| *PCOLCE* | 7 | 6.48 x 10^-3^ | 4.54 x 10^-2^ |
| *PDAP1* | 8 | 2.78 x 10^-2^ | 1.57 x 10^-1^ |
| *PDCD6* | 15 | 6.63 x 10^-3^ | 9.39 x 10^-2^ |
| *PDCD6IP* | 20 | 1.62 x 10^-1^ | 6.17 x 10^-1^ |
| *PDE2A* | 59 | 1.87 x 10^-2^ | 8.98 x 10^-1^ |
| *PDE4C* | 31 | 1.21 x 10^-2^ | 3.50 x 10^-1^ |
| *PDGFRB* | 57 | 1.38 x 10^-2^ | 7.87 x 10^-1^ |
| *PDHB* | 10 | 8.79 x 10^-2^ | 3.71 x 10^-1^ |
| *PDIA2* | 23 | 4.60 x 10^-2^ | 4.57 x 10^-1^ |
| *PDIA3* | 6 | 6.09 x 10^-1^ | 9.80 x 10^-1^ |
| *PDIA4* | 17 | 5.92 x 10^-3^ | 1.01 x 10^-1^ |
| *PDIA5* | 45 | 2.41 x 10^-2^ | 6.13 x 10^-1^ |
| *PDIA6* | 41 | 2.04 x 10^-2^ | 3.43 x 10^-1^ |
| *PDLIM1* | 39 | 8.54 x 10^-2^ | 9.09 x 10^-1^ |
| *PDLIM2* | 17 | 1.09 x 10^-1^ | 5.49 x 10^-1^ |
| *PDXDC1* | 11 | 1.46 x 10^-1^ | 3.66 x 10^-1^ |
| *PDXK* | 27 | 1.59 x 10^-2^ | 3.60 x 10^-1^ |
| *PDZK1IP1* | 19 | 1.24 x 10^-1^ | 5.18 x 10^-1^ |
| *PEA15* | 34 | 6.30 x 10^-4^ | 1.75 x 10^-2^ |
| *PEBP1* | 16 | 8.58 x 10^-2^ | 5.94 x 10^-1^ |
| *PECAM1* | 21 | 6.88 x 10^-3^ | 1.24 x 10^-1^ |
| *PELI3* | 11 | 2.94 x 10^-3^ | 1.87 x 10^-2^ |
| *PEMT* | 29 | 1.77 x 10^-1^ | 8.91 x 10^-1^ |
| *PEPD* | 55 | 3.62 x 10^-3^ | 9.51 x 10^-2^ |
| *PER1* | 26 | 3.00 x 10^-2^ | 4.83 x 10^-1^ |
| *PERLD1* | 10 | 1.23 x 10^-1^ | 2.88 x 10^-1^ |
| *PERP* | 36 | 5.47 x 10^-2^ | 4.99 x 10^-1^ |
| *PEX14* | 53 | 7.79 x 10^-3^ | 1.84 x 10^-1^ |
| *PEX16* | 12 | 9.36 x 10^-2^ | 4.65 x 10^-1^ |
| *PFDN1* | 20 | 3.01 x 10^-1^ | 9.95 x 10^-1^ |
| *PFDN2* | 11 | 3.09 x 10^-2^ | 2.96 x 10^-1^ |
| *PFDN5* | 11 | 2.77 x 10^-1^ | 7.91 x 10^-1^ |
| *PFKFB3* | 75 | 1.55 x 10^-2^ | 8.59 x 10^-1^ |
| *PFKL* | 28 | 8.18 x 10^-2^ | 6.66 x 10^-1^ |
| *PFKM* | 18 | 1.08 x 10^-1^ | 5.15 x 10^-1^ |
| *PFKP* | 83 | 3.30 x 10^-5^ | 1.20 x 10^-3^ |
| *PFN1* | 15 | 8.35 x 10^-3^ | 1.25 x 10^-1^ |
| *PFN2* | 11 | 2.35 x 10^-2^ | 2.32 x 10^-1^ |
| *PGAM1* | 12 | 1.81 x 10^-1^ | 5.93 x 10^-1^ |
| *PGC* | 19 | 2.33 x 10^-2^ | 3.17 x 10^-1^ |
| *PGD* | 12 | 2.15 x 10^-2^ | 9.18 x 10^-2^ |
| *PGF* | 15 | 2.98 x 10^-2^ | 4.34 x 10^-1^ |
| *PGLS* | 21 | 1.97 x 10^-2^ | 3.88 x 10^-1^ |
| *PGLYRP3* | 29 | 2.13 x 10^-2^ | 3.48 x 10^-1^ |
| *PGLYRP4* | 27 | 2.13 x 10^-2^ | 3.60 x 10^-1^ |
| *PGM1* | 34 | 2.99 x 10^-1^ | 9.76 x 10^-1^ |
| *PGS1* | 29 | 2.19 x 10^-2^ | 6.14 x 10^-1^ |
| *PH-4* | 6 | 1.26 x 10^-1^ | 1.84 x 10^-1^ |
| *PHB* | 25 | 6.61 x 10^-2^ | 7.60 x 10^-1^ |
| *PHB2* | 11 | 3.17 x 10^-2^ | 2.20 x 10^-1^ |
| *PHC2* | 19 | 2.30 x 10^-1^ | 9.56 x 10^-1^ |
| *PHF1* | 10 | 9.25 x 10^-4^ | 5.70 x 10^-3^ |
| *PHF13* | 25 | 6.74 x 10^-2^ | 7.66 x 10^-1^ |
| *PHF15* | 33 | 3.29 x 10^-3^ | 5.95 x 10^-2^ |
| *PHF5A* | 12 | 7.88 x 10^-2^ | 7.66 x 10^-1^ |
| *PHGDH* | 37 | 1.59 x 10^-1^ | 9.09 x 10^-1^ |
| *PHKG2* | 3 | 3.78 x 10^-1^ | 7.00 x 10^-1^ |
| *PHLDA3* | 34 | 9.30 x 10^-2^ | 9.03 x 10^-1^ |
| *PHOSPHO1* | 17 | 1.39 x 10^-2^ | 7.55 x 10^-2^ |
| *PHPT1* | 7 | 3.12 x 10^-1^ | 5.06 x 10^-1^ |
| *PHYHD1* | 12 | 2.18 x 10^-1^ | 9.24 x 10^-1^ |
| *PI16* | 38 | 8.74 x 10^-2^ | 9.59 x 10^-1^ |
| *PI4KA* | 21 | 2.86 x 10^-1^ | 1.00 |
| *PI4KB* | 16 | 6.68 x 10^-3^ | 3.37 x 10^-2^ |
| *PIF1* | 11 | 2.83 x 10^-1^ | 8.89 x 10^-1^ |
| *PIGG* | 21 | 8.58 x 10^-2^ | 3.67 x 10^-1^ |
| *PIGP* | 14 | 3.18 x 10^-2^ | 2.41 x 10^-1^ |
| *PIGR* | 17 | 4.89 x 10^-3^ | 6.28 x 10^-2^ |
| *PIGT* | 28 | 1.09 x 10^-2^ | 2.23 x 10^-1^ |
| *PIGY* | 16 | 2.51 x 10^-1^ | 7.03 x 10^-1^ |
| *PIH1D1* | 17 | 2.85 x 10^-2^ | 4.84 x 10^-1^ |
| *PIK3C2B* | 36 | 1.73 x 10^-2^ | 3.31 x 10^-1^ |
| *PIK3IP1* | 15 | 4.69 x 10^-2^ | 3.29 x 10^-1^ |
| *PIM1* | 22 | 1.00 x 10^-2^ | 2.21 x 10^-1^ |
| *PINK1* | 31 | 1.91 x 10^-2^ | 3.00 x 10^-1^ |
| *PIP5K1C* | 27 | 3.37 x 10^-2^ | 7.52 x 10^-1^ |
| *PITPNA* | 29 | 5.47 x 10^-2^ | 4.18 x 10^-1^ |
| *PITPNB* | 28 | 1.04 x 10^-3^ | 1.96 x 10^-2^ |
| *PITPNC1* | 71 | 2.26 x 10^-2^ | 9.11 x 10^-1^ |
| *PKD1* | 10 | 8.35 x 10^-2^ | 8.35 x 10^-1^ |
| *PKIG* | 23 | 3.80 x 10^-2^ | 3.19 x 10^-1^ |
| *PKM2* | 5 | 4.89 x 10^-1^ | 8.01 x 10^-1^ |
| *PKN1* | 29 | 9.09 x 10^-3^ | 2.36 x 10^-1^ |
| *PLA2G10* | 1 | 4.88 x 10^-1^ | 4.77 x 10^-1^ |
| *PLA2G1B* | 15 | 3.26 x 10^-1^ | 9.18 x 10^-1^ |
| *PLA2G4B* | 13 | 9.20 x 10^-2^ | 9.16 x 10^-1^ |
| *PLA2G6* | 30 | 7.68 x 10^-2^ | 7.22 x 10^-1^ |
| *PLAC8* | 13 | 7.20 x 10^-2^ | 7.87 x 10^-1^ |
| *PLAT* | 13 | 2.63 x 10^-1^ | 1.00 |
| *PLAUR* | 31 | 1.22 x 10^-2^ | 2.45 x 10^-1^ |
| *PLB1* | 76 | 5.29 x 10^-3^ | 2.59 x 10^-1^ |
| *PLCD1* | 19 | 1.45 x 10^-2^ | 2.13 x 10^-1^ |
| *PLCD4* | 9 | 1.45 x 10^-2^ | 1.68 x 10^-2^ |
| *PLCG1* | 11 | 4.30 x 10^-1^ | 9.09 x 10^-1^ |
| *PLCG2* | 120 | 1.45 x 10^-3^ | 1.66 x 10^-1^ |
| *PLD3* | 14 | 8.38 x 10^-2^ | 4.99 x 10^-1^ |
| *PLEC1* | 20 | 1.29 x 10^-2^ | 1.76 x 10^-1^ |
| *PLEK2* | 15 | 6.09 x 10^-2^ | 3.62 x 10^-1^ |
| *PLEKHB2* | 10 | 5.37 x 10^-1^ | 9.96 x 10^-1^ |
| *PLEKHF1* | 24 | 1.24 x 10^-1^ | 7.50 x 10^-1^ |
| *PLEKHJ1* | 17 | 1.01 x 10^-1^ | 4.83 x 10^-1^ |
| *PLEKHO2* | 16 | 2.89 x 10^-1^ | 8.82 x 10^-1^ |
| *PLLP* | 16 | 2.29 x 10^-2^ | 2.00 x 10^-1^ |
| *PLOD1* | 20 | 2.05 x 10^-1^ | 7.75 x 10^-1^ |
| *PLOD3* | 15 | 3.59 x 10^-2^ | 2.45 x 10^-1^ |
| *PLTP* | 25 | 1.40 x 10^-2^ | 1.37 x 10^-1^ |
| *PLUNC* | 22 | 1.28 x 10^-1^ | 4.02 x 10^-1^ |
| *PLVAP* | 20 | 1.51 x 10^-1^ | 9.11 x 10^-1^ |
| *PLXNB2* | 9 | 2.53 x 10^-2^ | 2.16 x 10^-1^ |
| *PLXND1* | 21 | 7.74 x 10^-2^ | 6.39 x 10^-1^ |
| *PMM1* | 7 | 1.80 x 10^-1^ | 7.90 x 10^-1^ |
| *PMP22* | 30 | 6.77 x 10^-3^ | 1.11 x 10^-1^ |
| *PMPCA* | 13 | 1.18 x 10^-2^ | 5.83 x 10^-2^ |
| *PMPCB* | 5 | 1.26 x 10^-1^ | 3.28 x 10^-1^ |
| *PMVK* | 7 | 1.20 x 10^-1^ | 4.02 x 10^-1^ |
| *PNKD* | 24 | 2.24 x 10^-2^ | 5.38 x 10^-1^ |
| *PNPLA2* | 10 | 9.58 x 10^-3^ | 1.57 x 10^-2^ |
| *PNPLA6* | 24 | 1.59 x 10^-1^ | 9.59 x 10^-1^ |
| *PNRC1* | 21 | 2.63 x 10^-3^ | 2.86 x 10^-2^ |
| *PODXL* | 37 | 2.90 x 10^-2^ | 7.36 x 10^-1^ |
| *POGK* | 18 | 7.02 x 10^-2^ | 6.87 x 10^-1^ |
| *POLD2* | 22 | 2.81 x 10^-2^ | 2.73 x 10^-1^ |
| *POLDIP2* | 13 | 1.58 x 10^-1^ | 9.32 x 10^-1^ |
| *POLR1B* | 16 | 1.37 x 10^-2^ | 2.05 x 10^-1^ |
| *POLR1D* | 36 | 8.84 x 10^-3^ | 2.38 x 10^-1^ |
| *POLR2C* | 19 | 3.29 x 10^-2^ | 2.16 x 10^-1^ |
| *POLR2E* | 25 | 4.64 x 10^-2^ | 7.06 x 10^-1^ |
| *POLR2F* | 6 | 5.58 x 10^-1^ | 8.27 x 10^-1^ |
| *POLR2G* | 9 | 5.80 x 10^-2^ | 3.14 x 10^-1^ |
| *POLR2H* | 20 | 2.42 x 10^-3^ | 4.25 x 10^-2^ |
| *POLR2I* | 12 | 9.35 x 10^-2^ | 5.75 x 10^-1^ |
| *POLR2J* | 8 | 2.08 x 10^-1^ | 9.52 x 10^-1^ |
| *POLR2L* | 9 | 9.58 x 10^-3^ | 1.33 x 10^-2^ |
| *POLR3E* | 11 | 4.02 x 10^-3^ | 3.70 x 10^-2^ |
| *POLR3GL* | 8 | 2.92 x 10^-1^ | 7.92 x 10^-1^ |
| *POLR3H* | 11 | 7.88 x 10^-2^ | 8.67 x 10^-1^ |
| *POLS* | 43 | 1.11 x 10^-1^ | 9.94 x 10^-1^ |
| *POMP* | 30 | 1.47 x 10^-3^ | 1.84 x 10^-2^ |
| *PON2* | 40 | 4.10 x 10^-2^ | 3.91 x 10^-1^ |
| *PON3* | 37 | 3.54 x 10^-2^ | 3.50 x 10^-1^ |
| *POP4* | 41 | 9.34 x 10^-2^ | 8.78 x 10^-1^ |
| *POP5* | 18 | 1.40 x 10^-1^ | 4.40 x 10^-1^ |
| *POP7* | 7 | 5.94 x 10^-1^ | 9.74 x 10^-1^ |
| *POPDC2* | 27 | 1.02 x 10^-1^ | 8.05 x 10^-1^ |
| *POR* | 18 | 3.05 x 10^-1^ | 9.12 x 10^-1^ |
| *PPA1* | 30 | 1.31 x 10^-1^ | 5.49 x 10^-1^ |
| *PPAP2A* | 20 | 5.00 x 10^-2^ | 6.43 x 10^-1^ |
| *PPAP2B* | 46 | 1.90 x 10^-2^ | 8.72 x 10^-1^ |
| *PPBP* | 16 | 2.39 x 10^-1^ | 8.30 x 10^-1^ |
| *PPIA* | 7 | 5.30 x 10^-1^ | 9.20 x 10^-1^ |
| *PPIB* | 6 | 2.07 x 10^-1^ | 7.30 x 10^-1^ |
| *PPIC* | 17 | 1.03 x 10^-1^ | 8.32 x 10^-1^ |
| *PPIF* | 24 | 1.78 x 10^-1^ | 9.58 x 10^-1^ |
| *PPL* | 43 | 1.66 x 10^-3^ | 6.97 x 10^-2^ |
| *PPM1F* | 20 | 5.12 x 10^-2^ | 5.61 x 10^-1^ |
| *PPM1G* | 8 | 7.20 x 10^-2^ | 1.41 x 10^-2^ |
| *PPM1L* | 64 | 4.00 x 10^-2^ | 6.97 x 10^-1^ |
| *PPME1* | 18 | 6.91 x 10^-2^ | 6.66 x 10^-1^ |
| *PPP1CA* | 11 | 1.07 x 10^-2^ | 2.37 x 10^-2^ |
| *PPP1CC* | 8 | 3.86 x 10^-1^ | 8.85 x 10^-1^ |
| *PPP1R11* | 55 | 9.81 x 10^-2^ | 9.59 x 10^-1^ |
| *PPP1R13B* | 20 | 1.11 x 10^-2^ | 1.28 x 10^-1^ |
| *PPP1R13L* | 17 | 1.40 x 10^-1^ | 7.49 x 10^-1^ |
| *PPP1R14A* | 12 | 1.50 x 10^-3^ | 7.39 x 10^-3^ |
| *PPP1R14B* | 12 | 6.33 x 10^-2^ | 6.91 x 10^-1^ |
| *PPP1R14C* | 81 | 1.10 x 10^-2^ | 8.90 x 10^-1^ |
| *PPP1R14D* | 13 | 1.14 x 10^-1^ | 5.67 x 10^-1^ |
| *PPP1R15A* | 21 | 1.55 x 10^-2^ | 2.97 x 10^-1^ |
| *PPP1R1B* | 11 | 1.23 x 10^-1^ | 4.23 x 10^-1^ |
| *PPP1R3C* | 31 | 1.73 x 10^-2^ | 2.15 x 10^-1^ |
| *PPP2CA* | 10 | 4.65 x 10^-1^ | 9.75 x 10^-1^ |
| *PPP2CB* | 16 | 9.79 x 10^-3^ | 7.30 x 10^-2^ |
| *PPP2R1A* | 48 | 2.20 x 10^-2^ | 1.00 |
| *PPP2R2A* | 39 | 3.91 x 10^-2^ | 7.75 x 10^-1^ |
| *PPP2R5D* | 17 | 1.37 x 10^-1^ | 5.44 x 10^-1^ |
| *PPP4C* | 8 | 1.84 x 10^-1^ | 9.24 x 10^-1^ |
| *PPP4R1* | 38 | 4.27 x 10^-2^ | 9.13 x 10^-1^ |
| *PPT1* | 30 | 1.73 x 10^-1^ | 9.37 x 10^-1^ |
| *PRB4* | 17 | 1.16 x 10^-2^ | 1.89 x 10^-1^ |
| *PRCP* | 37 | 2.51 x 10^-1^ | 9.52 x 10^-1^ |
| *PRDX1* | 12 | 3.51 x 10^-1^ | 9.18 x 10^-1^ |
| *PRDX2* | 6 | 5.25 x 10^-3^ | 2.60 x 10^-2^ |
| *PRDX5* | 12 | 1.32 x 10^-2^ | 1.00 x 10^-1^ |
| *PRDX6* | 14 | 7.73 x 10^-3^ | 4.23 x 10^-2^ |
| *PREB* | 11 | 3.45 x 10^-2^ | 1.87 x 10^-1^ |
| *PRELID1* | 9 | 2.00 x 10^-1^ | 9.56 x 10^-1^ |
| *PRELP* | 5 | 2.67 x 10^-1^ | 9.55 x 10^-1^ |
| *PRF1* | 35 | 5.64 x 10^-2^ | 7.10 x 10^-1^ |
| *PRG4* | 18 | 2.84 x 10^-2^ | 3.05 x 10^-1^ |
| *PRICKLE1* | 58 | 3.18 x 10^-2^ | 8.81 x 10^-1^ |
| *PRICKLE4* | 10 | 2.76 x 10^-2^ | 2.25 x 10^-1^ |
| *PRKAB1* | 14 | 8.56 x 10^-2^ | 2.29 x 10^-1^ |
| *PRKACA* | 8 | 5.87 x 10^-2^ | 1.10 x 10^-1^ |
| *PRKAG1* | 8 | 1.73 x 10^-1^ | 5.51 x 10^-1^ |
| *PRKAR1A* | 17 | 2.69 x 10^-1^ | 8.82 x 10^-1^ |
| *PRKAR2A* | 7 | 1.77 x 10^-1^ | 3.07 x 10^-1^ |
| *PRKCD* | 27 | 3.23 x 10^-2^ | 5.22 x 10^-1^ |
| *PRKCDBP* | 30 | 7.38 x 10^-2^ | 7.44 x 10^-1^ |
| *PRKCH* | 86 | 7.59 x 10^-2^ | 5.75 x 10^-1^ |
| *PRKCSH* | 11 | 2.82 x 10^-2^ | 2.74 x 10^-1^ |
| *PRKCZ* | 21 | 7.81 x 10^-2^ | 8.79 x 10^-1^ |
| *PRKD2* | 22 | 8.28 x 10^-2^ | 9.36 x 10^-1^ |
| *PRMT1* | 12 | 5.83 x 10^-2^ | 6.81 x 10^-1^ |
| *PRNP* | 42 | 2.30 x 10^-2^ | 7.46 x 10^-1^ |
| *PRODH* | 11 | 2.12 x 10^-2^ | 2.33 x 10^-1^ |
| *PROKR2* | 39 | 1.62 x 10^-2^ | 6.25 x 10^-1^ |
| *ProSAPiP1* | 22 | 8.56 x 10^-4^ | 1.65 x 10^-2^ |
| *PRPF19* | 18 | 1.48 x 10^-1^ | 6.80 x 10^-1^ |
| *PRPF31* | 27 | 3.87 x 10^-4^ | 1.03 x 10^-2^ |
| *PRPF38A* | 6 | 4.06 x 10^-2^ | 2.44 x 10^-1^ |
| *PRPF6* | 18 | 2.51 x 10^-1^ | 8.63 x 10^-1^ |
| *PRPF8* | 20 | 5.23 x 10^-3^ | 6.17 x 10^-2^ |
| *PRPH* | 9 | 1.96 x 10^-1^ | 9.62 x 10^-1^ |
| *PRPH2* | 24 | 5.97 x 10^-2^ | 9.35 x 10^-1^ |
| *PRPSAP1* | 26 | 6.97 x 10^-2^ | 7.02 x 10^-1^ |
| *PRR11* | 9 | 3.45 x 10^-3^ | 1.42 x 10^-2^ |
| *PRR13* | 10 | 2.13 x 10^-1^ | 5.61 x 10^-1^ |
| *PRR14* | 2 | 6.76 x 10^-1^ | 8.67 x 10^-1^ |
| *PRR4* | 47 | 1.32 x 10^-2^ | 5.72 x 10^-1^ |
| *PRSS21* | 15 | 9.05 x 10^-2^ | 8.99 x 10^-1^ |
| *PRSS23* | 24 | 3.33 x 10^-1^ | 9.49 x 10^-1^ |
| *PRSS8* | 7 | 9.82 x 10^-2^ | 1.91 x 10^-1^ |
| *PSAP* | 36 | 6.61 x 10^-2^ | 9.03 x 10^-1^ |
| *PSKH1* | 7 | 6.28 x 10^-1^ | 7.63 x 10^-1^ |
| *PSMA1* | 22 | 2.73 x 10^-2^ | 6.00 x 10^-1^ |
| *PSMA5* | 8 | 5.48 x 10^-2^ | 4.39 x 10^-1^ |
| *PSMA6* | 24 | 2.05 x 10^-2^ | 1.45 x 10^-1^ |
| *PSMA7* | 23 | 1.18 x 10^-1^ | 8.92 x 10^-1^ |
| *PSMB1* | 23 | 1.22 x 10^-2^ | 1.45 x 10^-1^ |
| *PSMB10* | 4 | 6.58 x 10^-1^ | 8.90 x 10^-1^ |
| *PSMB2* | 7 | 1.66 x 10^-1^ | 9.46 x 10^-1^ |
| *PSMB3* | 22 | 5.55 x 10^-2^ | 7.53 x 10^-1^ |
| *PSMB4* | 18 | 2.58 x 10^-2^ | 1.81 x 10^-1^ |
| *PSMB5* | 17 | 7.07 x 10^-2^ | 4.94 x 10^-1^ |
| *PSMB6* | 29 | 7.70 x 10^-3^ | 2.23 x 10^-1^ |
| *PSMB7* | 26 | 6.21 x 10^-2^ | 9.86 x 10^-1^ |
| *PSMB8* | 72 | 2.76 x 10^-2^ | 8.39 x 10^-1^ |
| *PSMB9* | 67 | 2.76 x 10^-2^ | 7.62 x 10^-1^ |
| *PSMC1* | 15 | 2.22 x 10^-2^ | 3.33 x 10^-1^ |
| *PSMC5* | 7 | 4.69 x 10^-2^ | 1.32 x 10^-1^ |
| *PSMD1* | 30 | 2.90 x 10^-2^ | 8.70 x 10^-1^ |
| *PSMD2* | 19 | 2.42 x 10^-3^ | 3.12 x 10^-2^ |
| *PSMD3* | 20 | 7.82 x 10^-3^ | 6.30 x 10^-2^ |
| *PSMD4* | 8 | 6.68 x 10^-3^ | 1.69 x 10^-2^ |
| *PSMD6* | 22 | 2.40 x 10^-1^ | 9.59 x 10^-1^ |
| *PSMD7* | 13 | 1.99 x 10^-2^ | 1.12 x 10^-1^ |
| *PSMD8* | 12 | 7.17 x 10^-3^ | 8.61 x 10^-2^ |
| *PSMD9* | 17 | 6.43 x 10^-3^ | 9.88 x 10^-2^ |
| *PSME1* | 19 | 1.06 x 10^-1^ | 5.08 x 10^-1^ |
| *PSME2* | 19 | 1.06 x 10^-1^ | 5.11 x 10^-1^ |
| *PSME3* | 3 | 5.47 x 10^-1^ | 1.00 |
| *PSMF1* | 46 | 4.69 x 10^-2^ | 9.58 x 10^-1^ |
| *PSMG2* | 21 | 9.10 x 10^-4^ | 1.12 x 10^-2^ |
| *PTBP1* | 24 | 2.79 x 10^-2^ | 6.69 x 10^-1^ |
| *PTCRA* | 20 | 1.37 x 10^-1^ | 4.24 x 10^-1^ |
| *PTDSS1* | 27 | 9.29 x 10^-2^ | 8.29 x 10^-1^ |
| *PTGDS* | 8 | 2.04 x 10^-1^ | 8.70 x 10^-1^ |
| *PTGES3* | 12 | 7.88 x 10^-2^ | 5.53 x 10^-1^ |
| *PTGFRN* | 53 | 3.20 x 10^-2^ | 7.71 x 10^-1^ |
| *PTGIR* | 19 | 8.51 x 10^-2^ | 9.12 x 10^-1^ |
| *PTHR1* | 18 | 2.37 x 10^-2^ | 1.54 x 10^-1^ |
| *PTMA* | 15 | 6.45 x 10^-2^ | 4.55 x 10^-1^ |
| *PTP4A2* | 15 | 6.29 x 10^-2^ | 6.72 x 10^-1^ |
| *PTPN18* | 9 | 1.26 x 10^-1^ | 9.71 x 10^-1^ |
| *PTPN23* | 2 | 1.16 x 10^-1^ | 2.94 x 10^-2^ |
| *PTPN6* | 12 | 3.17 x 10^-2^ | 2.69 x 10^-1^ |
| *PTPRCAP* | 12 | 1.56 x 10^-2^ | 1.59 x 10^-1^ |
| *PTPRE* | 91 | 6.93 x 10^-3^ | 2.98 x 10^-1^ |
| *PTPRF* | 25 | 1.81 x 10^-2^ | 2.50 x 10^-1^ |
| *PTPRK* | 90 | 6.34 x 10^-3^ | 4.96 x 10^-1^ |
| *PTPRM* | 234 | 3.67 x 10^-3^ | 5.75 x 10^-1^ |
| *PTPRU* | 30 | 1.42 x 10^-1^ | 8.80 x 10^-1^ |
| *PTRF* | 13 | 7.58 x 10^-2^ | 5.77 x 10^-1^ |
| *PTTG1IP* | 45 | 4.00 x 10^-3^ | 1.32 x 10^-1^ |
| *PTTG2* | 42 | 4.92 x 10^-2^ | 9.91 x 10^-1^ |
| *PUF60* | 11 | 1.24 x 10^-1^ | 5.77 x 10^-1^ |
| *PUM1* | 22 | 3.16 x 10^-2^ | 2.20 x 10^-1^ |
| *PVRL2* | 22 | 7.24 x 10^-3^ | 1.50 x 10^-1^ |
| *PXDN* | 47 | 9.09 x 10^-3^ | 1.96 x 10^-1^ |
| *PXK* | 25 | 6.98 x 10^-2^ | 5.65 x 10^-1^ |
| *PXMP2* | 12 | 1.14 x 10^-1^ | 8.14 x 10^-1^ |
| *PXMP4* | 9 | 1.33 x 10^-2^ | 8.31 x 10^-2^ |
| *PXN* | 16 | 1.60 x 10^-2^ | 2.40 x 10^-1^ |
| *PYCARD* | 3 | 8.40 x 10^-1^ | 9.27 x 10^-1^ |
| *PYCR2* | 16 | 3.48 x 10^-2^ | 4.73 x 10^-1^ |
| *PZP* | 50 | 2.62 x 10^-2^ | 7.21 x 10^-1^ |
| *QARS* | 4 | 2.20 x 10^-1^ | 5.81 x 10^-1^ |
| *QDPR* | 31 | 4.60 x 10^-2^ | 4.31 x 10^-1^ |
| *QRICH1* | 7 | 2.20 x 10^-1^ | 3.84 x 10^-1^ |
| *QSOX1* | 43 | 6.82 x 10^-3^ | 2.09 x 10^-1^ |
| *R3HDM2* | 9 | 3.06 x 10^-2^ | 2.56 x 10^-1^ |
| *RAB10* | 12 | 6.32 x 10^-3^ | 2.50 x 10^-2^ |
| *RAB11A* | 16 | 1.84 x 10^-1^ | 9.01 x 10^-1^ |
| *RAB11B* | 12 | 2.78 x 10^-2^ | 2.76 x 10^-1^ |
| *RAB11FIP1* | 16 | 8.46 x 10^-2^ | 5.18 x 10^-1^ |
| *RAB11FIP5* | 13 | 2.83 x 10^-1^ | 9.42 x 10^-1^ |
| *RAB13* | 7 | 1.40 x 10^-1^ | 2.77 x 10^-1^ |
| *RAB14* | 16 | 5.60 x 10^-2^ | 6.44 x 10^-1^ |
| *RAB17* | 54 | 1.35 x 10^-1^ | 8.96 x 10^-1^ |
| *RAB1A* | 27 | 4.33 x 10^-2^ | 8.39 x 10^-1^ |
| *RAB1B* | 12 | 1.90 x 10^-1^ | 5.64 x 10^-1^ |
| *RAB20* | 53 | 8.55 x 10^-3^ | 2.77 x 10^-1^ |
| *RAB25* | 12 | 4.57 x 10^-2^ | 2.86 x 10^-1^ |
| *RAB2B* | 31 | 1.41 x 10^-2^ | 2.78 x 10^-1^ |
| *RAB31* | 93 | 5.44 x 10^-3^ | 3.84 x 10^-1^ |
| *RAB32* | 15 | 5.24 x 10^-2^ | 7.86 x 10^-1^ |
| *RAB34* | 6 | 5.27 x 10^-1^ | 7.99 x 10^-1^ |
| *RAB35* | 11 | 5.11 x 10^-2^ | 2.37 x 10^-1^ |
| *RAB36* | 20 | 8.20 x 10^-2^ | 3.32 x 10^-1^ |
| *RAB3GAP1* | 20 | 2.29 x 10^-2^ | 1.84 x 10^-1^ |
| *RAB3IL1* | 24 | 4.42 x 10^-2^ | 4.10 x 10^-1^ |
| *RAB4B* | 18 | 1.07 x 10^-1^ | 7.17 x 10^-1^ |
| *RAB5B* | 16 | 6.61 x 10^-2^ | 6.25 x 10^-1^ |
| *RAB5C* | 10 | 1.56 x 10^-1^ | 8.10 x 10^-1^ |
| *RAB6IP1* | 31 | 6.67 x 10^-2^ | 5.74 x 10^-1^ |
| *RAB7A* | 15 | 1.52 x 10^-3^ | 8.56 x 10^-3^ |
| *RAB8A* | 27 | 3.03 x 10^-2^ | 4.98 x 10^-1^ |
| *RABAC1* | 7 | 3.27 x 10^-1^ | 9.59 x 10^-1^ |
| *RABEP2* | 7 | 1.13 x 10^-1^ | 3.48 x 10^-1^ |
| *RABL4* | 24 | 8.75 x 10^-2^ | 8.25 x 10^-1^ |
| *RAC1* | 21 | 4.28 x 10^-2^ | 3.05 x 10^-1^ |
| *RAC2* | 48 | 1.93 x 10^-2^ | 7.26 x 10^-1^ |
| *RAD1* | 20 | 6.20 x 10^-2^ | 3.66 x 10^-1^ |
| *RAD23A* | 12 | 1.04 x 10^-3^ | 6.95 x 10^-3^ |
| *RAD9A* | 11 | 1.07 x 10^-2^ | 2.37 x 10^-2^ |
| *RAE1* | 24 | 2.94 x 10^-2^ | 4.02 x 10^-1^ |
| *RALB* | 11 | 5.50 x 10^-2^ | 2.90 x 10^-1^ |
| *RALGDS* | 33 | 4.51 x 10^-2^ | 9.05 x 10^-1^ |
| *RALY* | 14 | 2.20 x 10^-1^ | 6.80 x 10^-1^ |
| *RAMP1* | 46 | 6.46 x 10^-2^ | 7.61 x 10^-1^ |
| *RAMP2* | 6 | 2.37 x 10^-1^ | 9.58 x 10^-1^ |
| *RAMP3* | 57 | 9.14 x 10^-3^ | 5.21 x 10^-1^ |
| *RAN* | 14 | 1.73 x 10^-1^ | 9.60 x 10^-1^ |
| *RAP1A* | 47 | 3.37 x 10^-2^ | 7.57 x 10^-1^ |
| *RAP1B* | 9 | 4.19 x 10^-2^ | 9.94 x 10^-2^ |
| *RAP1GAP* | 69 | 9.04 x 10^-3^ | 4.78 x 10^-1^ |
| *RARRES2* | 11 | 1.99 x 10^-2^ | 1.20 x 10^-1^ |
| *RARRES3* | 16 | 1.69 x 10^-2^ | 9.93 x 10^-2^ |
| *RASD1* | 17 | 1.77 x 10^-1^ | 9.70 x 10^-1^ |
| *RASGRP4* | 16 | 7.17 x 10^-3^ | 1.15 x 10^-1^ |
| *RASIP1* | 27 | 4.48 x 10^-2^ | 6.08 x 10^-1^ |
| *RASL12* | 9 | 8.33 x 10^-2^ | 3.47 x 10^-1^ |
| *RASSF1* | 7 | 1.18 x 10^-1^ | 5.09 x 10^-1^ |
| *RASSF5* | 38 | 9.59 x 10^-2^ | 9.64 x 10^-1^ |
| *RASSF7* | 9 | 6.75 x 10^-2^ | 5.66 x 10^-1^ |
| *RBBP4* | 9 | 4.17 x 10^-1^ | 8.70 x 10^-1^ |
| *RBCK1* | 35 | 2.38 x 10^-2^ | 2.61 x 10^-1^ |
| *RBED1* | 22 | 1.60 x 10^-2^ | 1.83 x 10^-1^ |
| *RBM15B* | 16 | 1.60 x 10^-1^ | 5.56 x 10^-1^ |
| *RBM17* | 50 | 3.27 x 10^-2^ | 3.64 x 10^-1^ |
| *RBM23* | 19 | 3.24 x 10^-1^ | 9.16 x 10^-1^ |
| *RBM35B* | 5 | 1.76 x 10^-1^ | 4.67 x 10^-1^ |
| *RBM42* | 15 | 2.76 x 10^-3^ | 1.40 x 10^-2^ |
| *RBM4B* | 4 | 2.41 x 10^-2^ | 8.79 x 10^-2^ |
| *RBM5* | 17 | 1.07 x 10^-1^ | 8.21 x 10^-1^ |
| *RBP1* | 21 | 4.05 x 10^-2^ | 8.50 x 10^-1^ |
| *RBP7* | 8 | 1.95 x 10^-2^ | 1.16 x 10^-1^ |
| *RBPMS* | 35 | 9.76 x 10^-2^ | 4.93 x 10^-1^ |
| *RBX1* | 6 | 4.63 x 10^-2^ | 1.75 x 10^-1^ |
| *RCC2* | 22 | 1.50 x 10^-1^ | 4.83 x 10^-1^ |
| *RDBP* | 23 | 1.94 x 10^-2^ | 2.78 x 10^-1^ |
| *REC8* | 20 | 1.06 x 10^-1^ | 5.85 x 10^-1^ |
| *REEP4* | 19 | 1.02 x 10^-2^ | 6.19 x 10^-2^ |
| *REEP5* | 22 | 1.05 x 10^-3^ | 4.60 x 10^-3^ |
| *RELA* | 11 | 3.56 x 10^-1^ | 8.81 x 10^-1^ |
| *REPIN1* | 15 | 7.99 x 10^-2^ | 3.84 x 10^-1^ |
| *RER1* | 33 | 2.72 x 10^-2^ | 7.69 x 10^-1^ |
| *RETN* | 21 | 3.21 x 10^-2^ | 6.74 x 10^-1^ |
| *RETSAT* | 23 | 1.60 x 10^-2^ | 1.82 x 10^-1^ |
| *REXO2* | 21 | 2.55 x 10^-2^ | 1.83 x 10^-1^ |
| *RFNG* | 9 | 9.84 x 10^-2^ | 4.56 x 10^-1^ |
| *RFTN1* | 89 | 8.18 x 10^-3^ | 4.33 x 10^-1^ |
| *RFXANK* | 15 | 3.73 x 10^-3^ | 5.59 x 10^-2^ |
| *RG9MTD2* | 19 | 5.77 x 10^-2^ | 3.88 x 10^-1^ |
| *RGL1* | 75 | 2.16 x 10^-2^ | 7.24 x 10^-1^ |
| *RGL2* | 14 | 3.08 x 10^-2^ | 1.32 x 10^-1^ |
| *RGMA* | 61 | 7.46 x 10^-2^ | 9.34 x 10^-1^ |
| *RGS19* | 14 | 2.51 x 10^-1^ | 1.00 |
| *RGS3* | 45 | 1.26 x 10^-2^ | 4.96 x 10^-1^ |
| *RHBDD2* | 10 | 3.05 x 10^-1^ | 6.23 x 10^-1^ |
| *RHBDF1* | 14 | 5.48 x 10^-3^ | 6.48 x 10^-2^ |
| *RHEB* | 44 | 4.34 x 10^-2^ | 1.00 |
| *RHOA* | 5 | 4.03 x 10^-1^ | 8.98 x 10^-1^ |
| *RHOB* | 26 | 1.06 x 10^-1^ | 9.37 x 10^-1^ |
| *RHOBTB2* | 23 | 1.20 x 10^-1^ | 9.67 x 10^-1^ |
| *RHOC* | 14 | 8.70 x 10^-2^ | 7.32 x 10^-1^ |
| *RHOD* | 7 | 5.60 x 10^-2^ | 3.86 x 10^-1^ |
| *RHOF* | 17 | 1.93 x 10^-1^ | 9.81 x 10^-1^ |
| *RHOG* | 28 | 1.41 x 10^-2^ | 3.66 x 10^-1^ |
| *RHOT2* | 14 | 1.99 x 10^-1^ | 9.55 x 10^-1^ |
| *RIN2* | 84 | 9.28 x 10^-3^ | 6.09 x 10^-1^ |
| *RIN3* | 88 | 2.06 x 10^-2^ | 6.29 x 10^-1^ |
| *RING1* | 24 | 1.91 x 10^-3^ | 1.72 x 10^-2^ |
| *RIPK4* | 73 | 4.05 x 10^-2^ | 9.41 x 10^-1^ |
| *RNASE1* | 42 | 1.74 x 10^-2^ | 3.34 x 10^-1^ |
| *RNASEH1* | 15 | 3.88 x 10^-2^ | 4.33 x 10^-1^ |
| *RNASEH2C* | 7 | 7.52 x 10^-2^ | 5.27 x 10^-1^ |
| *RNASET2* | 28 | 3.39 x 10^-2^ | 8.16 x 10^-1^ |
| *RNF10* | 21 | 1.40 x 10^-1^ | 3.25 x 10^-1^ |
| *RNF11* | 9 | 3.61 x 10^-1^ | 8.60 x 10^-1^ |
| *RNF112* | 10 | 1.85 x 10^-2^ | 1.85 x 10^-1^ |
| *RNF114* | 19 | 4.44 x 10^-2^ | 4.66 x 10^-1^ |
| *RNF115* | 11 | 1.42 x 10^-1^ | 7.78 x 10^-1^ |
| *RNF122* | 17 | 3.96 x 10^-1^ | 9.61 x 10^-1^ |
| *RNF130* | 43 | 6.65 x 10^-2^ | 5.26 x 10^-1^ |
| *RNF135* | 7 | 1.23 x 10^-1^ | 4.49 x 10^-1^ |
| *RNF145* | 27 | 9.47 x 10^-2^ | 7.49 x 10^-1^ |
| *RNF149* | 16 | 2.59 x 10^-3^ | 2.34 x 10^-2^ |
| *RNF167* | 17 | 8.35 x 10^-3^ | 1.42 x 10^-1^ |
| *RNF19B* | 17 | 4.52 x 10^-1^ | 8.92 x 10^-1^ |
| *RNF34* | 12 | 4.98 x 10^-2^ | 2.78 x 10^-1^ |
| *RNF4* | 17 | 4.16 x 10^-2^ | 5.64 x 10^-1^ |
| *RNF44* | 21 | 1.59 x 10^-2^ | 3.35 x 10^-1^ |
| *RNF5* | 47 | 1.18 x 10^-2^ | 5.41 x 10^-1^ |
| *RNH1* | 9 | 6.61 x 10^-2^ | 3.46 x 10^-1^ |
| *RNPEP* | 29 | 3.06 x 10^-3^ | 6.30 x 10^-2^ |
| *RNPEPL1* | 19 | 1.71 x 10^-2^ | 3.10 x 10^-1^ |
| *RNPS1* | 13 | 6.04 x 10^-2^ | 4.09 x 10^-1^ |
| *RNUXA* | 22 | 3.92 x 10^-2^ | 2.83 x 10^-1^ |
| *ROGDI* | 14 | 2.12 x 10^-2^ | 2.26 x 10^-1^ |
| *ROPN1L* | 20 | 5.76 x 10^-2^ | 3.15 x 10^-1^ |
| *RPA2* | 22 | 2.21 x 10^-1^ | 9.74 x 10^-1^ |
| *RPAIN* | 17 | 7.64 x 10^-3^ | 8.86 x 10^-2^ |
| *RPL10A* | 24 | 1.11 x 10^-2^ | 2.65 x 10^-1^ |
| *RPL11* | 14 | 1.25 x 10^-1^ | 9.62 x 10^-1^ |
| *RPL12* | 16 | 1.05 x 10^-1^ | 5.30 x 10^-1^ |
| *RPL13* | 12 | 3.03 x 10^-2^ | 1.77 x 10^-1^ |
| *RPL13A* | 17 | 2.85 x 10^-2^ | 4.84 x 10^-1^ |
| *RPL14* | 7 | 1.41 x 10^-1^ | 1.33 x 10^-1^ |
| *RPL15* | 15 | 2.45 x 10^-1^ | 8.93 x 10^-1^ |
| *RPL17* | 16 | 3.25 x 10^-1^ | 8.67 x 10^-1^ |
| *RPL18* | 24 | 1.11 x 10^-1^ | 8.49 x 10^-1^ |
| *RPL18A* | 22 | 1.73 x 10^-1^ | 8.24 x 10^-1^ |
| *RPL19* | 22 | 1.12 x 10^-1^ | 8.66 x 10^-1^ |
| *RPL21* | 17 | 1.95 x 10^-2^ | 1.56 x 10^-1^ |
| *RPL22* | 15 | 3.82 x 10^-2^ | 5.73 x 10^-1^ |
| *RPL23* | 22 | 1.02 x 10^-2^ | 1.25 x 10^-1^ |
| *RPL23A* | 7 | 5.27 x 10^-1^ | 8.42 x 10^-1^ |
| *RPL24* | 4 | 1.14 x 10^-1^ | 4.54 x 10^-1^ |
| *RPL27* | 6 | 9.89 x 10^-3^ | 2.04 x 10^-2^ |
| *RPL27A* | 18 | 8.25 x 10^-2^ | 9.44 x 10^-1^ |
| *RPL28* | 18 | 3.93 x 10^-1^ | 9.36 x 10^-1^ |
| *RPL29* | 5 | 1.41 x 10^-1^ | 7.06 x 10^-1^ |
| *RPL3* | 31 | 3.17 x 10^-2^ | 9.83 x 10^-1^ |
| *RPL30* | 30 | 2.48 x 10^-1^ | 9.33 x 10^-1^ |
| *RPL31* | 28 | 5.54 x 10^-2^ | 7.03 x 10^-1^ |
| *RPL32* | 19 | 1.79 x 10^-2^ | 3.40 x 10^-1^ |
| *RPL34* | 8 | 1.21 x 10^-1^ | 2.00 x 10^-1^ |
| *RPL35* | 17 | 4.98 x 10^-3^ | 5.51 x 10^-2^ |
| *RPL35A* | 15 | 1.32 x 10^-2^ | 3.82 x 10^-2^ |
| *RPL36* | 6 | 4.11 x 10^-1^ | 9.72 x 10^-1^ |
| *RPL36AL* | 10 | 6.42 x 10^-2^ | 6.42 x 10^-1^ |
| *RPL37* | 19 | 3.36 x 10^-2^ | 2.75 x 10^-1^ |
| *RPL37A* | 19 | 1.13 x 10^-1^ | 5.19 x 10^-1^ |
| *RPL38* | 25 | 1.17 x 10^-2^ | 2.64 x 10^-1^ |
| *RPL4* | 8 | 2.79 x 10^-1^ | 1.00 |
| *RPL41* | 9 | 1.33 x 10^-1^ | 6.44 x 10^-1^ |
| *RPL5* | 7 | 5.07 x 10^-1^ | 8.11 x 10^-1^ |
| *RPL6* | 3 | 1.24 x 10^-2^ | 3.43 x 10^-2^ |
| *RPL7* | 17 | 5.77 x 10^-2^ | 4.67 x 10^-1^ |
| *RPL7A* | 10 | 2.48 x 10^-1^ | 9.70 x 10^-1^ |
| *RPL7L1* | 14 | 8.24 x 10^-3^ | 9.18 x 10^-2^ |
| *RPL8* | 14 | 5.64 x 10^-2^ | 7.33 x 10^-1^ |
| *RPL9* | 29 | 1.07 x 10^-2^ | 2.44 x 10^-1^ |
| *RPLP0* | 7 | 1.60 x 10^-2^ | 8.04 x 10^-2^ |
| *RPLP1* | 18 | 4.41 x 10^-2^ | 3.13 x 10^-1^ |
| *RPLP2* | 10 | 9.58 x 10^-3^ | 1.58 x 10^-2^ |
| *RPN1* | 13 | 1.24 x 10^-2^ | 9.43 x 10^-2^ |
| *RPN2* | 16 | 2.01 x 10^-2^ | 3.22 x 10^-1^ |
| *RPP21* | 33 | 7.63 x 10^-2^ | 5.18 x 10^-1^ |
| *RPP25* | 9 | 2.41 x 10^-1^ | 8.20 x 10^-1^ |
| *RPP30* | 16 | 9.38 x 10^-2^ | 5.12 x 10^-1^ |
| *RPS10* | 9 | 2.55 x 10^-2^ | 2.21 x 10^-1^ |
| *RPS11* | 15 | 2.85 x 10^-2^ | 4.27 x 10^-1^ |
| *RPS12* | 24 | 7.31 x 10^-2^ | 4.14 x 10^-1^ |
| *RPS13* | 5 | 4.82 x 10^-1^ | 9.42 x 10^-1^ |
| *RPS14* | 15 | 1.40 x 10^-1^ | 6.32 x 10^-1^ |
| *RPS15* | 9 | 8.78 x 10^-2^ | 3.08 x 10^-1^ |
| *RPS15A* | 6 | 1.72 x 10^-1^ | 9.06 x 10^-1^ |
| *RPS16* | 19 | 3.91 x 10^-2^ | 4.38 x 10^-1^ |
| *RPS17* | 13 | 3.01 x 10^-1^ | 1.00 |
| *RPS19* | 10 | 1.00 x 10^-1^ | 2.59 x 10^-1^ |
| *RPS19BP1* | 19 | 1.02 x 10^-1^ | 8.20 x 10^-1^ |
| *RPS2* | 20 | 1.55 x 10^-1^ | 9.64 x 10^-1^ |
| *RPS20* | 9 | 4.29 x 10^-1^ | 9.18 x 10^-1^ |
| *RPS21* | 20 | 1.43 x 10^-1^ | 9.13 x 10^-1^ |
| *RPS23* | 17 | 1.37 x 10^-1^ | 5.09 x 10^-1^ |
| *RPS24* | 10 | 1.74 x 10^-2^ | 1.61 x 10^-1^ |
| *RPS25* | 15 | 9.45 x 10^-2^ | 9.88 x 10^-1^ |
| *RPS26* | 9 | 1.36 x 10^-1^ | 7.52 x 10^-1^ |
| *RPS27* | 7 | 1.40 x 10^-1^ | 2.77 x 10^-1^ |
| *RPS27A* | 19 | 2.70 x 10^-1^ | 1.00 |
| *RPS27L* | 18 | 4.54 x 10^-2^ | 5.03 x 10^-1^ |
| *RPS28* | 14 | 2.78 x 10^-2^ | 1.19 x 10^-1^ |
| *RPS29* | 12 | 1.54 x 10^-1^ | 8.42 x 10^-1^ |
| *RPS3* | 24 | 5.75 x 10^-2^ | 7.67 x 10^-1^ |
| *RPS3A* | 7 | 5.24 x 10^-1^ | 9.72 x 10^-1^ |
| *RPS5* | 18 | 3.20 x 10^-1^ | 8.96 x 10^-1^ |
| *RPS6* | 13 | 3.89 x 10^-1^ | 9.38 x 10^-1^ |
| *RPS6KA1* | 21 | 2.97 x 10^-1^ | 9.52 x 10^-1^ |
| *RPS6KA2* | 213 | 2.05 x 10^-2^ | 6.65 x 10^-1^ |
| *RPS6KA4* | 16 | 4.36 x 10^-3^ | 5.12 x 10^-2^ |
| *RPS7* | 23 | 3.08 x 10^-2^ | 3.37 x 10^-1^ |
| *RPS8* | 13 | 2.23 x 10^-1^ | 8.35 x 10^-1^ |
| *RPS9* | 20 | 2.70 x 10^-1^ | 8.53 x 10^-1^ |
| *RPSA* | 12 | 1.85 x 10^-2^ | 3.66 x 10^-2^ |
| *RPUSD1* | 25 | 6.13 x 10^-2^ | 7.70 x 10^-1^ |
| *RRAGA* | 34 | 3.04 x 10^-3^ | 7.90 x 10^-2^ |
| *RRAGC* | 20 | 6.27 x 10^-2^ | 4.30 x 10^-1^ |
| *RRAS* | 9 | 5.83 x 10^-2^ | 3.65 x 10^-1^ |
| *RRBP1* | 43 | 4.09 x 10^-2^ | 7.30 x 10^-1^ |
| *RSAD1* | 20 | 1.97 x 10^-2^ | 1.92 x 10^-1^ |
| *RSPRY1* | 17 | 5.30 x 10^-2^ | 4.10 x 10^-1^ |
| *RSU1* | 96 | 3.85 x 10^-2^ | 9.78 x 10^-1^ |
| *RTKN* | 6 | 5.45 x 10^-1^ | 7.99 x 10^-1^ |
| *RTN3* | 9 | 1.71 x 10^-1^ | 7.43 x 10^-1^ |
| *RTN4* | 48 | 3.45 x 10^-2^ | 8.71 x 10^-1^ |
| *RUFY1* | 19 | 1.68 x 10^-1^ | 7.03 x 10^-1^ |
| *RUSC1* | 4 | 2.40 x 10^-1^ | 3.08 x 10^-1^ |
| *RUVBL2* | 20 | 5.70 x 10^-3^ | 8.39 x 10^-2^ |
| *RXRA* | 41 | 1.41 x 10^-2^ | 5.77 x 10^-1^ |
| *RYBP* | 29 | 1.23 x 10^-2^ | 3.09 x 10^-1^ |
| *S100A10* | 12 | 3.13 x 10^-2^ | 1.37 x 10^-1^ |
| *S100A11* | 10 | 3.13 x 10^-2^ | 1.47 x 10^-1^ |
| *S100A13* | 6 | 6.40 x 10^-1^ | 9.26 x 10^-1^ |
| *S100A14* | 4 | 7.08 x 10^-1^ | 9.26 x 10^-1^ |
| *S100A16* | 4 | 7.08 x 10^-1^ | 9.26 x 10^-1^ |
| *S100A2* | 1 | 2.19 x 10^-1^ | 9.58 x 10^-2^ |
| *S100A4* | 2 | 2.19 x 10^-1^ | 1.70 x 10^-1^ |
| *S100A6* | 3 | 2.19 x 10^-1^ | 1.76 x 10^-1^ |
| *S100A8* | 20 | 2.13 x 10^-2^ | 2.00 x 10^-1^ |
| *S100A9* | 23 | 2.13 x 10^-2^ | 2.96 x 10^-1^ |
| *S100P* | 30 | 1.94 x 10^-1^ | 9.33 x 10^-1^ |
| *S1PR1* | 31 | 8.07 x 10^-3^ | 2.49 x 10^-1^ |
| *S1PR4* | 24 | 1.66 x 10^-1^ | 9.32 x 10^-1^ |
| *SAE1* | 15 | 4.49 x 10^-2^ | 2.96 x 10^-1^ |
| *SALL2* | 43 | 3.40 x 10^-3^ | 1.28 x 10^-1^ |
| *SAP18* | 32 | 4.58 x 10^-2^ | 6.83 x 10^-1^ |
| *SAPS1* | 25 | 3.91 x 10^-2^ | 3.48 x 10^-1^ |
| *SAPS3* | 25 | 4.57 x 10^-2^ | 8.90 x 10^-1^ |
| *SARS* | 34 | 1.11 x 10^-1^ | 8.81 x 10^-1^ |
| *SARS2* | 17 | 1.12 x 10^-1^ | 9.49 x 10^-1^ |
| *SART1* | 17 | 4.44 x 10^-2^ | 2.59 x 10^-1^ |
| *SASH1* | 117 | 1.48 x 10^-2^ | 6.38 x 10^-1^ |
| *SATB1* | 28 | 7.93 x 10^-3^ | 1.04 x 10^-1^ |
| *SC4MOL* | 21 | 1.17 x 10^-1^ | 7.80 x 10^-1^ |
| *SCAMP2* | 14 | 9.49 x 10^-2^ | 5.75 x 10^-1^ |
| *SCAMP3* | 4 | 2.76 x 10^-1^ | 5.05 x 10^-1^ |
| *SCAMP5* | 17 | 1.35 x 10^-1^ | 9.37 x 10^-1^ |
| *SCAND1* | 9 | 4.49 x 10^-1^ | 7.74 x 10^-1^ |
| *SCAP* | 5 | 1.16 x 10^-1^ | 8.14 x 10^-2^ |
| *SCARF1* | 18 | 2.87 x 10^-2^ | 2.15 x 10^-1^ |
| *SCGB1A1* | 35 | 3.30 x 10^-2^ | 5.74 x 10^-1^ |
| *SCMH1* | 30 | 1.00 x 10^-1^ | 7.85 x 10^-1^ |
| *SCN10A* | 47 | 1.59 x 10^-2^ | 7.49 x 10^-1^ |
| *SCNM1* | 13 | 9.45 x 10^-2^ | 4.72 x 10^-1^ |
| *SCNN1A* | 29 | 1.77 x 10^-1^ | 9.10 x 10^-1^ |
| *SCNN1B* | 28 | 6.60 x 10^-2^ | 7.83 x 10^-1^ |
| *SCPEP1* | 22 | 1.73 x 10^-3^ | 1.78 x 10^-2^ |
| *SCRIB* | 10 | 2.30 x 10^-1^ | 5.30 x 10^-1^ |
| *SCYL1* | 10 | 5.82 x 10^-2^ | 5.82 x 10^-1^ |
| *SDAD1* | 24 | 3.99 x 10^-2^ | 5.44 x 10^-1^ |
| *SDC1* | 31 | 9.38 x 10^-2^ | 8.04 x 10^-1^ |
| *SDC4* | 30 | 4.46 x 10^-2^ | 5.73 x 10^-1^ |
| *SDCBP* | 17 | 2.58 x 10^-1^ | 1.00 |
| *SDF2* | 3 | 7.12 x 10^-1^ | 8.21 x 10^-1^ |
| *SDF2L1* | 16 | 1.45 x 10^-2^ | 1.37 x 10^-1^ |
| *SDF4* | 14 | 1.62 x 10^-1^ | 9.06 x 10^-1^ |
| *SDHA* | 20 | 6.63 x 10^-3^ | 1.33 x 10^-1^ |
| *SDHC* | 29 | 6.00 x 10^-2^ | 9.39 x 10^-1^ |
| *SEC11A* | 17 | 3.86 x 10^-2^ | 5.50 x 10^-1^ |
| *SEC13* | 31 | 3.51 x 10^-1^ | 1.00 |
| *SEC14L1* | 41 | 2.84 x 10^-2^ | 9.11 x 10^-1^ |
| *SEC16A* | 22 | 1.18 x 10^-2^ | 1.62 x 10^-1^ |
| *SEC24C* | 9 | 4.73 x 10^-1^ | 9.31 x 10^-1^ |
| *SEC31A* | 12 | 4.58 x 10^-1^ | 9.19 x 10^-1^ |
| *SEC61A1* | 10 | 2.92 x 10^-1^ | 9.58 x 10^-1^ |
| *SEC61B* | 14 | 9.78 x 10^-2^ | 9.58 x 10^-1^ |
| *SEC61G* | 21 | 2.17 x 10^-1^ | 8.48 x 10^-1^ |
| *SECTM1* | 9 | 3.87 x 10^-1^ | 9.86 x 10^-1^ |
| *SELENBP1* | 17 | 2.18 x 10^-2^ | 9.56 x 10^-2^ |
| *SELO* | 20 | 2.53 x 10^-2^ | 5.07 x 10^-1^ |
| *SELP* | 61 | 9.67 x 10^-2^ | 7.91 x 10^-1^ |
| *SELPLG* | 19 | 7.45 x 10^-3^ | 4.33 x 10^-2^ |
| *SELS* | 27 | 1.51 x 10^-2^ | 4.09 x 10^-1^ |
| *SELT* | 22 | 8.04 x 10^-2^ | 8.87 x 10^-1^ |
| *SEMA3F* | 13 | 2.22 x 10^-1^ | 8.37 x 10^-1^ |
| *SEMA4A* | 17 | 8.48 x 10^-3^ | 9.11 x 10^-2^ |
| *SEMA4B* | 23 | 2.33 x 10^-1^ | 9.59 x 10^-1^ |
| *SEMA4C* | 13 | 3.09 x 10^-2^ | 1.22 x 10^-1^ |
| *SEPHS2* | 9 | 1.62 x 10^-1^ | 9.88 x 10^-1^ |
| *SEPN1* | 13 | 3.37 x 10^-2^ | 2.04 x 10^-1^ |
| *SEPP1* | 11 | 8.39 x 10^-2^ | 8.40 x 10^-1^ |
| *SEPT2* | 16 | 2.62 x 10^-1^ | 8.75 x 10^-1^ |
| *SEPT9* | 89 | 5.92 x 10^-2^ | 9.42 x 10^-1^ |
| *SEPW1* | 24 | 3.34 x 10^-2^ | 5.85 x 10^-1^ |
| *SEPX1* | 20 | 1.55 x 10^-1^ | 9.44 x 10^-1^ |
| *SERBP1* | 17 | 9.05 x 10^-3^ | 5.74 x 10^-2^ |
| *SERF2* | 6 | 8.27 x 10^-2^ | 4.96 x 10^-1^ |
| *SERINC3* | 14 | 3.18 x 10^-2^ | 2.60 x 10^-1^ |
| *SERP1* | 19 | 2.44 x 10^-1^ | 8.61 x 10^-1^ |
| *SERPINA1* | 53 | 1.45 x 10^-2^ | 1.47 x 10^-1^ |
| *SERPINA3* | 63 | 1.06 x 10^-2^ | 1.92 x 10^-1^ |
| *SERPINB1* | 40 | 2.39 x 10^-3^ | 9.58 x 10^-2^ |
| *SERPINB6* | 40 | 1.64 x 10^-1^ | 9.30 x 10^-1^ |
| *SERPINE1* | 18 | 4.09 x 10^-2^ | 2.13 x 10^-1^ |
| *SERPINF1* | 26 | 4.67 x 10^-3^ | 3.96 x 10^-2^ |
| *SERPING1* | 20 | 3.50 x 10^-2^ | 4.16 x 10^-1^ |
| *SERTAD1* | 9 | 5.27 x 10^-3^ | 3.91 x 10^-2^ |
| *SESN1* | 20 | 3.10 x 10^-2^ | 4.96 x 10^-1^ |
| *SET* | 3 | 9.46 x 10^-2^ | 2.74 x 10^-1^ |
| *SETD5* | 24 | 1.59 x 10^-1^ | 1.00 |
| *SF1* | 11 | 1.41 x 10^-1^ | 6.45 x 10^-1^ |
| *SF3A1* | 23 | 1.51 x 10^-2^ | 2.63 x 10^-1^ |
| *SF3A2* | 14 | 1.01 x 10^-1^ | 6.11 x 10^-1^ |
| *SF3B1* | 7 | 3.07 x 10^-1^ | 7.61 x 10^-1^ |
| *SF3B4* | 9 | 2.27 x 10^-1^ | 7.15 x 10^-1^ |
| *SF3B5* | 27 | 4.82 x 10^-3^ | 1.30 x 10^-1^ |
| *SFN* | 5 | 4.69 x 10^-1^ | 9.23 x 10^-1^ |
| *SFRP2* | 15 | 8.33 x 10^-2^ | 5.02 x 10^-1^ |
| *SFRS16* | 11 | 4.13 x 10^-2^ | 4.41 x 10^-1^ |
| *SFRS2* | 18 | 2.88 x 10^-3^ | 4.01 x 10^-2^ |
| *SFRS2B* | 26 | 1.03 x 10^-2^ | 2.39 x 10^-1^ |
| *SFRS3* | 12 | 1.93 x 10^-1^ | 7.59 x 10^-1^ |
| *SFRS4* | 14 | 4.02 x 10^-1^ | 9.13 x 10^-1^ |
| *SFRS5* | 13 | 4.35 x 10^-2^ | 5.33 x 10^-1^ |
| *SFRS7* | 15 | 9.17 x 10^-2^ | 7.00 x 10^-1^ |
| *SFRS8* | 26 | 2.98 x 10^-2^ | 5.20 x 10^-1^ |
| *SFRS9* | 11 | 9.48 x 10^-2^ | 5.61 x 10^-1^ |
| *SFT2D1* | 21 | 1.10 x 10^-1^ | 4.82 x 10^-1^ |
| *SFTPB* | 20 | 2.99 x 10^-2^ | 5.97 x 10^-1^ |
| *SFTPC* | 21 | 1.02 x 10^-2^ | 8.71 x 10^-2^ |
| *SFTPD* | 19 | 4.07 x 10^-3^ | 6.58 x 10^-2^ |
| *SGK1* | 22 | 2.15 x 10^-2^ | 3.86 x 10^-1^ |
| *SGSH* | 23 | 1.56 x 10^-1^ | 8.80 x 10^-1^ |
| *SGSM2* | 24 | 2.56 x 10^-1^ | 9.85 x 10^-1^ |
| *SH2B2* | 13 | 1.05 x 10^-2^ | 1.07 x 10^-1^ |
| *SH2B3* | 7 | 2.55 x 10^-1^ | 1.00 |
| *SH2D1B* | 36 | 9.97 x 10^-3^ | 3.50 x 10^-1^ |
| *SH2D3C* | 16 | 1.17 x 10^-1^ | 8.92 x 10^-1^ |
| *SH3BGRL3* | 20 | 6.26 x 10^-2^ | 2.84 x 10^-1^ |
| *SH3BP4* | 55 | 1.79 x 10^-2^ | 4.32 x 10^-1^ |
| *SH3BP5* | 42 | 2.06 x 10^-2^ | 5.15 x 10^-1^ |
| *SH3GLB1* | 10 | 1.19 x 10^-1^ | 5.45 x 10^-1^ |
| *SH3TC1* | 25 | 5.70 x 10^-2^ | 8.01 x 10^-1^ |
| *SHC1* | 9 | 1.94 x 10^-2^ | 4.38 x 10^-2^ |
| *SHC2* | 32 | 2.26 x 10^-2^ | 7.24 x 10^-1^ |
| *SHISA5* | 9 | 5.41 x 10^-1^ | 1.00 |
| *SIGIRR* | 17 | 1.63 x 10^-2^ | 1.39 x 10^-1^ |
| *SIGLEC11* | 22 | 3.32 x 10^-2^ | 1.84 x 10^-1^ |
| *SIL1* | 27 | 1.27 x 10^-2^ | 3.41 x 10^-2^ |
| *SIRT2* | 15 | 2.99 x 10^-1^ | 8.75 x 10^-1^ |
| *SIRT3* | 42 | 1.08 x 10^-1^ | 8.43 x 10^-1^ |
| *SIRT6* | 21 | 1.79 x 10^-2^ | 2.21 x 10^-1^ |
| *SIVA1* | 8 | 6.64 x 10^-2^ | 3.06 x 10^-1^ |
| *SKAP1* | 39 | 1.24 x 10^-1^ | 9.23 x 10^-1^ |
| *SKIP* | 24 | 5.62 x 10^-2^ | 4.67 x 10^-1^ |
| *SKIV2L* | 21 | 1.94 x 10^-2^ | 2.97 x 10^-1^ |
| *SKP1* | 14 | 4.65 x 10^-1^ | 8.45 x 10^-1^ |
| *SLA* | 52 | 8.50 x 10^-3^ | 9.24 x 10^-2^ |
| *SLA2* | 9 | 6.23 x 10^-2^ | 2.69 x 10^-1^ |
| *SLAMF6* | 25 | 1.75 x 10^-1^ | 9.15 x 10^-1^ |
| *SLBP* | 12 | 1.00 x 10^-1^ | 8.56 x 10^-1^ |
| *SLC11A1* | 18 | 2.24 x 10^-2^ | 4.04 x 10^-1^ |
| *SLC12A7* | 19 | 1.96 x 10^-2^ | 3.32 x 10^-1^ |
| *SLC15A3* | 26 | 2.54 x 10^-2^ | 3.64 x 10^-1^ |
| *SLC16A3* | 9 | 1.76 x 10^-1^ | 4.79 x 10^-1^ |
| *SLC17A5* | 26 | 3.71 x 10^-2^ | 6.27 x 10^-1^ |
| *SLC17A8* | 20 | 1.81 x 10^-1^ | 9.68 x 10^-1^ |
| *SLC22A14* | 18 | 2.02 x 10^-1^ | 8.34 x 10^-1^ |
| *SLC22A18* | 30 | 3.58 x 10^-2^ | 3.60 x 10^-1^ |
| *SLC22A23* | 92 | 1.38 x 10^-2^ | 6.03 x 10^-1^ |
| *SLC23A1* | 6 | 1.09 x 10^-1^ | 4.31 x 10^-1^ |
| *SLC25A1* | 25 | 3.43 x 10^-2^ | 4.69 x 10^-1^ |
| *SLC25A11* | 17 | 8.35 x 10^-3^ | 1.42 x 10^-1^ |
| *SLC25A2* | 20 | 1.37 x 10^-1^ | 9.55 x 10^-1^ |
| *SLC25A20* | 3 | 1.77 x 10^-1^ | 2.06 x 10^-1^ |
| *SLC25A29* | 10 | 1.66 x 10^-1^ | 9.61 x 10^-1^ |
| *SLC25A3* | 22 | 1.56 x 10^-1^ | 8.69 x 10^-1^ |
| *SLC25A36* | 14 | 2.00 x 10^-1^ | 9.29 x 10^-1^ |
| *SLC25A38* | 11 | 1.85 x 10^-2^ | 5.64 x 10^-2^ |
| *SLC25A39* | 12 | 1.99 x 10^-1^ | 8.05 x 10^-1^ |
| *SLC25A4* | 20 | 2.10 x 10^-1^ | 1.00 |
| *SLC27A3* | 22 | 1.05 x 10^-1^ | 9.44 x 10^-1^ |
| *SLC29A3* | 47 | 2.26 x 10^-2^ | 3.31 x 10^-1^ |
| *SLC2A14* | 25 | 3.32 x 10^-2^ | 2.89 x 10^-1^ |
| *SLC2A3* | 6 | 1.08 x 10^-1^ | 1.76 x 10^-1^ |
| *SLC2A4RG* | 18 | 7.14 x 10^-3^ | 7.90 x 10^-2^ |
| *SLC2A9* | 80 | 4.36 x 10^-2^ | 8.34 x 10^-1^ |
| *SLC31A2* | 40 | 1.47 x 10^-2^ | 5.30 x 10^-1^ |
| *SLC34A2* | 23 | 4.18 x 10^-2^ | 6.16 x 10^-1^ |
| *SLC35A4* | 8 | 5.25 x 10^-1^ | 8.75 x 10^-1^ |
| *SLC35B1* | 9 | 5.61 x 10^-2^ | 2.48 x 10^-1^ |
| *SLC35B2* | 19 | 2.00 x 10^-1^ | 9.26 x 10^-1^ |
| *SLC35C1* | 20 | 4.70 x 10^-2^ | 3.93 x 10^-1^ |
| *SLC35E1* | 14 | 2.88 x 10^-2^ | 1.28 x 10^-1^ |
| *SLC35E2* | 3 | 2.43 x 10^-1^ | 6.84 x 10^-1^ |
| *SLC37A2* | 35 | 8.39 x 10^-3^ | 2.72 x 10^-1^ |
| *SLC38A10* | 20 | 2.41 x 10^-2^ | 2.90 x 10^-1^ |
| *SLC39A1* | 5 | 2.84 x 10^-1^ | 5.90 x 10^-1^ |
| *SLC39A3* | 15 | 2.07 x 10^-1^ | 9.88 x 10^-1^ |
| *SLC39A4* | 21 | 4.12 x 10^-2^ | 1.36 x 10^-1^ |
| *SLC3A2* | 16 | 5.80 x 10^-2^ | 7.23 x 10^-1^ |
| *SLC40A1* | 30 | 1.39 x 10^-1^ | 8.92 x 10^-1^ |
| *SLC41A3* | 35 | 1.27 x 10^-1^ | 6.34 x 10^-1^ |
| *SLC43A3* | 21 | 1.38 x 10^-2^ | 2.91 x 10^-1^ |
| *SLC44A2* | 6 | 1.74 x 10^-1^ | 1.95 x 10^-1^ |
| *SLC45A4* | 41 | 3.66 x 10^-4^ | 1.16 x 10^-2^ |
| *SLC5A5* | 24 | 4.75 x 10^-3^ | 5.73 x 10^-2^ |
| *SLC6A12* | 46 | 2.74 x 10^-2^ | 4.03 x 10^-1^ |
| *SLC6A13* | 34 | 2.74 x 10^-2^ | 2.36 x 10^-1^ |
| *SLC6A4* | 18 | 1.99 x 10^-2^ | 3.57 x 10^-1^ |
| *SLC7A7* | 49 | 3.76 x 10^-2^ | 9.33 x 10^-1^ |
| *SLC9A1* | 11 | 2.80 x 10^-1^ | 9.39 x 10^-1^ |
| *SLC9A3R1* | 24 | 8.19 x 10^-2^ | 9.70 x 10^-1^ |
| *SLCO2A1* | 55 | 5.24 x 10^-3^ | 2.02 x 10^-1^ |
| *SLCO2B1* | 29 | 4.35 x 10^-2^ | 8.38 x 10^-1^ |
| *SLCO3A1* | 127 | 3.24 x 10^-2^ | 9.83 x 10^-1^ |
| *SLCO4A1* | 37 | 1.87 x 10^-2^ | 2.47 x 10^-1^ |
| *SLIT2* | 115 | 1.51 x 10^-3^ | 8.71 x 10^-2^ |
| *SLMAP* | 25 | 3.47 x 10^-2^ | 2.87 x 10^-1^ |
| *SLN* | 28 | 1.49 x 10^-2^ | 2.01 x 10^-1^ |
| *SLPI* | 18 | 9.21 x 10^-2^ | 9.06 x 10^-1^ |
| *SMAD6* | 52 | 3.66 x 10^-3^ | 1.25 x 10^-1^ |
| *SMAD7* | 31 | 6.12 x 10^-2^ | 4.63 x 10^-1^ |
| *SMARCA4* | 23 | 3.30 x 10^-2^ | 5.59 x 10^-1^ |
| *SMARCC2* | 8 | 8.79 x 10^-2^ | 3.37 x 10^-1^ |
| *SMARCD2* | 7 | 4.69 x 10^-2^ | 1.32 x 10^-1^ |
| *SMARCD3* | 32 | 2.55 x 10^-2^ | 7.91 x 10^-1^ |
| *SMCR7L* | 21 | 6.77 x 10^-2^ | 6.71 x 10^-1^ |
| *SMPD4* | 2 | 4.79 x 10^-2^ | 8.43 x 10^-3^ |
| *SMTN* | 30 | 2.10 x 10^-1^ | 1.00 |
| *SND1* | 55 | 3.82 x 10^-3^ | 2.04 x 10^-1^ |
| *SNF1LK* | 39 | 1.77 x 10^-2^ | 6.89 x 10^-1^ |
| *SNF8* | 10 | 7.00 x 10^-3^ | 2.06 x 10^-2^ |
| *SNN* | 21 | 8.59 x 10^-2^ | 9.96 x 10^-1^ |
| *SNRP70* | 25 | 1.93 x 10^-2^ | 4.82 x 10^-1^ |
| *SNRPA* | 20 | 1.07 x 10^-1^ | 7.96 x 10^-1^ |
| *SNRPB* | 36 | 4.40 x 10^-2^ | 7.59 x 10^-1^ |
| *SNRPC* | 8 | 1.98 x 10^-4^ | 1.49 x 10^-4^ |
| *SNRPD2* | 17 | 4.31 x 10^-2^ | 7.23 x 10^-1^ |
| *SNRPF* | 35 | 8.51 x 10^-2^ | 8.48 x 10^-1^ |
| *SNRPG* | 16 | 1.22 x 10^-1^ | 9.15 x 10^-1^ |
| *SNRPN* | 78 | 9.55 x 10^-3^ | 6.97 x 10^-1^ |
| *SNTA1* | 6 | 5.45 x 10^-2^ | 3.27 x 10^-1^ |
| *SNUPN* | 14 | 1.11 x 10^-1^ | 6.34 x 10^-1^ |
| *SNX1* | 8 | 2.07 x 10^-1^ | 6.34 x 10^-1^ |
| *SNX11* | 9 | 3.83 x 10^-1^ | 7.19 x 10^-1^ |
| *SNX15* | 17 | 3.24 x 10^-2^ | 5.51 x 10^-1^ |
| *SNX17* | 3 | 7.20 x 10^-2^ | 2.55 x 10^-2^ |
| *SNX22* | 5 | 2.07 x 10^-1^ | 6.01 x 10^-1^ |
| *SNX27* | 23 | 4.33 x 10^-2^ | 9.11 x 10^-1^ |
| *SNX3* | 20 | 2.53 x 10^-1^ | 9.30 x 10^-1^ |
| *SNX33* | 10 | 1.11 x 10^-1^ | 3.90 x 10^-1^ |
| *SNX5* | 34 | 3.24 x 10^-2^ | 4.62 x 10^-1^ |
| *SNX9* | 45 | 1.49 x 10^-2^ | 1.26 x 10^-1^ |
| *SOCS2* | 23 | 2.03 x 10^-1^ | 8.90 x 10^-1^ |
| *SOCS4* | 9 | 3.15 x 10^-1^ | 6.82 x 10^-1^ |
| *SOD1* | 17 | 1.67 x 10^-2^ | 8.92 x 10^-2^ |
| *SOD3* | 25 | 8.00 x 10^-3^ | 1.62 x 10^-1^ |
| *SON* | 9 | 2.30 x 10^-1^ | 7.23 x 10^-1^ |
| *SORBS2* | 172 | 1.73 x 10^-3^ | 2.73 x 10^-1^ |
| *SORBS3* | 17 | 1.09 x 10^-1^ | 7.55 x 10^-1^ |
| *SORL1* | 50 | 2.70 x 10^-3^ | 1.02 x 10^-1^ |
| *SOX13* | 51 | 1.36 x 10^-1^ | 9.65 x 10^-1^ |
| *SOX17* | 23 | 2.59 x 10^-1^ | 9.61 x 10^-1^ |
| *SPAG7* | 16 | 1.81 x 10^-1^ | 9.21 x 10^-1^ |
| *SPARC* | 33 | 1.63 x 10^-2^ | 3.77 x 10^-1^ |
| *SPARCL1* | 35 | 8.59 x 10^-2^ | 3.95 x 10^-1^ |
| *SPATA20* | 26 | 4.34 x 10^-2^ | 6.35 x 10^-1^ |
| *SPCS1* | 6 | 2.86 x 10^-1^ | 8.33 x 10^-1^ |
| *SPCS2* | 13 | 4.92 x 10^-1^ | 9.57 x 10^-1^ |
| *SPEN* | 21 | 5.59 x 10^-4^ | 1.17 x 10^-2^ |
| *SPG21* | 14 | 9.62 x 10^-2^ | 8.96 x 10^-1^ |
| *SPG7* | 16 | 3.03 x 10^-2^ | 2.58 x 10^-1^ |
| *SPINT1* | 15 | 1.14 x 10^-1^ | 6.72 x 10^-1^ |
| *SPINT2* | 14 | 1.50 x 10^-3^ | 9.19 x 10^-3^ |
| *SPIRE2* | 16 | 8.35 x 10^-2^ | 9.90 x 10^-1^ |
| *SPNS1* | 14 | 3.28 x 10^-2^ | 3.55 x 10^-1^ |
| *SPOCK2* | 25 | 1.09 x 10^-1^ | 9.69 x 10^-1^ |
| *SPON2* | 15 | 2.11 x 10^-1^ | 8.90 x 10^-1^ |
| *SPOP* | 19 | 4.06 x 10^-2^ | 2.33 x 10^-1^ |
| *SPPL2A* | 19 | 1.73 x 10^-1^ | 6.28 x 10^-1^ |
| *SPR* | 14 | 1.82 x 10^-1^ | 8.58 x 10^-1^ |
| *SPRN* | 14 | 1.04 x 10^-1^ | 8.63 x 10^-1^ |
| *SPRY1* | 16 | 1.66 x 10^-1^ | 8.09 x 10^-1^ |
| *SPRY2* | 22 | 3.71 x 10^-3^ | 4.22 x 10^-2^ |
| *SPSB2* | 18 | 1.97 x 10^-2^ | 1.53 x 10^-1^ |
| *SPTAN1* | 10 | 1.41 x 10^-1^ | 7.42 x 10^-1^ |
| *SPTBN1* | 61 | 4.59 x 10^-3^ | 2.22 x 10^-1^ |
| *SPTBN5* | 41 | 8.22 x 10^-2^ | 1.00 |
| *SQLE* | 23 | 1.03 x 10^-1^ | 7.57 x 10^-1^ |
| *SQRDL* | 48 | 2.98 x 10^-3^ | 1.23 x 10^-1^ |
| *SQSTM1* | 17 | 1.44 x 10^-1^ | 9.03 x 10^-1^ |
| *SREBF1* | 10 | 7.64 x 10^-2^ | 2.85 x 10^-1^ |
| *SREBF2* | 26 | 2.38 x 10^-1^ | 1.00 |
| *SRGN* | 27 | 1.92 x 10^-2^ | 4.41 x 10^-1^ |
| *SRI* | 14 | 5.62 x 10^-2^ | 7.87 x 10^-1^ |
| *SRM* | 11 | 1.98 x 10^-1^ | 8.03 x 10^-1^ |
| *SRP14* | 31 | 1.49 x 10^-2^ | 2.97 x 10^-1^ |
| *SRP68* | 17 | 6.97 x 10^-2^ | 6.72 x 10^-1^ |
| *SRP9* | 27 | 4.30 x 10^-3^ | 4.99 x 10^-2^ |
| *SRPR* | 22 | 1.14 x 10^-1^ | 9.15 x 10^-1^ |
| *SRPRB* | 29 | 1.56 x 10^-2^ | 2.82 x 10^-1^ |
| *SRRM1* | 11 | 4.16 x 10^-1^ | 9.46 x 10^-1^ |
| *SRRM2* | 10 | 3.70 x 10^-2^ | 3.55 x 10^-1^ |
| *SRXN1* | 42 | 1.52 x 10^-1^ | 9.21 x 10^-1^ |
| *SSBP4* | 13 | 7.56 x 10^-2^ | 2.94 x 10^-1^ |
| *SSH1* | 21 | 5.72 x 10^-2^ | 4.87 x 10^-1^ |
| *SSNA1* | 2 | 2.99 x 10^-1^ | 5.98 x 10^-1^ |
| *SSR2* | 9 | 3.22 x 10^-1^ | 7.14 x 10^-1^ |
| *SSU72* | 7 | 1.81 x 10^-1^ | 6.03 x 10^-1^ |
| *ST13* | 12 | 4.63 x 10^-2^ | 4.30 x 10^-1^ |
| *ST3GAL5* | 35 | 2.15 x 10^-2^ | 2.30 x 10^-1^ |
| *ST6GALNAC2* | 24 | 5.50 x 10^-3^ | 1.16 x 10^-1^ |
| *ST6GALNAC3* | 194 | 3.72 x 10^-2^ | 9.54 x 10^-1^ |
| *ST6GALNAC4* | 17 | 3.49 x 10^-2^ | 4.70 x 10^-1^ |
| *STAB1* | 10 | 3.75 x 10^-1^ | 1.00 |
| *STAC* | 56 | 3.37 x 10^-3^ | 1.11 x 10^-1^ |
| *STAP2* | 22 | 9.39 x 10^-2^ | 7.05 x 10^-1^ |
| *STARD10* | 21 | 6.36 x 10^-2^ | 4.72 x 10^-1^ |
| *STARD3* | 12 | 1.23 x 10^-1^ | 3.81 x 10^-1^ |
| *STARD3NL* | 23 | 1.64 x 10^-2^ | 7.76 x 10^-2^ |
| *STARD7* | 10 | 6.09 x 10^-3^ | 4.04 x 10^-2^ |
| *STAT3* | 16 | 5.92 x 10^-3^ | 7.07 x 10^-2^ |
| *STAT5A* | 8 | 5.68 x 10^-3^ | 1.08 x 10^-2^ |
| *STAT5B* | 9 | 5.68 x 10^-3^ | 1.12 x 10^-2^ |
| *STAT6* | 17 | 2.37 x 10^-2^ | 9.69 x 10^-2^ |
| *STAU1* | 14 | 5.59 x 10^-2^ | 6.17 x 10^-1^ |
| *STEAP3* | 36 | 5.63 x 10^-2^ | 1.00 |
| *STK10* | 69 | 1.55 x 10^-2^ | 4.90 x 10^-1^ |
| *STK11* | 11 | 3.27 x 10^-1^ | 9.00 x 10^-1^ |
| *STK19* | 25 | 1.94 x 10^-2^ | 3.54 x 10^-1^ |
| *STK24* | 80 | 3.71 x 10^-3^ | 1.58 x 10^-1^ |
| *STK25* | 20 | 4.95 x 10^-2^ | 5.35 x 10^-1^ |
| *STK32A* | 77 | 1.77 x 10^-2^ | 8.70 x 10^-1^ |
| *STK36* | 11 | 1.45 x 10^-2^ | 2.86 x 10^-2^ |
| *STK40* | 23 | 3.07 x 10^-1^ | 9.60 x 10^-1^ |
| *STOM* | 20 | 7.82 x 10^-2^ | 4.56 x 10^-1^ |
| *STOML2* | 13 | 1.88 x 10^-3^ | 1.75 x 10^-2^ |
| *STRA13* | 7 | 9.84 x 10^-2^ | 3.67 x 10^-1^ |
| *STRAP* | 7 | 4.19 x 10^-2^ | 1.44 x 10^-1^ |
| *STUB1* | 13 | 1.99 x 10^-1^ | 9.80 x 10^-1^ |
| *STX10* | 15 | 3.59 x 10^-2^ | 5.38 x 10^-1^ |
| *STX12* | 10 | 2.03 x 10^-1^ | 9.35 x 10^-1^ |
| *STX18* | 47 | 3.23 x 10^-3^ | 1.24 x 10^-1^ |
| *STX1A* | 15 | 6.60 x 10^-2^ | 8.40 x 10^-1^ |
| *STX4* | 1 | 2.06 x 10^-1^ | 8.51 x 10^-2^ |
| *STX8* | 111 | 1.48 x 10^-2^ | 3.39 x 10^-1^ |
| *STXBP1* | 17 | 6.68 x 10^-2^ | 4.43 x 10^-1^ |
| *STXBP2* | 15 | 3.76 x 10^-1^ | 9.42 x 10^-1^ |
| *SUCLG1* | 10 | 3.14 x 10^-1^ | 8.27 x 10^-1^ |
| *SULT1A1* | 7 | 2.44 x 10^-1^ | 6.26 x 10^-1^ |
| *SULT2B1* | 30 | 1.11 x 10^-1^ | 8.62 x 10^-1^ |
| *SUMF2* | 8 | 1.29 x 10^-2^ | 7.64 x 10^-2^ |
| *SUMO2* | 7 | 2.69 x 10^-1^ | 9.59 x 10^-1^ |
| *SUMO3* | 35 | 1.09 x 10^-2^ | 1.42 x 10^-1^ |
| *SUPT4H1* | 29 | 2.95 x 10^-2^ | 2.86 x 10^-1^ |
| *SUPT5H* | 26 | 3.91 x 10^-2^ | 6.56 x 10^-1^ |
| *SUPT6H* | 5 | 5.49 x 10^-1^ | 8.21 x 10^-1^ |
| *SURF1* | 9 | 2.48 x 10^-1^ | 9.70 x 10^-1^ |
| *SYNGR2* | 29 | 7.12 x 10^-2^ | 6.90 x 10^-1^ |
| *SYNPO* | 20 | 8.11 x 10^-2^ | 7.88 x 10^-1^ |
| *SYPL1* | 32 | 1.95 x 10^-3^ | 3.42 x 10^-2^ |
| *SYT14L* | 9 | 2.12 x 10^-2^ | 3.06 x 10^-2^ |
| *SYT8* | 20 | 2.89 x 10^-2^ | 2.93 x 10^-1^ |
| *SYTL1* | 8 | 7.36 x 10^-3^ | 4.33 x 10^-2^ |
| *TAC3* | 9 | 6.82 x 10^-3^ | 9.92 x 10^-3^ |
| *TACC1* | 23 | 1.74 x 10^-1^ | 9.84 x 10^-1^ |
| *TACC2* | 132 | 2.01 x 10^-2^ | 7.69 x 10^-1^ |
| *TACSTD1* | 21 | 2.41 x 10^-3^ | 1.23 x 10^-2^ |
| *TACSTD2* | 21 | 1.33 x 10^-1^ | 8.82 x 10^-1^ |
| *TAF10* | 22 | 1.08 x 10^-2^ | 2.31 x 10^-1^ |
| *TAF5L* | 20 | 1.42 x 10^-1^ | 6.19 x 10^-1^ |
| *TAF6* | 13 | 1.07 x 10^-1^ | 6.62 x 10^-1^ |
| *TAGAP* | 30 | 3.44 x 10^-1^ | 9.62 x 10^-1^ |
| *TAGLN* | 17 | 3.12 x 10^-2^ | 3.55 x 10^-1^ |
| *TAGLN2* | 40 | 1.18 x 10^-1^ | 9.84 x 10^-1^ |
| *TALDO1* | 18 | 9.58 x 10^-3^ | 7.19 x 10^-2^ |
| *TAP1* | 74 | 2.76 x 10^-2^ | 8.47 x 10^-1^ |
| *TAPBP* | 20 | 3.08 x 10^-2^ | 2.36 x 10^-1^ |
| *TARBP2* | 11 | 2.13 x 10^-1^ | 8.08 x 10^-1^ |
| *TAS2R40* | 6 | 1.57 x 10^-1^ | 9.41 x 10^-1^ |
| *TATDN2* | 37 | 2.74 x 10^-1^ | 1.00 |
| *TAX1BP3* | 23 | 4.14 x 10^-2^ | 6.06 x 10^-1^ |
| *TBC1D1* | 123 | 2.67 x 10^-2^ | 8.55 x 10^-1^ |
| *TBC1D10A* | 31 | 1.51 x 10^-2^ | 4.63 x 10^-1^ |
| *TBC1D10B* | 5 | 1.89 x 10^-1^ | 8.78 x 10^-1^ |
| *TBC1D14* | 56 | 1.02 x 10^-2^ | 5.40 x 10^-1^ |
| *TBC1D20* | 27 | 2.38 x 10^-2^ | 5.02 x 10^-1^ |
| *TBC1D22A* | 147 | 1.59 x 10^-2^ | 6.40 x 10^-1^ |
| *TBC1D2B* | 31 | 6.27 x 10^-2^ | 9.34 x 10^-1^ |
| *TBC1D5* | 62 | 5.85 x 10^-2^ | 9.41 x 10^-1^ |
| *TBC1D8* | 53 | 1.04 x 10^-2^ | 4.20 x 10^-1^ |
| *TBC1D9B* | 26 | 9.30 x 10^-2^ | 9.77 x 10^-1^ |
| *TBCA* | 31 | 6.09 x 10^-2^ | 2.56 x 10^-1^ |
| *TBCB* | 14 | 9.35 x 10^-2^ | 6.79 x 10^-1^ |
| *TBCC* | 16 | 5.97 x 10^-2^ | 8.96 x 10^-1^ |
| *TBX2* | 22 | 6.95 x 10^-3^ | 1.53 x 10^-1^ |
| *TBX21* | 15 | 4.97 x 10^-2^ | 2.94 x 10^-1^ |
| *TBXAS1* | 77 | 1.39 x 10^-2^ | 3.51 x 10^-1^ |
| *TCEB1* | 24 | 2.11 x 10^-2^ | 1.07 x 10^-1^ |
| *TCEB2* | 11 | 2.22 x 10^-1^ | 7.20 x 10^-1^ |
| *TCEB3* | 12 | 2.20 x 10^-1^ | 9.62 x 10^-1^ |
| *TCF20* | 17 | 1.73 x 10^-1^ | 4.12 x 10^-1^ |
| *TCF21* | 19 | 9.47 x 10^-2^ | 7.84 x 10^-1^ |
| *TCF25* | 12 | 1.86 x 10^-1^ | 8.77 x 10^-1^ |
| *TCF7* | 22 | 3.80 x 10^-3^ | 7.99 x 10^-2^ |
| *TCF7L1* | 82 | 1.60 x 10^-2^ | 4.10 x 10^-1^ |
| *TCF7L2* | 52 | 2.83 x 10^-2^ | 8.95 x 10^-1^ |
| *TCIRG1* | 15 | 1.27 x 10^-2^ | 9.79 x 10^-2^ |
| *TCN2* | 38 | 1.56 x 10^-2^ | 4.05 x 10^-1^ |
| *TCTA* | 1 | 8.98 x 10^-1^ | 8.98 x 10^-1^ |
| *TCTN1* | 5 | 6.31 x 10^-1^ | 9.17 x 10^-1^ |
| *TCTN2* | 17 | 2.61 x 10^-2^ | 3.74 x 10^-2^ |
| *TCTN3* | 18 | 1.32 x 10^-1^ | 8.14 x 10^-1^ |
| *TDRD10* | 19 | 3.04 x 10^-3^ | 2.91 x 10^-2^ |
| *TEAD2* | 13 | 1.50 x 10^-2^ | 6.80 x 10^-2^ |
| *TECTB* | 26 | 1.67 x 10^-1^ | 7.68 x 10^-1^ |
| *TEGT* | 10 | 2.82 x 10^-1^ | 6.51 x 10^-1^ |
| *TENC1* | 23 | 9.26 x 10^-2^ | 9.06 x 10^-1^ |
| *TERF2* | 21 | 2.16 x 10^-2^ | 3.06 x 10^-1^ |
| *TERF2IP* | 10 | 1.85 x 10^-2^ | 6.82 x 10^-2^ |
| *TESK1* | 18 | 4.93 x 10^-3^ | 8.87 x 10^-2^ |
| *TESK2* | 20 | 2.32 x 10^-1^ | 9.88 x 10^-1^ |
| *TEX10* | 19 | 1.36 x 10^-1^ | 1.00 |
| *TEX261* | 25 | 1.91 x 10^-3^ | 3.76 x 10^-2^ |
| *TFDP1* | 18 | 3.23 x 10^-2^ | 5.73 x 10^-1^ |
| *TFF3* | 49 | 6.91 x 10^-4^ | 2.71 x 10^-2^ |
| *TFG* | 22 | 1.65 x 10^-2^ | 3.37 x 10^-1^ |
| *TFRC* | 24 | 3.47 x 10^-2^ | 8.34 x 10^-1^ |
| *TGFB1* | 15 | 3.32 x 10^-5^ | 2.75 x 10^-4^ |
| *TGFB1I1* | 10 | 1.58 x 10^-1^ | 8.51 x 10^-1^ |
| *TGFBI* | 38 | 3.05 x 10^-2^ | 9.58 x 10^-1^ |
| *TGFBR2* | 56 | 1.54 x 10^-2^ | 7.48 x 10^-1^ |
| *TGFBR3* | 98 | 1.56 x 10^-3^ | 7.48 x 10^-2^ |
| *TGIF1* | 43 | 5.12 x 10^-2^ | 7.87 x 10^-1^ |
| *TGM2* | 22 | 5.26 x 10^-2^ | 8.10 x 10^-1^ |
| *TGM7* | 14 | 5.49 x 10^-2^ | 3.54 x 10^-1^ |
| *TGOLN2* | 30 | 1.60 x 10^-2^ | 2.49 x 10^-1^ |
| *TH1L* | 12 | 1.28 x 10^-1^ | 2.13 x 10^-1^ |
| *THAP11* | 5 | 4.13 x 10^-1^ | 6.76 x 10^-1^ |
| *THAP4* | 10 | 1.63 x 10^-2^ | 1.63 x 10^-1^ |
| *THAP7* | 39 | 7.45 x 10^-2^ | 9.19 x 10^-1^ |
| *THBD* | 37 | 1.02 x 10^-2^ | 1.89 x 10^-1^ |
| *THEM2* | 27 | 7.19 x 10^-2^ | 9.61 x 10^-1^ |
| *TIE1* | 12 | 2.83 x 10^-2^ | 3.40 x 10^-1^ |
| *TIMM10* | 10 | 1.39 x 10^-1^ | 7.67 x 10^-1^ |
| *TIMM13* | 15 | 5.84 x 10^-2^ | 3.98 x 10^-1^ |
| *TIMM8B* | 8 | 1.88 x 10^-1^ | 3.59 x 10^-1^ |
| *TIMM9* | 9 | 3.20 x 10^-2^ | 2.48 x 10^-1^ |
| *TIMP2* | 42 | 3.69 x 10^-2^ | 9.76 x 10^-1^ |
| *TIMP3* | 71 | 4.19 x 10^-2^ | 9.86 x 10^-1^ |
| *TINAGL1* | 12 | 1.92 x 10^-2^ | 5.72 x 10^-2^ |
| *TINF2* | 17 | 2.60 x 10^-2^ | 2.55 x 10^-1^ |
| *TIPARP* | 24 | 2.28 x 10^-1^ | 9.50 x 10^-1^ |
| *TJAP1* | 14 | 1.27 x 10^-1^ | 4.71 x 10^-1^ |
| *TJP1* | 32 | 1.70 x 10^-3^ | 4.38 x 10^-2^ |
| *TKT* | 26 | 3.23 x 10^-2^ | 5.11 x 10^-1^ |
| *TLE1* | 58 | 5.49 x 10^-3^ | 2.83 x 10^-1^ |
| *TLE2* | 26 | 6.30 x 10^-2^ | 8.92 x 10^-1^ |
| *TM2D3* | 17 | 1.23 x 10^-1^ | 4.16 x 10^-1^ |
| *TM4SF1* | 30 | 3.33 x 10^-2^ | 7.74 x 10^-1^ |
| *TM7SF2* | 17 | 1.46 x 10^-1^ | 1.00 |
| *TM9SF1* | 19 | 1.06 x 10^-1^ | 5.37 x 10^-1^ |
| *TM9SF2* | 50 | 1.27 x 10^-4^ | 4.31 x 10^-3^ |
| *TM9SF4* | 16 | 1.37 x 10^-1^ | 9.53 x 10^-1^ |
| *TMBIM1* | 12 | 6.15 x 10^-2^ | 5.47 x 10^-1^ |
| *TMBIM4* | 20 | 1.82 x 10^-1^ | 8.67 x 10^-1^ |
| *TMC4* | 28 | 3.71 x 10^-2^ | 8.54 x 10^-1^ |
| *TMC6* | 29 | 9.61 x 10^-2^ | 8.54 x 10^-1^ |
| *TMED10* | 13 | 2.27 x 10^-1^ | 4.79 x 10^-1^ |
| *TMED2* | 14 | 5.51 x 10^-2^ | 2.16 x 10^-1^ |
| *TMED3* | 22 | 5.53 x 10^-2^ | 8.59 x 10^-1^ |
| *TMED6* | 15 | 2.16 x 10^-2^ | 2.95 x 10^-1^ |
| *TMED9* | 7 | 2.59 x 10^-1^ | 9.16 x 10^-1^ |
| *TMEM100* | 26 | 8.90 x 10^-2^ | 8.18 x 10^-1^ |
| *TMEM109* | 17 | 5.56 x 10^-2^ | 6.79 x 10^-1^ |
| *TMEM11* | 16 | 1.28 x 10^-1^ | 7.11 x 10^-1^ |
| *TMEM115* | 7 | 1.45 x 10^-1^ | 7.65 x 10^-1^ |
| *TMEM120A* | 13 | 3.21 x 10^-1^ | 9.12 x 10^-1^ |
| *TMEM125* | 18 | 5.48 x 10^-2^ | 3.82 x 10^-1^ |
| *TMEM128* | 22 | 4.56 x 10^-2^ | 3.55 x 10^-1^ |
| *TMEM134* | 11 | 1.56 x 10^-2^ | 9.81 x 10^-2^ |
| *TMEM139* | 24 | 1.47 x 10^-3^ | 3.54 x 10^-2^ |
| *TMEM140* | 13 | 1.59 x 10^-1^ | 8.90 x 10^-1^ |
| *TMEM141* | 13 | 1.24 x 10^-1^ | 7.96 x 10^-1^ |
| *TMEM147* | 25 | 6.52 x 10^-2^ | 8.60 x 10^-1^ |
| *TMEM14A* | 37 | 1.76 x 10^-1^ | 9.32 x 10^-1^ |
| *TMEM14B* | 14 | 8.37 x 10^-2^ | 4.55 x 10^-1^ |
| *TMEM160* | 7 | 4.49 x 10^-2^ | 3.06 x 10^-1^ |
| *TMEM161A* | 18 | 9.34 x 10^-2^ | 9.82 x 10^-1^ |
| *TMEM174* | 26 | 1.03 x 10^-1^ | 8.64 x 10^-1^ |
| *TMEM175* | 19 | 3.33 x 10^-1^ | 8.48 x 10^-1^ |
| *TMEM176A* | 36 | 4.77 x 10^-3^ | 8.83 x 10^-2^ |
| *TMEM176B* | 39 | 4.77 x 10^-3^ | 9.49 x 10^-2^ |
| *TMEM182* | 23 | 6.76 x 10^-3^ | 4.16 x 10^-2^ |
| *TMEM183A* | 22 | 9.78 x 10^-2^ | 5.19 x 10^-1^ |
| *TMEM184B* | 28 | 1.68 x 10^-2^ | 4.32 x 10^-1^ |
| *TMEM203* | 1 | 9.55 x 10^-1^ | 9.55 x 10^-1^ |
| *TMEM204* | 31 | 6.96 x 10^-2^ | 8.36 x 10^-1^ |
| *TMEM208* | 8 | 2.87 x 10^-1^ | 9.51 x 10^-1^ |
| *TMEM214* | 5 | 2.27 x 10^-1^ | 9.20 x 10^-1^ |
| *TMEM222* | 8 | 7.36 x 10^-3^ | 4.62 x 10^-2^ |
| *TMEM41A* | 7 | 3.94 x 10^-1^ | 8.90 x 10^-1^ |
| *TMEM43* | 20 | 4.50 x 10^-2^ | 4.55 x 10^-1^ |
| *TMEM49* | 15 | 5.58 x 10^-2^ | 8.37 x 10^-1^ |
| *TMEM50A* | 1 | 6.04 x 10^-1^ | 6.04 x 10^-1^ |
| *TMEM53* | 18 | 2.77 x 10^-1^ | 9.57 x 10^-1^ |
| *TMEM55B* | 34 | 1.30 x 10^-3^ | 4.38 x 10^-2^ |
| *TMEM59* | 12 | 6.56 x 10^-2^ | 6.20 x 10^-1^ |
| *TMEM63B* | 28 | 1.95 x 10^-3^ | 3.74 x 10^-2^ |
| *TMEM66* | 20 | 1.40 x 10^-2^ | 1.60 x 10^-1^ |
| *TMEM70* | 20 | 3.25 x 10^-2^ | 1.03 x 10^-1^ |
| *TMEM86A* | 27 | 8.33 x 10^-2^ | 9.20 x 10^-1^ |
| *TMEM87A* | 15 | 1.31 x 10^-1^ | 5.43 x 10^-1^ |
| *TMEM93* | 20 | 4.14 x 10^-2^ | 6.26 x 10^-1^ |
| *TMEM97* | 14 | 4.14 x 10^-2^ | 5.80 x 10^-1^ |
| *TMEM98* | 20 | 5.60 x 10^-2^ | 1.00 |
| *TMEM9B* | 18 | 4.65 x 10^-1^ | 1.00 |
| *TMOD4* | 12 | 9.45 x 10^-2^ | 4.33 x 10^-1^ |
| *TMPRSS2* | 49 | 1.54 x 10^-2^ | 6.33 x 10^-1^ |
| *TMSB10* | 14 | 4.80 x 10^-2^ | 4.64 x 10^-1^ |
| *TNFAIP1* | 11 | 1.58 x 10^-1^ | 9.46 x 10^-1^ |
| *TNFAIP2* | 16 | 2.89 x 10^-2^ | 4.46 x 10^-1^ |
| *TNFAIP3* | 17 | 4.25 x 10^-2^ | 4.52 x 10^-1^ |
| *TNFAIP8L1* | 19 | 9.54 x 10^-2^ | 8.94 x 10^-1^ |
| *TNFAIP8L2* | 13 | 9.45 x 10^-2^ | 4.72 x 10^-1^ |
| *TNFRSF12A* | 14 | 1.79 x 10^-1^ | 8.43 x 10^-1^ |
| *TNFRSF14* | 9 | 1.04 x 10^-1^ | 7.41 x 10^-1^ |
| *TNFRSF1A* | 21 | 1.77 x 10^-1^ | 8.61 x 10^-1^ |
| *TNFSF13* | 4 | 4.38 x 10^-1^ | 7.79 x 10^-1^ |
| *TNIP1* | 76 | 4.22 x 10^-3^ | 3.03 x 10^-1^ |
| *TNIP2* | 15 | 2.94 x 10^-2^ | 3.54 x 10^-1^ |
| *TNK1* | 23 | 2.60 x 10^-2^ | 5.60 x 10^-1^ |
| *TNNC1* | 9 | 1.93 x 10^-1^ | 1.00 |
| *TNPO1* | 16 | 1.59 x 10^-1^ | 5.37 x 10^-1^ |
| *TNPO2* | 9 | 3.20 x 10^-2^ | 2.88 x 10^-1^ |
| *TNS1* | 82 | 5.79 x 10^-2^ | 9.88 x 10^-1^ |
| *TNS3* | 98 | 5.20 x 10^-2^ | 9.45 x 10^-1^ |
| *TNXB* | 22 | 4.52 x 10^-2^ | 5.34 x 10^-1^ |
| *TOB1* | 14 | 3.11 x 10^-1^ | 9.55 x 10^-1^ |
| *TOB2* | 10 | 7.88 x 10^-2^ | 6.38 x 10^-1^ |
| *TOM1* | 35 | 4.82 x 10^-2^ | 7.57 x 10^-1^ |
| *TOMM20* | 7 | 9.04 x 10^-4^ | 3.34 x 10^-3^ |
| *TOMM34* | 24 | 6.82 x 10^-5^ | 1.01 x 10^-3^ |
| *TOMM40* | 19 | 7.24 x 10^-3^ | 1.27 x 10^-1^ |
| *TOMM7* | 38 | 7.37 x 10^-2^ | 6.38 x 10^-1^ |
| *TOR1B* | 28 | 2.40 x 10^-2^ | 5.19 x 10^-1^ |
| *TOR3A* | 20 | 2.68 x 10^-2^ | 1.34 x 10^-1^ |
| *TOX2* | 76 | 3.14 x 10^-2^ | 5.40 x 10^-1^ |
| *TP53I3* | 9 | 3.41 x 10^-3^ | 2.01 x 10^-2^ |
| *TPCN1* | 17 | 8.96 x 10^-3^ | 1.26 x 10^-1^ |
| *TPCN2* | 32 | 8.04 x 10^-3^ | 2.34 x 10^-1^ |
| *TPD52L1* | 44 | 8.23 x 10^-3^ | 4.56 x 10^-2^ |
| *TPD52L2* | 18 | 4.72 x 10^-3^ | 3.72 x 10^-2^ |
| *TPI1* | 19 | 1.97 x 10^-2^ | 1.62 x 10^-1^ |
| *TPM1* | 35 | 1.62 x 10^-1^ | 8.36 x 10^-1^ |
| *TPP1* | 26 | 1.08 x 10^-2^ | 2.76 x 10^-1^ |
| *TPSAB1* | 15 | 5.53 x 10^-3^ | 5.30 x 10^-2^ |
| *TPSB2* | 13 | 5.53 x 10^-3^ | 5.27 x 10^-2^ |
| *TPST2* | 54 | 2.38 x 10^-2^ | 3.69 x 10^-1^ |
| *TPT1* | 10 | 1.20 x 10^-1^ | 8.67 x 10^-1^ |
| *TRADD* | 7 | 1.56 x 10^-1^ | 7.78 x 10^-1^ |
| *TRAK1* | 48 | 3.45 x 10^-2^ | 9.66 x 10^-1^ |
| *TRAM1* | 19 | 9.15 x 10^-3^ | 1.74 x 10^-1^ |
| *TRAP1* | 29 | 6.53 x 10^-2^ | 7.75 x 10^-1^ |
| *TRAPPC3* | 17 | 2.59 x 10^-3^ | 4.02 x 10^-2^ |
| *TRAPPC4* | 15 | 9.45 x 10^-2^ | 9.88 x 10^-1^ |
| *TRAPPC6A* | 13 | 1.38 x 10^-1^ | 6.73 x 10^-1^ |
| *TRAPPC9* | 232 | 1.64 x 10^-2^ | 9.24 x 10^-1^ |
| *TREM1* | 43 | 4.60 x 10^-2^ | 4.98 x 10^-1^ |
| *TRIAP1* | 9 | 9.48 x 10^-2^ | 4.11 x 10^-1^ |
| *TRIB1* | 35 | 7.72 x 10^-3^ | 9.88 x 10^-2^ |
| *TRIB3* | 36 | 2.38 x 10^-2^ | 2.53 x 10^-1^ |
| *TRIM11* | 14 | 5.17 x 10^-1^ | 9.40 x 10^-1^ |
| *TRIM21* | 35 | 3.20 x 10^-2^ | 5.55 x 10^-1^ |
| *TRIM22* | 52 | 2.39 x 10^-2^ | 3.55 x 10^-1^ |
| *TRIM26* | 32 | 8.37 x 10^-2^ | 6.45 x 10^-1^ |
| *TRIM27* | 16 | 2.69 x 10^-1^ | 7.55 x 10^-1^ |
| *TRIM28* | 9 | 4.44 x 10^-1^ | 9.76 x 10^-1^ |
| *TRIM32* | 24 | 4.29 x 10^-3^ | 1.03 x 10^-1^ |
| *TRIM44* | 36 | 1.89 x 10^-1^ | 9.39 x 10^-1^ |
| *TRIM56* | 19 | 4.09 x 10^-2^ | 2.83 x 10^-1^ |
| *TRIM8* | 16 | 3.44 x 10^-2^ | 4.49 x 10^-1^ |
| *TRIOBP* | 27 | 2.87 x 10^-2^ | 1.96 x 10^-1^ |
| *TRIP10* | 30 | 2.48 x 10^-2^ | 7.45 x 10^-1^ |
| *TRIP6* | 15 | 1.60 x 10^-2^ | 1.51 x 10^-1^ |
| *TRMT5* | 6 | 2.15 x 10^-1^ | 7.08 x 10^-1^ |
| *TRPV2* | 19 | 2.80 x 10^-1^ | 9.76 x 10^-1^ |
| *TSC22D1* | 34 | 1.09 x 10^-1^ | 9.52 x 10^-1^ |
| *TSEN34* | 19 | 2.59 x 10^-1^ | 8.58 x 10^-1^ |
| *TSG101* | 15 | 5.73 x 10^-2^ | 3.47 x 10^-1^ |
| *TSHZ1* | 58 | 2.72 x 10^-2^ | 6.29 x 10^-1^ |
| *TSPAN1* | 10 | 3.28 x 10^-1^ | 9.48 x 10^-1^ |
| *TSPAN13* | 62 | 1.81 x 10^-2^ | 6.96 x 10^-1^ |
| *TSPAN14* | 29 | 6.70 x 10^-3^ | 3.80 x 10^-2^ |
| *TSPAN15* | 47 | 7.40 x 10^-3^ | 2.42 x 10^-1^ |
| *TSPAN3* | 17 | 1.26 x 10^-1^ | 9.63 x 10^-1^ |
| *TSPAN31* | 12 | 5.23 x 10^-3^ | 1.85 x 10^-2^ |
| *TSPAN4* | 10 | 9.58 x 10^-3^ | 1.59 x 10^-2^ |
| *TSPO* | 40 | 2.41 x 10^-2^ | 3.62 x 10^-1^ |
| *TSPYL1* | 9 | 1.96 x 10^-1^ | 4.84 x 10^-1^ |
| *TSSK3* | 7 | 9.09 x 10^-2^ | 4.53 x 10^-1^ |
| *TST* | 27 | 5.61 x 10^-2^ | 9.63 x 10^-1^ |
| *TSTA3* | 23 | 9.88 x 10^-2^ | 5.53 x 10^-1^ |
| *TTC19* | 15 | 2.14 x 10^-2^ | 1.44 x 10^-1^ |
| *TTC39C* | 51 | 3.50 x 10^-3^ | 6.56 x 10^-2^ |
| *TTLL12* | 46 | 2.15 x 10^-2^ | 2.37 x 10^-1^ |
| *TTPAL* | 14 | 3.18 x 10^-2^ | 2.19 x 10^-1^ |
| *TUBA1A* | 6 | 1.15 x 10^-1^ | 3.55 x 10^-1^ |
| *TUBA1B* | 3 | 2.27 x 10^-1^ | 6.51 x 10^-1^ |
| *TUBA1C* | 12 | 1.15 x 10^-1^ | 7.10 x 10^-1^ |
| *TUBA3C* | 16 | 5.36 x 10^-2^ | 7.50 x 10^-1^ |
| *TUBA4A* | 14 | 1.76 x 10^-1^ | 8.68 x 10^-1^ |
| *TUBB* | 23 | 2.38 x 10^-2^ | 1.67 x 10^-1^ |
| *TUBB2A* | 16 | 3.54 x 10^-2^ | 5.27 x 10^-1^ |
| *TUBB4* | 20 | 1.16 x 10^-2^ | 6.34 x 10^-2^ |
| *TUBB6* | 18 | 5.73 x 10^-2^ | 8.67 x 10^-1^ |
| *TUFM* | 5 | 2.50 x 10^-1^ | 2.88 x 10^-1^ |
| *TWF2* | 7 | 4.01 x 10^-1^ | 9.27 x 10^-1^ |
| *TXN* | 37 | 1.44 x 10^-1^ | 9.89 x 10^-1^ |
| *TXN2* | 26 | 1.71 x 10^-3^ | 3.20 x 10^-2^ |
| *TXNDC15* | 15 | 1.82 x 10^-1^ | 7.46 x 10^-1^ |
| *TXNDC17* | 18 | 3.46 x 10^-2^ | 6.24 x 10^-1^ |
| *TXNDC4* | 14 | 1.37 x 10^-1^ | 3.78 x 10^-1^ |
| *TXNDC5* | 84 | 7.74 x 10^-3^ | 4.53 x 10^-1^ |
| *TXNIP* | 10 | 2.92 x 10^-1^ | 7.13 x 10^-1^ |
| *TXNL1* | 17 | 3.18 x 10^-2^ | 5.16 x 10^-1^ |
| *TXNL4A* | 15 | 2.00 x 10^-1^ | 1.00 |
| *TXNL4B* | 15 | 8.00 x 10^-3^ | 8.65 x 10^-2^ |
| *TXNRD1* | 35 | 2.32 x 10^-2^ | 3.82 x 10^-1^ |
| *TXNRD2* | 52 | 1.44 x 10^-1^ | 9.64 x 10^-1^ |
| *TYMP* | 24 | 3.57 x 10^-2^ | 8.21 x 10^-1^ |
| *TYROBP* | 9 | 1.40 x 10^-2^ | 4.50 x 10^-2^ |
| *TYSND1* | 33 | 2.04 x 10^-2^ | 5.81 x 10^-1^ |
| *U2AF1* | 33 | 4.45 x 10^-2^ | 8.44 x 10^-1^ |
| *UBA52* | 7 | 1.58 x 10^-1^ | 3.79 x 10^-1^ |
| *UBA7* | 8 | 4.09 x 10^-1^ | 8.75 x 10^-1^ |
| *UBAC1* | 25 | 1.93 x 10^-2^ | 2.40 x 10^-1^ |
| *UBAP1* | 17 | 1.43 x 10^-1^ | 9.49 x 10^-1^ |
| *UBAP2* | 34 | 2.33 x 10^-1^ | 1.00 |
| *UBB* | 8 | 2.99 x 10^-1^ | 9.00 x 10^-1^ |
| *UBC* | 25 | 3.79 x 10^-2^ | 2.53 x 10^-1^ |
| *UBE2C* | 18 | 1.55 x 10^-1^ | 6.09 x 10^-1^ |
| *UBE2D2* | 9 | 1.03 x 10^-2^ | 3.26 x 10^-2^ |
| *UBE2D3* | 10 | 4.10 x 10^-1^ | 8.87 x 10^-1^ |
| *UBE2E1* | 30 | 2.28 x 10^-2^ | 6.07 x 10^-1^ |
| *UBE2E3* | 20 | 6.54 x 10^-2^ | 7.03 x 10^-1^ |
| *UBE2G1* | 17 | 4.81 x 10^-2^ | 5.37 x 10^-1^ |
| *UBE2H* | 31 | 5.22 x 10^-2^ | 6.72 x 10^-1^ |
| *UBE2I* | 18 | 1.19 x 10^-2^ | 8.64 x 10^-2^ |
| *UBE2J2* | 11 | 2.05 x 10^-1^ | 7.93 x 10^-1^ |
| *UBE2L3* | 13 | 1.92 x 10^-2^ | 2.25 x 10^-1^ |
| *UBE2L6* | 17 | 3.50 x 10^-2^ | 4.32 x 10^-1^ |
| *UBE2M* | 9 | 4.44 x 10^-1^ | 9.76 x 10^-1^ |
| *UBE2Q1* | 17 | 3.55 x 10^-2^ | 4.10 x 10^-1^ |
| *UBE2R2* | 24 | 2.58 x 10^-1^ | 9.17 x 10^-1^ |
| *UBE2S* | 16 | 3.93 x 10^-1^ | 1.00 |
| *UBE2Z* | 13 | 2.39 x 10^-3^ | 1.50 x 10^-2^ |
| *UBL5* | 9 | 3.01 x 10^-1^ | 9.38 x 10^-1^ |
| *UBN1* | 29 | 8.65 x 10^-2^ | 9.22 x 10^-1^ |
| *UBQLNL* | 54 | 1.27 x 10^-2^ | 1.88 x 10^-1^ |
| *UBXN1* | 6 | 1.66 x 10^-1^ | 5.47 x 10^-1^ |
| *UBXN6* | 16 | 2.04 x 10^-1^ | 8.53 x 10^-1^ |
| *UCHL1* | 23 | 1.76 x 10^-1^ | 1.00 |
| *UCK1* | 36 | 3.40 x 10^-2^ | 1.25 x 10^-1^ |
| *UCP2* | 24 | 6.57 x 10^-2^ | 3.46 x 10^-1^ |
| *UFD1L* | 17 | 7.48 x 10^-2^ | 6.98 x 10^-1^ |
| *UGCG* | 55 | 9.66 x 10^-2^ | 9.82 x 10^-1^ |
| *UHMK1* | 18 | 2.25 x 10^-1^ | 5.62 x 10^-1^ |
| *UIMC1* | 25 | 1.11 x 10^-1^ | 8.94 x 10^-1^ |
| *UNC13B* | 28 | 1.88 x 10^-3^ | 4.30 x 10^-2^ |
| *UNC45A* | 22 | 5.14 x 10^-3^ | 9.75 x 10^-2^ |
| *UNC84B* | 15 | 1.78 x 10^-2^ | 1.72 x 10^-1^ |
| *UNC93B1* | 13 | 1.27 x 10^-2^ | 7.83 x 10^-2^ |
| *UPF1* | 12 | 1.04 x 10^-3^ | 1.06 x 10^-2^ |
| *UPK3B* | 3 | 5.90 x 10^-1^ | 8.74 x 10^-1^ |
| *UPP1* | 21 | 3.69 x 10^-2^ | 1.75 x 10^-1^ |
| *UQCR* | 8 | 1.23 x 10^-2^ | 7.47 x 10^-2^ |
| *UQCRB* | 17 | 1.30 x 10^-1^ | 4.45 x 10^-1^ |
| *UQCRC1* | 14 | 2.35 x 10^-1^ | 9.89 x 10^-1^ |
| *UQCRC2* | 4 | 3.20 x 10^-1^ | 8.82 x 10^-1^ |
| *UQCRFS1* | 19 | 5.74 x 10^-3^ | 5.46 x 10^-2^ |
| *UQCRH* | 18 | 5.26 x 10^-2^ | 3.16 x 10^-1^ |
| *UQCRQ* | 12 | 1.38 x 10^-1^ | 4.73 x 10^-1^ |
| *URG4* | 23 | 3.75 x 10^-1^ | 8.85 x 10^-1^ |
| *URM1* | 13 | 1.77 x 10^-1^ | 9.34 x 10^-1^ |
| *UROD* | 11 | 5.64 x 10^-2^ | 1.27 x 10^-1^ |
| *UROS* | 9 | 4.84 x 10^-2^ | 1.36 x 10^-1^ |
| *USF2* | 29 | 2.65 x 10^-3^ | 5.76 x 10^-2^ |
| *USMG5* | 10 | 1.08 x 10^-1^ | 1.88 x 10^-1^ |
| *USP13* | 40 | 3.47 x 10^-3^ | 9.96 x 10^-2^ |
| *USP21* | 12 | 7.05 x 10^-2^ | 2.33 x 10^-1^ |
| *USP22* | 27 | 1.25 x 10^-3^ | 8.59 x 10^-3^ |
| *USP30* | 22 | 4.92 x 10^-2^ | 8.76 x 10^-1^ |
| *USP4* | 9 | 4.03 x 10^-1^ | 8.48 x 10^-1^ |
| *USP48* | 47 | 8.53 x 10^-2^ | 8.71 x 10^-1^ |
| *UXS1* | 27 | 6.39 x 10^-2^ | 9.96 x 10^-1^ |
| *VAMP3* | 18 | 1.26 x 10^-1^ | 9.41 x 10^-1^ |
| *VAMP5* | 17 | 7.96 x 10^-2^ | 9.30 x 10^-1^ |
| *VAPA* | 43 | 9.35 x 10^-3^ | 3.10 x 10^-1^ |
| *VASP* | 20 | 1.24 x 10^-1^ | 7.69 x 10^-1^ |
| *VAT1* | 9 | 9.89 x 10^-3^ | 2.27 x 10^-2^ |
| *VAV1* | 49 | 4.96 x 10^-2^ | 8.78 x 10^-1^ |
| *VCL* | 19 | 2.32 x 10^-1^ | 9.73 x 10^-1^ |
| *VCP* | 12 | 1.30 x 10^-2^ | 1.11 x 10^-1^ |
| *VDAC1* | 18 | 3.68 x 10^-3^ | 1.91 x 10^-2^ |
| *VDAC2* | 13 | 2.41 x 10^-1^ | 8.39 x 10^-1^ |
| *VDAC3* | 5 | 3.17 x 10^-1^ | 7.55 x 10^-1^ |
| *VEGFA* | 43 | 5.98 x 10^-2^ | 9.47 x 10^-1^ |
| *VEGFB* | 13 | 6.33 x 10^-2^ | 7.37 x 10^-1^ |
| *VGLL4* | 77 | 4.22 x 10^-2^ | 9.74 x 10^-1^ |
| *VIM* | 20 | 9.10 x 10^-2^ | 9.16 x 10^-1^ |
| *VIPR1* | 23 | 6.83 x 10^-3^ | 9.44 x 10^-2^ |
| *VIT* | 86 | 7.64 x 10^-2^ | 9.84 x 10^-1^ |
| *VKORC1* | 6 | 9.82 x 10^-2^ | 1.11 x 10^-1^ |
| *VPS11* | 15 | 9.45 x 10^-2^ | 8.16 x 10^-1^ |
| *VPS18* | 16 | 3.49 x 10^-2^ | 3.12 x 10^-1^ |
| *VPS24* | 13 | 2.17 x 10^-1^ | 9.96 x 10^-1^ |
| *VPS25* | 4 | 6.24 x 10^-1^ | 9.58 x 10^-1^ |
| *VPS26B* | 20 | 8.48 x 10^-2^ | 5.87 x 10^-1^ |
| *VPS28* | 21 | 4.12 x 10^-2^ | 1.36 x 10^-1^ |
| *VPS35* | 1 | 3.03 x 10^-1^ | 1.84 x 10^-1^ |
| *VPS37C* | 23 | 1.12 x 10^-1^ | 9.82 x 10^-1^ |
| *VPS4A* | 17 | 2.16 x 10^-2^ | 2.35 x 10^-1^ |
| *VPS52* | 23 | 1.91 x 10^-3^ | 2.06 x 10^-2^ |
| *VPS72* | 13 | 9.45 x 10^-2^ | 3.99 x 10^-1^ |
| *VRK3* | 25 | 3.67 x 10^-2^ | 2.40 x 10^-1^ |
| *VSIG2* | 19 | 1.26 x 10^-1^ | 9.78 x 10^-1^ |
| *VTI1B* | 13 | 1.22 x 10^-2^ | 1.37 x 10^-1^ |
| *VWA2* | 23 | 8.50 x 10^-3^ | 5.23 x 10^-2^ |
| *VWF* | 71 | 4.84 x 10^-3^ | 2.67 x 10^-1^ |
| *WARS* | 13 | 3.57 x 10^-1^ | 9.61 x 10^-1^ |
| *WASF2* | 13 | 7.36 x 10^-3^ | 8.19 x 10^-2^ |
| *WBP1* | 4 | 8.78 x 10^-1^ | 7.20 x 10^-1^ |
| *WBP2* | 11 | 1.04 x 10^-1^ | 4.38 x 10^-1^ |
| *WBSCR16* | 1 | 8.61 x 10^-1^ | 8.61 x 10^-1^ |
| *WBSCR22* | 15 | 6.60 x 10^-2^ | 8.40 x 10^-1^ |
| *WDR1* | 39 | 7.77 x 10^-3^ | 1.42 x 10^-1^ |
| *WDR12* | 9 | 1.60 x 10^-1^ | 5.46 x 10^-1^ |
| *WDR23* | 14 | 6.56 x 10^-2^ | 6.49 x 10^-1^ |
| *WDR26* | 13 | 9.17 x 10^-3^ | 1.19 x 10^-1^ |
| *WDR34* | 4 | 1.41 x 10^-1^ | 5.65 x 10^-1^ |
| *WDR40A* | 19 | 1.79 x 10^-1^ | 9.91 x 10^-1^ |
| *WDR54* | 6 | 5.45 x 10^-1^ | 9.59 x 10^-1^ |
| *WDR6* | 5 | 1.26 x 10^-1^ | 1.92 x 10^-1^ |
| *WDR73* | 14 | 1.38 x 10^-1^ | 7.71 x 10^-1^ |
| *WDR79* | 16 | 6.06 x 10^-2^ | 7.51 x 10^-1^ |
| *WDR82* | 13 | 1.27 x 10^-1^ | 9.79 x 10^-1^ |
| *WDR85* | 7 | 1.88 x 10^-1^ | 1.00 |
| *WDTC1* | 13 | 4.44 x 10^-2^ | 5.77 x 10^-1^ |
| *WFDC1* | 65 | 1.03 x 10^-3^ | 6.06 x 10^-2^ |
| *WFDC2* | 32 | 1.09 x 10^-2^ | 3.48 x 10^-1^ |
| *WFDC5* | 18 | 1.45 x 10^-4^ | 9.11 x 10^-4^ |
| *WFDC6* | 23 | 1.69 x 10^-1^ | 1.00 |
| *WFS1* | 31 | 4.95 x 10^-2^ | 5.51 x 10^-1^ |
| *WIF1* | 20 | 8.70 x 10^-2^ | 4.03 x 10^-1^ |
| *WIPF2* | 5 | 2.37 x 10^-1^ | 9.91 x 10^-1^ |
| *WIPI2* | 18 | 5.42 x 10^-2^ | 7.19 x 10^-1^ |
| *WISP2* | 18 | 1.39 x 10^-1^ | 9.47 x 10^-1^ |
| *WNT3A* | 13 | 5.20 x 10^-2^ | 6.64 x 10^-1^ |
| *WWOX* | 480 | 5.32 x 10^-4^ | 2.41 x 10^-1^ |
| *WWP2* | 29 | 5.78 x 10^-2^ | 4.42 x 10^-1^ |
| *XBP1* | 11 | 2.25 x 10^-2^ | 2.31 x 10^-1^ |
| *XDH* | 51 | 6.19 x 10^-2^ | 9.32 x 10^-1^ |
| *XIRP1* | 28 | 2.05 x 10^-2^ | 5.75 x 10^-1^ |
| *XKR8* | 19 | 3.35 x 10^-1^ | 8.85 x 10^-1^ |
| *XPC* | 29 | 1.87 x 10^-1^ | 9.19 x 10^-1^ |
| *XPO6* | 22 | 1.20 x 10^-1^ | 9.55 x 10^-1^ |
| *XRCC6* | 7 | 2.63 x 10^-1^ | 7.52 x 10^-1^ |
| *YARS* | 16 | 7.19 x 10^-3^ | 6.94 x 10^-2^ |
| *YBX1* | 19 | 1.05 x 10^-2^ | 4.16 x 10^-2^ |
| *YIF1A* | 9 | 6.79 x 10^-2^ | 5.53 x 10^-1^ |
| *YIPF2* | 6 | 1.58 x 10^-1^ | 6.04 x 10^-1^ |
| *YIPF3* | 10 | 1.34 x 10^-1^ | 5.28 x 10^-1^ |
| *YPEL3* | 9 | 1.84 x 10^-1^ | 8.51 x 10^-1^ |
| *YPEL5* | 25 | 1.45 x 10^-1^ | 9.28 x 10^-1^ |
| *YTHDF1* | 19 | 1.72 x 10^-2^ | 1.19 x 10^-1^ |
| *YWHAB* | 22 | 1.92 x 10^-4^ | 3.49 x 10^-3^ |
| *YWHAE* | 33 | 9.33 x 10^-2^ | 8.38 x 10^-1^ |
| *YWHAH* | 21 | 6.54 x 10^-2^ | 9.59 x 10^-1^ |
| *YWHAQ* | 27 | 1.02 x 10^-2^ | 9.99 x 10^-2^ |
| *YY1* | 12 | 7.21 x 10^-2^ | 7.93 x 10^-1^ |
| *YY1AP1* | 3 | 9.91 x 10^-2^ | 1.92 x 10^-1^ |
| *ZBED2* | 13 | 5.99 x 10^-2^ | 7.00 x 10^-1^ |
| *ZBED3* | 20 | 9.08 x 10^-3^ | 9.09 x 10^-2^ |
| *ZBTB12* | 19 | 6.09 x 10^-3^ | 3.97 x 10^-2^ |
| *ZBTB16* | 122 | 1.68 x 10^-3^ | 1.47 x 10^-1^ |
| *ZBTB20* | 127 | 6.25 x 10^-3^ | 7.88 x 10^-1^ |
| *ZBTB4* | 20 | 1.23 x 10^-3^ | 2.18 x 10^-2^ |
| *ZBTB45* | 9 | 3.28 x 10^-1^ | 9.76 x 10^-1^ |
| *ZBTB5* | 37 | 8.45 x 10^-2^ | 9.58 x 10^-1^ |
| *ZC3H11A* | 25 | 1.68 x 10^-1^ | 8.08 x 10^-1^ |
| *ZC3H3* | 51 | 1.02 x 10^-1^ | 9.14 x 10^-1^ |
| *ZCCHC17* | 19 | 5.21 x 10^-3^ | 1.92 x 10^-2^ |
| *ZCCHC24* | 41 | 1.32 x 10^-1^ | 9.76 x 10^-1^ |
| *ZDHHC16* | 15 | 1.44 x 10^-1^ | 4.39 x 10^-1^ |
| *ZDHHC4* | 8 | 8.17 x 10^-3^ | 4.54 x 10^-2^ |
| *ZDHHC5* | 12 | 1.04 x 10^-1^ | 4.87 x 10^-1^ |
| *ZDHHC7* | 38 | 7.63 x 10^-2^ | 9.93 x 10^-1^ |
| *ZDHHC8* | 27 | 6.89 x 10^-2^ | 9.96 x 10^-1^ |
| *ZFAND3* | 65 | 2.74 x 10^-3^ | 3.96 x 10^-2^ |
| *ZFAND5* | 20 | 8.57 x 10^-3^ | 4.69 x 10^-2^ |
| *ZFAND6* | 42 | 5.34 x 10^-3^ | 1.77 x 10^-1^ |
| *ZFP36* | 20 | 3.91 x 10^-2^ | 5.18 x 10^-1^ |
| *ZFP36L1* | 33 | 9.35 x 10^-3^ | 1.11 x 10^-1^ |
| *ZFP36L2* | 24 | 1.41 x 10^-2^ | 1.74 x 10^-1^ |
| *ZFYVE1* | 27 | 2.01 x 10^-1^ | 9.75 x 10^-1^ |
| *ZFYVE19* | 11 | 1.14 x 10^-1^ | 5.85 x 10^-1^ |
| *ZFYVE27* | 25 | 1.22 x 10^-1^ | 8.24 x 10^-1^ |
| *ZMAT2* | 8 | 3.13 x 10^-1^ | 9.11 x 10^-1^ |
| *ZMIZ1* | 57 | 4.58 x 10^-2^ | 7.40 x 10^-1^ |
| *ZMIZ2* | 9 | 4.10 x 10^-1^ | 9.20 x 10^-1^ |
| *ZMYND12* | 19 | 2.83 x 10^-3^ | 1.42 x 10^-2^ |
| *ZNF160* | 43 | 2.13 x 10^-2^ | 4.45 x 10^-1^ |
| *ZNF169* | 21 | 1.13 x 10^-1^ | 9.26 x 10^-1^ |
| *ZNF19* | 19 | 2.43 x 10^-2^ | 3.62 x 10^-1^ |
| *ZNF207* | 7 | 3.82 x 10^-1^ | 9.70 x 10^-1^ |
| *ZNF219* | 36 | 6.04 x 10^-3^ | 9.23 x 10^-2^ |
| *ZNF263* | 14 | 8.47 x 10^-2^ | 4.41 x 10^-1^ |
| *ZNF274* | 26 | 1.17 x 10^-2^ | 1.10 x 10^-1^ |
| *ZNF331* | 41 | 2.81 x 10^-2^ | 4.37 x 10^-1^ |
| *ZNF337* | 9 | 9.41 x 10^-2^ | 4.10 x 10^-1^ |
| *ZNF341* | 20 | 1.33 x 10^-2^ | 2.60 x 10^-1^ |
| *ZNF366* | 33 | 9.22 x 10^-2^ | 9.59 x 10^-1^ |
| *ZNF395* | 24 | 7.43 x 10^-3^ | 1.63 x 10^-1^ |
| *ZNF398* | 22 | 2.39 x 10^-3^ | 3.93 x 10^-2^ |
| *ZNF414* | 18 | 3.45 x 10^-2^ | 3.65 x 10^-1^ |
| *ZNF444* | 24 | 1.97 x 10^-1^ | 5.45 x 10^-1^ |
| *ZNF576* | 18 | 1.75 x 10^-2^ | 2.66 x 10^-1^ |
| *ZNF579* | 15 | 8.60 x 10^-2^ | 6.55 x 10^-1^ |
| *ZNF593* | 24 | 6.70 x 10^-2^ | 9.22 x 10^-1^ |
| *ZNF611* | 22 | 5.36 x 10^-2^ | 3.55 x 10^-1^ |
| *ZNF622* | 29 | 9.15 x 10^-2^ | 5.43 x 10^-1^ |
| *ZNF629* | 4 | 3.78 x 10^-1^ | 6.85 x 10^-1^ |
| *ZNF672* | 13 | 3.64 x 10^-3^ | 3.00 x 10^-2^ |
| *ZNF692* | 12 | 3.64 x 10^-3^ | 2.61 x 10^-2^ |
| *ZNF7* | 13 | 5.64 x 10^-2^ | 6.77 x 10^-1^ |
| *ZNF747* | 14 | 4.96 x 10^-2^ | 4.52 x 10^-1^ |
| *ZNF8* | 27 | 3.72 x 10^-2^ | 5.43 x 10^-1^ |
| *ZNFX1* | 21 | 2.10 x 10^-2^ | 1.94 x 10^-1^ |
| *ZNHIT1* | 12 | 3.59 x 10^-2^ | 1.57 x 10^-1^ |
| *ZNHIT3* | 11 | 5.12 x 10^-1^ | 9.44 x 10^-1^ |
| *ZRANB2* | 11 | 1.45 x 10^-1^ | 5.78 x 10^-1^ |
| *ZSCAN18* | 21 | 1.83 x 10^-2^ | 3.69 x 10^-1^ |
| *ZYX* | 22 | 9.31 x 10^-3^ | 1.80 x 10^-1^ |
